# Supplementary material for: The Iflaviruses Sacbrood virus and Deformed wing virus evoke different transcriptional responses in the honeybee which may facilitate their horizontal or vertical transmission
Source: PeerJ. 2016 Jan 18;4:e1591. doi: 10.7717/peerj.1591 (PMC4727977; doi:10.7717/peerj.1591)
Supplement: Table S1 [file peerj-04-1591-s001.pdf]

Table S1. Oligonucleotides used in this study.

Primer ID	Sequence (5'–3)	Description	GenBank accession of the target sequence
59	GTTTGTATGAGGTTATACTTCAAGGAG	Deformed wing virus, 8004-8030, For	AJ489744
60	GCCATGCAATCCTTCAGTACCAGC	Deformed wing virus, 8143-8120, Rev	AJ489744
498	GATACAGTGGACTCTTATACC	Sacbrood virus, 371-391, For	AF092924
499	GATTCTTCGTCCACTCTCATCAC	Sacbrood virus, 481-459, Rev	AF092924
508	TCCTACGGGAGGCAGCAGT	Universal bacterial rRNA primer, For	
509	GGACTACCAGGGTATCTAATCCTGTT	Universal bacterial rRNA primer, Rev	
147	CAAAAAAACTCGTCATATGTTGCCAACTG	Rp49, GB47740, Apis mellifera, For	NM_001011587
148	GCATCATTAAAACTTCCAGTTCCTTG	Rp49, GB47740, Apis mellifera, Rev	NM_001011587
500	CCGACTCGTTTCCGACGAC	Hymenoptaecin, GB51223, Apis mellifera, For	NM_001011615
501	CGTCTCCTGTCATTCCATTC	Hymenoptaecin, GB51223, Apis mellifera, Rev	NM_001011615
502	GCATTTTGAGAATGAAGAACG	Defensin 1, GB41428, Apis mellifera, For	NM_001011616
503	CAAACTGAGACAGTTAGCAG	Defensin 1, GB41428, , Apis mellifera, Rev	NM_001011616
504	TTACTTCCGTTCTGCTACTC	Serpin, GB48820, Apis mellifera, For	XM_003251765
505	GTTGTCATTTCATTCAAATCACC	Serpin, GB48820, Apis mellifera, Rev	XM_003251765
506	GTCCTCATCACGGATAGAC	PPAE, GB50013, Apis mellifera, For	XM_001121888
507	TAATTTCAACGATGGAGAAGTC	PPAE, GB50013, Apis mellifera, Rev	XM_001121888
