# Supplementary material for: The Iflaviruses Sacbrood virus and Deformed wing virus evoke different transcriptional responses in the honeybee which may facilitate their horizontal or vertical transmission
Source: PeerJ. 2016 Jan 18;4:e1591. doi: 10.7717/peerj.1591 (PMC4727977; doi:10.7717/peerj.1591)
Supplement: Table S2 [file peerj-04-1591-s002.pdf]

Table S2
Differentially expressed genes in the orally infected honeybees (DESeq, P adj < 0.05 and edgeR, FDR <0.05)

**Contrast 1<br>High SBV+DWV and high DWV vs. control**				
**Gene Label**	**logFC**	**logCPM**	**FDR**	**Flybase ortholog**
GB49888	4.782291949	4.920483398	0.000839692	FBgn0000473
GB41806	2.448766608	7.067372554	0.036597941	FBgn0038088
GB50550	2.368293243	7.695759508	0.047735983	0
GB48148	3.178511546	7.011996701	0.048993407	FBgn0034140

**Contrast 2<br>High SBV+DWV vs. high DWV and control**				
**Gene Label**	**logFC**	**logCPM**	**FDR**	**Flybase ortholog**
GB47618	10.170755	6.023045369	6.72846E-36	FBgn0010385
GB50423	9.917126921	9.276559483	3.08696E-56	FBgn0052055
GB42310	9.851545633	2.883747954	1.23532E-11	FBgn0045827
GB48823	9.764361824	8.088295566	9.56317E-51	FBgn0004778
GB50363	9.53997851	2.584894239	4.29986E-09	FBgn0038642
GB41428	9.20267233	8.297256453	5.1447E-42	FBgn0010385
GB47520	8.994094874	3.841783073	6.81639E-18	FBgn0045827
GB41637	8.722966732	6.714678486	1.17332E-48	FBgn0030905
GB40298	8.683571285	1.674812148	3.37206E-05	FBgn0035132
GB51126	8.590695782	1.398736265	2.08567E-05	FBgn0036009
GB54001	8.590695782	1.437538685	3.05373E-05	FBgn0036481
GB45912	8.431672479	10.27712242	2.82631E-56	FBgn0011296
GB51306	8.321492291	5.949462992	1.34923E-40	0
GB43112	8.240072812	8.892681415	3.32565E-49	FBgn0028573
GB43007	8.10503905	2.927099397	5.16208E-10	FBgn0030592
GB55435	7.947090212	4.82018636	1.37977E-27	FBgn0020626
GB47475	7.93608286	10.07591896	6.86763E-54	FBgn0011296
GB48146	7.902433092	6.014683365	8.66977E-26	FBgn0038071
GB51223	7.893658758	10.69912764	2.20263E-46	FBgn0014002
GB53798	7.88464312	8.97154533	9.53055E-34	FBgn0032132
GB41097	7.724340028	3.227935183	7.43375E-14	FBgn0051954
GB50121	7.391396136	8.723347799	2.20263E-46	FBgn0029167
GB54238	7.308477775	6.164990774	7.06203E-35	FBgn0053547
GB42623	7.266808393	1.822002775	1.12266E-06	FBgn0026427
GB45954	7.237958149	5.995057293	5.57364E-25	FBgn0053196
GB51583	7.187887728	3.648612078	3.31747E-16	FBgn0036117
GB42626	7.162694515	7.93485195	2.11238E-41	FBgn0005613
GB45910	6.975388925	11.49345499	1.67095E-46	FBgn0011296
GB48833	6.909072753	7.557638029	1.83854E-25	FBgn0004778
GB49890	6.829178349	7.06275395	8.49894E-24	FBgn0033302
GB48134	6.795703039	9.585711333	1.53101E-26	FBgn0001258
GB55515	6.739522986	7.981396027	1.16606E-27	FBgn0036262
GB49219	6.704990947	3.72959709	7.74114E-16	FBgn0031905
GB50477	6.691507392	8.378788984	1.20345E-35	FBgn0263774
GB45909	6.661537327	10.66079939	6.90655E-44	FBgn0011296
GB51174	6.551602858	9.242495221	5.86269E-39	FBgn0033661
GB49385	6.5145164	1.83927412	1.03023E-06	FBgn0051201
GB47318	6.474279228	8.614413862	2.12513E-17	FBgn0032835
GB54908	6.436678096	1.971687895	1.53892E-05	FBgn0263705
GB45906	6.366562377	10.39646993	5.25977E-39	FBgn0011296
GB46223	6.249636624	3.49555269	3.50319E-12	FBgn0034470

GB42797	6.201146741	7.622719751	9.41433E-37	FBgn0037288
GB54881	6.196789672	5.409476204	2.20484E-25	FBgn0036829
GB42540	6.123355772	7.215007867	1.28399E-36	FBgn0037329
GB43924	6.083756246	9.482472247	6.11734E-36	FBgn0263072
GB41709	6.015464	7.776472657	1.24084E-32	FBgn0030691
GB41361	5.944732329	1.846207159	1.87225E-05	FBgn0036575
GB51419	5.917372649	5.890624885	4.56684E-26	FBgn0035439
GB45907	5.804299902	9.02004699	1.40679E-34	FBgn0011296
GB40137	5.774948624	5.383954053	8.49894E-24	FBgn0038595
GB44871	5.677074883	6.55615111	2.01646E-33	FBgn0038074
GB55203	5.662836105	5.852273453	5.47113E-28	FBgn0034856
GB54139	5.644931574	4.836767889	2.97583E-21	FBgn0000299
GB42621	5.631536926	9.318730577	1.15029E-30	FBgn0016075
GB41636	5.615778672	5.485473795	1.8297E-30	FBgn0013733
GB51200	5.58305334	1.502228608	7.24634E-05	FBgn0005658
GB41284	5.524559683	6.353795394	3.21163E-29	FBgn0050197
GB40227	5.485685121	3.556384482	1.91266E-13	FBgn0036316
GB49888	5.470604474	4.754204531	6.00427E-17	FBgn0000473
GB52100	5.443112965	11.06553446	4.33231E-28	FBgn0000299
GB42425	5.438445859	1.984160937	1.87141E-06	FBgn0039640
GB53860	5.416753202	8.653838562	2.03827E-26	FBgn0026575
GB41708	5.387940233	8.394160271	3.509E-28	FBgn0036597
GB41722	5.359018878	9.485742499	7.02727E-22	FBgn0033246
GB45713	5.331054057	3.139890371	1.2776E-10	FBgn0031975
GB41222	5.331054057	3.103855461	5.80359E-11	FBgn0035132
GB47546	5.322187589	4.246557067	2.49851E-15	0
GB42554	5.315828519	4.735046798	6.44542E-21	FBgn0036191
GB44633	5.309607691	2.374758102	8.22023E-08	0
GB48148	5.272996012	6.950545684	4.20548E-25	FBgn0034140
GB43027	5.134496115	3.116353181	4.40988E-09	0
GB49886	5.120366188	6.335781354	6.69832E-25	FBgn0033302
GB40288	5.096753134	4.101619891	6.68364E-11	FBgn0033302
GB55029	5.043051345	5.015171509	1.77237E-20	FBgn0031176
GB45708	5.014993323	3.414357887	1.5615E-10	FBgn0031975
GB42099	5.012639651	2.890808596	3.26696E-09	FBgn0027600
GB50880	4.97231926	3.539218914	6.08391E-11	FBgn0263968
GB43739	4.958182667	3.107138378	1.30308E-09	FBgn0035779
GB47127	4.805141038	3.454368611	1.78758E-10	FBgn0261832
GB44168	4.790425819	4.385518954	2.18311E-15	FBgn0000490
GB49442	4.783442833	2.716259579	2.88056E-07	FBgn0036237
GB52428	4.77382648	5.876290038	5.48242E-28	FBgn0037126
GB50481	4.701168499	3.437593359	3.14406E-09	FBgn0023479
GB44996	4.691371024	9.759459676	2.83094E-19	FBgn0002564
GB48820	4.681074062	10.27767152	9.67969E-23	FBgn0028985
GB52919	4.652304323	7.26697432	8.76756E-23	FBgn0036617
GB43713	4.609163142	6.19299976	7.8597E-27	FBgn0038037
GB42514	4.598304636	3.440523895	4.0663E-10	FBgn0037297
GB51218	4.590144875	4.889556171	4.62137E-18	0
GB52525	4.57176458	2.005737992	1.89416E-05	FBgn0032248
GB41706	4.564904075	4.422508374	6.55161E-14	FBgn0028533
GB49552	4.554831469	5.511767822	1.57232E-15	FBgn0035501
GB52598	4.551840161	9.161733943	6.96559E-25	FBgn0032213
GB47270	4.529763918	3.202008898	6.55764E-08	FBgn0015032
GB48391	4.525923025	3.086413862	1.4715E-06	FBgn0038135
GB42985	4.490660274	5.994748913	2.5774E-15	0
GB40708	4.47479715	8.46955382	2.33336E-23	FBgn0029507

GB54404	4.456233188	5.853795011	5.31933E-25	FBgn0051522
GB50124	4.451843597	3.115013684	2.49292E-07	0
GB47563	4.43920437	2.153727506	9.5133E-06	FBgn0035575
GB49993	4.423690504	7.151554427	4.30199E-17	FBgn0035787
GB51467	4.392680345	1.598346366	0.0002703	FBgn0034140
GB43208	4.353013558	8.532927606	8.60896E-23	FBgn0036985
GB54289	4.322586371	5.883161528	1.19679E-22	FBgn0085407
GB40148	4.315825695	4.536959532	7.18595E-11	FBgn0032810
GB42900	4.31460024	10.48182674	9.86718E-19	FBgn0261564
GB50609	4.299543038	11.13250063	4.46098E-21	FBgn0001219
GB45714	4.295096004	1.98962032	3.11926E-05	FBgn0031975
GB49825	4.293411941	1.58636751	0.001437484	FBgn0024150
GB45911	4.277710134	5.403373554	6.94648E-21	FBgn0011296
GB45913	4.238007373	11.60389423	1.94215E-22	FBgn0011296
GB56028	4.227448218	6.726762606	4.2535E-22	FBgn0036665
GB55208	4.2172577	1.966405313	0.000104648	FBgn0041712
GB52721	4.203959748	5.138952685	1.94414E-15	FBgn0002576
GB47771	4.186805063	1.4361291	0.001292755	FBgn0034745
GB49441	4.181722366	7.696658693	2.45299E-20	FBgn0003450
GB50608	4.177410797	4.638526795	7.87016E-12	FBgn0033495
GB47805	4.172485983	4.916074207	3.42664E-14	FBgn0043575
GB46587	4.156120361	8.461552804	5.67019E-18	FBgn0053998
GB55204	4.153173329	4.706978229	3.43988E-11	FBgn0039896
GB44146	4.144290488	3.997160795	9.98384E-11	FBgn0034709
GB42146	4.143776571	7.554664208	1.04197E-13	FBgn0085446
GB49848	4.134979045	1.925101533	0.00052156	FBgn0038095
GB44552	4.08291145	2.713818114	0.000969678	FBgn0005633
GB41283	4.076489707	6.455600261	1.03639E-19	FBgn0003137
GB48079	4.075277377	3.029746129	1.56536E-05	FBgn0051954
GB46230	4.074295982	2.477719565	0.000894622	FBgn0034470
GB54219	4.060353219	9.450692119	4.19452E-19	FBgn0086708
GB42410	4.052773313	5.182989959	1.13046E-12	FBgn0034638
GB49440	4.052303053	5.937498762	2.8717E-22	FBgn0039630
GB46142	4.04818933	4.547024756	1.60657E-11	FBgn0026144
GB48969	4.048154797	4.182839201	8.91184E-08	FBgn0025592
GB50526	4.042056495	5.721377747	7.8263E-21	FBgn0038652
GB55205	4.041432879	5.945069028	5.89925E-19	FBgn0039896
GB47248	3.972307954	5.374068287	4.59841E-18	FBgn0031146
GB50116	3.971997848	4.069842944	8.79961E-07	FBgn0036495
GB52348	3.934042576	3.694784956	6.0767E-09	FBgn0003162
GB41642	3.900223873	5.515701119	5.53609E-18	0
GB50005	3.885125181	8.242995382	7.33676E-16	FBgn0063923
GB42244	3.87760767	7.9334127	1.73656E-19	FBgn0043903
GB45797	3.870783836	3.904473887	2.08052E-07	FBgn0039896
GB42135	3.865503137	4.349674256	9.12916E-12	FBgn0038201
GB42053	3.863856438	4.597538307	2.05952E-11	FBgn0031381
GB55452	3.848427065	8.985308451	1.2062E-11	FBgn0052626
GB46286	3.846648648	3.634693032	6.00324E-08	FBgn0032144
GB54231	3.821795535	3.367967977	8.75755E-07	FBgn0024352
GB41418	3.811644944	4.667921915	7.74781E-12	FBgn0002526
GB55451	3.800268975	9.547457444	1.07262E-14	FBgn0002526
GB52318	3.764868534	3.201800934	1.87864E-05	FBgn0031907
GB48020	3.763125238	3.556038981	7.03927E-06	FBgn0034294
GB49544	3.761545146	2.203978122	0.00017126	FBgn0051150
GB48903	3.759454142	4.481071795	4.93549E-12	FBgn0038139
GB47580	3.753340666	5.253876826	2.23514E-15	FBgn0032817

GB55068	3.710589069	3.616612597	1.28813E-07	FBgn0085351
GB55211	3.6614321	3.387139155	1.22015E-05	FBgn0032601
GB45796	3.6614321	3.34411938	2.6497E-06	FBgn0004034
GB40021	3.651577951	10.92732312	5.34045E-17	FBgn0036124
GB46774	3.645756011	9.304639432	5.90595E-15	FBgn0031322
GB53028	3.622783286	6.307606413	6.48192E-19	FBgn0032116
GB47381	3.615821627	1.810363988	0.000743159	FBgn0053817
GB54732	3.582623332	5.054497313	8.19435E-13	FBgn0030309
GB45955	3.558645202	9.282098341	2.7896E-16	FBgn0260746
GB40136	3.547358687	3.353781563	3.83514E-06	FBgn0038595
GB51383	3.479958282	2.619720611	0.000315426	FBgn0033302
GB44203	3.473817449	3.38273504	2.16862E-05	FBgn0037465
GB43716	3.465565706	5.308057825	7.99251E-14	FBgn0038037
GB44503	3.438312692	3.729974251	5.21423E-07	FBgn0085201
GB46640	3.429629864	3.954126163	1.52942E-07	FBgn0033628
GB54942	3.427617748	4.938248802	2.19301E-11	FBgn0051720
GB54941	3.418836615	5.952880729	1.46404E-16	FBgn0051720
GB50883	3.412791801	5.672687142	3.27828E-13	FBgn0032638
GB55212	3.402314018	4.583690771	5.92347E-07	FBgn0039896
GB44995	3.384738019	2.677806985	3.40356E-05	FBgn0051008
GB43362	3.365493394	5.84155463	9.08987E-15	FBgn0030421
GB55890	3.359974425	1.928917258	0.002782661	FBgn0034275
GB46013	3.357693875	6.430152481	2.82509E-16	FBgn0032010
GB42434	3.351597754	2.938403273	0.000655624	FBgn0034580
GB44634	3.326655868	5.214218178	4.56429E-13	FBgn0243512
GB53978	3.316742099	5.454582553	1.10761E-10	FBgn0023479
GB40573	3.315897469	2.913984084	6.76919E-05	FBgn0261834
GB50761	3.308444299	3.920689255	7.22434E-07	FBgn0038485
GB43711	3.298834832	2.081957316	0.003050198	FBgn0038037
GB43728	3.292408016	5.900124046	1.53839E-16	FBgn0038037
GB48905	3.283060103	7.751561085	9.25247E-12	FBgn0010226
GB44043	3.281043651	3.43061253	3.00729E-05	FBgn0028841
GB54983	3.2705082	3.144147035	4.41113E-05	FBgn0035537
GB49887	3.26728581	3.316842995	1.10361E-05	FBgn0033304
GB47505	3.265340609	2.88807366	0.00013742	FBgn0053870
GB49672	3.250389048	4.343644032	4.08618E-09	FBgn0038842
GB54233	3.23554729	4.693895699	3.22763E-09	FBgn0030964
GB50550	3.230861847	7.671943929	6.50322E-10	0
GB52360	3.22841053	7.62444488	1.91624E-12	FBgn0034886
GB52631	3.223649093	5.631205363	3.86263E-12	FBgn0003495
GB45696	3.196782716	6.691876971	6.49189E-13	FBgn0000567
GB52920	3.184190176	4.292621035	6.80255E-08	FBgn0036618
GB42829	3.179125407	8.404264717	5.12224E-13	FBgn0034405
GB45157	3.166537226	5.060101343	2.68837E-11	FBgn0013753
GB47407	3.156560924	3.068049287	2.5735E-05	FBgn0013981
GB42217	3.135604878	3.279168864	1.63597E-05	FBgn0039755
GB48687	3.131979843	6.351160333	1.46574E-14	FBgn0243514
GB40287	3.120434473	2.97459632	0.000183772	FBgn0033302
GB48129	3.118702903	1.891102847	0.004845844	FBgn0029880
GB41202	3.111647733	5.430286693	7.66648E-12	FBgn0027600
GB47142	3.080261435	8.838866639	2.72716E-12	FBgn0030608
GB49154	3.041473786	4.969100136	2.55513E-09	FBgn0040491
GB50906	3.041420324	5.720374349	7.07967E-11	FBgn0032421
GB41623	3.00669171	3.442303117	0.000333541	FBgn0035430
GB40967	2.985745163	5.43215594	1.60103E-09	FBgn0005626
GB47940	2.974195044	8.953548048	6.06087E-12	FBgn0034096

GB50290	2.936958601	6.446802267	1.57017E-13	FBgn0033033
GB46438	2.925049009	5.67508812	2.19301E-11	FBgn0027594
GB45495	2.917008714	10.18043918	1.61318E-09	FBgn0001233
GB54097	2.894879856	8.020188106	1.25752E-10	FBgn0011672
GB44004	2.870169532	4.932291827	9.9111E-09	FBgn0034490
GB55889	2.869928777	6.059073273	6.19553E-13	FBgn0035049
GB44744	2.857066175	3.749269055	4.96442E-05	FBgn0036101
GB47939	2.856806118	4.991338673	7.03264E-09	FBgn0040296
GB45701	2.851827767	6.032038602	2.06696E-12	FBgn0027930
GB53318	2.850988765	4.603661267	2.05528E-07	FBgn0001291
GB49147	2.843415515	3.131188159	0.000988885	FBgn0026565
GB48505	2.835204063	9.933678992	2.46924E-10	FBgn0026415
GB52829	2.824059616	10.88200321	3.99869E-10	FBgn0026415
GB51650	2.817625306	4.531553085	1.076E-07	FBgn0034392
GB52642	2.80952298	6.715202391	1.82742E-10	FBgn0030251
GB42981	2.80617376	7.416934196	1.07006E-10	FBgn0040323
GB51671	2.803269154	6.270001872	4.57929E-12	FBgn0030869
GB53865	2.79304581	6.306108981	3.7489E-12	FBgn0013984
GB50156	2.783530239	5.274694612	1.00977E-09	FBgn0039225
GB53353	2.773480895	2.984395661	0.000611533	FBgn0013984
GB42433	2.770112383	4.568963085	3.11941E-07	FBgn0261797
GB41806	2.75343728	7.045997663	9.56754E-06	FBgn0038088
GB54315	2.751728706	3.270979629	0.003393493	FBgn0034426
GB53209	2.737382423	3.084312619	0.001239318	FBgn0261565
GB55030	2.735025376	8.950551586	7.68046E-10	FBgn0046706
GB48194	2.719192491	4.568835643	5.72603E-07	FBgn0039755
GB48271	2.715282471	2.255212077	0.003508414	FBgn0000210
GB55590	2.703653974	8.369812174	2.48687E-09	FBgn0025814
GB45248	2.683962215	7.425041698	6.74725E-10	FBgn0032180
GB54946	2.661195975	3.363438116	0.000325178	FBgn0040299
GB53143	2.650947545	6.431812267	2.88881E-11	FBgn0016122
GB41301	2.650123472	9.436894087	6.2303E-09	FBgn0000083
GB47279	2.629644876	3.98792693	5.0286E-05	FBgn0033696
GB48841	2.627570078	4.350610065	1.03217E-05	FBgn0038819
GB43783	2.61574732	4.677734154	5.44646E-06	FBgn0032924
GB55301	2.590598321	7.770589241	4.2672E-09	FBgn0028926
GB54541	2.589426975	6.682011189	1.89127E-09	FBgn0031973
GB49885	2.583715996	7.07468414	1.13179E-06	FBgn0033304
GB40141	2.577328318	8.168262926	2.01417E-08	FBgn0038738
GB48310	2.571972309	6.167724126	3.70719E-10	FBgn0038412
GB53077	2.567560318	4.954771456	4.62448E-07	FBgn0039055
GB55930	2.557008446	3.933140044	0.000335251	FBgn0038072
GB48634	2.556268626	8.604374314	1.53781E-07	FBgn0035438
GB44005	2.555927966	6.443953448	1.78758E-10	FBgn0034490
GB47749	2.553959482	6.593055117	7.21289E-10	FBgn0039419
GB40976	2.527773093	12.22168755	8.23576E-08	FBgn0001233
GB51345	2.524940219	8.269098065	2.27387E-08	FBgn0039151
GB43708	2.509963639	11.88310773	1.06539E-06	FBgn0015221
GB42741	2.495765962	5.229341692	6.23134E-06	FBgn0050496
GB46984	2.490306421	6.527970831	3.92223E-06	FBgn0086691
GB47931	2.479602137	5.373468002	6.73041E-07	FBgn0036316
GB50508	2.448247344	6.321935869	2.4933E-09	FBgn0243514
GB49347	2.445671913	6.974296852	1.25501E-07	FBgn0033883
GB50137	2.442375079	4.988265987	1.14632E-05	FBgn0004378
GB43784	2.434424728	5.680629812	3.19632E-08	FBgn0035770
GB52191	2.432371464	7.575076736	1.86998E-08	FBgn0051217

GB51760	2.400986674	5.906286613	3.98397E-08	FBgn0000406
GB51613	2.395385862	5.060576299	1.30042E-06	FBgn0263219
GB50226	2.395042291	11.0095476	1.58074E-07	FBgn0022355
GB44055	2.39108735	6.266150343	1.00517E-08	FBgn0000250
GB55070	2.381619521	5.845712641	5.43368E-08	FBgn0027843
GB53141	2.379051557	4.853082359	2.61849E-06	FBgn0033786
GB45023	2.360318626	7.44254863	2.76315E-07	FBgn0032783
GB49327	2.350297169	5.438236563	6.77684E-07	FBgn0039131
GB47723	2.347703712	4.197632284	0.000605	FBgn0037387
GB40119	2.343061688	4.570330715	1.48093E-05	FBgn0016013
GB43184	2.340147007	8.065669203	1.34786E-07	FBgn0030245
GB54949	2.329848218	5.342586423	2.01547E-07	FBgn0028978
GB47478	2.325750831	7.952043277	8.9471E-08	FBgn0035438
GB55302	2.313869754	9.430404939	3.9988E-07	FBgn0033644
GB41736	2.309364402	3.687827079	0.000734672	FBgn0013988
GB46612	2.276565321	4.698004041	1.71078E-05	FBgn0033936
GB46601	2.272874566	3.68173792	0.001181447	FBgn0035583
GB48344	2.26880725	4.703170319	2.09526E-05	FBgn0025631
GB43330	2.268429594	8.558057837	7.72683E-05	FBgn0024319
GB49775	2.265470376	8.901196612	1.02244E-06	FBgn0011296
GB50448	2.265266381	4.681141923	3.47721E-05	FBgn0039804
GB51724	2.263705744	3.199663661	0.003618781	FBgn0039768
GB42558	2.261601765	3.926140768	0.000522749	FBgn0041585
GB47104	2.259284642	7.837508485	2.765E-07	FBgn0034162
GB52810	2.258885461	5.730556837	6.41411E-08	FBgn0039896
GB54294	2.246653918	5.348872664	1.87415E-06	FBgn0035348
GB54947	2.246195541	4.574890343	2.97551E-05	FBgn0036235
GB45688	2.244756654	9.002339208	2.06568E-07	FBgn0033883
GB48436	2.241271011	6.464447299	5.36359E-08	FBgn0002567
GB51043	2.217630887	4.302890786	0.000189701	FBgn0023535
GB53831	2.216895026	6.953068566	5.9893E-06	FBgn0033382
GB44167	2.202146914	4.460528237	0.00015309	FBgn0033240
GB46813	2.190053447	4.877239421	6.89021E-05	FBgn0010246
GB49614	2.179500147	5.810367624	1.076E-07	FBgn0250732
GB49849	2.177731106	3.297536991	0.004628208	0
GB54367	2.176984344	4.931865344	1.63749E-05	FBgn0030884
GB55096	2.17435104	8.821110349	1.96169E-06	FBgn0002719
GB48029	2.172383813	5.813703102	1.28794E-07	FBgn0032219
GB45875	2.172046247	4.176304267	0.000637027	FBgn0035132
GB50009	2.170435938	7.654800752	8.05236E-07	FBgn0040532
GB53549	2.162601404	4.904334253	7.03404E-05	FBgn0031037
GB45700	2.153315036	9.491236108	1.03023E-06	FBgn0000533
GB44214	2.147672217	4.812380225	3.98007E-05	FBgn0014141
GB43573	2.139862277	7.612954769	6.90516E-07	FBgn0032638
GB55701	2.134870981	7.791425802	0.000131042	FBgn0036857
GB47382	2.12418177	4.419590318	0.000279698	FBgn0013981
GB51238	2.117315205	4.191243175	0.000503537	FBgn0086687
GB43731	2.115288048	10.90541882	7.37893E-05	FBgn0015222
GB44213	2.109788837	7.378667895	2.67273E-06	FBgn0014141
GB47107	2.094150347	7.917916849	1.21782E-05	FBgn0036165
GB54611	2.091958655	9.552642001	1.56536E-05	FBgn0028984
GB53221	2.077836922	4.484066296	0.000761893	0
GB50418	2.073289942	6.385467929	1.67766E-06	FBgn0262473
GB51741	2.070024134	7.079613578	1.18533E-06	FBgn0030310
GB45704	2.069882014	6.129470686	3.05325E-07	FBgn0053229
GB51665	2.068204536	7.728356844	1.50011E-06	FBgn0032074

GB55007	2.067022557	3.6535846	0.003386016	FBgn0030051
GB47849	2.063211243	6.496266772	5.57386E-07	FBgn0038516
GB42084	2.057189687	6.377104183	5.06709E-07	FBgn0035132
GB40759	2.053309136	9.337097952	3.95436E-06	FBgn0043841
GB47301	2.049912229	5.386165275	5.7056E-06	FBgn0034605
GB53847	2.039990188	6.276315551	6.52653E-07	FBgn0014906
GB46686	2.036639496	5.022211054	7.48405E-05	FBgn0033913
GB47463	2.033472188	7.078138157	1.74911E-06	FBgn0001104
GB51551	2.022671107	7.671332948	2.932E-06	FBgn0035499
GB55016	2.015039009	9.933898052	5.89985E-06	FBgn0033188
GB42692	2.013652664	4.80380271	0.000115735	FBgn0003964
GB50421	2.011218811	10.50730788	5.17874E-06	FBgn0000416
GB40508	2.001915744	5.195365538	0.000637027	FBgn0034804
GB53550	2.001824235	6.372861071	3.52013E-06	FBgn0031037
GB54426	2.000511613	6.326738387	3.56609E-05	FBgn0052512
GB49899	1.995698319	3.982703139	0.002820346	FBgn0263846
GB47055	1.994460668	5.671366836	2.20774E-05	FBgn0031307
GB48195	1.986678384	6.992252208	2.82555E-05	FBgn0086687
GB49688	1.981224174	8.623063707	8.958E-06	FBgn0004577
GB40758	1.980399639	8.512288696	1.18939E-05	FBgn0033926
GB52115	1.980309692	7.762080221	1.79369E-05	FBgn0025456
GB52074	1.977598986	7.79939581	2.77542E-05	FBgn0004654
GB50043	1.967754435	6.322874769	1.81838E-06	FBgn0259178
GB53755	1.9586701	4.962733962	0.000444741	FBgn0029690
GB48626	1.94409645	6.916313598	3.11547E-05	FBgn0031528
GB44967	1.944032405	5.316446911	0.00016793	FBgn0042094
GB42865	1.93203318	5.467417394	2.42765E-05	FBgn0036732
GB45639	1.913118164	4.054229537	0.003177791	FBgn0005612
GB52341	1.909770569	6.418208056	4.07082E-06	FBgn0013987
GB55998	1.895491263	5.910480861	5.80105E-06	FBgn0027538
GB47310	1.888525544	5.902063344	7.73085E-06	FBgn0030791
GB45052	1.887107213	7.046687224	2.09385E-05	FBgn0026376
GB47415	1.882756265	7.103185822	1.25152E-05	FBgn0031538
GB48598	1.868605019	6.247305621	5.14558E-06	FBgn0011674
GB40565	1.856550243	5.950684468	8.52467E-06	FBgn0262866
GB45676	1.85104137	5.399524606	7.26114E-05	FBgn0035617
GB52630	1.847100262	5.198503513	0.000170763	FBgn0036101
GB50136	1.838958992	8.329198314	5.87687E-05	FBgn0037007
GB55511	1.832432823	5.702755414	2.81622E-05	FBgn0031461
GB49774	1.829019529	5.16437121	0.003230288	0
GB50603	1.817929561	4.793047168	0.000942341	FBgn0050410
GB55805	1.815738598	5.456057752	7.03932E-05	FBgn0050118
GB47880	1.81382656	8.751282522	6.02078E-05	FBgn0003462
GB52278	1.804103423	5.346503788	0.000164047	FBgn0014141
GB44344	1.798871547	6.925380365	2.90034E-05	FBgn0053120
GB41807	1.794392883	7.904236813	4.55183E-05	FBgn0030740
GB42466	1.794225928	4.795281554	0.001050511	FBgn0031760
GB44803	1.787896784	6.198278695	0.000233329	FBgn0035904
GB42306	1.787112938	5.115028844	0.00119512	FBgn0262526
GB50598	1.778903397	9.077918968	0.001141836	FBgn0086254
GB43572	1.776290018	7.947394972	4.75311E-05	FBgn0015575
GB49390	1.775817621	5.509974442	0.000113082	FBgn0028341
GB52158	1.76427617	6.33798316	7.12438E-05	FBgn0031975
GB44143	1.7640494	6.201091229	0.000198236	FBgn0037022
GB42738	1.757624977	5.718424808	4.70763E-05	FBgn0011204
GB43945	1.749540016	6.01127428	3.23253E-05	FBgn0061200

GB45497	1.748879282	6.627743041	8.83117E-05	FBgn0050296
GB54051	1.733190904	5.680099731	0.000456953	FBgn0263607
GB50010	1.732095022	4.490274039	0.003550301	FBgn0035575
GB51210	1.732065067	6.653931141	7.95127E-05	FBgn0039492
GB53401	1.731397121	6.007178249	5.48911E-05	FBgn0028550
GB45617	1.716460693	6.538667488	8.22459E-05	FBgn0028343
GB49928	1.714399146	5.34350604	0.000382193	FBgn0036546
GB45736	1.707668895	7.365062243	0.000127841	FBgn0016693
GB45135	1.703829768	6.492162048	9.71413E-05	FBgn0028982
GB49607	1.701399834	9.601213078	0.00015388	FBgn0032949
GB41388	1.699024386	6.162047598	5.8805E-05	FBgn0001128
GB42329	1.696029891	7.040473434	0.000104425	FBgn0262975
GB40603	1.69108342	5.918997613	6.45889E-05	FBgn0022800
GB42141	1.690617026	9.347962448	0.00021535	FBgn0035811
GB48052	1.686912458	5.070727967	0.00117727	FBgn0035542
GB47740	1.685754429	8.212925967	0.000233818	FBgn0036995
GB47929	1.675795353	4.827065635	0.003082943	FBgn0025686
GB42057	1.652835465	4.791608361	0.003528856	FBgn0037121
GB44882	1.651434398	6.79273323	0.000173609	FBgn0035094
GB41313	1.650472941	6.845401927	0.000263175	FBgn0036501
GB41735	1.649604163	7.096156217	0.000186952	FBgn0036565
GB41604	1.648294061	7.73441778	0.0002166	FBgn0030529
GB41293	1.632654415	5.82726853	0.000381027	FBgn0014340
GB43409	1.626429055	5.214179695	0.001690623	FBgn0025679
GB50970	1.625084902	9.111121882	0.000375138	FBgn0040064
GB43188	1.614428774	4.796681965	0.003617852	FBgn0034602
GB53805	1.612422767	6.140282014	0.00013832	FBgn0038098
GB49580	1.610888253	4.91429606	0.00322602	FBgn0033452
GB45147	1.606764281	8.549596806	0.000586551	FBgn0031913
GB53412	1.605704719	7.486474519	0.001491592	FBgn0027571
GB46766	1.597559089	7.242145718	0.00045945	FBgn0034577
GB40083	1.597282859	5.691682973	0.000416149	FBgn0032358
GB42184	1.595367487	5.665690654	0.002824039	FBgn0030603
GB45350	1.592045855	5.942301023	0.000222343	FBgn0261642
GB41965	1.583874534	9.580600576	0.001922681	FBgn0031696
GB40866	1.559595822	12.86510576	0.000762282	FBgn0001219
GB46367	1.551260316	6.663269001	0.002483397	FBgn0011693
GB55103	1.545727132	5.228817945	0.002299096	FBgn0039509
GB49657	1.538412012	6.786207575	0.000760922	FBgn0004606
GB49259	1.537988613	7.384982562	0.003150315	FBgn0030872
GB55482	1.535856988	7.021534167	0.00068071	FBgn0010620
GB48883	1.526069492	5.233017677	0.002955789	FBgn0264652
GB49123	1.521792221	6.576178192	0.000620619	FBgn0035719
GB46276	1.514594721	8.102700218	0.000836527	FBgn0033799
GB42500	1.514539948	6.395734927	0.000469527	FBgn0035976
GB43920	1.511856964	5.928111097	0.000594551	FBgn0034958
GB50430	1.50728161	5.264271306	0.003038114	FBgn0263929
GB43560	1.506410201	6.240900505	0.00231805	FBgn0044050
GB42685	1.501618622	6.555539575	0.000986952	FBgn0040323
GB54331	1.496249135	9.859088462	0.00120486	FBgn0013770
GB50149	1.495672778	6.768759251	0.002178129	FBgn0040827
GB46266	1.493541603	5.372238209	0.002191942	FBgn0016126
GB48672	1.492826389	5.447754264	0.002437093	FBgn0037697
GB55440	1.483692073	6.337767301	0.000690424	FBgn0262468
GB45038	1.482910204	6.229011495	0.00285636	FBgn0037010
GB40735	1.480862391	9.444426113	0.001835419	FBgn0000064

GB42940	1.478865397	7.364357502	0.001230415	FBgn0013305
GB55544	1.475737642	6.562579203	0.001482841	FBgn0261274
GB44100	1.472893177	5.92070429	0.000543897	FBgn0013733
GB43831	1.468136193	6.483055445	0.001183882	FBgn0031069
GB40718	1.466913496	8.874727046	0.002842718	FBgn0020653
GB47565	1.466457419	9.043511933	0.003693923	FBgn0023095
GB49826	1.462634826	6.969007796	0.002603275	FBgn0037612
GB53043	1.450585424	7.480442075	0.003211338	FBgn0020762
GB42142	1.443721822	5.657492202	0.00206635	FBgn0001078
GB52702	1.443375715	7.66100419	0.002481717	FBgn0034909
GB49757	1.441503227	9.040927204	0.002814735	FBgn0037913
GB42608	1.439024111	6.106088315	0.00114104	FBgn0264294
GB48853	1.418503207	7.433255886	0.002678004	FBgn0032456
GB44751	1.413933455	7.898109424	0.003256041	FBgn0032167
GB47201	1.406328732	8.34968477	0.002566996	FBgn0004657
GB46537	1.400499563	6.119676417	0.001256338	FBgn0000477
GB52033	1.39945186	5.902646511	0.002939788	FBgn0261258
GB40232	1.392910364	10.32018289	0.004444945	FBgn0040309
GB49240	1.392169946	7.221970059	0.002677557	FBgn0012036
GB42831	1.388429699	6.18658234	0.001984336	FBgn0039633
GB40577	1.364072433	6.250439974	0.002272919	FBgn0040319
GB48812	1.363536112	7.075185278	0.003784355	FBgn0037718
GB55369	1.36273635	6.445681611	0.004420236	FBgn0033177
GB40773	1.347269385	7.295583552	0.003784405	FBgn0030485
GB44081	1.343386788	6.416317211	0.004786957	FBgn0035674
GB40578	1.335367491	6.515950853	0.003575209	FBgn0028703
GB50271	1.320932101	6.804825703	0.004211447	FBgn0035432
GB50680	1.312838156	6.420464602	0.004034301	FBgn0031662
GB42327	1.309950097	5.789586696	0.003751188	FBgn0262975
GB53046	1.285802499	6.152651306	0.003648854	FBgn0031220
GB52793	1.285062245	5.899444993	0.00464346	FBgn0029896
GB44404	-1.435116633	6.08034992	0.004978223	FBgn0050491
GB41143	-1.479951869	8.033504119	0.003871	FBgn0037001
GB51759	-1.504213001	7.531704003	0.004359259	FBgn0032601
GB49584	-1.507612003	6.941096282	0.004440768	FBgn0086712
GB46282	-1.507803419	6.433774849	0.004148398	FBgn0027587
GB43228	-1.509140964	6.073272886	0.004410925	FBgn0035372
GB47177	-1.509975403	6.57066915	0.002676086	FBgn0037549
GB49845	-1.51422555	9.012603032	0.004891313	FBgn0001311
GB55568	-1.51683811	7.18915877	0.004387039	FBgn0038742
GB40489	-1.523075033	7.891929993	0.003575209	FBgn0019957
GB41908	-1.527801948	7.038100152	0.004902616	FBgn0039936
GB43537	-1.54100915	6.353374504	0.004511808	FBgn0033907
GB41850	-1.549265131	5.943809618	0.004912707	FBgn0029167
GB51481	-1.558954779	7.013584841	0.00276635	FBgn0031464
GB50847	-1.564904367	6.454904838	0.002566307	FBgn0030093
GB51800	-1.568593981	7.923485395	0.003073144	FBgn0015903
GB49533	-1.578868578	6.463272221	0.003596178	FBgn0033109
GB49328	-1.581508088	7.074700725	0.002299455	FBgn0033464
GB48254	-1.582948705	7.827326636	0.004619207	FBgn0003174
GB42959	-1.585125239	5.968648439	0.003756183	FBgn0000183
GB52997	-1.589121875	7.760569684	0.002055785	FBgn0262110
GB46793	-1.595074695	5.864206934	0.004919148	FBgn0050185
GB45153	-1.604168181	7.985594305	0.003528292	FBgn0031771
GB42043	-1.607088438	7.023307342	0.002668701	FBgn0051159
GB42840	-1.608756882	7.633738971	0.003617852	FBgn0032305

GB43611	-1.60937482	6.881550763	0.003172085	FBgn0263705
GB43639	-1.619500245	5.84302092	0.004670707	FBgn0034939
GB50558	-1.62322435	6.204229154	0.002449158	FBgn0036135
GB41366	-1.629966817	6.835006768	0.003156038	FBgn0053196
GB40703	-1.633600807	8.369020779	0.0022068	FBgn0036715
GB45540	-1.634017284	6.600065363	0.002220576	FBgn0000097
GB42661	-1.6356107	5.95914259	0.003186785	FBgn0051759
GB41701	-1.637098473	6.925850936	0.00177605	FBgn0050122
GB55911	-1.639984779	6.798405829	0.003478868	FBgn0026059
GB45372	-1.644652128	6.20465972	0.001054891	FBgn0031821
GB50586	-1.651395851	7.12441562	0.002369677	FBgn0003319
GB49325	-1.653759466	6.074823962	0.002970179	FBgn0011604
GB48981	-1.655122082	7.245374284	0.001130791	FBgn0034517
GB46431	-1.657838884	7.54211533	0.001608526	FBgn0037249
GB41609	-1.663601982	6.207479329	0.001831816	FBgn0030631
GB55808	-1.664979951	7.906526541	0.00203265	FBgn0020306
GB50683	-1.669109942	8.098953794	0.001591201	FBgn0037855
GB53670	-1.672488383	7.272982983	0.002639764	FBgn0034087
GB51768	-1.674830946	7.16312205	0.002622195	FBgn0036333
GB42821	-1.67552522	6.505250682	0.003320013	FBgn0036581
GB47905	-1.678033174	6.151705376	0.000942707	FBgn0033205
GB42557	-1.678992279	6.323872371	0.001199027	FBgn0039203
GB52252	-1.680262105	7.786136919	0.001922681	FBgn0031107
GB43456	-1.680621802	6.248512786	0.003013537	FBgn0034476
GB43114	-1.695821146	5.668816801	0.004902616	FBgn0030508
GB43693	-1.697251505	6.046648404	0.001130791	FBgn0036910
GB55356	-1.699071876	6.953103471	0.001608526	FBgn0029835
GB43123	-1.704551462	9.661071162	0.001944427	FBgn0263132
GB53657	-1.709732967	6.847179474	0.00130215	FBgn0037093
GB49977	-1.714224357	7.384434224	0.001336822	FBgn0039709
GB42484	-1.717084354	7.192292586	0.001457984	FBgn0039640
GB43890	-1.718786949	6.555780355	0.001362353	FBgn0033431
GB43304	-1.722503006	7.07555429	0.001239164	FBgn0037963
GB50870	-1.725089492	5.885049956	0.002677979	FBgn0034001
GB41900	-1.72616803	6.187828529	0.002165808	FBgn0259110
GB46902	-1.745889037	6.087942972	0.001050511	FBgn0035146
GB43143	-1.746301611	6.265427515	0.000490246	FBgn0033495
GB48948	-1.747970567	6.430783145	0.000777496	FBgn0032649
GB51104	-1.750626544	9.222737095	0.001313616	FBgn0028371
GB55944	-1.755455185	5.490991214	0.004628208	FBgn0038323
GB50408	-1.759670954	6.802436359	0.001598834	FBgn0036726
GB50769	-1.763359175	6.263698649	0.000773598	FBgn0000008
GB44799	-1.764642613	6.312157818	0.001079143	FBgn0030060
GB55843	-1.764951866	6.102765411	0.000896008	FBgn0035253
GB53010	-1.765426052	8.257002731	0.000745233	FBgn0037579
GB55274	-1.767268212	7.098787525	0.000776066	FBgn0026374
GB49764	-1.780353478	6.894212698	0.000593019	FBgn0261277
GB40405	-1.781100853	6.979782332	0.000986952	FBgn0003159
GB44683	-1.784148237	7.75489143	0.003575209	FBgn0250838
GB45152	-1.792452824	11.23878128	0.000567913	FBgn0261341
GB50101	-1.795401907	7.437727609	0.000404963	FBgn0039594
GB45111	-1.797103179	5.707993212	0.002138404	FBgn0010772
GB50061	-1.805285973	7.604639016	0.00040695	FBgn0001321
GB49308	-1.826740009	6.852069004	0.000974881	FBgn0085638
GB43613	-1.836418129	6.924455934	0.000514174	FBgn0263705
GB48497	-1.840972035	7.996480281	0.000773598	FBgn0010470

GB46680	-1.844219151	5.633873119	0.0022068	FBgn0031849
GB44187	-1.845396641	5.402865358	0.00356123	FBgn0028387
GB55640	-1.853660483	5.322596931	0.003651181	FBgn0034500
GB55395	-1.855778751	5.51780097	0.00322602	FBgn0029837
GB53716	-1.856468757	6.208810764	0.000238984	FBgn0033887
GB54362	-1.85815393	5.768852616	0.000942639	FBgn0033633
GB48578	-1.859024455	5.236696417	0.004186065	FBgn0011297
GB43953	-1.868439803	6.485635834	0.000300404	FBgn0023094
GB40010	-1.874804951	8.68730327	0.00145277	FBgn0261836
GB45122	-1.875300304	5.462652018	0.003870123	FBgn0029514
GB41290	-1.87628515	6.933215266	0.000177706	FBgn0051224
GB44661	-1.877217456	6.730714963	0.001372182	FBgn0259173
GB41746	-1.877861347	6.366113967	0.000301021	FBgn0011592
GB48454	-1.883249327	8.229173773	0.000294432	FBgn0000108
GB42015	-1.885932566	6.461369934	0.000149322	FBgn0037838
GB48577	-1.89082913	6.741366175	0.000471896	FBgn0001083
GB47451	-1.902761867	5.765191697	0.000419864	FBgn0033633
GB55328	-1.903671875	8.133657854	0.000859545	FBgn0003721
GB43054	-1.904014235	7.486285234	0.00068071	FBgn0261259
GB47199	-1.910017635	6.567081621	0.000191263	FBgn0010222
GB54685	-1.916288677	5.569800555	0.00125024	FBgn0039356
GB53012	-1.92245026	5.726702583	0.002137544	FBgn0010473
GB40808	-1.926565032	7.796380151	0.000183624	FBgn0029688
GB45932	-1.932011733	5.270907898	0.003013677	FBgn0020440
GB47321	-1.932370193	6.380254219	0.000621295	FBgn0039111
GB49095	-1.932428317	6.804095516	0.000130116	FBgn0062413
GB46297	-1.941686294	10.84003592	0.001110771	FBgn0035736
GB51814	-1.944373311	5.27693403	0.004766666	FBgn0001112
GB54893	-1.964277255	6.067445643	0.000485309	FBgn0031251
GB43084	-1.964312021	6.672699447	0.000361873	FBgn0035238
GB40445	-1.965897096	9.711109624	0.000169446	FBgn0031918
GB53933	-1.970478572	6.743403417	0.000162056	FBgn0038799
GB43137	-1.97754052	5.134432911	0.004440768	FBgn0261873
GB41625	-1.988001882	8.819338711	0.000116036	FBgn0035844
GB45484	-1.991418834	6.367994387	0.000131576	FBgn0037447
GB46268	-1.999138538	8.557096153	0.000105739	FBgn0024183
GB41372	-2.000810456	5.993787025	0.000187465	FBgn0038641
GB42653	-2.007026652	7.387963774	0.000295221	FBgn0011288
GB42676	-2.007204397	5.097213141	0.003572966	FBgn0033769
GB54538	-2.009532565	6.26795122	0.000721536	FBgn0262870
GB47349	-2.010024283	6.08776625	0.00015309	FBgn0036298
GB47938	-2.013120508	9.021225895	0.00012913	FBgn0031879
GB52925	-2.014222579	6.297984463	0.000130805	FBgn0086911
GB52955	-2.018076069	6.687215603	0.000387246	FBgn0051559
GB50891	-2.024718845	6.455594963	0.000260771	FBgn0034716
GB40993	-2.026572893	5.718662003	0.00114104	FBgn0038247
GB49283	-2.027262883	6.637279028	0.000259612	FBgn0036398
GB41849	-2.031234485	5.211885794	0.001805586	FBgn0054056
GB42904	-2.033904694	6.51126581	0.000119978	FBgn0262681
GB55275	-2.035427605	7.793256956	4.20189E-05	FBgn0036289
GB54144	-2.048762239	6.19424408	0.000869591	FBgn0259233
GB48813	-2.050088938	6.97158088	0.002218299	FBgn0066365
GB50690	-2.051247458	7.306862421	7.06068E-05	FBgn0261397
GB44426	-2.053105772	6.417219465	3.23129E-05	FBgn0037537
GB50753	-2.057665259	6.23274643	4.29031E-05	FBgn0028474
GB55298	-2.058701502	6.165687666	0.000285219	FBgn0011227

GB51511	-2.059221179	6.090109636	8.57862E-05	FBgn0261261
GB40203	-2.05955359	7.493428116	6.89021E-05	FBgn0051028
GB55241	-2.059621061	6.329637452	8.07657E-05	FBgn0030685
GB50672	-2.0616846	7.653278521	0.000202632	FBgn0086736
GB54347	-2.072692406	6.915967127	0.000177706	FBgn0259734
GB49930	-2.076573294	6.816787905	3.37347E-05	FBgn0028491
GB40263	-2.078148556	7.149232489	0.000334937	0
GB41731	-2.083881975	5.111582063	0.003970057	FBgn0262636
GB46274	-2.090137735	7.415766588	0.000214935	FBgn0030648
GB55483	-2.096835322	8.669309444	0.000848219	FBgn0005666
GB43602	-2.103686019	5.313082231	0.002537846	FBgn0052082
GB40106	-2.111861661	5.407034279	0.000927135	FBgn0264672
GB49870	-2.130293806	6.772379053	0.000121079	FBgn0036821
GB55615	-2.131204132	8.088312388	3.62893E-05	FBgn0034860
GB46015	-2.136523012	5.183597023	0.001608526	FBgn0033753
GB40671	-2.144371475	5.440457908	0.000537956	FBgn0011592
GB51625	-2.147025474	4.755344486	0.004016063	FBgn0033548
GB43612	-2.159051903	6.173120458	2.58882E-05	FBgn0263705
GB51063	-2.164673629	8.769315111	6.49407E-05	FBgn0051973
GB48576	-2.165273963	6.817066851	0.00010569	FBgn0031879
GB55781	-2.166944615	8.651358578	0.000102334	FBgn0053196
GB50295	-2.171609603	6.662750206	3.76602E-05	FBgn0031879
GB41863	-2.175228983	5.383129375	0.000707324	FBgn0029830
GB43941	-2.17925856	7.027014413	6.22058E-05	FBgn0031195
GB43552	-2.190733321	7.102650441	2.0861E-05	FBgn0035575
GB48837	-2.192895198	8.55770549	7.31827E-05	FBgn0039008
GB42796	-2.193462586	5.902920732	0.000227321	FBgn0250839
GB53651	-2.197233672	4.936070508	0.001869505	FBgn0011676
GB42976	-2.200521404	5.805042271	0.000201002	FBgn0004449
GB46886	-2.219079637	5.152270501	0.001301506	FBgn0010399
GB49106	-2.223906736	8.812143004	2.0801E-05	FBgn0250789
GB47082	-2.227145827	5.230342135	0.001000405	FBgn0030660
GB54643	-2.239118228	7.778633754	2.09385E-05	FBgn0019960
GB41270	-2.245502944	9.641543533	2.28907E-05	FBgn0026077
GB49534	-2.245707317	9.688519996	1.02901E-05	FBgn0027341
GB41203	-2.250419782	10.69920613	2.53072E-05	FBgn0026077
GB50538	-2.255957478	5.2131202	0.000568415	FBgn0263986
GB48062	-2.264420716	5.461757909	0.000304572	FBgn0033702
GB43163	-2.290536636	6.583962133	1.00865E-05	FBgn0027503
GB49726	-2.296137758	6.234704617	1.61007E-05	FBgn0003137
GB54127	-2.300548546	4.740917656	0.003715993	FBgn0003353
GB41670	-2.312788133	6.596842873	6.26866E-05	FBgn0010497
GB49184	-2.316964861	5.406431488	0.000351411	FBgn0032749
GB51722	-2.31735256	7.246132443	1.01456E-05	FBgn0035936
GB54213	-2.325434805	7.31378183	1.3437E-05	FBgn0052000
GB41888	-2.326407512	6.731664573	1.4597E-05	FBgn0036398
GB41861	-2.330539953	6.362874641	4.13536E-05	FBgn0053196
GB41855	-2.335403914	7.014019093	9.60886E-06	FBgn0262867
GB49543	-2.352298183	4.711403871	0.003267918	FBgn0036381
GB45937	-2.363288676	8.054774659	8.97915E-06	FBgn0013765
GB46701	-2.363609017	6.862130469	7.04352E-06	FBgn0051204
GB42884	-2.365462487	4.645775447	0.003543166	FBgn0051146
GB47496	-2.373978557	4.537123082	0.003003025	FBgn0039667
GB47513	-2.384752357	5.288251197	0.000308607	FBgn0000448
GB54390	-2.387376487	6.8096501	3.9913E-06	FBgn0025393
GB50085	-2.398062943	4.453019407	0.00322602	FBgn0052736

GB43198	-2.405308261	8.181419331	3.41654E-05	FBgn0000667
GB42804	-2.412290036	8.167590382	3.8548E-06	FBgn0033192
GB40810	-2.420627546	5.107711999	0.000305631	FBgn0035805
GB42612	-2.424012301	8.923911498	9.18433E-06	FBgn0033603
GB54483	-2.424414037	7.302996295	2.35056E-06	FBgn0035539
GB41862	-2.4290223	5.299566138	0.000623884	FBgn0259680
GB42239	-2.446251677	7.346769532	3.67695E-06	FBgn0034943
GB40393	-2.449647971	6.11608568	0.000212859	FBgn0032211
GB46151	-2.457515881	5.083897286	0.001822932	FBgn0082585
GB54076	-2.460084437	6.597074882	2.43332E-05	FBgn0032785
GB52446	-2.460084437	6.620452862	7.29278E-06	FBgn0020300
GB43231	-2.462690862	7.255186414	1.20546E-05	FBgn0035888
GB52447	-2.466717231	5.166289523	0.000338088	FBgn0036780
GB50564	-2.467223082	6.744375255	0.000355185	FBgn0027527
GB53064	-2.470496039	6.630207368	0.000111271	FBgn0010482
GB46077	-2.489962243	4.942008264	0.000455477	FBgn0030729
GB42823	-2.497380439	5.809093379	1.58793E-05	FBgn0036180
GB45235	-2.516514871	5.023120502	0.001380381	FBgn0052432
GB54313	-2.519823251	6.553733832	1.63509E-06	FBgn0030884
GB50262	-2.522301391	5.060027317	0.000464922	FBgn0034911
GB55912	-2.543833229	7.913060699	1.02221E-06	FBgn0026059
GB43205	-2.5465465	5.783398189	1.56813E-05	FBgn0032598
GB41260	-2.550089008	5.34622232	8.99115E-05	FBgn0259247
GB47869	-2.551768105	4.274362768	0.004532756	FBgn0037448
GB55559	-2.552186631	5.268514695	0.000111542	FBgn0033679
GB49809	-2.558420029	5.092747063	0.000366297	FBgn0040351
GB42487	-2.558426858	7.36126224	6.31497E-07	FBgn0260450
GB44842	-2.567303986	6.927016363	6.07468E-07	FBgn0082582
GB41684	-2.574894251	4.864327268	0.000446379	FBgn0031730
GB46368	-2.583275456	5.4222694	4.97999E-05	FBgn0011693
GB55359	-2.591859155	7.780884024	7.07447E-07	FBgn0036365
GB42296	-2.611125754	5.383170777	9.82534E-05	FBgn0038511
GB46113	-2.61161121	5.698671553	5.03671E-06	FBgn0053143
GB50013	-2.612339849	4.95637901	0.001135114	FBgn0036891
GB50567	-2.616445288	6.607747077	8.91525E-07	FBgn0037416
GB47971	-2.621352765	5.90012264	1.27401E-05	FBgn0033917
GB54136	-2.626262722	5.132019837	0.000292539	FBgn0264272
GB46302	-2.634273833	4.539004743	0.00206635	FBgn0262738
GB53970	-2.642916359	8.45925576	2.33861E-07	FBgn0035574
GB44548	-2.647240913	5.806317071	7.20797E-06	FBgn0001112
GB49785	-2.647976106	4.7651953	0.000541705	FBgn0026438
GB44209	-2.653256587	8.31540558	1.05193E-06	FBgn0037537
GB50866	-2.657066739	7.912451855	2.06091E-07	FBgn0010435
GB50453	-2.660295722	6.217967165	1.69142E-07	FBgn0039805
GB44316	-2.663140023	5.075697706	0.000194883	FBgn0033095
GB51442	-2.668126495	7.03628178	1.90558E-06	FBgn0052036
GB46817	-2.6689562	9.695489554	5.66705E-06	FBgn0053257
GB54743	-2.673164508	6.108392333	1.55062E-07	FBgn0004959
GB47004	-2.679790479	4.975920044	0.000236092	FBgn0031849
GB44560	-2.680905338	5.733198258	4.95188E-06	FBgn0003149
GB46597	-2.686827929	5.774577988	1.50898E-06	FBgn0013953
GB52077	-2.692883661	4.384774228	0.002143037	FBgn0003068
GB55765	-2.70045578	6.617793644	1.01844E-06	FBgn0000442
GB42493	-2.714055654	7.208632821	8.19178E-07	FBgn0028573
GB48234	-2.723631509	4.619637079	0.001096737	FBgn0000464
GB55895	-2.724907207	5.486832184	7.61099E-05	FBgn0040705

GB44223	-2.747445514	7.500518957	1.02221E-06	FBgn0027611
GB51736	-2.749812668	8.361062339	1.34388E-06	FBgn0031957
GB40412	-2.756695721	4.216603737	0.002264873	FBgn0051475
GB40306	-2.762589594	5.884066482	1.29431E-06	FBgn0010435
GB42660	-2.770963666	4.188640345	0.004048412	FBgn0028878
GB43718	-2.78064191	5.832976285	8.33388E-07	FBgn0002938
GB45919	-2.781508025	5.078399862	7.77256E-05	FBgn0259164
GB41196	-2.786398322	7.152018549	4.2924E-07	FBgn0023518
GB53343	-2.790858333	6.735405955	4.02045E-07	FBgn0043903
GB45211	-2.80221946	8.563855767	2.31013E-07	FBgn0010423
GB50648	-2.817042919	5.736952871	6.16718E-06	FBgn0003319
GB40503	-2.819767324	8.584117413	2.28428E-08	FBgn0032350
GB44453	-2.8244131	5.590954429	4.28926E-06	FBgn0032804
GB47512	-2.829798021	5.058632237	0.000115358	FBgn0000448
GB47224	-2.831435488	7.737388559	1.53781E-07	FBgn0000568
GB50590	-2.841853611	5.663255831	0.000116669	FBgn0260228
GB52036	-2.843034525	5.076233248	0.000124833	FBgn0003975
GB44002	-2.851637671	5.981301539	7.87276E-07	FBgn0000299
GB55423	-2.85372927	4.26059643	0.002217319	FBgn0036662
GB52028	-2.853838765	9.045348609	1.25823E-07	FBgn0000556
GB53331	-2.871251604	6.788171027	5.11092E-08	FBgn0039294
GB52002	-2.875661712	5.37194517	3.37347E-05	FBgn0031414
GB47718	-2.877510434	4.517645139	0.001079847	FBgn0023091
GB55664	-2.884551792	5.839043287	4.51767E-07	FBgn0051374
GB46976	-2.899654135	5.860967184	9.99709E-05	FBgn0000451
GB52078	-2.903125514	5.328210216	6.03527E-06	FBgn0003068
GB53894	-2.904771268	4.082843495	0.002139034	FBgn0028474
GB54921	-2.906221115	6.377898963	1.0379E-07	FBgn0037487
GB53319	-2.909117585	7.20432798	6.54893E-08	FBgn0034301
GB54818	-2.918737371	7.339964367	1.60045E-07	FBgn0014863
GB42895	-2.924308061	4.81076442	0.000203022	FBgn0030716
GB41262	-2.928922414	5.692397792	1.64638E-06	FBgn0259247
GB45553	-2.930500089	5.401915835	2.35056E-06	FBgn0034157
GB44647	-2.942701529	6.408564037	4.58406E-08	FBgn0261999
GB44798	-2.944035185	7.680128577	2.59929E-08	FBgn0034797
GB55370	-2.944797634	5.456689319	3.9913E-06	FBgn0025878
GB48858	-2.952942581	7.716955783	4.23023E-08	FBgn0040601
GB41331	-2.96019617	4.149457019	0.001223801	0
GB47499	-2.960890588	8.024165311	1.65138E-08	FBgn0085384
GB40009	-2.962955519	4.595255805	0.000621295	FBgn0261836
GB45364	-2.966203206	6.77159402	5.6714E-08	FBgn0261509
GB49946	-2.97448518	5.330961417	1.53728E-05	FBgn0011653
GB47838	-2.977288169	8.160989346	6.36943E-09	FBgn0034974
GB40842	-2.983088631	7.159438146	1.01591E-08	FBgn0001112
GB47977	-2.984499906	10.28716856	6.13319E-08	FBgn0086906
GB45149	-2.987623424	6.741960507	1.67725E-07	FBgn0032785
GB53675	-2.997044089	5.217130139	1.27857E-05	FBgn0031999
GB45609	-3.002757486	7.446352992	4.2946E-07	FBgn0033079
GB45771	-3.004091233	6.126703046	2.85379E-08	0
GB50441	-3.007522197	8.206357011	1.7273E-07	FBgn0038405
GB48709	-3.029336143	6.235823852	1.5959E-08	FBgn0033061
GB55225	-3.050274859	7.395516008	3.26131E-09	FBgn0001083
GB44110	-3.062997819	5.460062717	2.59443E-06	FBgn0086673
GB53163	-3.068647172	4.261587599	0.000560927	FBgn0035113
GB40609	-3.070365499	8.022148038	5.2452E-07	FBgn0039210
GB55216	-3.080284468	5.29907906	1.3744E-05	FBgn0039896

GB41308	-3.0880186	8.974853366	9.2582E-08	FBgn0000046
GB49078	-3.092711855	5.179772468	5.22473E-06	FBgn0052645
GB50000	-3.106922138	8.966622274	2.43307E-09	FBgn0032453
GB53320	-3.116437382	3.890116791	0.003433432	FBgn0013813
GB55547	-3.117817138	7.764171122	8.05735E-08	FBgn0050172
GB41730	-3.134847515	3.820061623	0.004454065	FBgn0262636
GB49273	-3.134847515	3.817117989	0.004920776	FBgn0038837
GB42062	-3.135899719	8.541655853	6.61973E-09	FBgn0039527
GB40446	-3.138901547	6.788096187	3.69991E-09	FBgn0033869
GB47948	-3.139475851	7.059347336	1.67725E-07	FBgn0013988
GB54762	-3.141712771	5.177907872	2.09526E-05	FBgn0033362
GB53345	-3.16015359	8.389079519	9.28004E-10	FBgn0037217
GB43983	-3.169137589	6.549215684	1.83522E-09	FBgn0030617
GB55546	-3.176688598	4.569007955	0.000214264	FBgn0039501
GB44723	-3.18470417	4.528611137	0.000347709	FBgn0259240
GB51355	-3.18576997	5.253485578	2.95944E-06	FBgn0052627
GB54775	-3.192711714	8.794900518	1.42893E-09	FBgn0030027
GB45188	-3.194837583	7.450663075	6.2746E-10	FBgn0031630
GB55986	-3.199184887	4.86781615	5.90495E-05	FBgn0030884
GB48007	-3.200521696	8.11793732	6.09218E-09	FBgn0034412
GB54417	-3.202720565	4.320835236	0.000327187	FBgn0026268
GB47944	-3.208838584	5.768210685	7.50557E-07	FBgn0035410
GB55613	-3.21085354	5.542243463	2.46125E-06	FBgn0030161
GB43500	-3.219223853	3.894184531	0.003221013	FBgn0033196
GB47990	-3.226114042	7.439030435	4.32584E-09	FBgn0004117
GB41616	-3.241424973	5.214408649	2.02087E-05	FBgn0031449
GB42485	-3.250305102	5.449718999	6.05955E-07	FBgn0260450
GB44832	-3.277585893	6.201458722	4.32584E-09	FBgn0038629
GB52025	-3.283565358	8.250327905	6.05662E-10	FBgn0027570
GB50660	-3.285544255	6.380022906	8.79727E-10	FBgn0036995
GB46975	-3.295837129	6.229208791	5.49111E-06	FBgn0002183
GB43276	-3.306365055	5.864226846	8.66915E-08	FBgn0058470
GB49080	-3.307135242	8.429862449	4.55729E-10	FBgn0052645
GB49223	-3.318268293	8.062531375	4.4654E-09	FBgn0259108
GB51828	-3.340509935	5.796489228	1.07942E-07	FBgn0036377
GB44452	-3.341146411	6.972593677	3.88804E-09	FBgn0032803
GB40996	-3.343645229	5.167350349	6.64944E-06	FBgn0260793
GB42892	-3.360053287	5.445552814	1.54799E-07	FBgn0004650
GB55231	-3.362727816	7.026895846	3.60433E-10	FBgn0033602
GB40681	-3.369921497	4.446375402	0.000690424	FBgn0051522
GB40165	-3.377895894	10.61395049	6.35898E-09	FBgn0003065
GB43892	-3.386589019	8.10674224	1.31873E-10	FBgn0032945
GB42427	-3.392649373	8.369421336	7.78429E-10	FBgn0039640
GB45019	-3.405614869	5.793873008	2.67718E-06	FBgn0038894
GB55650	-3.407578119	7.485713807	8.14509E-10	FBgn0011286
GB50236	-3.415339851	6.627190433	1.88052E-06	FBgn0035788
GB41945	-3.423312384	7.449576904	6.5458E-10	FBgn0031097
GB54750	-3.434675162	5.327867112	3.62487E-07	FBgn0259226
GB48258	-3.453606507	4.158483289	0.000440289	FBgn0038151
GB44777	-3.456165419	8.83366404	1.47616E-10	FBgn0032299
GB43465	-3.478681384	6.626072045	1.2013E-10	FBgn0039208
GB49734	-3.480455745	5.368430624	3.37244E-06	FBgn0260386
GB51560	-3.488765772	9.657009042	5.03414E-11	FBgn0034022
GB41723	-3.489182566	8.624337686	9.66795E-12	FBgn0261269
GB47976	-3.490304265	6.257325544	6.21425E-10	FBgn0035308
GB43643	-3.492740345	5.62518911	3.52464E-07	FBgn0016694

GB50041	-3.493863656	3.590999711	0.003072201	FBgn0039749
GB40234	-3.501853128	5.119435235	2.09013E-06	FBgn0037565
GB51089	-3.506504851	4.517836749	0.000131576	FBgn0035917
GB51482	-3.518340572	7.454209367	1.5615E-10	FBgn0085446
GB52186	-3.524828158	5.500188313	1.4455E-07	FBgn0052808
GB42303	-3.526221411	6.32605534	8.14509E-10	FBgn0052816
GB55445	-3.527625785	4.538234747	0.000115735	FBgn0021767
GB47788	-3.535765675	3.599073631	0.00322602	FBgn0261588
GB40008	-3.537732861	7.086212761	1.10106E-09	FBgn0261836
GB55729	-3.543704723	6.913712365	6.15344E-11	FBgn0041712
GB44200	-3.556268969	3.692029785	0.00235249	FBgn0030491
GB55403	-3.573410129	6.97273478	8.57038E-11	FBgn0259994
GB49275	-3.578498141	6.023599863	1.20503E-08	FBgn0038837
GB41306	-3.581441076	8.727126538	4.2599E-10	FBgn0000046
GB50171	-3.590600916	7.254147418	2.16852E-10	FBgn0262717
GB41516	-3.626381798	4.653227169	3.35207E-05	FBgn0004865
GB54393	-3.627784168	6.97393073	3.92193E-11	FBgn0031646
GB41622	-3.63473211	5.190374852	0.000520042	FBgn0035429
GB48003	-3.647693529	5.087773263	0.000110869	FBgn0039214
GB47906	-3.651221754	9.713608684	4.28859E-12	FBgn0033731
GB43691	-3.656667632	6.706868344	2.22432E-10	FBgn0038017
GB51060	-3.689833811	4.311983666	0.000209589	FBgn0035625
GB46705	-3.69581431	8.695253638	3.99869E-10	FBgn0053519
GB51901	-3.70634341	7.050208961	3.21652E-11	FBgn0052645
GB54302	-3.732136012	5.715515736	6.89844E-08	FBgn0038986
GB47037	-3.750566683	7.850303059	4.61988E-11	FBgn0264562
GB41310	-3.775540295	9.672040549	2.76496E-12	FBgn0000046
GB55202	-3.777292658	4.815810538	4.87803E-06	FBgn0041711
GB45938	-3.784281508	5.201447778	1.21662E-07	FBgn0000075
GB42851	-3.820682823	4.366150143	0.001984881	FBgn0085472
GB48125	-3.822104532	7.066660061	1.2776E-10	FBgn0031869
GB42769	-3.827069378	10.67177941	2.37064E-13	FBgn0038405
GB51818	-3.839844453	4.848048444	5.33151E-06	FBgn0030591
GB53579	-3.840274261	5.718178609	8.69704E-09	FBgn0051148
GB46298	-3.848667181	7.265609047	1.16967E-09	FBgn0033728
GB44222	-3.869843605	7.324721881	9.39652E-11	FBgn0039897
GB45853	-3.875290164	4.928561207	7.07447E-07	FBgn0039075
GB42206	-3.887717452	6.956091862	1.35838E-10	FBgn0260856
GB49639	-3.892123229	7.871524556	2.40218E-10	FBgn0039648
GB50570	-3.893403079	6.643672694	5.08601E-10	FBgn0037419
GB55617	-3.918416017	5.949773116	2.10275E-11	FBgn0029836
GB42594	-3.919834962	6.376063289	1.89238E-12	FBgn0036834
GB42599	-3.922214585	4.528058852	9.23512E-05	FBgn0053196
GB52723	-3.922941887	6.654316884	2.26318E-11	FBgn0262647
GB54884	-3.92833463	9.152061925	1.35736E-13	FBgn0053978
GB43052	-3.949077218	9.03295533	6.24787E-11	FBgn0003149
GB46365	-3.972838844	5.005304792	3.90487E-06	FBgn0011693
GB46819	-4.001179807	6.691419298	5.18805E-13	FBgn0051296
GB55604	-4.03433268	4.654431739	4.14615E-06	FBgn0010316
GB50973	-4.038418871	7.314679859	4.86517E-11	FBgn0036044
GB55158	-4.057499745	6.803276276	7.87016E-12	FBgn0027495
GB43029	-4.06002748	8.603408254	1.16553E-11	FBgn0000047
GB53286	-4.065616165	8.461751854	1.55214E-13	FBgn0262111
GB47669	-4.081909544	12.15387274	2.33988E-14	FBgn0015766
GB53420	-4.114760152	4.203122261	4.2964E-05	FBgn0050044
GB41621	-4.135347834	4.21930215	4.75798E-05	FBgn0002440

GB41178	-4.148911528	4.222708227	4.99121E-05	FBgn0038727
GB52992	-4.15280059	8.134628616	1.64842E-09	FBgn0052354
GB40085	-4.155645852	4.165958118	0.000119978	FBgn0029807
GB55598	-4.164818888	8.764367283	2.98593E-12	FBgn0004028
GB42286	-4.175695654	6.31572118	5.24798E-13	FBgn0037122
GB42967	-4.179331296	3.36976743	0.001982059	FBgn0036096
GB55325	-4.180571303	6.881200375	1.59015E-11	FBgn0003721
GB52666	-4.188765101	5.211916641	3.75493E-08	FBgn0053196
GB50170	-4.19065469	8.356791909	1.91306E-14	FBgn0262717
GB46698	-4.197356626	5.445495307	6.13319E-08	FBgn0039862
GB50610	-4.201613022	10.89004997	3.18261E-16	FBgn0050101
GB48167	-4.203321249	7.965755238	3.47053E-12	FBgn0262508
GB41311	-4.210228114	8.7274862	5.48318E-13	FBgn0000045
GB49405	-4.221314667	4.248952609	0.000341746	0
GB43053	-4.22655522	8.855998964	7.97615E-12	FBgn0003149
GB52824	-4.246974849	6.040284286	1.12187E-10	FBgn0033869
GB46038	-4.266203903	5.224531879	2.765E-07	FBgn0052072
GB48443	-4.268459841	7.651438855	1.19437E-14	FBgn0029922
GB40944	-4.273288781	8.158438294	4.21044E-15	FBgn0034903
GB53197	-4.273469451	3.52245991	0.000536906	FBgn0039633
GB50529	-4.277964737	4.263408988	0.000247893	FBgn0037421
GB45732	-4.293104834	8.473616347	1.89623E-15	FBgn0036486
GB42889	-4.316439528	5.591468436	1.10586E-09	FBgn0039478
GB41495	-4.335764953	5.641055614	2.53932E-10	FBgn0016693
GB41946	-4.366099845	8.804405331	7.54701E-16	FBgn0022770
GB42891	-4.376297588	7.792940077	1.03426E-13	FBgn0024366
GB49646	-4.389204035	7.38213646	1.07113E-14	FBgn0038511
GB50006	-4.390132778	3.481918795	0.001729146	FBgn0039927
GB42673	-4.400442211	7.877163022	3.89399E-15	FBgn0032405
GB43580	-4.401088499	10.39392782	9.84937E-17	FBgn0039126
GB40623	-4.402090443	4.363092048	4.82985E-05	FBgn0013733
GB42469	-4.436397484	5.444309647	2.49737E-09	FBgn0036939
GB40007	-4.444266991	6.501239387	2.33716E-12	FBgn0261836
GB41241	-4.468035365	5.978787972	3.66592E-11	FBgn0033633
GB50450	-4.473019023	10.19739245	7.26396E-12	FBgn0001250
GB46310	-4.475009393	7.660304011	1.19943E-14	FBgn0000551
GB41792	-4.478651525	9.508022055	1.33093E-15	FBgn0040601
GB50062	-4.488192589	5.819426949	8.93974E-12	FBgn0037665
GB42852	-4.495295242	4.440148045	3.39046E-05	FBgn0085472
GB45654	-4.513891955	6.314768053	8.26996E-14	FBgn0025837
GB50766	-4.527933327	7.25744027	2.42926E-15	FBgn0264357
GB47223	-4.531989613	4.534483996	3.03161E-06	FBgn0000568
GB42358	-4.576485287	7.324338886	5.87011E-16	FBgn0087011
GB55396	-4.58285974	7.880538415	1.29315E-15	FBgn0052694
GB48216	-4.585709871	6.384125284	4.11426E-15	FBgn0031692
GB54133	-4.588746585	3.616695946	0.000773598	FBgn0038958
GB41771	-4.590756884	6.382530023	3.50568E-14	FBgn0024897
GB49400	-4.611551566	6.550512455	1.8355E-14	FBgn0262599
GB51698	-4.619247392	13.43425843	5.17146E-18	FBgn0002564
GB53935	-4.636767069	3.9280953	0.000118266	FBgn0011476
GB53516	-4.648635121	8.581974222	1.44925E-16	FBgn0043792
GB55611	-4.66714878	5.280435365	3.54085E-08	FBgn0010399
GB50439	-4.686885525	9.612173279	1.12795E-14	FBgn0039480
GB42704	-4.695531891	7.953213311	4.11249E-15	FBgn0037290
GB42890	-4.705260875	5.812747146	8.90179E-06	FBgn0039027
GB51214	-4.70749075	9.59767265	1.49813E-14	FBgn0004169

GB40607	-4.716877107	5.283312277	5.7012E-07	FBgn0037556
GB48967	-4.723190852	4.67455409	2.80468E-06	FBgn0260006
GB51407	-4.725236523	6.812992005	7.27311E-13	FBgn0050069
GB52179	-4.758963991	3.910908645	0.000173123	FBgn0026314
GB50650	-4.768549026	6.632816403	2.26913E-09	FBgn0033359
GB50574	-4.785486053	5.116036559	0.000242544	FBgn0051561
GB42580	-4.7864039	6.290932829	1.05804E-13	FBgn0261446
GB49270	-4.79749969	3.838695588	0.000160575	FBgn0032402
GB49401	-4.798272153	4.806499112	3.11941E-07	FBgn0030257
GB49979	-4.80592517	3.899016175	4.60786E-05	FBgn0043550
GB53288	-4.806853762	4.876959681	2.95944E-06	FBgn0040211
GB50572	-4.808740522	7.318274697	7.75907E-14	FBgn0040279
GB42596	-4.811829871	7.909046319	4.55259E-12	FBgn0021742
GB40228	-4.815193774	6.144671291	4.05199E-15	0
GB53986	-4.822868842	5.421556736	8.05899E-10	FBgn0026268
GB48769	-4.848193235	5.399533355	1.31568E-08	FBgn0032362
GB54456	-4.849877274	5.862134241	1.56914E-13	FBgn0032462
GB51787	-4.851676144	9.519302701	1.64684E-17	FBgn0002772
GB49802	-4.851971991	5.808111412	4.10874E-11	FBgn0086906
GB46386	-4.859307391	5.458893665	9.68789E-11	FBgn0050420
GB50636	-4.889525953	9.327619975	1.74339E-18	FBgn0031097
GB41904	-4.897799451	7.01249198	9.84937E-17	FBgn0030884
GB54963	-4.902039077	4.981497759	4.98158E-07	FBgn0039335
GB53119	-4.904322103	10.26874224	1.20711E-10	0
GB48474	-4.930525076	10.08498913	1.60426E-19	FBgn0035398
GB42533	-4.957659583	6.251845714	9.87899E-15	FBgn0040233
GB54970	-4.971144321	9.313416683	2.22138E-13	FBgn0035089
GB48979	-4.97429035	5.043080181	2.77912E-09	FBgn0037323
GB42909	-4.987749594	6.149874522	5.68362E-09	FBgn0039027
GB52184	-4.998561045	6.427960413	1.70971E-12	0
GB49795	-5.003096906	7.686428676	2.84486E-12	FBgn0029681
GB52105	-5.00927124	6.488416303	1.74734E-10	FBgn0051807
GB40771	-5.014283951	6.457770548	1.21039E-13	FBgn0033763
GB40240	-5.018834859	10.74927451	1.07363E-17	FBgn0002773
GB54767	-5.045342038	4.115338064	1.55188E-05	FBgn0030947
GB40837	-5.050501554	10.06177316	5.6625E-19	FBgn0037323
GB40299	-5.07896436	5.044131205	5.88697E-07	FBgn0035513
GB43298	-5.081758766	9.451654337	1.62563E-16	FBgn0030539
GB42908	-5.093982189	6.426870599	8.73157E-07	FBgn0039027
GB41015	-5.101821441	11.86330233	1.2788E-18	FBgn0031957
GB53517	-5.114550818	6.683980526	1.08222E-15	FBgn0035935
GB50828	-5.125223861	7.802580055	7.36255E-18	FBgn0261836
GB50612	-5.127728644	5.050657362	5.25337E-05	FBgn0050101
GB40604	-5.128487422	6.484501513	1.46788E-07	FBgn0261526
GB53524	-5.129949967	7.830893429	6.81639E-18	FBgn0043792
GB48066	-5.192521664	6.127479921	3.30995E-14	FBgn0020389
GB51653	-5.206724426	11.47942674	2.28381E-16	FBgn0264695
GB49772	-5.232534432	6.927856205	2.40978E-12	FBgn0029681
GB41383	-5.234954385	5.216126889	2.47196E-07	FBgn0035788
GB47947	-5.243076319	8.052803305	4.25507E-16	FBgn0013988
GB53112	-5.264269776	6.294392883	1.49086E-14	FBgn0034289
GB48161	-5.27006227	9.772815793	1.13472E-20	FBgn0034391
GB45850	-5.281921471	9.730544533	3.47895E-16	FBgn0035636
GB46385	-5.302159421	4.415837825	3.18864E-07	FBgn0003292
GB49268	-5.303263267	5.279873592	4.19724E-10	FBgn0038837
GB48860	-5.35568434	6.287806126	1.99221E-13	FBgn0038819

GB51652	-5.361528363	8.812402068	3.33143E-15	FBgn0264695
GB50827	-5.436894418	8.461822873	3.3657E-19	FBgn0051205
GB42911	-5.488578251	5.845076453	1.9828E-06	FBgn0053299
GB43181	-5.53104701	8.112792763	7.96471E-11	FBgn0037992
GB42702	-5.538412918	10.08391916	1.77277E-23	FBgn0037288
GB42763	-5.54001719	8.094787076	2.09105E-19	FBgn0023388
GB54489	-5.560863922	5.616823128	2.54535E-13	FBgn0052829
GB44561	-5.562633512	6.805217234	7.80898E-17	FBgn0039075
GB45764	-5.570557783	8.259169206	5.68382E-21	FBgn0004117
GB55100	-5.586526799	6.810371491	9.35428E-15	FBgn0003016
GB48107	-5.590611244	7.620552938	3.42467E-18	FBgn0017561
GB42912	-5.601825696	7.880161336	9.68068E-14	FBgn0053196
GB51441	-5.605015751	11.88383665	2.58092E-24	FBgn0019960
GB50563	-5.63803938	4.434380649	0.000563174	FBgn0037413
GB47752	-5.650168899	7.346994788	5.59993E-17	FBgn0001992
GB50562	-5.666917118	8.38010292	1.32085E-07	FBgn0037411
GB49689	-5.691423371	8.400241884	7.02747E-22	FBgn0004577
GB52634	-5.693582482	4.708216619	2.92871E-08	FBgn0040087
GB48861	-5.693810809	7.557611151	1.16919E-17	FBgn0038819
GB46973	-5.706007278	8.480500495	1.58576E-21	FBgn0263038
GB47118	-5.708045131	9.16392099	1.2905E-22	FBgn0038439
GB45860	-5.752134736	7.205251047	9.63399E-14	FBgn0066365
GB53120	-5.756254626	7.227763674	1.90962E-17	FBgn0013733
GB40447	-5.783002443	8.733287709	1.56291E-15	FBgn0033869
GB47126	-5.790169643	4.826038022	5.22145E-09	FBgn0004577
GB47550	-5.80849882	5.715260965	4.29337E-12	FBgn0052537
GB53110	-5.817095683	14.17832188	4.88083E-23	FBgn0034289
GB49021	-5.821032265	10.78099321	3.07282E-23	FBgn0034030
GB48174	-5.838074585	9.823040797	1.31328E-20	FBgn0033158
GB55614	-5.839095648	7.745301277	4.46895E-20	FBgn0011225
GB50613	-5.858958574	11.6433098	6.43324E-20	FBgn0050101
GB54791	-5.872695383	4.947867426	6.24293E-10	FBgn0003089
GB42582	-5.909735726	7.433201852	4.8934E-19	FBgn0035280
GB52052	-5.913540253	8.574834696	2.51517E-18	FBgn0029690
GB48173	-5.941587183	8.444805639	3.89013E-20	FBgn0033158
GB46518	-5.961228168	9.184098924	7.31037E-12	0
GB50875	-6.029038816	8.011586482	1.97119E-18	FBgn0053126
GB42597	-6.057635881	13.17490254	7.8597E-27	FBgn0032538
GB40624	-6.08301011	9.109670027	1.06877E-24	FBgn0259247
GB46585	-6.083297796	12.88830198	1.83854E-25	FBgn0032538
GB46591	-6.116275523	12.55518039	5.39413E-26	FBgn0032538
GB50442	-6.12132607	6.092354723	4.15002E-17	FBgn0039480
GB50449	-6.148744464	7.975815472	1.03639E-19	0
GB43769	-6.175264063	7.499174969	2.01547E-07	FBgn0037411
GB40659	-6.199643489	5.227072823	2.76826E-11	FBgn0043043
GB50451	-6.22039319	11.82883866	1.40678E-23	FBgn0053196
GB42888	-6.267283871	10.53464528	5.20919E-26	FBgn0053196
GB49394	-6.283050227	8.820655725	3.80631E-24	FBgn0259247
GB44399	-6.286359943	11.83025295	3.71015E-23	FBgn0050101
GB55612	-6.334598756	8.689104652	7.11861E-25	FBgn0263216
GB45957	-6.359998109	8.661041826	1.96961E-22	FBgn0034157
GB41624	-6.38691439	7.310161333	2.15409E-20	FBgn0035845
GB55599	-6.505274643	6.938965723	1.35114E-16	FBgn0052656
GB46414	-6.557423614	5.354843175	7.23412E-07	0
GB45763	-6.55763701	7.733775953	4.67523E-19	FBgn0004117
GB53525	-6.585266639	7.045538171	3.84459E-19	FBgn0035641

GB53113	-6.611990587	9.015994339	1.54748E-21	FBgn0040496
GB47946	-6.715593543	5.667965701	8.37179E-14	FBgn0013988
GB52104	-6.745467257	5.707155227	1.25413E-13	FBgn0039479
GB47903	-6.756355417	8.211310569	3.74474E-24	FBgn0036110
GB42910	-6.833670856	6.570412016	1.32109E-08	FBgn0053299
GB48794	-6.87499506	11.81852613	1.15029E-30	FBgn0004034
GB46394	-6.94435205	7.858837353	1.06877E-24	FBgn0035091
GB43877	-6.956379293	6.805400244	6.88107E-17	FBgn0034883
GB46399	-7.153183032	8.993390014	3.92573E-24	FBgn0032256
GB50876	-7.259560389	6.142196603	2.33716E-12	FBgn0053126
GB53776	-8.099737368	2.650528954	0.003528047	FBgn0011592
GB43805	-8.122375269	2.591915423	0.003109795	FBgn0029843
GB47536	-8.28170344	2.726603252	0.001567272	FBgn0038181
GB47129	-8.369485096	2.747114111	0.003784405	FBgn0041711
GB42635	-8.434248478	2.74737882	0.004434669	FBgn0085458
GB53313	-8.469991324	2.82229345	0.002165808	0
GB46403	-8.547315013	3.061985482	0.000545027	FBgn0036299
GB52635	-8.555656194	2.957106505	0.000432216	FBgn0034071
GB48489	-8.58039423	2.961044376	0.000863191	FBgn0031835
GB48796	-8.705611	3.154815695	0.000225734	FBgn0012344
GB49962	-8.96556751	3.309630302	0.000253539	FBgn0004511
GB49295	-9.108489444	3.453956848	4.94432E-05	FBgn0014859
GB42641	-9.158634304	3.439247605	0.00013399	FBgn0039200
GB53689	-9.212379985	3.588446098	2.97551E-05	FBgn0032707
GB50611	-9.228120865	3.631057141	0.000448985	FBgn0050101
GB43367	-9.314213679	3.452639216	0.001072525	FBgn0053003
GB44199	-9.367302331	3.727243255	7.84076E-06	FBgn0052635
GB54457	-9.418506553	3.798329447	6.002E-06	FBgn0052829
GB47120	-9.570298806	3.927832741	8.73157E-07	FBgn0038880
GB54904	-9.630762359	3.982327738	1.19573E-06	FBgn0032402
GB53285	-9.715096006	4.003488356	2.18356E-06	FBgn0002709
GB52017	-9.766304748	4.041213929	1.33065E-06	FBgn0037552
GB51819	-9.919579063	4.26130191	4.68755E-08	FBgn0030590
GB42581	-10.44689763	4.782268213	1.18467E-10	FBgn0035873
GB46417	-10.50404493	4.65421034	1.87871E-06	FBgn0037662
GB50887	-10.51905168	4.803666443	1.97437E-10	FBgn0029961
GB50577	-10.59991805	4.807480617	1.01135E-07	FBgn0037427
GB47927	-10.72906377	4.933531409	1.36655E-09	0

Contrast 3
High DWV alone vs. control

Gene Label	logFC	logCPM	FDR	Flybase ortholog
GB40949	1.973596654	5.250360424	0.009903999	FBgn0039633
GB51046	1.925530595	8.159162805	0.0143602	FBgn0030306
GB47822	1.923396465	5.627661861	0.009870622	FBgn0034726
GB52690	1.887696469	5.184948937	0.018431057	FBgn0033100
GB54882	1.876488975	4.943596619	0.031832385	FBgn0030611
GB40653	1.862193524	10.34275892	0.019369986	FBgn0032518
GB48780	1.860627521	5.52461989	0.009870622	FBgn0052022
GB45554	1.854606	5.40760147	0.018527416	FBgn0000173
GB50601	1.843917143	8.533089762	0.019369986	FBgn0038224
GB46222	1.841750325	9.680706742	0.046834472	FBgn0034470
GB48338	1.822932651	5.024331604	0.037028055	FBgn0030480
GB41806	1.814293129	5.553014369	0.029882617	FBgn0038088

GB41161	1.773367682	4.977949489	0.046834472	FBgn0086447
GB46785	1.768406753	5.787025573	0.010106983	FBgn0037378
GB54555	1.765057288	6.651431725	0.009870622	FBgn0039868
GB54462	1.76374221	6.226457157	0.008176697	FBgn0035541
GB40862	1.7621768	6.040998751	0.009870622	FBgn0033085
GB50724	1.760680425	4.988613432	0.037528353	FBgn0026566
GB40892	1.759749647	6.250081989	0.009870622	FBgn0260401
GB40492	1.756850908	9.480902251	0.034644314	FBgn0030616
GB52470	1.754113283	5.892640513	0.009870622	FBgn0050105
GB55895	1.749968806	6.013105282	0.009903999	FBgn0040705
GB54280	1.748802913	5.681438483	0.013099305	FBgn0036277
GB54970	1.745402987	10.01706966	0.037971201	FBgn0035089
GB45629	1.741709922	8.750642528	0.031696781	FBgn0031228
GB45443	1.737105022	5.904061673	0.009870622	FBgn0037469
GB48916	1.734332239	5.985575224	0.014562109	FBgn0034744
GB53957	1.729746447	5.424252936	0.031061547	FBgn0261067
GB42821	1.728499769	6.963597321	0.009903999	FBgn0036581
GB53539	1.727642033	6.084759337	0.009870622	FBgn0039764
GB54652	1.724237644	7.134927148	0.018431057	FBgn0039765
GB46748	1.724056554	6.729122008	0.009903999	FBgn0034579
GB53184	1.721405991	4.85522973	0.037528353	FBgn0032906
GB51410	1.718243312	6.053920697	0.009870622	FBgn0035110
GB47877	1.713698053	5.506076003	0.032293987	FBgn0039335
GB48835	1.710729673	5.023379402	0.0444491	0
GB43875	1.709530219	6.173740484	0.009870622	FBgn0051800
GB51003	1.708039165	6.473015787	0.009870622	FBgn0051913
GB53819	1.70672222	5.714801471	0.0143602	FBgn0028980
GB50371	1.695279785	5.051932204	0.037528353	FBgn0050147
GB52258	1.694677811	5.712990185	0.013099305	FBgn0033527
GB44173	1.688862949	5.762414736	0.010499177	FBgn0243513
GB55782	1.685818826	7.045620651	0.020279435	FBgn0033570
GB51993	1.683915498	5.956412388	0.009903999	FBgn0035592
GB55222	1.68061697	5.601365798	0.016986996	FBgn0031403
GB54312	1.679350632	5.81030781	0.013099305	FBgn0038678
GB50981	1.674386656	5.688039683	0.03097423	FBgn0035169
GB54752	1.67329874	5.168291585	0.037099234	FBgn0030434
GB42615	1.672525185	4.97760221	0.046834472	FBgn0042112
GB44600	1.668282396	5.606301943	0.017373875	FBgn0035398
GB51206	1.660507773	8.263046489	0.035215471	FBgn0015031
GB44903	1.659086573	7.573210731	0.037528353	FBgn0015614
GB49433	1.654933218	6.121355045	0.010106983	FBgn0029148
GB52888	1.653012974	5.187974796	0.043332436	FBgn0030834
GB54681	1.65291092	5.223329647	0.037028055	FBgn0035271
GB40270	1.651089224	5.772535414	0.020279435	FBgn0037979
GB44781	1.649456023	4.951498305	0.03721973	FBgn0262603
GB54227	1.647831003	6.634712633	0.013099305	FBgn0036519
GB40018	1.641860146	5.856465632	0.014926471	FBgn0261789
GB40590	1.638924298	6.209304522	0.009870622	FBgn0031766
GB46619	1.638301767	5.274813634	0.041784913	FBgn0043002
GB53661	1.632760444	6.952912277	0.022512639	FBgn0035247
GB50032	1.631243827	7.466500227	0.035215471	FBgn0036918
GB42698	1.631060525	5.85759882	0.013099305	FBgn0010622
GB54387	1.629215059	6.887059107	0.020279435	FBgn0037899
GB47331	1.627416343	6.022796491	0.009870622	FBgn0036580
GB50230	1.624314852	6.876420023	0.035806998	FBgn0035521
GB44033	1.619009041	10.48412793	0.046834472	FBgn0031980

GB47311	1.616054301	6.011621106	0.014562109	FBgn0031189
GB55479	1.610495187	6.446643144	0.014562109	FBgn0041249
GB51118	1.609934568	6.900108459	0.018476012	FBgn0034361
GB42887	1.608600991	7.016461682	0.031832385	FBgn0031381
GB47192	1.603249311	5.980252764	0.014562109	FBgn0036916
GB46688	1.6028816	5.47939471	0.031832385	FBgn0033199
GB48958	1.602523342	7.286352881	0.031327438	FBgn0040931
GB48201	1.596636449	5.922632498	0.01232611	FBgn0050481
GB50335	1.596000748	7.226786467	0.031832385	FBgn0050354
GB49250	1.595462091	5.936248572	0.016986996	FBgn0037933
GB51884	1.594963432	6.949870182	0.033581868	FBgn0029882
GB54205	1.59035348	5.716488368	0.019369986	FBgn0034402
GB53604	1.582873299	5.21207526	0.046834472	FBgn0015544
GB52644	1.582451586	7.849135975	0.044390068	FBgn0016119
GB50167	1.580845465	6.441892874	0.017373875	FBgn0036774
GB49956	1.580233281	6.33894492	0.014926471	FBgn0039751
GB43388	1.580205814	5.383920179	0.043460422	FBgn0013347
GB54100	1.576106609	6.365660823	0.01698474	FBgn0035469
GB48484	1.574837092	6.375173758	0.018527416	FBgn0038426
GB55961	1.573549044	6.800454915	0.047199172	FBgn0263852
GB43376	1.571480729	6.511939895	0.018527416	FBgn0040076
GB45058	1.569312404	5.907558079	0.014562109	FBgn0036107
GB45210	1.568597814	6.850125404	0.037971201	FBgn0025700
GB53410	1.560429875	5.700848146	0.021464184	FBgn0035436
GB47650	1.557397408	8.513511637	0.048723494	FBgn0023170
GB47473	1.552774971	6.174990561	0.022512639	FBgn0040899
GB51498	1.549472186	5.82846544	0.017373875	FBgn0033402
GB51597	1.548176758	6.194906675	0.014562109	FBgn0004832
GB55473	1.546605769	5.635434149	0.033672785	FBgn0040890
GB49365	1.546263185	5.473048766	0.033581868	FBgn0053198
GB44571	1.54424588	5.188771487	0.047322004	FBgn0031897
GB55109	1.544198823	5.98183979	0.019952476	FBgn0034988
GB55161	1.541822409	5.634316672	0.025257869	FBgn0051251
GB41973	1.539129047	7.014495383	0.047199172	FBgn0037843
GB46194	1.538118236	5.234598453	0.046166715	FBgn0039929
GB44443	1.5357306	5.248940614	0.046834472	FBgn0039135
GB47321	1.533456822	6.861856255	0.025257869	FBgn0039111
GB51106	1.530236957	6.115948685	0.017373875	FBgn0033485
GB50549	1.525245706	5.671416275	0.025550709	FBgn0036668
GB51539	1.524535023	6.692546334	0.020279435	FBgn0034986
GB52587	1.524487726	5.246811153	0.048723494	FBgn0032871
GB53513	1.523605762	4.962105864	0.046834472	FBgn0011291
GB40032	1.522590126	6.498718645	0.016468033	FBgn0031361
GB42998	1.513658021	6.411502477	0.020279435	FBgn0038307
GB42488	1.510755192	5.498326011	0.03097423	FBgn0052576
GB54326	1.510384169	5.278662999	0.046834472	FBgn0029522
GB42631	1.508945718	6.064884231	0.037528353	FBgn0034641
GB54976	1.504381093	7.159922	0.047424863	FBgn0034791
GB45653	1.504247234	5.491066058	0.046834472	FBgn0032732
GB53335	1.503561989	5.295503791	0.046834472	FBgn0035211
GB55157	1.502782982	7.184756951	0.048723494	FBgn0035603
GB55920	1.499624634	5.511967933	0.041784913	FBgn0037777
GB54450	1.499547982	6.420741786	0.015736955	FBgn0038302
GB42717	1.497373351	5.448782145	0.048723494	FBgn0262360
GB42863	1.495508682	5.745251134	0.046834472	FBgn0035692
GB50928	1.49276359	6.014738768	0.035427863	FBgn0040389

GB46272	1.489923991	6.166343987	0.040942403	FBgn0085220
GB41330	1.489540185	6.758457956	0.033581868	FBgn0040954
GB42205	1.489366436	5.301225556	0.046834472	FBgn0039404
GB55156	1.481968684	5.598172853	0.035215471	FBgn0035532
GB46911	1.479734814	6.796520859	0.03827246	FBgn0038331
GB45367	1.47809449	5.514657575	0.044390068	FBgn0028992
GB48682	1.47699117	5.909705092	0.032179221	FBgn0039557
GB55105	1.476316804	5.96085296	0.022512639	FBgn0086362
GB49234	1.475394533	6.163156507	0.020279435	FBgn0026089
GB40399	1.474160008	6.845004872	0.043460422	FBgn0260858
GB50727	1.472609149	7.144952785	0.046834472	FBgn0010741
GB45946	1.472578205	5.405927554	0.044390068	FBgn0034000
GB45264	1.470524795	6.045554139	0.022512639	FBgn0031639
GB42017	1.469326723	5.450382624	0.044390068	FBgn0050159
GB51581	1.468832126	6.904397999	0.035215471	FBgn0034854
GB43187	1.466862084	6.275780507	0.038394324	FBgn0085468
GB50870	1.465030931	6.318805636	0.020279435	FBgn0034001
GB44608	1.463435861	6.85190464	0.033581868	FBgn0034645
GB55298	1.462638683	6.651338823	0.031061547	FBgn0011227
GB48646	1.458647853	5.932629715	0.035806998	FBgn0004908
GB53663	1.456596285	6.231261305	0.019952476	FBgn0038106
GB51900	1.453862051	6.50456445	0.031061547	FBgn0038746
GB45947	1.453109663	6.746670214	0.037528353	FBgn0024841
GB48470	1.451599303	6.430776027	0.031233643	FBgn0036847
GB48293	1.450521376	6.813760613	0.033581868	FBgn0039159
GB55628	1.448613391	5.725503633	0.038394324	FBgn0051957
GB49402	1.447686878	5.763310972	0.031696781	FBgn0032821
GB47802	1.446740499	6.447865586	0.032179221	FBgn0011509
GB41648	1.445925515	6.615429798	0.033581868	FBgn0067317
GB48754	1.440681796	5.631701201	0.03810028	FBgn0039664
GB54371	1.439604801	6.714492003	0.037971201	FBgn0029755
GB48781	1.437114077	5.546756236	0.048901681	FBgn0030711
GB51624	1.437078577	6.293947342	0.021212993	FBgn0035534
GB45307	1.435413539	6.663570132	0.037528353	FBgn0036300
GB47965	1.43450171	6.328786197	0.031061547	FBgn0038989
GB49013	1.434420603	6.600254077	0.048901681	FBgn0033378
GB47093	1.425786615	6.547197264	0.037028055	FBgn0052708
GB49299	1.425314097	5.710960302	0.033581868	FBgn0030088
GB51623	1.423830567	6.401837578	0.037528353	FBgn0033754
GB43639	1.423816833	6.259705012	0.029232799	FBgn0034939
GB54183	1.419244716	6.600501462	0.036802857	FBgn0003275
GB52250	1.415170624	6.417190415	0.046834472	FBgn0260390
GB50033	1.410831859	5.842133163	0.037528353	FBgn0038586
GB45824	1.409267276	6.038273609	0.035806998	FBgn0023129
GB53186	1.394636069	5.832400226	0.037528353	FBgn0026741
GB46503	1.388949727	5.513549149	0.046834472	FBgn0039218
GB41657	1.388442824	5.788782164	0.046834472	0
GB53955	1.38823351	5.921466933	0.038394324	FBgn0031871
GB52806	1.387380154	6.175456718	0.031696781	FBgn0035337
GB49606	1.387273415	6.288090912	0.037028055	FBgn0014391
GB42083	1.383044647	6.445788271	0.037028055	0
GB54655	1.381334371	6.142168525	0.035806998	FBgn0037567
GB51484	1.379677733	6.107263048	0.045383402	FBgn0002736
GB55932	1.378719361	6.48501583	0.041998413	FBgn0053002
GB46793	1.376213337	6.275557306	0.032179221	FBgn0050185
GB41905	1.372741162	5.968107229	0.043332436	FBgn0262467

GB47724	1.36805845	6.047424563	0.045951864	FBgn0260407
GB42667	1.364540378	5.828414014	0.037528353	FBgn0260861
GB53037	1.363534198	6.029731022	0.043332436	FBgn0038662
GB55427	1.361166132	6.173908765	0.034981623	FBgn0035088
GB43537	1.354848358	6.776005476	0.045383402	FBgn0033907
GB47176	1.352703637	6.146445214	0.046834472	FBgn0033480
GB51746	1.351781011	6.088175587	0.034981623	0
GB48874	1.351357489	6.424125035	0.044710336	FBgn0038166
GB55062	1.35077453	6.025409542	0.043460422	FBgn0039835
GB53270	1.350732444	6.416879887	0.03810028	FBgn0028919
GB49522	1.350039258	5.945363807	0.043460422	FBgn0024196
GB46896	1.349582871	6.165729838	0.04557923	FBgn0010531
GB48064	1.345947364	6.594566604	0.044390068	FBgn0026261
GB44434	1.345234765	5.737856665	0.0444491	FBgn0039690
GB47591	1.342417764	5.640343209	0.047322004	FBgn0034242
GB45549	1.342204914	6.199747293	0.03810028	FBgn0010438
GB54677	1.341957696	6.507958767	0.037528353	FBgn0034232
GB48707	1.339664259	6.327295635	0.034898287	FBgn0024222
GB51224	1.33730523	5.908131654	0.044710336	FBgn0027359
GB50130	1.333992602	6.086857766	0.043013384	FBgn0037489
GB52800	1.328336317	6.192352034	0.038394324	FBgn0023521
GB40686	1.328260817	5.714713399	0.047435062	FBgn0032033
GB44188	1.323678386	5.761468565	0.045997142	FBgn0031979
GB44891	1.323415277	6.702912224	0.046834472	FBgn0039212
GB53418	1.32190045	5.892947436	0.04972344	FBgn0031836
GB48424	1.321599187	5.846690234	0.048904665	FBgn0033195
GB46602	1.317442353	6.257973144	0.041784913	FBgn0261788
GB55944	1.31724419	5.910604131	0.048901681	FBgn0038323
GB41490	1.315327947	6.133338729	0.044710336	FBgn0036812
GB40955	1.313794825	6.214378383	0.043332436	FBgn0046114
GB44925	1.308658446	6.002521942	0.047795993	FBgn0028688
GB44417	1.308477022	6.255411926	0.046754923	FBgn0038808
GB48414	1.305272235	6.407324086	0.044390068	FBgn0260859
GB51081	1.300260904	6.169468969	0.037971201	FBgn0036922
GB45739	1.297477016	6.218352811	0.046834472	FBgn0037709
GB45256	1.292699083	6.256663193	0.046834472	FBgn0033813
GB40349	1.283959215	6.405720167	0.046834472	FBgn0025336
GB54855	1.275544483	6.218304699	0.046834472	FBgn0032726
GB53192	1.268402911	6.200135236	0.046834472	FBgn0002526
GB54469	1.267968719	6.347556381	0.04557923	FBgn0035980
GB42039	1.259923291	6.369064173	0.047435062	FBgn0038235
GB54237	-1.552550515	5.667619847	0.047258032	FBgn0025640
GB55388	-1.826040799	7.243835441	0.038394324	FBgn0033763

Contrast 4
High SBV+DWV vs. control

Gene Label	logFC	logCPM	FDR	Flybase ortholog
GB48823	9.975963839	8.417408128	9.30709E-39	FBgn0004778
GB47618	9.827890531	6.362719241	2.35958E-28	FBgn0010385
GB50423	9.687174813	9.60167835	6.10994E-42	FBgn0052055
GB42310	9.533793853	3.365841499	3.88731E-08	FBgn0045827
GB43007	9.451446179	3.374417338	7.98401E-07	FBgn0030592
GB41428	9.328092198	8.624252521	1.27755E-31	FBgn0010385
GB50363	9.222319575	3.080676699	3.86486E-06	FBgn0038642

GB47520	8.655274915	4.253049259	3.50255E-13	FBgn0045827
GB41637	8.520853159	7.052127165	1.0992E-37	FBgn0030905
GB45912	8.469586208	10.59916742	6.10994E-42	FBgn0011296
GB51583	8.459516563	4.023620558	4.93732E-12	FBgn0036117
GB40298	8.366299716	2.211387043	0.001799407	FBgn0035132
GB51126	8.27348174	1.995096509	0.001197429	FBgn0036009
GB54001	8.27348174	2.016415861	0.001521608	FBgn0036481
GB43112	8.143481253	9.218087316	1.22271E-36	FBgn0028573
GB51223	8.05719313	11.01821985	5.59009E-37	FBgn0014002
GB48146	7.993392793	6.353596168	7.27656E-21	FBgn0038071
GB51306	7.974619547	6.286502708	9.95087E-33	0
GB47475	7.96831875	10.39741105	1.12207E-40	FBgn0011296
GB45584	7.952718772	1.611578215	0.005303794	FBgn0036481
GB55435	7.893551009	5.166972688	5.03355E-22	FBgn0020626
GB53798	7.81835809	9.295255704	9.27916E-24	FBgn0032132
GB44824	7.690848552	1.282696226	0.011936386	FBgn0036278
GB48149	7.539621061	1.14186126	0.025394515	FBgn0028579
GB41097	7.381732963	3.683106039	7.13235E-10	FBgn0051954
GB50121	7.245072656	9.048188585	4.01567E-35	FBgn0029167
GB54238	7.11720077	6.496912999	1.24643E-29	FBgn0053547
GB45954	7.065846041	6.334972115	9.02035E-20	FBgn0053196
GB42626	7.042181319	8.262359378	5.89229E-32	FBgn0005613
GB45910	6.95915614	11.81098282	1.75528E-34	FBgn0011296
GB42623	6.928497292	2.385927831	0.00016434	FBgn0026427
GB41361	6.850699395	2.308999492	0.000314394	FBgn0036575
GB55515	6.684262045	8.305748408	3.46768E-20	FBgn0036262
GB48833	6.683182146	7.884694975	3.34349E-19	FBgn0004778
GB45909	6.669953266	10.97736109	5.32259E-33	FBgn0011296
GB48134	6.640245978	9.90610792	2.78677E-19	FBgn0001258
GB50477	6.605059242	8.702711817	2.68747E-26	FBgn0263774
GB49219	6.601796479	4.135748581	2.03612E-11	FBgn0031905
GB49890	6.577252438	7.392343432	3.36435E-18	FBgn0033302
GB45906	6.458869763	10.70981552	9.97873E-31	FBgn0011296
GB51174	6.419326178	9.563229124	1.5538E-29	FBgn0033661
GB49888	6.384027752	4.969066396	2.47567E-22	FBgn0000473
GB46223	6.354099549	3.898467873	2.19951E-09	FBgn0034470
GB49385	6.172323183	2.395391404	0.000131975	FBgn0051201
GB54881	6.164393495	5.747745532	1.18394E-21	FBgn0036829
GB47318	6.142526381	8.933951971	4.62317E-18	FBgn0032835
GB54908	6.094525286	2.463523717	0.001031614	FBgn0263705
GB43924	6.084312345	9.797848933	6.83455E-27	FBgn0263072
GB42797	6.06963865	7.948741933	7.37787E-29	FBgn0037288
GB51196	6.005809075	1.455012827	0.017288065	FBgn0028847
GB42540	5.971309992	7.542585215	9.6575E-30	FBgn0037329
GB51419	5.967494676	6.219174285	7.96955E-23	FBgn0035439
GB41709	5.950683941	8.099583571	4.1927E-25	FBgn0030691
GB40137	5.939271002	5.704320675	1.53118E-22	FBgn0038595
GB45907	5.84626461	9.333251005	6.83455E-27	FBgn0011296
GB42425	5.752558188	2.457173301	4.81244E-05	FBgn0039640
GB41284	5.627548769	6.670613729	2.68747E-26	FBgn0050197
GB42621	5.563237542	9.632901977	7.96955E-23	FBgn0016075
GB52705	5.511505186	1.679432892	0.004911705	FBgn0085512
GB51833	5.494225217	0.646694889	0.044002455	FBgn0029762
GB41636	5.493197387	5.785091883	4.29544E-29	FBgn0013733
GB44871	5.488186037	6.88067849	1.47702E-28	FBgn0038074
GB41708	5.444500833	8.704285942	4.40495E-22	FBgn0036597

GB55203	5.436293714	6.175917193	1.11037E-24	FBgn0034856
GB47546	5.423008969	4.601256498	1.6752E-13	0
GB52100	5.4036091	11.37408553	8.24234E-21	FBgn0000299
GB54139	5.352604118	5.163817006	2.1002E-18	FBgn0000299
GB41222	5.260971249	3.537554868	4.12275E-08	FBgn0035132
GB40227	5.249862308	3.959180069	2.32124E-10	FBgn0036316
GB53860	5.244356987	8.970756912	7.4758E-21	FBgn0026575
GB51200	5.239484073	2.030418926	0.002704488	FBgn0005658
GB43739	5.223543827	3.488392039	4.11693E-08	FBgn0035779
GB55029	5.19400533	5.310218076	9.27766E-21	FBgn0031176
GB50880	5.192202908	3.891159108	2.75365E-10	FBgn0263968
GB43027	5.185119517	3.53256746	4.94789E-07	0
GB44633	5.171349194	2.868746884	4.05874E-05	0
GB41722	5.163085651	9.801415936	3.08826E-17	FBgn0033246
GB45713	5.115874509	3.57554981	6.95305E-08	FBgn0031975
GB43775	5.098407475	1.13412521	0.013508927	FBgn0052683
GB42554	5.062613642	5.053685002	1.53422E-18	FBgn0036191
GB40288	5.032085155	4.470607992	3.04766E-08	FBgn0033302
GB49886	5.02148742	6.659986126	9.3425E-21	FBgn0033302
GB48148	4.982300872	7.260592941	1.73232E-24	FBgn0034140
GB53930	4.929451391	0.958310946	0.025816214	FBgn0030003
GB42099	4.918199343	3.337071906	3.1647E-07	FBgn0027600
GB41717	4.892922198	1.498034732	0.004881958	FBgn0036871
GB49825	4.872403514	1.918927485	0.001134963	FBgn0024150
GB48820	4.867237402	10.56197049	9.52545E-21	FBgn0028985
GB50481	4.85457148	3.797548036	3.04454E-08	FBgn0023479
GB49442	4.834169478	3.143004307	1.19463E-05	FBgn0036237
GB52428	4.817533139	6.15890429	1.12512E-28	FBgn0037126
GB50116	4.816326234	4.185880851	8.22273E-11	FBgn0036495
GB45708	4.758042968	3.820691395	1.07009E-07	FBgn0031975
GB41369	4.756053569	1.373314989	0.008317797	FBgn0019972
GB50124	4.747934531	3.457090551	1.20688E-06	0
GB55209	4.738051108	0.746735807	0.047976525	FBgn0030647
GB51218	4.696977306	5.160933109	1.53522E-18	0
GB52919	4.679661839	7.572477237	1.17385E-18	FBgn0036617
GB44168	4.661743549	4.731122135	4.71501E-13	FBgn0000490
GB43713	4.628461871	6.490436593	2.91117E-25	FBgn0038037
GB42514	4.6141124	3.812345277	1.89458E-08	FBgn0037297
GB47127	4.6141124	3.850784416	4.66417E-08	FBgn0261832
GB40218	4.604826078	1.343097472	0.024417547	FBgn0021760
GB49552	4.60081581	5.816128621	3.49206E-14	FBgn0035501
GB48079	4.578252801	3.292208072	8.04962E-06	FBgn0051954
GB41706	4.569971291	4.756519725	6.19716E-12	FBgn0028533
GB44996	4.568998713	10.06578285	3.7612E-14	FBgn0002564
GB48391	4.554138203	3.475409395	4.80614E-05	FBgn0038135
GB51861	4.52584164	1.642645394	0.033370526	FBgn0264078
GB54404	4.525256213	6.121473284	5.49161E-26	FBgn0051522
GB48969	4.512470578	4.419453031	1.25232E-08	FBgn0025592
GB49993	4.487443702	7.448783024	5.26474E-14	FBgn0035787
GB52598	4.485618548	9.463627067	3.44998E-19	FBgn0032213
GB48130	4.435869993	0.992608776	0.023674369	FBgn0061197
GB40708	4.431139511	8.770612777	3.62844E-18	FBgn0029507
GB42985	4.427612664	6.31078854	2.78676E-12	0
GB49848	4.408373156	2.296876711	0.001375199	FBgn0038095
GB52525	4.386724966	2.490088201	0.000557293	FBgn0032248
GB54289	4.375845059	6.162550309	7.96955E-23	FBgn0085407

GB47563	4.369425745	2.62146271	0.000543316	FBgn0035575
GB47270	4.338798692	3.611306592	8.71009E-06	FBgn0015032
GB50609	4.312801166	11.42003131	2.09335E-16	FBgn0001219
GB52721	4.309893087	5.417099922	3.32546E-15	FBgn0002576
GB46230	4.291291677	2.821553607	0.002701727	FBgn0034470
GB43208	4.260919614	8.835178394	8.53189E-18	FBgn0036985
GB41283	4.255535706	6.715810621	4.65698E-21	FBgn0003137
GB45748	4.244469711	0.774011431	0.034381033	FBgn0015714
GB47398	4.237745519	1.240358494	0.046767901	FBgn0035060
GB45911	4.234400725	5.67152759	3.62627E-21	FBgn0011296
GB55206	4.230515996	1.89091984	0.009877679	FBgn0034856
GB44552	4.222721851	3.055454823	0.004084849	FBgn0005633
GB48020	4.21919196	3.773353159	2.04649E-06	FBgn0034294
GB45913	4.218993123	11.89248093	2.22149E-17	FBgn0011296
GB55204	4.201251447	5.014175878	6.25586E-10	FBgn0039896
GB42900	4.157394828	10.78508222	2.23952E-14	FBgn0261564
GB50608	4.154481829	4.959357077	7.41958E-10	FBgn0033495
GB40148	4.150354383	4.878796812	1.31826E-08	FBgn0032810
GB55208	4.147588776	2.421489874	0.002602132	FBgn0041712
GB56028	4.142724552	7.035446284	3.80592E-18	FBgn0036665
GB49441	4.133958846	7.995475552	4.83092E-16	FBgn0003450
GB50526	4.132490769	5.970333973	2.47849E-22	FBgn0038652
GB55205	4.084584352	6.224717414	2.34731E-18	FBgn0039896
GB45714	4.080289933	2.465481317	0.000846476	FBgn0031975
GB51467	4.046669938	2.113447333	0.002611818	FBgn0034140
GB47248	4.041357704	5.617043237	1.51147E-19	FBgn0031146
GB54219	4.024356569	9.739541645	1.54451E-14	FBgn0086708
GB46286	4.021911515	3.920698252	1.37578E-07	FBgn0032144
GB49440	4.008289933	6.212340223	5.1429E-22	FBgn0039630
GB46587	4.000656174	8.765332652	4.63344E-14	FBgn0053998
GB42053	3.989385319	4.867934819	4.78171E-11	FBgn0031381
GB46142	3.984553035	4.870130278	1.36367E-09	FBgn0026144
GB42146	3.954449082	7.863642439	1.16464E-10	FBgn0085446
GB42410	3.947060772	5.497416218	1.47704E-10	FBgn0034638
GB44146	3.942675169	4.345665864	1.61486E-08	FBgn0034709
GB52318	3.922582709	3.523837418	9.01187E-05	FBgn0031907
GB47805	3.90734622	5.211704137	5.02005E-13	FBgn0043575
GB49544	3.90149385	2.566429997	0.000563491	FBgn0051150
GB42135	3.883526112	4.645827292	1.20048E-10	FBgn0038201
GB54231	3.868303974	3.710862018	1.48333E-05	FBgn0024352
GB50005	3.866013539	8.528113832	3.25413E-12	FBgn0063923
GB47580	3.863875153	5.480476569	3.26204E-17	FBgn0032817
GB55068	3.841939933	3.906304886	4.46687E-07	FBgn0085351
GB47771	3.840936173	1.944451718	0.020870977	FBgn0034745
GB41418	3.820915455	4.953572289	4.65526E-11	FBgn0002526
GB42244	3.816240325	8.224996058	1.31135E-15	FBgn0043903
GB45797	3.80902794	4.244695515	1.22869E-05	FBgn0039896
GB53115	3.782584972	1.553539354	0.010832449	FBgn0036608
GB45796	3.776223883	3.656894199	2.49045E-05	FBgn0004034
GB47579	3.762041542	2.921398984	0.000881417	FBgn0016684
GB52348	3.740325191	4.051602013	5.68224E-07	FBgn0003162
GB41642	3.732489338	5.787057101	6.23524E-18	0
GB55211	3.722688172	3.711397724	0.000106108	FBgn0032601
GB48903	3.69654056	4.780609553	1.16464E-10	FBgn0038139
GB46774	3.684105765	9.572437896	5.73761E-12	FBgn0031322
GB51383	3.676908959	2.931073836	0.000832151	FBgn0033302

GB42434	3.670027302	3.172062328	0.00056252	FBgn0034580
GB55452	3.664136563	9.28669327	1.24396E-08	FBgn0052626
GB56013	3.645716342	1.467636045	0.021412554	FBgn0035849
GB40021	3.634028389	11.20050727	2.8631E-13	FBgn0036124
GB40136	3.621231566	3.668072265	2.21625E-05	FBgn0038595
GB50550	3.607729335	7.860666893	2.03532E-13	0
GB44203	3.583804144	3.691037322	0.000185038	FBgn0037465
GB55451	3.583347288	9.85017612	5.86448E-13	FBgn0002526
GB47381	3.580440394	2.230932925	0.007047794	FBgn0053817
GB54732	3.542257094	5.330627719	5.24873E-12	FBgn0030309
GB53028	3.540302413	6.592565188	4.2113E-17	FBgn0032116
GB45955	3.496577622	9.56085769	1.55015E-12	FBgn0260746
GB43362	3.481244241	6.060507373	1.93286E-16	FBgn0030421
GB55212	3.461323206	4.85905624	4.75014E-06	FBgn0039896
GB53978	3.440300342	5.680252985	1.92349E-11	FBgn0023479
GB46013	3.423557163	6.679846567	4.74407E-16	FBgn0032010
GB54983	3.38055012	3.437281979	0.000219142	FBgn0035537
GB41806	3.376257869	7.129965632	3.55751E-12	FBgn0038088
GB44043	3.353724907	3.718679835	0.00017705	FBgn0028841
GB48905	3.353066505	8.002920667	5.80005E-10	FBgn0010226
GB40573	3.349422112	3.242217054	0.00056569	FBgn0261834
GB50883	3.345391432	5.947647934	4.04394E-12	FBgn0032638
GB54941	3.344407794	6.214095738	2.68615E-16	FBgn0051720
GB43716	3.343830347	5.576841828	3.06265E-13	FBgn0038037
GB44634	3.328547019	5.453029163	3.49816E-13	FBgn0243512
GB54233	3.326655816	4.920975872	1.73649E-09	FBgn0030964
GB44503	3.324183365	4.064061312	1.42153E-05	FBgn0085201
GB52631	3.28445539	5.865409563	2.34322E-12	FBgn0003495
GB40110	3.281888653	1.962609055	0.028105464	FBgn0061200
GB54942	3.278231842	5.2066546	8.01629E-11	FBgn0051720
GB45696	3.263977743	6.939947452	7.77778E-12	FBgn0000567
GB50761	3.257571878	4.232312014	1.21401E-05	FBgn0038485
GB43728	3.211333498	6.153763169	2.12879E-16	FBgn0038037
GB46640	3.203964792	4.285154837	7.91371E-06	FBgn0033628
GB42829	3.203393446	8.656743436	1.05389E-10	FBgn0034405
GB44995	3.192159502	3.06697647	0.001253475	FBgn0051008
GB43711	3.180610135	2.475333302	0.027143461	FBgn0038037
GB55890	3.178537068	2.343760694	0.017940017	FBgn0034275
GB49672	3.166966842	4.630768683	5.96831E-08	FBgn0038842
GB55707	3.16264217	1.671755254	0.045649062	FBgn0037063
GB45157	3.158691595	5.296840875	2.70034E-11	FBgn0013753
GB52920	3.143898689	4.573804555	7.50043E-07	FBgn0036618
GB49887	3.123639142	3.666277002	0.000288346	FBgn0033304
GB41623	3.123639142	3.695902953	0.000997655	FBgn0035430
GB47407	3.068715684	3.407748549	0.000311202	FBgn0013981
GB52360	3.046777266	7.914467028	2.35717E-10	FBgn0034886
GB45495	3.028548961	10.39956753	1.79242E-08	FBgn0001233
GB44744	3.022568042	3.94833066	3.86421E-05	FBgn0036101
GB41202	3.009122368	5.67523618	9.11681E-12	FBgn0027600
GB48687	2.991603375	6.623541563	1.94255E-13	FBgn0243514
GB47142	2.988869501	9.107425573	1.69564E-09	FBgn0030608
GB47885	2.986516408	2.086291535	0.019290761	FBgn0038095
GB42217	2.978427662	3.624485009	0.000411703	FBgn0039755
GB40287	2.968203166	3.332207694	0.001901531	FBgn0033302
GB47505	2.960159413	3.251862212	0.001952641	FBgn0053870
GB49154	2.951342099	5.227631174	1.82149E-08	FBgn0040491

GB50906	2.925165439	5.993423686	1.50829E-09	FBgn0032421
GB44004	2.917412777	5.120821725	1.79643E-09	FBgn0034490
GB47940	2.914921559	9.211711449	2.62434E-09	FBgn0034096
GB52642	2.899512333	6.930959424	2.53864E-10	FBgn0030251
GB46984	2.897579484	6.606214096	7.81955E-11	FBgn0086691
GB46438	2.894976595	5.911964541	4.66699E-11	FBgn0027594
GB48260	2.879985327	2.007621968	0.035431021	FBgn0020306
GB50290	2.877621432	6.702584126	2.84695E-12	FBgn0033033
GB54097	2.873066734	8.268394461	1.61811E-08	FBgn0011672
GB49176	2.85542911	1.892584161	0.049624073	FBgn0030720
GB40967	2.844948257	5.707425686	2.50552E-08	FBgn0005626
GB48129	2.844055011	2.302889214	0.039978618	FBgn0029880
GB51671	2.843579198	6.488472885	4.27824E-12	FBgn0030869
GB47939	2.838088246	5.213224096	8.02027E-09	FBgn0040296
GB42981	2.833969186	7.65037402	2.61351E-09	FBgn0040323
GB55590	2.831345377	8.571230223	4.91221E-09	FBgn0025814
GB43708	2.815875297	12.0273355	7.28451E-09	FBgn0015221
GB53318	2.812275168	4.853331871	1.32094E-06	FBgn0001291
GB43783	2.807786682	4.808376865	4.16978E-07	FBgn0032924
GB51650	2.798500038	4.764823747	3.1647E-07	FBgn0034392
GB48634	2.796734737	8.765538906	6.12462E-09	FBgn0035438
GB48505	2.788596279	10.18140069	5.87847E-08	FBgn0026415
GB55889	2.788462626	6.298612425	9.93213E-13	FBgn0035049
GB52829	2.786237192	11.12648294	7.78996E-08	FBgn0026415
GB41301	2.781631178	9.632452562	1.20876E-08	FBgn0000083
GB55030	2.766037293	9.176040487	2.96058E-08	FBgn0046706
GB45701	2.747619187	6.283234668	1.08397E-11	FBgn0027930
GB53576	2.738183842	3.091719321	0.015034349	FBgn0261922
GB53209	2.736575937	3.36228686	0.004367363	FBgn0261565
GB49147	2.71578247	3.461338812	0.006903891	FBgn0026565
GB53865	2.707274628	6.556665741	3.60463E-11	FBgn0013984
GB55930	2.673367575	4.12908182	0.0005564	FBgn0038072
GB46749	2.672526597	2.183494292	0.035970639	FBgn0034580
GB50156	2.666219306	5.510072122	1.39477E-09	FBgn0039225
GB54315	2.664500187	3.574225364	0.020834325	FBgn0034426
GB42433	2.643400694	4.835384221	2.90084E-06	FBgn0261797
GB45248	2.604842884	7.677838973	5.52901E-08	FBgn0032180
GB53143	2.601226536	6.672144209	3.43688E-10	FBgn0016122
GB44005	2.591377149	6.650607283	2.20185E-10	FBgn0034490
GB53353	2.582823016	3.325663583	0.005959024	FBgn0013984
GB40976	2.5824245	12.42764931	1.12161E-06	FBgn0001233
GB48841	2.5794707	4.601867423	5.64078E-05	FBgn0038819
GB48194	2.574993494	4.833798002	4.47887E-06	FBgn0039755
GB43784	2.559878412	5.811206575	2.9539E-10	FBgn0035770
GB48271	2.557685721	2.614212697	0.029858682	FBgn0000210
GB55301	2.557671095	8.006589908	1.98939E-07	FBgn0028926
GB48310	2.546776968	6.380975253	2.58271E-10	FBgn0038412
GB42741	2.544734597	5.42689537	7.07896E-06	FBgn0050496
GB43330	2.543126278	8.681191514	5.49878E-06	FBgn0024319
GB40141	2.524097807	8.407504362	1.38707E-06	FBgn0038738
GB54946	2.501311077	3.672594514	0.002225344	FBgn0040299
GB47749	2.476155045	6.834551283	8.23673E-09	FBgn0039419
GB47723	2.471409945	4.356627017	0.000430984	FBgn0037387
GB47279	2.471062879	4.277200393	0.000431539	FBgn0033696
GB54541	2.470296076	6.941268607	8.14984E-08	FBgn0031973
GB50226	2.464513529	11.20120234	9.12463E-07	FBgn0022355

GB52191	2.455328265	7.784980142	1.81919E-07	FBgn0051217
GB47931	2.45108398	5.593340764	2.2396E-06	FBgn0036316
GB55070	2.44892335	6.011504344	7.18416E-09	FBgn0027843
GB53077	2.441024022	5.191849408	1.42958E-06	FBgn0039055
GB53831	2.404695561	7.088060617	5.06066E-07	FBgn0033382
GB49347	2.394552339	7.209655106	3.03427E-06	FBgn0033883
GB51345	2.392546765	8.527308799	1.83966E-06	FBgn0039151
GB49885	2.38952027	7.354137607	3.7973E-05	FBgn0033304
GB43184	2.388481877	8.261765095	7.7989E-07	FBgn0030245
GB47478	2.386709259	8.142381912	2.98117E-07	FBgn0035438
GB44055	2.372408239	6.47793325	2.90335E-08	FBgn0000250
GB53141	2.351804037	5.050829581	3.73901E-06	FBgn0033786
GB51760	2.350528836	6.123452056	9.53288E-08	FBgn0000406
GB40119	2.326643774	4.768330161	2.35027E-05	FBgn0016013
GB50508	2.325867355	6.565797434	1.89524E-08	FBgn0243514
GB41127	2.31814033	2.801415086	0.04859317	FBgn0029896
GB45023	2.309525123	7.671563593	7.14471E-06	FBgn0032783
GB43731	2.308785313	11.03661569	1.97962E-05	FBgn0015222
GB40047	2.307945371	2.925388989	0.0258441	FBgn0016034
GB46601	2.302776825	3.88104145	0.002408818	FBgn0035583
GB45875	2.294743157	4.302483579	0.000305288	FBgn0035132
GB47104	2.292987852	8.031729449	1.9437E-06	FBgn0034162
GB55302	2.289602791	9.645454339	1.19517E-05	FBgn0033644
GB55096	2.270280105	8.988512557	3.23322E-06	FBgn0002719
GB51613	2.26352513	5.299804063	6.11113E-06	FBgn0263219
GB47107	2.250564093	8.056086126	3.11065E-06	FBgn0036165
GB40508	2.250375347	5.234513261	1.01795E-05	FBgn0034804
GB46813	2.246709244	5.033387606	3.92965E-05	FBgn0010246
GB50137	2.2347321	5.25006488	4.58176E-05	FBgn0004378
GB49327	2.229762758	5.667801226	1.83588E-06	FBgn0039131
GB54949	2.224386767	5.557372079	3.48011E-07	FBgn0028978
GB48574	2.220788955	9.064840911	1.45312E-05	FBgn0040070
GB48344	2.210939256	4.910107353	4.25665E-05	FBgn0025631
GB54947	2.210652258	4.773003791	5.88609E-05	FBgn0036235
GB41736	2.207930186	3.945938813	0.003519995	FBgn0013988
GB42558	2.193236683	4.166786146	0.002609378	FBgn0041585
GB46612	2.192135703	4.916009974	5.71534E-05	FBgn0033936
GB50448	2.182410323	4.90453721	0.000145007	FBgn0039804
GB45688	2.181388866	9.226134947	1.11722E-05	FBgn0033883
GB48436	2.16576965	6.689283968	4.88175E-07	FBgn0002567
GB43722	2.159158018	4.115336738	0.007558904	FBgn0085428
GB54294	2.156805292	5.565148179	4.8342E-06	FBgn0035348
GB48195	2.156662459	7.108344829	2.32304E-06	FBgn0086687
GB52810	2.151482677	5.943843746	1.06052E-07	FBgn0039896
GB49775	2.144655139	9.143809303	4.63529E-05	FBgn0011296
GB46277	2.13926228	3.048550999	0.035970639	FBgn0029720
GB50009	2.137305528	7.865445712	1.15806E-05	FBgn0040532
GB44167	2.133758853	4.683324983	0.000626133	FBgn0033240
GB51724	2.133319291	3.481529894	0.017556664	FBgn0039768
GB49614	2.126949416	6.002253504	9.14651E-08	FBgn0250732
GB52074	2.12402376	7.929469905	8.17755E-06	FBgn0004654
GB48029	2.118217726	6.005336906	1.16292E-07	FBgn0032219
GB51043	2.114046043	4.540287046	0.000919202	FBgn0023535
GB49849	2.113382523	3.54415973	0.017777857	0
GB43573	2.106395821	7.821363432	9.02469E-06	FBgn0032638
GB50418	2.104413179	6.556788702	1.67651E-06	FBgn0262473

GB50522	2.094976191	3.582740531	0.013812464	FBgn0037772
GB54367	2.09236012	5.135405989	2.79392E-05	FBgn0030884
GB42084	2.082433986	6.545450764	3.49866E-07	FBgn0035132
GB51238	2.079954027	4.39762049	0.001395313	FBgn0086687
GB53620	2.06634396	3.728535081	0.010054731	FBgn0261800
GB51741	2.056396521	7.275547169	7.12059E-06	FBgn0030310
GB45700	2.054735324	9.720837301	4.08753E-05	FBgn0000533
GB47382	2.05393817	4.639141854	0.001003108	FBgn0013981
GB44967	2.053157164	5.414403992	1.73124E-05	FBgn0042094
GB51665	2.043654576	7.928786975	1.65796E-05	FBgn0032074
GB44214	2.041632721	5.032131674	0.000130132	FBgn0014141
GB55701	2.033330748	8.024249696	0.001960991	FBgn0036857
GB54611	2.026796313	9.766579599	0.000332084	FBgn0028984
GB42426	2.011564458	5.896959627	0.000230656	FBgn0039640
GB47463	1.996129804	7.280448987	1.37335E-05	FBgn0001104
GB53549	1.991868575	5.152414313	0.000368142	FBgn0031037
GB44803	1.986044129	6.249081109	1.57976E-06	FBgn0035904
GB41387	1.98419856	3.421073636	0.044833816	FBgn0001128
GB55007	1.975233498	3.8877172	0.011059972	FBgn0030051
GB47301	1.973918371	5.576004469	7.44566E-06	FBgn0034605
GB50421	1.96913005	10.70839518	8.96581E-05	FBgn0000416
GB51551	1.964624405	7.880590083	3.89829E-05	FBgn0035499
GB42692	1.962737733	4.9884136	0.0001877	FBgn0003964
GB44213	1.961263004	7.6253422	5.74332E-05	FBgn0014141
GB55016	1.960335222	10.13991115	0.000116802	FBgn0033188
GB45704	1.955976659	6.344611025	8.54012E-07	FBgn0053229
GB53847	1.950861149	6.490226443	3.09944E-06	FBgn0014906
GB47380	1.944580571	3.574443908	0.028410267	FBgn0053812
GB40759	1.939667617	9.566643106	0.000119228	FBgn0043841
GB47849	1.938747527	6.726869437	4.563E-06	FBgn0038516
GB53221	1.93104962	4.734193308	0.004123881	0
GB48626	1.930702257	7.102891378	0.00014207	FBgn0031528
GB50043	1.927030371	6.512773407	4.41831E-06	FBgn0259178
GB52115	1.920480994	7.970607217	0.000246586	FBgn0025456
GB46686	1.906499069	5.237685228	0.000221675	FBgn0033913
GB43504	1.906221491	3.502773445	0.047534262	FBgn0001257
GB49688	1.904766714	8.835782329	0.000173641	FBgn0004577
GB45453	1.900114615	4.375433593	0.004765012	FBgn0027655
GB40758	1.893759711	8.728529083	0.000230656	FBgn0033926
GB50598	1.888694368	9.20520048	0.001220022	FBgn0086254
GB47310	1.881735437	6.057842518	4.11344E-06	FBgn0030791
GB54426	1.878967052	6.558567527	0.00034774	FBgn0052512
GB50603	1.858096604	4.912479321	0.000448154	FBgn0050410
GB49039	1.848302779	3.617198244	0.039564026	FBgn0034433
GB54051	1.843234599	5.758371002	3.2861E-05	FBgn0263607
GB40565	1.842422768	6.104784694	4.05701E-06	FBgn0262866
GB52341	1.836489164	6.620456028	1.82809E-05	FBgn0013987
GB55998	1.834620151	6.090672565	6.83392E-06	FBgn0027538
GB53755	1.832279537	5.186782415	0.001571017	FBgn0029690
GB53550	1.823698127	6.615005939	1.67223E-05	FBgn0031037
GB49899	1.819554507	4.240100993	0.011685874	FBgn0263846
GB47055	1.816639189	5.907892793	7.98283E-05	FBgn0031307
GB52496	1.81246692	4.857555006	0.000788752	FBgn0026570
GB50136	1.812271027	8.512974499	0.000448154	FBgn0037007
GB42865	1.811689513	5.665971137	3.80149E-05	FBgn0036732
GB45639	1.803315477	4.28009955	0.012166729	FBgn0005612

GB48598	1.798350722	6.440417387	1.42062E-05	FBgn0011674
GB42466	1.796843167	4.934907305	0.000819164	FBgn0031760
GB53244	1.795398588	4.238575421	0.010928607	FBgn0039621
GB55511	1.788500841	5.868736691	2.3657E-05	FBgn0031461
GB45052	1.788222332	7.263972263	0.0002379	FBgn0026376
GB47880	1.78630048	8.934168755	0.000482768	FBgn0003462
GB54480	1.782616437	5.001928209	0.000760205	FBgn0032515
GB41807	1.772751262	8.08308626	0.000259189	FBgn0030740
GB52158	1.769398477	6.495262818	9.83534E-05	FBgn0031975
GB47415	1.768750366	7.324896218	0.000138065	FBgn0031538
GB52630	1.758594443	5.386078863	0.000304575	FBgn0036101
GB53412	1.742990569	7.578053815	0.00040248	FBgn0027571
GB45676	1.74149265	5.596896894	0.000134896	FBgn0035617
GB41837	1.732361422	4.151924236	0.017558695	FBgn0038475
GB42738	1.729979022	5.871685367	3.59322E-05	FBgn0011204
GB50010	1.729620237	4.632726678	0.004213415	FBgn0035575
GB44143	1.723335605	6.379382729	0.000419405	FBgn0037022
GB55805	1.720164605	5.638377505	8.82373E-05	FBgn0050118
GB43572	1.710501642	8.143397849	0.000428948	FBgn0015575
GB45497	1.710047263	6.806448456	0.00024558	FBgn0050296
GB51210	1.702360716	6.828811341	0.000264076	FBgn0039492
GB45617	1.699660232	6.704421814	0.000175666	FBgn0028343
GB43945	1.699186103	6.175911844	2.88026E-05	FBgn0061200
GB45135	1.692933917	6.651752742	0.000137174	FBgn0028982
GB45736	1.689019485	7.536162532	0.000508	FBgn0016693
GB47486	1.685862292	3.763406661	0.046153396	FBgn0053812
GB44344	1.683442476	7.142846424	0.000277567	FBgn0053120
GB49607	1.678602919	9.772922401	0.001012482	FBgn0032949
GB41313	1.678408903	6.987614632	0.000345352	FBgn0036501
GB40029	1.674247978	4.483353588	0.014910936	FBgn0011741
GB42306	1.671981372	5.326030244	0.003796021	FBgn0262526
GB53401	1.671613893	6.179116109	6.25873E-05	FBgn0028550
GB52278	1.669693371	5.549035072	0.000327489	FBgn0014141
GB42141	1.665347376	9.520344946	0.001308977	FBgn0035811
GB49928	1.661229261	5.504866617	0.000420644	FBgn0036546
GB52275	1.659053074	4.254519987	0.022351267	FBgn0029831
GB46367	1.647948063	6.763390394	0.001155083	FBgn0011693
GB48052	1.644831807	5.229228204	0.001683796	FBgn0035542
GB49390	1.644723348	5.711111102	0.000257354	FBgn0028341
GB42057	1.634176909	4.934777062	0.00358227	FBgn0037121
GB41388	1.634021247	6.339486462	0.0001143	FBgn0001128
GB50970	1.617261356	9.270423806	0.001538639	FBgn0040064
GB49580	1.614436247	5.03832494	0.002520069	FBgn0033452
GB44882	1.612159539	6.96806401	0.000730239	FBgn0035094
GB42329	1.610431039	7.239212633	0.000690956	FBgn0262975
GB46766	1.606718597	7.390153626	0.000953955	FBgn0034577
GB41604	1.600856228	7.913373129	0.001253475	FBgn0030529
GB40083	1.593700827	5.81558463	0.000193878	FBgn0032358
GB45147	1.592980826	8.710482222	0.002378389	FBgn0031913
GB41293	1.589913035	5.987711527	0.000500208	FBgn0014340
GB49773	1.589554962	9.224053746	0.002004696	FBgn0003231
GB53567	1.587724356	4.429596857	0.01621565	FBgn0030482
GB40603	1.58702848	6.105505932	0.000109203	FBgn0022800
GB43560	1.57876302	6.336713024	0.000759524	FBgn0044050
GB44774	1.574501083	4.028352092	0.046671814	FBgn0032076
GB43353	1.573794531	4.785741976	0.006395335	FBgn0035490

GB41965	1.566677061	9.7399938	0.007844704	FBgn0031696
GB49774	1.56215425	5.435149366	0.010229281	0
GB45350	1.560830817	6.090451343	0.000230656	FBgn0261642
GB43188	1.559286102	4.955739734	0.005486985	FBgn0034602
GB47740	1.555864962	8.430953695	0.002840741	FBgn0036995
GB41735	1.549957471	7.298462091	0.001350043	FBgn0036565
GB53805	1.545114255	6.311605444	0.000235734	FBgn0038098
GB55544	1.539884833	6.664310086	0.000649808	FBgn0261274
GB52454	1.539412198	4.254189392	0.029946304	FBgn0037714
GB51220	1.532092946	4.601239242	0.016647277	FBgn0032801
GB43409	1.525881956	5.401496075	0.004074571	FBgn0025679
GB43920	1.523189795	6.041191919	0.000244927	FBgn0034958
GB44045	1.519703613	8.145967027	0.00983451	FBgn0015924
GB40866	1.518575295	13.03370776	0.004329948	FBgn0001219
GB54056	1.515605703	7.246440104	0.002476793	FBgn0029823
GB49339	1.50675214	4.878401263	0.008362034	FBgn0050345
GB48672	1.506074616	5.547163564	0.000835556	FBgn0037697
GB53428	1.50575897	4.690186488	0.016023649	FBgn0031261
GB55103	1.505041032	5.371246411	0.002250147	FBgn0039509
GB40718	1.504945569	8.999698772	0.004656986	FBgn0020653
GB45038	1.499939271	6.357068925	0.002897913	FBgn0037010
GB55440	1.495676338	6.462597697	0.000540393	FBgn0262468
GB54331	1.4941728	10.00474234	0.003795692	FBgn0013770
GB50430	1.482300203	5.391753882	0.002360588	FBgn0263929
GB47929	1.481604414	5.059150866	0.009619571	FBgn0025686
GB49757	1.480038536	9.162650163	0.004367363	FBgn0037913
GB44613	1.47769509	4.586441905	0.042379453	FBgn0000320
GB55482	1.474462222	7.19755672	0.002754428	FBgn0010620
GB43831	1.469512207	6.618233856	0.001421829	FBgn0031069
GB53043	1.468088902	7.612906594	0.00560765	FBgn0020762
GB41915	1.466239687	5.135250682	0.007923136	FBgn0032839
GB40673	1.462524178	7.408355088	0.003396783	FBgn0030737
GB42500	1.461817268	6.55992124	0.00102768	FBgn0035976
GB40727	1.461647884	6.009703692	0.000656307	FBgn0030670
GB52702	1.456445811	7.794704914	0.004545604	FBgn0034909
GB48407	1.456344073	4.162074899	0.049060018	FBgn0003964
GB48883	1.452127706	5.394706655	0.004621279	FBgn0264652
GB49259	1.44641993	7.57718716	0.017161757	FBgn0030872
GB51125	1.446131205	8.084843532	0.010832449	FBgn0025885
GB46034	1.445686871	4.947508674	0.011079908	FBgn0036341
GB42940	1.445632046	7.523728447	0.004074571	FBgn0013305
GB40232	1.443286249	10.43189364	0.006100696	FBgn0040309
GB43964	1.442067253	4.679538527	0.016622352	FBgn0019662
GB48255	1.438217261	6.878070248	0.003236525	FBgn0039850
GB45735	1.434551295	4.713038379	0.036487496	FBgn0038912
GB49123	1.433946995	6.762289926	0.002560814	FBgn0035719
GB44081	1.432426206	6.484216888	0.001086478	FBgn0035674
GB46276	1.431197713	8.288848854	0.005555027	FBgn0033799
GB49657	1.430288649	6.986532855	0.004890939	FBgn0004606
GB47432	1.428230057	6.40022197	0.005900023	FBgn0020764
GB42424	1.421068509	4.929178613	0.018402057	0
GB44751	1.420894758	8.032471384	0.00652745	FBgn0032167
GB47394	1.420246209	5.015852545	0.017667254	FBgn0039714
GB52033	1.412234353	6.009690198	0.001589126	FBgn0261258
GB46266	1.409964425	5.535317007	0.003309054	FBgn0016126
GB55455	1.39923273	7.012697393	0.006049808	FBgn0034223

GB53245	1.398118849	6.949952584	0.004048037	FBgn0034372
GB42685	1.390205187	6.752220877	0.004501932	FBgn0040323
GB49826	1.389640716	7.146546392	0.010436375	FBgn0037612
GB50900	1.388870248	4.887409179	0.01940528	FBgn0017482
GB41818	1.384853088	8.097453243	0.007585319	FBgn0031992
GB47565	1.384706875	9.224798921	0.020649469	FBgn0023095
GB44100	1.381502844	6.087420138	0.000823004	FBgn0013733
GB42608	1.378916319	6.259784799	0.001512742	FBgn0264294
GB50149	1.375249068	6.972429483	0.012282567	FBgn0040827
GB48812	1.370634569	7.202377352	0.00567286	FBgn0037718
GB42168	1.367423815	7.919587956	0.009983198	FBgn0033906
GB48853	1.357080547	7.602262634	0.010157995	FBgn0032456
GB42831	1.356251552	6.323366197	0.002064591	FBgn0039633
GB50096	1.353657489	5.809616254	0.002897913	FBgn0011205
GB55420	1.34152846	6.959525494	0.00801343	FBgn0086378
GB40735	1.338393359	9.656483378	0.015144064	FBgn0000064
GB47201	1.338367078	8.520816061	0.012564959	FBgn0004657
GB50902	1.337565936	10.51012594	0.01418611	FBgn0001091
GB51753	1.335679912	6.98822318	0.012731145	0
GB42142	1.335551959	5.833196897	0.004037997	FBgn0001078
GB49240	1.333054551	7.386724288	0.009042772	FBgn0012036
GB40577	1.332181909	6.388552428	0.002897913	FBgn0040319
GB45678	1.331377882	5.87718428	0.002286054	FBgn0033226
GB50680	1.330010866	6.527626212	0.002921484	FBgn0031662
GB54507	1.322289104	10.62886709	0.021624861	FBgn0087002
GB48884	1.319334381	4.736046532	0.046886594	FBgn0264652
GB41778	1.314123968	6.445943154	0.012282567	FBgn0038257
GB55369	1.312884777	6.599655232	0.009958458	FBgn0033177
GB44511	1.311876247	5.677498599	0.003618433	FBgn0262699
GB50784	1.308604219	5.152258565	0.022090803	FBgn0051922
GB49539	1.307315544	5.336717016	0.016192646	FBgn0038055
GB40909	1.305433205	6.227288652	0.003780591	FBgn0032476
GB40578	1.303971262	6.656999187	0.005945219	FBgn0028703
GB53023	1.302329562	5.554572789	0.010302269	FBgn0035147
GB51273	1.301101717	7.07371217	0.012746549	FBgn0037949
GB40899	1.296476763	4.898096088	0.035464295	FBgn0030485
GB46318	1.293448851	7.572599834	0.024586358	FBgn0053126
GB55242	1.28876365	5.719658247	0.006160752	FBgn0050269
GB54863	1.286638782	7.456004563	0.014423799	FBgn0250848
GB42354	1.286578672	7.182977424	0.011322293	FBgn0038745
GB40773	1.286418685	7.458326923	0.012850387	FBgn0030485
GB50271	1.286292923	6.949898332	0.009951578	FBgn0035432
GB50513	1.276750906	8.207761356	0.020437464	FBgn0035964
GB45666	1.272657116	5.191573089	0.037131754	FBgn0038306
GB46537	1.27189377	6.308343691	0.003284002	FBgn0000477
GB40861	1.266913324	4.696116875	0.047639806	FBgn0030026
GB51251	1.25577307	6.011471513	0.003618433	FBgn0037551
GB46184	1.253831172	7.511944517	0.024895689	FBgn0261283
GB46048	1.253736278	5.311163359	0.019866514	FBgn0016641
GB51683	1.253672168	9.594598659	0.024873367	FBgn0000083
GB51740	1.248316699	6.693369515	0.011251899	FBgn0032943
GB40534	1.245534674	6.098318041	0.004003967	FBgn0023407
GB48059	1.244609691	5.078565966	0.035253719	FBgn0000546
GB46201	1.244609691	5.075564562	0.031970319	FBgn0036843
GB44537	1.242255043	6.733509687	0.012731145	FBgn0086347
GB45648	1.239840337	5.56942723	0.012694262	FBgn0013983

GB54968	1.233855576	5.427015792	0.011911289	FBgn0033380
GB54123	1.233155071	5.140908418	0.030214207	FBgn0034045
GB42327	1.231227174	5.938912294	0.005380204	FBgn0262975
GB49352	1.229365149	5.47244542	0.012746549	FBgn0037986
GB49419	1.228287264	5.122936021	0.032373414	FBgn0037445
GB46218	1.227307984	6.076409763	0.015049345	FBgn0034094
GB42281	1.225584137	5.905643916	0.00589524	FBgn0032482
GB51503	1.223991933	4.992696841	0.03991821	FBgn0003256
GB45456	1.22078705	5.587916533	0.011333035	FBgn0264357
GB52793	1.219099679	6.0407688	0.006067345	FBgn0029896
GB55529	1.213704403	6.32183642	0.007340172	FBgn0032704
GB55575	1.213230355	5.438299936	0.0209588	FBgn0040238
GB53046	1.211373435	6.305151923	0.006012	FBgn0031220
GB43130	1.211296171	6.271607509	0.008213492	FBgn0259245
GB54764	1.20849777	5.279512373	0.029884272	FBgn0026404
GB54052	1.207342153	5.003454174	0.044245709	FBgn0036273
GB43392	1.20101445	5.446434189	0.015637241	FBgn0261436
GB49519	1.200113596	6.083152484	0.009484375	FBgn0029911
GB40799	1.19717594	6.04601078	0.006814952	FBgn0035113
GB54369	1.196648045	6.60876483	0.019304423	FBgn0086355
GB50857	1.196470036	6.424357636	0.011119052	FBgn0027581
GB42616	1.180322193	6.725762328	0.02613731	FBgn0041629
GB42614	1.179742711	6.232920111	0.007729033	FBgn0032230
GB47395	1.176107368	7.073018159	0.023600221	FBgn0039714
GB46068	1.174426312	6.215183188	0.008374536	FBgn0060296
GB53540	1.173802877	6.813159904	0.024275471	FBgn0263350
GB54861	1.173744567	6.987155572	0.024018463	FBgn0250848
GB53146	1.173304644	6.436152547	0.011149329	FBgn0039130
GB41603	1.17234126	8.487817239	0.042295373	FBgn0029092
GB48111	1.171860979	7.905428356	0.030189778	FBgn0002284
GB54934	1.17130737	5.545638822	0.020622141	FBgn0063485
GB53532	1.168238459	5.316593671	0.038752876	FBgn0044826
GB50730	1.164890035	8.734308656	0.04438076	FBgn0026418
GB41836	1.164356928	7.361864066	0.030216143	FBgn0037218
GB44804	1.163338389	5.839109083	0.011767196	FBgn0035235
GB47626	1.161184917	5.240341417	0.046322291	FBgn0030052
GB42995	1.16026196	5.842164575	0.016540525	FBgn0036887
GB52510	1.159307148	5.830533405	0.020235468	FBgn0033266
GB46580	1.151289689	5.92245542	0.011028886	FBgn0036141
GB44710	1.148555381	7.050194686	0.028729238	FBgn0037684
GB48993	1.148215278	5.267979249	0.048988963	FBgn0013772
GB41027	1.147443462	5.82343713	0.010655601	FBgn0036505
GB40745	1.14450824	5.446355556	0.021573026	FBgn0034913
GB51071	1.143056642	5.663698915	0.016689492	FBgn0033735
GB42969	1.142885745	6.868172227	0.041271236	FBgn0030452
GB46270	1.13823165	6.496888313	0.015372174	FBgn0025740
GB47638	1.136831184	6.700993917	0.031382455	FBgn0032292
GB47159	1.134360688	6.573678727	0.016360985	FBgn0019972
GB52427	1.134136635	7.3515801	0.036066988	FBgn0024238
GB40335	1.132387744	6.154274658	0.011973939	FBgn0039774
GB40251	1.131974994	5.909385167	0.029423819	FBgn0003396
GB45024	1.129002919	5.319854226	0.04224453	FBgn0050051
GB40978	1.127653183	7.39471128	0.035114232	FBgn0029687
GB41591	1.125888277	5.488606003	0.04251763	FBgn0028540
GB52710	1.124449566	5.766512978	0.024153443	0
GB48378	1.121823036	5.924099539	0.014780106	FBgn0053526

GB50534	1.121362629	6.486323713	0.018196636	FBgn0039044
GB54753	1.121050656	8.390878625	0.049624073	FBgn0000579
GB41520	1.120383704	6.089688118	0.015999393	FBgn0001139
GB50813	1.120158139	6.655635052	0.020916376	FBgn0023172
GB55535	1.119684756	6.068305757	0.011439352	FBgn0003410
GB55426	1.119670368	8.598142509	0.049930288	FBgn0087007
GB44312	1.119572643	5.557461875	0.021573026	FBgn0036988
GB53415	1.118849406	7.269183498	0.036803932	FBgn0036318
GB54673	1.118069875	5.29076026	0.048535828	FBgn0027865
GB44980	1.116888669	7.450498114	0.046767901	FBgn0032910
GB51496	1.115520259	5.388269267	0.041068296	FBgn0250819
GB47482	1.112331117	5.562823016	0.022420423	FBgn0053843
GB44976	1.109609273	6.153159044	0.017642616	FBgn0016076
GB46275	1.10894249	5.345455066	0.03994067	FBgn0004106
GB47614	1.10499348	6.091221385	0.015034349	FBgn0259163
GB41549	1.104101273	5.842473419	0.021818503	FBgn0011837
GB52742	1.103335046	6.568441269	0.021636336	FBgn0020521
GB52152	1.101825405	5.974614624	0.018799562	FBgn0034894
GB54083	1.099193142	5.396161251	0.04881314	FBgn0004395
GB45775	1.098032092	5.72700975	0.034437145	FBgn0034166
GB41808	1.097253494	7.160436735	0.040346151	FBgn0051694
GB54158	1.093729679	5.376931035	0.04422699	FBgn0260933
GB42000	1.088451198	6.386379099	0.043473243	FBgn0000479
GB52725	1.08570616	6.014595026	0.018851229	FBgn0034410
GB52189	1.085039156	6.132758409	0.017339984	FBgn0037989
GB50375	1.084028542	5.347770219	0.036487496	FBgn0038275
GB54268	1.083527193	6.441396876	0.026095399	FBgn0031589
GB46962	1.078675167	7.206145873	0.045032573	FBgn0035772
GB49813	1.074075996	5.704131798	0.028622677	FBgn0029512
GB49471	1.073026654	6.838428406	0.044386874	FBgn0032509
GB50976	1.071830647	6.544537032	0.037199309	FBgn0083919
GB46896	1.071778389	6.130635572	0.026567657	FBgn0010531
GB54852	1.069949516	6.704303617	0.039651064	FBgn0032006
GB49949	1.067284231	6.594705087	0.028324965	FBgn0035850
GB42417	1.06535729	5.382667189	0.049111869	FBgn0052280
GB40540	1.065055849	6.096247979	0.034012097	FBgn0033916
GB44658	1.060894068	6.111128013	0.016940699	FBgn0030183
GB44789	1.060391151	5.921818184	0.02774923	FBgn0034118
GB49284	1.055899362	6.300733247	0.037491624	FBgn0262516
GB55259	1.055203786	5.652619026	0.03533313	FBgn0033987
GB41225	1.050294365	6.314424255	0.020778984	FBgn0033635
GB50265	1.049854755	6.442952083	0.033163144	FBgn0001149
GB50731	1.048650681	5.620521594	0.031929106	FBgn0036932
GB54164	1.043821295	6.16912726	0.021624861	FBgn0051145
GB41660	1.042000064	5.695202681	0.048698801	FBgn0026199
GB51019	1.037701223	6.598142726	0.035842958	FBgn0261554
GB45040	1.035354007	6.336693735	0.038239344	FBgn0043364
GB54188	1.035149606	5.880023628	0.028519506	FBgn0031950
GB40999	1.022216192	5.873047122	0.035253719	FBgn0031030
GB51188	1.019919727	6.543217713	0.037079303	FBgn0033476
GB41036	1.018191537	5.888520105	0.035423337	FBgn0015558
GB55287	1.000093222	5.800387848	0.04591511	FBgn0037012
GB48126	0.998497574	6.302054787	0.044386874	FBgn0030869
GB42726	0.995500512	6.051358152	0.0334288	FBgn0040877
GB46291	0.98972851	6.571530756	0.04627052	FBgn0037063
GB46921	0.986818613	6.189435782	0.042026859	FBgn0033028

GB41916	0.976231107	5.861619472	0.044372243	FBgn0051248
GB53070	0.959701668	6.195777984	0.039988438	FBgn0052380
GB40417	0.956070153	6.063746244	0.044731037	FBgn0030769
GB50218	-1.012131746	6.056209397	0.046767901	FBgn0022774
GB44445	-1.041599118	6.239267301	0.038048933	FBgn0031868
GB43628	-1.054044759	6.220456881	0.048698801	FBgn0035445
GB45612	-1.061611644	6.634119751	0.039793879	FBgn0004368
GB45623	-1.069688374	6.567795127	0.038013647	FBgn0000709
GB48102	-1.072152187	6.441951786	0.035550663	FBgn0040507
GB42196	-1.073316628	6.365553122	0.038155607	FBgn0032252
GB52743	-1.074408317	6.125640023	0.031647061	FBgn0004875
GB41901	-1.082158808	6.430916733	0.037072658	FBgn0035228
GB40517	-1.090372837	6.250403671	0.031432017	FBgn0029708
GB41882	-1.091136152	6.568340348	0.034265445	FBgn0029936
GB52618	-1.098239309	6.644326341	0.042689394	FBgn0038744
GB44959	-1.100153517	6.29508967	0.040118612	0
GB44679	-1.102910446	7.128541459	0.04830927	FBgn0027951
GB52611	-1.102926689	6.091429189	0.024018463	FBgn0034606
GB42147	-1.106997012	6.218149328	0.023780243	FBgn0030600
GB45120	-1.110168478	5.749263607	0.034173827	FBgn0003187
GB49382	-1.113108827	6.4931503	0.02825128	FBgn0004870
GB51337	-1.114950555	6.98474651	0.042059907	FBgn0264324
GB45313	-1.11659392	6.407172448	0.030186652	FBgn0036481
GB55822	-1.11970099	7.000383765	0.042455583	FBgn0261360
GB44673	-1.120362674	6.571898678	0.032373414	FBgn0044452
GB43537	-1.124692819	5.814746452	0.038574722	FBgn0033907
GB50409	-1.127321863	5.979608384	0.021650288	FBgn0038464
GB43238	-1.129390242	8.083153883	0.046004616	FBgn0039120
GB47596	-1.129460947	6.110160203	0.02119483	FBgn0032517
GB50232	-1.133219492	5.893343876	0.024978403	FBgn0039738
GB54092	-1.135225778	6.918048466	0.039909932	FBgn0010825
GB46700	-1.137264946	7.756301423	0.049624073	FBgn0263077
GB54780	-1.138586649	6.282125538	0.023080943	FBgn0261787
GB46630	-1.141466159	6.959800842	0.03738176	FBgn0086697
GB41909	-1.148107079	6.022757035	0.020778984	FBgn0041161
GB46526	-1.148558157	7.126850185	0.034502649	FBgn0035802
GB52472	-1.15406159	6.9317087	0.033963531	FBgn0023081
GB55003	-1.155485683	6.188192346	0.027961434	FBgn0036801
GB48994	-1.159477816	6.232466606	0.02057185	FBgn0015278
GB46614	-1.16058101	8.195650489	0.044187803	FBgn0053208
GB45318	-1.162340166	8.738660085	0.043473243	FBgn0052499
GB48533	-1.163515871	7.757863818	0.036803932	FBgn0004401
GB49960	-1.167159043	5.97372302	0.018508126	FBgn0030895
GB53948	-1.167340633	7.685406905	0.036388284	FBgn0037060
GB54301	-1.169036464	6.211080721	0.049687319	FBgn0030605
GB51748	-1.172202865	6.139665635	0.015634162	FBgn0029157
GB50515	-1.17456726	6.509490554	0.019144509	FBgn0025702
GB44155	-1.175643784	7.377109885	0.031037333	FBgn0039728
GB43108	-1.18035789	5.801670357	0.023892958	FBgn0034689
GB53672	-1.180629474	9.152513621	0.037240713	FBgn0014163
GB41803	-1.184343478	7.891400741	0.032167223	FBgn0262519
GB42579	-1.18434354	5.990062933	0.013813425	FBgn0262738
GB41239	-1.185331188	6.660198177	0.031706919	FBgn0034878
GB53323	-1.185592961	5.378406057	0.045630493	FBgn0045063
GB40699	-1.186509033	6.463193837	0.01949561	FBgn0029114
GB43207	-1.188832001	5.966674662	0.015728672	FBgn0261260

GB54731	-1.188938142	5.504930024	0.041196655	FBgn0035799
GB45881	-1.195021553	6.585352619	0.021266743	FBgn0259166
GB51494	-1.199238275	8.673996346	0.037716129	FBgn0034356
GB48851	-1.202566812	5.751610641	0.020778984	FBgn0011225
GB42356	-1.203358252	7.191768768	0.024037459	FBgn0051550
GB41629	-1.203674531	6.918572303	0.024492936	FBgn0025936
GB40075	-1.20504542	6.39578422	0.01498088	FBgn0262743
GB46479	-1.210921957	7.038816931	0.022420423	FBgn0044324
GB42740	-1.213115885	6.145190381	0.011439352	FBgn0038341
GB53753	-1.213450513	5.789472175	0.019304423	FBgn0030796
GB49957	-1.215418198	5.65147826	0.022636454	FBgn0044328
GB47106	-1.215476367	8.118396067	0.027727402	FBgn0017566
GB47181	-1.217244861	6.9266721	0.026735486	FBgn0031021
GB46725	-1.219719723	5.687422248	0.022709641	FBgn0259211
GB44180	-1.221936257	6.147861591	0.010787971	FBgn0015600
GB44404	-1.229301128	5.77548772	0.014091796	FBgn0050491
GB41740	-1.231055325	6.295138907	0.01414006	FBgn0021760
GB53125	-1.231793658	6.748006595	0.022636454	FBgn0015391
GB49584	-1.235162372	6.531371181	0.026554905	FBgn0086712
GB50202	-1.236252376	7.693565796	0.024492936	FBgn0028577
GB42567	-1.236348341	5.843954947	0.012926377	FBgn0259175
GB53790	-1.237113935	5.96926544	0.010655601	FBgn0052758
GB49098	-1.237939411	5.503061996	0.024018463	FBgn0020445
GB49777	-1.239002341	5.288817788	0.026845832	FBgn0040336
GB45717	-1.239127996	7.36273207	0.021701636	FBgn0000317
GB42959	-1.243909593	5.518304858	0.017161757	FBgn0000183
GB53565	-1.24596045	7.160689741	0.023098629	FBgn0038180
GB55578	-1.249561296	6.093419072	0.008352991	FBgn0040752
GB43099	-1.252162514	6.691808527	0.018078672	FBgn0028982
GB55586	-1.253446286	7.309311641	0.020103818	FBgn0053196
GB42043	-1.254019863	6.518913022	0.021573026	FBgn0051159
GB54130	-1.255549034	5.780899577	0.020778984	FBgn0053554
GB49749	-1.256603555	5.855757854	0.011952812	FBgn0039589
GB51800	-1.257015945	7.453361524	0.023988253	FBgn0015903
GB50870	-1.26229273	5.306575082	0.020469687	FBgn0034001
GB40489	-1.263044202	7.481343758	0.021961571	FBgn0019957
GB40348	-1.263953025	7.296472514	0.021637974	FBgn0015589
GB52948	-1.264431888	5.60416818	0.020778984	FBgn0041706
GB51775	-1.276459777	7.370072505	0.017161757	FBgn0011225
GB47675	-1.280087721	6.010777638	0.013003728	FBgn0003089
GB47661	-1.280837392	6.000393509	0.009916574	FBgn0003423
GB40519	-1.280986069	5.940916802	0.009453552	FBgn0032741
GB53305	-1.282806066	6.036530646	0.007363876	FBgn0085447
GB54474	-1.283053224	6.633595265	0.022754713	FBgn0043070
GB52985	-1.285546977	8.710335246	0.020733401	FBgn0043884
GB43798	-1.287314212	6.981249215	0.014780106	FBgn0000542
GB45413	-1.291093552	5.705250554	0.015747903	FBgn0085403
GB45017	-1.293590335	6.521714156	0.007585319	FBgn0051719
GB50158	-1.294061927	11.04771897	0.017825316	FBgn0003279
GB50213	-1.296872534	5.939055738	0.016152704	FBgn0033816
GB50675	-1.297527699	6.824507407	0.015742296	FBgn0259978
GB50407	-1.298133097	5.509931491	0.019290761	FBgn0000163
GB42098	-1.30306931	5.267654284	0.036487496	FBgn0001311
GB45564	-1.307976968	6.786357924	0.012417585	FBgn0027585
GB44772	-1.31220623	7.502093681	0.01472248	FBgn0031515
GB50408	-1.313203247	6.190019337	0.020535848	FBgn0036726

GB48425	-1.31333077	7.249076502	0.015935842	FBgn0050147
GB44482	-1.313901	5.998030491	0.008198544	FBgn0261794
GB49067	-1.314248218	6.539135302	0.007386361	FBgn0033782
GB51966	-1.316208278	5.44209607	0.013757462	FBgn0038968
GB49836	-1.32192498	6.017423732	0.007340172	FBgn0053265
GB55755	-1.322005268	5.481026878	0.016373131	FBgn0003016
GB40741	-1.324182256	5.808441828	0.007905609	FBgn0052133
GB44656	-1.324599996	5.908082015	0.006582239	FBgn0000289
GB40865	-1.326014805	9.634918979	0.015770532	FBgn0061197
GB54226	-1.329281081	5.394602537	0.02045903	FBgn0262029
GB52827	-1.332380219	6.901883198	0.010495245	FBgn0260742
GB47177	-1.333373265	6.266668573	0.009981156	FBgn0037549
GB48192	-1.334895011	5.919488278	0.005016816	FBgn0003016
GB42564	-1.338372145	6.253730303	0.00567286	FBgn0042693
GB48028	-1.342521388	5.707945005	0.010612633	FBgn0023076
GB52106	-1.343459015	5.674654706	0.009248317	FBgn0004885
GB44333	-1.347141056	9.250815273	0.014423799	FBgn0034253
GB42728	-1.356711441	5.910295482	0.008987059	FBgn0264255
GB55897	-1.358316483	5.084324248	0.036487496	FBgn0034075
GB52253	-1.359821143	8.76961996	0.012953834	FBgn0261710
GB47320	-1.360944577	5.104689249	0.02186642	FBgn0039020
GB40363	-1.362846942	5.993923835	0.008156179	FBgn0086698
GB55485	-1.364626731	5.766148974	0.006560406	FBgn0026573
GB46618	-1.365784593	8.915481127	0.012274627	FBgn0002528
GB53574	-1.367587642	6.255567777	0.003490829	FBgn0052600
GB44941	-1.368324614	7.387715039	0.010151248	FBgn0020443
GB55162	-1.370844386	7.068134334	0.011036644	FBgn0030870
GB43884	-1.37147972	4.966512374	0.045630493	FBgn0031252
GB48946	-1.372647292	7.497078529	0.011245664	FBgn0038725
GB51332	-1.376366666	5.168577477	0.022648112	FBgn0028487
GB50871	-1.376533442	6.283121942	0.004084849	FBgn0025625
GB51962	-1.376550655	8.777155331	0.011858291	FBgn0086902
GB42489	-1.381915286	6.744660081	0.01378901	FBgn0016081
GB43338	-1.382407456	5.576176403	0.009531735	FBgn0020251
GB48385	-1.383176737	7.823826744	0.010107707	FBgn0040271
GB45145	-1.385306364	7.002221288	0.007341842	FBgn0250785
GB48573	-1.387015048	7.371025817	0.01583855	FBgn0032026
GB42428	-1.397214873	5.592127074	0.008519719	FBgn0038407
GB53782	-1.40114847	6.471801004	0.002861493	FBgn0261564
GB49308	-1.40125894	6.25201007	0.010952441	FBgn0085638
GB52424	-1.404730535	6.404913896	0.002713233	FBgn0023023
GB52213	-1.405999509	5.45231898	0.012024083	FBgn0035798
GB54699	-1.408833638	6.74329744	0.007705866	FBgn0038676
GB55375	-1.413265856	7.60914946	0.007845807	FBgn0083956
GB54806	-1.414809124	6.410068219	0.006271116	FBgn0051100
GB41143	-1.417487681	7.839616295	0.007340172	FBgn0037001
GB52722	-1.419726126	5.42120563	0.009134209	FBgn0030946
GB54851	-1.421695951	5.732646281	0.0044157	FBgn0037000
GB55568	-1.422408197	6.961725315	0.009484375	FBgn0038742
GB42899	-1.424726821	5.641434207	0.006647866	FBgn0085224
GB44799	-1.42536315	5.830294115	0.006591691	FBgn0030060
GB52469	-1.427035253	6.953010084	0.005220168	FBgn0029095
GB45355	-1.427988399	5.425643051	0.008912148	FBgn0038953
GB51558	-1.428042601	5.471486817	0.012568344	FBgn0050263
GB43978	-1.430253812	7.212009581	0.006443952	FBgn0036483
GB55549	-1.432945511	5.016346871	0.030334955	FBgn0039617

GB43223	-1.434703206	5.265517524	0.014588233	FBgn0040228
GB47299	-1.437201202	6.518050656	0.003563804	FBgn0039084
GB51335	-1.437252835	7.16471906	0.005170603	FBgn0036762
GB44030	-1.437372972	6.638476906	0.004869088	FBgn0031384
GB52848	-1.438205026	7.521734795	0.005641825	FBgn0030266
GB43684	-1.438250141	5.429266483	0.00884554	FBgn0030778
GB47321	-1.439792569	5.728792195	0.007196919	FBgn0039111
GB49732	-1.445599224	5.651633447	0.007962712	FBgn0262562
GB40077	-1.445978217	8.211436248	0.009139859	FBgn0027601
GB55982	-1.446502993	5.861217964	0.004504339	FBgn0035146
GB44899	-1.447061449	7.462453896	0.005304666	FBgn0261885
GB43617	-1.458337073	6.440171808	0.001935954	FBgn0038467
GB46429	-1.459757343	7.54368401	0.005471971	FBgn0000527
GB48658	-1.462826886	8.255941427	0.005608549	FBgn0002306
GB48488	-1.466161432	4.911888523	0.02540553	FBgn0017429
GB49380	-1.466464288	6.002527547	0.002476793	FBgn0034145
GB47043	-1.46787234	7.023935562	0.004765012	FBgn0261439
GB52266	-1.476336015	6.637357542	0.004387618	FBgn0004598
GB45148	-1.478582415	5.446774264	0.008019524	FBgn0032783
GB43459	-1.479152106	6.859271472	0.004911705	FBgn0003862
GB53010	-1.484022618	7.793279439	0.006129654	FBgn0037579
GB51791	-1.488961699	6.069319139	0.00112874	FBgn0038108
GB45905	-1.491827425	6.715382419	0.00481365	FBgn0027287
GB43935	-1.493242483	7.597567081	0.014383808	FBgn0032685
GB50627	-1.495455362	7.31627435	0.004074571	FBgn0039620
GB44998	-1.496370476	5.477077346	0.005229046	FBgn0037707
GB55532	-1.496480936	4.771880785	0.033338936	FBgn0037051
GB48981	-1.497051238	6.937951028	0.004083542	FBgn0034517
GB50455	-1.498289763	7.342211503	0.004919102	FBgn0011217
GB41250	-1.501804481	6.449871803	0.001185691	FBgn0036624
GB43179	-1.504608336	7.369381487	0.003662537	FBgn0027866
GB55837	-1.509197577	7.873245418	0.003623868	FBgn0032940
GB51759	-1.509675644	7.416382189	0.003719881	FBgn0032601
GB46543	-1.514717415	5.717818433	0.002845354	FBgn0051216
GB48065	-1.51867425	5.766050406	0.00252838	FBgn0250833
GB54551	-1.520850657	5.804920275	0.001823125	FBgn0259677
GB42482	-1.525659877	6.463668869	0.003792644	FBgn0028670
GB42374	-1.526458619	6.982310723	0.002459476	FBgn0023458
GB50257	-1.529510399	5.921754009	0.001297043	FBgn0002577
GB50179	-1.530585974	5.3760793	0.010334081	FBgn0005558
GB49967	-1.532650059	5.398637468	0.007913708	FBgn0029155
GB44831	-1.534746608	4.810243654	0.025109919	FBgn0038632
GB44893	-1.538174139	4.970473581	0.016477455	FBgn0033582
GB55388	-1.538300084	7.106344678	0.002602132	FBgn0033763
GB47305	-1.542489926	5.695388894	0.001993687	FBgn0003742
GB50681	-1.544661095	5.51841992	0.003470675	FBgn0037855
GB45632	-1.545766042	5.164450714	0.012641556	FBgn0033749
GB44368	-1.54966328	5.860149251	0.001459929	FBgn0052699
GB51481	-1.552430066	6.886386934	0.002373109	FBgn0031464
GB44134	-1.557095613	8.94740666	0.008332095	FBgn0003888
GB46282	-1.55775342	6.394612301	0.000913961	FBgn0027587
GB45372	-1.559334505	6.009168896	0.000980113	FBgn0031821
GB50677	-1.561380741	6.521445834	0.003229088	FBgn0035085
GB43693	-1.564156677	5.802218365	0.001468575	FBgn0036910
GB44570	-1.568279812	8.654331652	0.004388006	FBgn0037447
GB43228	-1.570402464	6.066143864	0.00044164	FBgn0035372

GB41850	-1.573040255	5.886302187	0.001291197	FBgn0029167
GB48355	-1.573234145	7.370430859	0.001721586	FBgn0031857
GB41989	-1.573390436	5.797022365	0.002622077	FBgn0033661
GB49328	-1.573431951	6.946149191	0.0018063	FBgn0033464
GB41773	-1.575937093	9.820961935	0.003289094	FBgn0024989
GB55640	-1.576088607	4.927961949	0.011095962	FBgn0034500
GB49845	-1.576647145	8.959146589	0.002218882	FBgn0001311
GB43230	-1.582890462	5.643228474	0.001870292	FBgn0001253
GB41908	-1.582910529	6.98898126	0.001688629	FBgn0039936
GB52997	-1.585026847	7.628021348	0.001989616	FBgn0262110
GB43946	-1.591024276	5.273820994	0.00560765	FBgn0036483
GB46338	-1.593303257	5.009111543	0.012100079	FBgn0043550
GB55298	-1.596894338	5.542591537	0.002583969	FBgn0011227
GB55481	-1.600874272	6.055058845	0.000542938	FBgn0001991
GB41659	-1.605056253	5.454523686	0.004219619	FBgn0031081
GB44253	-1.608824943	5.910778015	0.001037233	FBgn0029881
GB49562	-1.610289784	5.340796068	0.007340172	FBgn0001186
GB49282	-1.613159497	4.935703385	0.014333012	FBgn0025624
GB54692	-1.613161022	6.243686557	0.000482801	FBgn0037622
GB50847	-1.613788582	6.411737417	0.000543771	FBgn0030093
GB45970	-1.616233999	5.097091194	0.010714979	FBgn0015609
GB43366	-1.629143426	4.873967175	0.015747903	FBgn0051663
GB47905	-1.635874455	6.00895751	0.000457125	FBgn0033205
GB46626	-1.636890805	5.615132809	0.003258694	FBgn0035199
GB44811	-1.636995039	5.246490849	0.005592885	FBgn0261555
GB48578	-1.643806537	4.907908958	0.010066785	FBgn0011297
GB54150	-1.646219975	6.089724402	0.000381498	FBgn0039061
GB53578	-1.649999835	4.815302994	0.016540525	FBgn0051414
GB45212	-1.65933401	6.570387762	0.000328698	FBgn0039187
GB48254	-1.662186716	7.794124195	0.00155009	FBgn0003174
GB52740	-1.667107567	5.060358833	0.007969715	FBgn0039224
GB52768	-1.668374401	5.799479922	0.000692963	FBgn0032779
GB50558	-1.674171856	6.170840971	0.000324707	FBgn0036135
GB46448	-1.675445719	5.756480945	0.001182561	FBgn0037203
GB50655	-1.677750533	4.551749544	0.021636336	FBgn0034364
GB41701	-1.678795021	6.856045931	0.000613173	FBgn0050122
GB45153	-1.679887072	7.949860667	0.001046418	FBgn0031771
GB49533	-1.680756303	6.486327636	0.000235333	FBgn0033109
GB51422	-1.681337655	5.716344828	0.001178378	FBgn0035799
GB41366	-1.683094434	6.780122962	0.001182561	FBgn0053196
GB55480	-1.683411075	5.215716361	0.005945144	FBgn0001991
GB43584	-1.685178481	4.550313607	0.024492936	FBgn0036433
GB43611	-1.68984253	6.86093741	0.000639953	FBgn0263705
GB40305	-1.690517245	7.829225721	0.00139163	FBgn0039909
GB43890	-1.690666134	6.40494113	0.001106408	FBgn0033431
GB41680	-1.693868841	7.538595326	0.000662249	FBgn0034975
GB47199	-1.695935405	6.193047004	0.000775532	FBgn0010222
GB42840	-1.696627269	7.615187962	0.000819164	FBgn0032305
GB44868	-1.696935476	5.266186847	0.00567286	FBgn0250862
GB45540	-1.697728804	6.564259474	0.000456782	FBgn0000097
GB40703	-1.697981042	8.312923646	0.000882379	FBgn0036715
GB55744	-1.698554326	4.853713494	0.01141311	FBgn0035260
GB43214	-1.699108369	5.294543729	0.004165578	FBgn0013811
GB50954	-1.69975637	5.157621896	0.005066367	FBgn0053988
GB48362	-1.701689148	5.221609729	0.005304666	FBgn0000489
GB41609	-1.701929554	6.154848836	0.000352919	FBgn0030631

GB46431	-1.701996264	7.466872912	0.000692386	FBgn0037249
GB54693	-1.710331323	6.883763616	0.000545486	FBgn0038924
GB54685	-1.710742073	5.243160247	0.002448367	FBgn0039356
GB42557	-1.711029539	6.25895522	0.000275746	FBgn0039203
GB55911	-1.718962886	6.7728769	0.000866506	FBgn0026059
GB47779	-1.719008215	5.602353074	0.000925908	FBgn0032797
GB56009	-1.72075281	5.595587466	0.000773245	FBgn0000182
GB44912	-1.725029486	5.115081819	0.004913706	FBgn0002441
GB49325	-1.725079898	6.069244233	0.000245268	FBgn0011604
GB43143	-1.725823736	6.137362037	0.000200251	FBgn0033495
GB45265	-1.726785506	6.984567288	0.001253475	FBgn0033484
GB50586	-1.727760627	7.093594418	0.00050546	FBgn0003319
GB50683	-1.728949236	8.038790092	0.000586843	FBgn0037855
GB53670	-1.729262213	7.215149763	0.000958404	FBgn0034087
GB53716	-1.731838027	5.957009581	0.000310234	FBgn0033887
GB42652	-1.733858067	8.360669288	0.003297569	FBgn0034603
GB55808	-1.737203295	7.863294857	0.00056252	FBgn0020306
GB42015	-1.737569039	6.169895059	0.000304616	FBgn0037838
GB52979	-1.739805394	5.618867556	0.000508522	FBgn0037757
GB46157	-1.748066396	4.370584254	0.030753457	FBgn0259167
GB51768	-1.756787289	7.138737538	0.000546836	FBgn0036333
GB48162	-1.759323003	5.584285623	0.00055173	FBgn0028879
GB49764	-1.759970667	6.738671201	0.000547902	FBgn0261277
GB52252	-1.762415907	7.755704951	0.000448386	FBgn0031107
GB44685	-1.7718009	7.204557118	0.000399479	FBgn0038532
GB50245	-1.772034699	6.443158495	0.000530088	FBgn0263109
GB40615	-1.774730034	5.29869011	0.001605539	FBgn0039178
GB55425	-1.777929161	5.078421259	0.003835155	FBgn0013984
GB55356	-1.779187535	6.926704729	0.000289206	FBgn0029835
GB43456	-1.78034967	6.265156054	0.000267501	FBgn0034476
GB53657	-1.782108033	6.814626799	0.000245268	FBgn0037093
GB54397	-1.785428479	5.00025246	0.005016816	FBgn0038986
GB49977	-1.788529061	7.344054144	0.000328698	FBgn0039709
GB42484	-1.788941502	7.150225215	0.000330188	FBgn0039640
GB43304	-1.792236133	7.033678736	0.000262866	FBgn0037963
GB48948	-1.793415005	6.374378139	0.000145343	FBgn0032649
GB42052	-1.795674235	4.631935187	0.01365845	FBgn0262112
GB43114	-1.799933131	5.718398983	0.000233123	FBgn0030508
GB43123	-1.801909847	9.638728478	0.000480347	FBgn0263132
GB55495	-1.803351599	5.247369341	0.002603591	FBgn0053653
GB41849	-1.809167051	4.869278802	0.004138696	FBgn0054056
GB54996	-1.810711366	5.438969828	0.001046418	FBgn0040849
GB46902	-1.811604489	6.073549098	9.01187E-05	FBgn0035146
GB50101	-1.812149096	7.322015625	0.000254117	FBgn0039594
GB41290	-1.814352865	6.727352358	0.000245268	FBgn0051224
GB42793	-1.816314492	4.293982815	0.030127865	FBgn0038294
GB44254	-1.816868908	7.59974148	0.00033882	FBgn0029881
GB50061	-1.818420171	7.480552425	0.000286816	FBgn0001321
GB45972	-1.81852511	6.830636257	0.00136475	FBgn0015609
GB40344	-1.819495587	4.665795644	0.009981156	FBgn0039655
GB47274	-1.822832473	5.578657087	0.000376795	FBgn0027556
GB47082	-1.822866754	4.689665037	0.004946799	FBgn0030660
GB55843	-1.826270481	6.080908557	8.01085E-05	FBgn0035253
GB55274	-1.832369157	7.049445609	0.000176844	FBgn0026374
GB41900	-1.839724844	6.231425685	7.07022E-05	FBgn0259110
GB47451	-1.843730071	5.603172502	0.000261925	FBgn0033633

GB45152	-1.846087486	11.15578654	0.000280744	FBgn0261341
GB51104	-1.847201908	9.200854437	0.00027463	FBgn0028371
GB45852	-1.847993658	6.099417061	4.33761E-05	FBgn0050021
GB50769	-1.848982917	6.265326287	5.11189E-05	FBgn0000008
GB45551	-1.865392373	4.58752685	0.010078381	FBgn0259923
GB40405	-1.871170609	6.963984187	0.000130374	FBgn0003159
GB51774	-1.872314832	4.341144282	0.020469687	FBgn0039536
GB52759	-1.88880347	5.386452165	0.00071788	FBgn0029834
GB51068	-1.889578892	5.314139586	0.00121524	FBgn0000121
GB49616	-1.893444106	4.592893539	0.013542361	FBgn0263132
GB45111	-1.894653149	5.747040217	7.76241E-05	FBgn0010772
GB43613	-1.903666381	6.876165231	0.000101899	FBgn0263705
GB49262	-1.905337046	8.109968957	0.000108666	FBgn0262526
GB49095	-1.916347428	6.649063506	0.00010743	FBgn0062413
GB44187	-1.928145804	5.42372913	0.000280744	FBgn0028387
GB54362	-1.934287969	5.772770005	5.60679E-05	FBgn0033633
GB48497	-1.938355015	7.973610186	0.00013272	FBgn0010470
GB46165	-1.938945193	5.339008636	0.001204284	FBgn0010389
GB46680	-1.939058195	5.668059993	8.07642E-05	FBgn0031849
GB44426	-1.939646145	6.161894911	4.04826E-05	FBgn0037537
GB49283	-1.948060348	6.397169321	0.000445327	FBgn0036398
GB48577	-1.949347532	6.686311511	8.56171E-05	FBgn0001083
GB43953	-1.950016058	6.471806348	1.87982E-05	FBgn0023094
GB51338	-1.954304187	4.267178431	0.017306479	FBgn0262110
GB41746	-1.955338532	6.351120898	1.827E-05	FBgn0011592
GB48454	-1.956593242	8.174013948	7.7554E-05	FBgn0000108
GB45681	-1.964745429	6.843430635	0.000130374	FBgn0010470
GB52097	-1.966638059	5.150922886	0.001515217	FBgn0058006
GB47678	-1.967429559	5.745832466	0.000104801	FBgn0035179
GB55395	-1.975832677	5.589164134	9.06634E-05	FBgn0029837
GB42447	-1.977747233	4.674058735	0.00560765	FBgn0024998
GB47949	-1.980380097	6.26845247	1.24049E-05	FBgn0013988
GB40808	-1.984366235	7.722928131	5.56797E-05	FBgn0029688
GB49730	-1.987540396	4.689947536	0.004989102	FBgn0262562
GB53933	-1.990205122	6.635786941	5.99861E-05	FBgn0038799
GB52910	-1.991662135	4.987628188	0.002710777	FBgn0024944
GB45932	-1.992244003	5.258533534	0.000410877	FBgn0020440
GB52937	-2.00121177	5.176790706	0.001577538	FBgn0000287
GB55275	-2.00550904	7.602960354	5.11189E-05	FBgn0036289
GB51814	-2.007318168	5.258986643	0.000937743	FBgn0001112
GB50003	-2.00945882	5.254912371	0.001191877	FBgn0260964
GB47845	-2.012554638	5.191634162	0.000561827	FBgn0037212
GB43054	-2.017111428	7.490179027	6.469E-05	FBgn0261259
GB52194	-2.018421065	5.09532258	0.001136596	FBgn0016075
GB44661	-2.02436432	6.792116003	4.04826E-05	FBgn0259173
GB40010	-2.02974635	8.739768387	6.86688E-05	FBgn0261836
GB42676	-2.035154667	5.03979218	0.001194919	FBgn0033769
GB55328	-2.036646548	8.155736417	6.469E-05	FBgn0003721
GB40445	-2.041023143	9.649677948	4.17707E-05	FBgn0031918
GB41625	-2.047772287	8.738462402	4.18903E-05	FBgn0035844
GB45484	-2.050106095	6.32301766	1.35264E-05	FBgn0037447
GB56032	-2.056953903	4.34681506	0.009981156	FBgn0034013
GB52014	-2.058536762	5.383709453	0.000295092	FBgn0000299
GB46268	-2.06132706	8.481130058	3.30966E-05	FBgn0024183
GB43084	-2.062450179	6.66493838	4.1552E-05	FBgn0035238
GB43137	-2.06314468	5.154324951	0.000493239	FBgn0261873

GB49930	-2.066238868	6.663242543	2.49775E-05	FBgn0028491
GB50953	-2.066589113	5.461614851	0.000123531	FBgn0053988
GB54144	-2.069489405	6.078120028	0.000462688	FBgn0259233
GB55615	-2.071922208	7.847714442	8.19374E-05	FBgn0034860
GB53012	-2.073314241	5.835883639	1.50624E-05	FBgn0010473
GB48813	-2.079086613	6.845869219	0.001998484	FBgn0066365
GB41372	-2.079431145	5.996318533	5.20443E-06	FBgn0038641
GB47349	-2.082447124	6.075243597	5.08598E-06	FBgn0036298
GB43586	-2.087778622	4.515947479	0.004757278	FBgn0034942
GB50690	-2.093298984	7.211588512	2.39852E-05	FBgn0261397
GB52925	-2.095079053	6.285389589	5.98745E-06	FBgn0086911
GB46297	-2.095658826	10.88557352	6.08407E-05	FBgn0035736
GB52955	-2.099735175	6.652699487	5.91783E-05	FBgn0051559
GB54893	-2.100307136	6.145692218	2.65826E-06	FBgn0031251
GB50753	-2.106002637	6.182359294	2.55746E-06	FBgn0028474
GB43552	-2.106654816	6.845249277	3.85966E-05	FBgn0035575
GB47938	-2.111739877	8.991316647	1.79932E-05	FBgn0031879
GB54347	-2.112634183	6.81949494	7.05154E-05	FBgn0259734
GB40993	-2.11437622	5.720206926	8.28101E-05	FBgn0038247
GB40203	-2.116183164	7.415689167	1.7022E-05	FBgn0051028
GB42904	-2.120023241	6.491512321	8.02995E-06	FBgn0262681
GB55895	-2.13193111	4.734181793	0.000561827	FBgn0040705
GB46221	-2.135137446	4.677159315	0.002567827	FBgn0259167
GB50507	-2.13724668	4.407792504	0.006619421	FBgn0261514
GB51511	-2.140277376	6.089645958	2.01795E-06	FBgn0261261
GB46145	-2.141146371	4.412323488	0.007568298	FBgn0032946
GB42653	-2.143456457	7.420499792	9.73307E-06	FBgn0011288
GB55241	-2.149547345	6.327513745	2.31614E-06	FBgn0030685
GB40694	-2.1496155	4.256526106	0.008332095	FBgn0037153
GB50672	-2.161644388	7.626753766	2.89004E-05	FBgn0086736
GB50891	-2.166604974	6.520065814	2.32477E-06	FBgn0034716
GB46663	-2.170247952	4.101046071	0.011854996	FBgn0032506
GB54538	-2.170967828	6.359411674	5.68892E-06	FBgn0262870
GB46274	-2.172398924	7.369278126	4.66435E-05	FBgn0030648
GB53116	-2.174127406	5.038185976	0.002602132	FBgn0086911
GB42130	-2.176000884	4.600941219	0.002759017	FBgn0028371
GB54416	-2.176466107	4.958692479	0.000612891	FBgn0030613
GB43602	-2.180858949	5.306047884	0.000278752	FBgn0052082
GB54868	-2.182396527	4.733764146	0.001486986	FBgn0031426
GB45115	-2.186968958	4.943058391	0.000933504	FBgn0050468
GB42350	-2.197090557	5.166313997	0.000217148	FBgn0029807
GB45218	-2.200131594	5.238996109	0.000250903	FBgn0035056
GB41731	-2.201588781	5.17122647	0.000249244	FBgn0262636
GB51063	-2.205060106	8.654923254	4.19499E-05	FBgn0051973
GB46015	-2.210543373	5.182687198	0.000168292	FBgn0033753
GB40106	-2.212639966	5.447241774	2.89502E-05	FBgn0264672
GB43612	-2.230123011	6.153245882	6.041E-07	FBgn0263705
GB55545	-2.231799506	5.088686382	0.000612891	FBgn0031860
GB40671	-2.234766172	5.465346385	1.96409E-05	FBgn0011592
GB50085	-2.23726162	4.163974657	0.006616969	FBgn0052736
GB40263	-2.239323281	7.218582838	4.39072E-06	0
GB46164	-2.242640553	4.474802742	0.005703211	FBgn0010389
GB40810	-2.243092865	4.801513323	0.000422028	FBgn0035805
GB49870	-2.250118287	6.789831919	4.12352E-06	FBgn0036821
GB48576	-2.258255905	6.793045747	1.2235E-05	FBgn0031879
GB53651	-2.259520049	4.922550459	0.000365369	FBgn0011676

GB49767	-2.262595692	4.344848219	0.006409526	FBgn0263219
GB41863	-2.264833895	5.4035996	3.97299E-05	FBgn0029830
GB50295	-2.272532851	6.654831993	2.13391E-06	FBgn0031879
GB50207	-2.273646693	3.964789607	0.014383808	FBgn0036542
GB55483	-2.276427974	8.749898958	1.92736E-05	FBgn0005666
GB41203	-2.287525653	10.56775492	1.8716E-05	FBgn0026077
GB43941	-2.297025462	7.035096273	1.95633E-06	FBgn0031195
GB50538	-2.298544107	5.168727036	9.50579E-05	FBgn0263986
GB54643	-2.299557762	7.695623788	5.99602E-06	FBgn0019960
GB55781	-2.299762327	8.664586283	5.24693E-06	FBgn0053196
GB42866	-2.303683509	4.696166499	0.00134622	FBgn0259246
GB43543	-2.304189831	4.801124129	0.001210738	FBgn0024963
GB42796	-2.310787373	5.939130698	4.70241E-06	FBgn0250839
GB48837	-2.312959031	8.553508385	5.68892E-06	FBgn0039008
GB46886	-2.314202596	5.182144424	8.75767E-05	FBgn0010399
GB49534	-2.316491814	9.609727552	2.93403E-06	FBgn0027341
GB49106	-2.31720257	8.767852443	2.63884E-06	FBgn0250789
GB49543	-2.33064748	4.576428615	0.002857007	FBgn0036381
GB41270	-2.33123855	9.580151452	5.62231E-06	FBgn0026077
GB42976	-2.337849669	5.887214947	9.5281E-07	FBgn0004449
GB43163	-2.343741916	6.511168113	1.46767E-06	FBgn0027503
GB48062	-2.351353277	5.476878462	9.69932E-06	FBgn0033702
GB44209	-2.359233013	7.754747995	7.87753E-06	FBgn0037537
GB51722	-2.359486556	7.137604465	3.33905E-06	FBgn0035936
GB47974	-2.371956632	6.061179065	1.24084E-05	FBgn0015575
GB45140	-2.373238941	5.127269971	7.80932E-05	FBgn0032129
GB44315	-2.375423476	4.275921013	0.003320369	FBgn0033095
GB42644	-2.376208112	4.052005571	0.008882024	FBgn0000039
GB44949	-2.38098485	4.626421768	0.000768331	FBgn0037368
GB54213	-2.382004933	7.230130452	3.41534E-06	FBgn0052000
GB51090	-2.38226148	4.454332747	0.002004696	FBgn0035917
GB40231	-2.392324407	4.275435366	0.003650816	FBgn0259244
GB49726	-2.395408482	6.243943698	2.40081E-07	FBgn0003137
GB48727	-2.400814278	3.822892409	0.015326511	FBgn0000560
GB47595	-2.406632745	3.8209395	0.014261774	FBgn0033654
GB50564	-2.409185095	6.492339343	0.000512798	FBgn0027527
GB50091	-2.410323288	5.0402493	9.6708E-05	FBgn0003499
GB41855	-2.415670353	6.962327291	1.01918E-06	FBgn0262867
GB41888	-2.420408994	6.708134759	7.91433E-07	FBgn0036398
GB54127	-2.420978928	4.800635021	0.000407462	FBgn0003353
GB47496	-2.422445342	4.501668786	0.0008941	FBgn0039667
GB55556	-2.423948662	3.835242193	0.013724739	FBgn0038921
GB49184	-2.427798537	5.45175979	8.71009E-06	FBgn0032749
GB42378	-2.438811028	6.175697042	3.01057E-06	FBgn0261799
GB46701	-2.439367284	6.806500636	7.30809E-07	FBgn0051204
GB54390	-2.442173205	6.730119825	6.79087E-07	FBgn0025393
GB40074	-2.443895641	4.117068322	0.005319708	FBgn0014859
GB41670	-2.449160209	6.636483565	1.13613E-06	FBgn0010497
GB54483	-2.455635423	7.178253526	7.43515E-07	FBgn0035539
GB42411	-2.467320228	4.144154605	0.003627822	FBgn0034636
GB45937	-2.475314827	8.037460993	4.814E-07	FBgn0013765
GB41861	-2.478265786	6.431110093	1.32551E-07	FBgn0053196
GB42884	-2.482837922	4.700907011	0.000382292	FBgn0051146
GB46699	-2.48810559	5.126330017	0.000919202	FBgn0263077
GB47513	-2.493871181	5.333031228	8.19462E-06	FBgn0000448
GB50567	-2.495591466	6.304960535	1.64976E-06	FBgn0037416

GB52447	-2.513341232	5.117873254	4.33761E-05	FBgn0036780
GB42804	-2.515105363	8.134492831	2.55397E-07	FBgn0033192
GB50228	-2.520844625	4.394078858	0.001538639	FBgn0039048
GB42239	-2.525504795	7.285804815	4.07168E-07	FBgn0034943
GB40393	-2.527549074	6.060629954	4.11362E-05	FBgn0032211
GB52446	-2.531282887	6.556572881	9.86775E-07	FBgn0020300
GB54926	-2.539439336	3.639640812	0.016843765	FBgn0036369
GB42612	-2.545812735	8.913669464	4.80354E-07	FBgn0033603
GB44939	-2.550219791	3.954498748	0.006875384	FBgn0026439
GB43198	-2.553961921	8.213643431	9.53177E-07	FBgn0000667
GB43231	-2.555956898	7.21338108	1.21689E-06	FBgn0035888
GB46077	-2.55599842	4.926770736	7.74226E-05	FBgn0030729
GB46368	-2.560571109	5.282438473	1.14899E-05	FBgn0011693
GB54076	-2.563901244	6.575838749	1.696E-06	FBgn0032785
GB41862	-2.564779429	5.373726542	1.05445E-05	FBgn0259680
GB41662	-2.565617167	3.669773555	0.014155874	FBgn0027548
GB46188	-2.577972002	4.7914217	0.000208313	FBgn0085425
GB43812	-2.578530085	3.677458268	0.012314163	FBgn0035192
GB55559	-2.579587104	5.194076402	2.32394E-05	FBgn0033679
GB42823	-2.587271404	5.808906127	3.2383E-07	FBgn0036180
GB41260	-2.60628321	5.309155936	6.83392E-06	FBgn0259247
GB50150	-2.610315604	3.699967163	0.012746549	FBgn0033958
GB49104	-2.617253247	4.231217066	0.003090902	FBgn0000567
GB43205	-2.618146071	5.753631578	5.60513E-07	FBgn0032598
GB42487	-2.620286575	7.274878873	8.59987E-08	FBgn0260450
GB54313	-2.620710311	6.54186644	3.40745E-08	FBgn0030884
GB46151	-2.622119247	5.19853514	3.44763E-05	FBgn0082585
GB44842	-2.6272153	6.847246177	6.0668E-08	FBgn0082582
GB53064	-2.628168553	6.689134878	1.69752E-06	FBgn0010482
GB40969	-2.629764001	4.259899943	0.00161421	FBgn0035711
GB51442	-2.63410931	6.812412596	2.12724E-06	FBgn0052036
GB54743	-2.638255677	5.939595771	2.09661E-08	FBgn0004959
GB55912	-2.640877909	7.870578396	5.44574E-08	FBgn0026059
GB47869	-2.64627801	4.298180072	0.001012482	FBgn0037448
GB55359	-2.646318875	7.67997007	1.54263E-07	FBgn0036365
GB41684	-2.653632921	4.866929648	5.72488E-05	FBgn0031730
GB50262	-2.654074489	5.137562131	1.02544E-05	FBgn0034911
GB50453	-2.666775497	6.099327975	1.21104E-08	FBgn0039805
GB46113	-2.672277662	5.670078574	1.15053E-07	FBgn0053143
GB44548	-2.680605707	5.715926808	8.40508E-07	FBgn0001112
GB49809	-2.685032361	5.160591328	9.32217E-06	FBgn0040351
GB45235	-2.695929701	5.16790519	1.53027E-05	FBgn0052432
GB46817	-2.707420772	9.562987392	4.75014E-06	FBgn0053257
GB53675	-2.709033224	4.773596928	2.49689E-05	FBgn0031999
GB53970	-2.713222161	8.369231916	4.10233E-08	FBgn0035574
GB47004	-2.717709681	4.913209433	6.57372E-05	FBgn0031849
GB49785	-2.720799553	4.75355083	9.74019E-05	FBgn0026438
GB42296	-2.729945999	5.434107509	1.41941E-06	FBgn0038511
GB50866	-2.732768107	7.834901445	1.82702E-08	FBgn0010435
GB46597	-2.734725565	5.726054653	3.13702E-08	FBgn0013953
GB51744	-2.742406599	3.822851732	0.006741597	FBgn0032338
GB54136	-2.7575608	5.196629564	9.49943E-06	FBgn0264272
GB46302	-2.760994474	4.603189428	0.000202064	FBgn0262738
GB44316	-2.761589269	5.100589726	1.03083E-05	FBgn0033095
GB47971	-2.769448048	5.986519413	1.47678E-08	FBgn0033917
GB55939	-2.773370514	3.470610601	0.012568344	FBgn0040373

GB45919	-2.774046973	4.957221174	2.50472E-05	FBgn0259164
GB55765	-2.776931202	6.557191525	7.92015E-08	FBgn0000442
GB50013	-2.790943119	5.094405823	1.56386E-05	FBgn0036891
GB45841	-2.793141212	3.864969012	0.005163309	FBgn0263239
GB44560	-2.795977474	5.780662228	1.47678E-08	FBgn0003149
GB40412	-2.804949919	4.170956112	0.001182561	FBgn0051475
GB42493	-2.807723943	7.166267125	4.59623E-08	FBgn0028573
GB44223	-2.808192867	7.402595059	2.31428E-07	FBgn0027611
GB46062	-2.809663868	3.881287253	0.003702999	FBgn0033524
GB52077	-2.812800381	4.442787299	0.000246699	FBgn0003068
GB43932	-2.817436602	3.499336479	0.014057918	FBgn0032506
GB45553	-2.837134323	5.179651875	1.03111E-06	FBgn0034157
GB48234	-2.846264172	4.680203126	7.28379E-05	FBgn0000464
GB40503	-2.859832692	8.44600288	7.45758E-09	FBgn0032350
GB45211	-2.865658278	8.461314388	5.85411E-08	FBgn0010423
GB43718	-2.868062535	5.835016914	5.53923E-09	FBgn0002938
GB42798	-2.872162524	5.95580609	1.8716E-05	FBgn0037288
GB40306	-2.873348311	5.915917049	3.24324E-09	FBgn0010435
GB44962	-2.879152908	3.929239916	0.002602132	FBgn0042696
GB44453	-2.881508853	5.550213499	1.16848E-07	FBgn0032804
GB44961	-2.88801489	3.557102777	0.011439352	FBgn0042696
GB53343	-2.889573359	6.703322685	1.19113E-08	FBgn0043903
GB50590	-2.891393358	5.565245862	2.88026E-05	FBgn0260228
GB53894	-2.894724435	3.963418414	0.001596367	FBgn0028474
GB47972	-2.901725477	3.561823423	0.01078842	FBgn0033917
GB42660	-2.902883517	4.261197648	0.000641346	FBgn0028878
GB41331	-2.910129686	3.980535749	0.001194919	0
GB51736	-2.911995172	8.402392192	7.71033E-09	FBgn0031957
GB41196	-2.912559189	7.155313032	5.14598E-09	FBgn0023518
GB50007	-2.915180987	4.253090354	0.001512742	FBgn0039858
GB42942	-2.928761842	3.589569467	0.011095962	FBgn0040297
GB52078	-2.929370617	5.255319968	4.79876E-07	FBgn0003068
GB53331	-2.938154303	6.712827601	2.44897E-09	FBgn0039294
GB50648	-2.939495996	5.777115272	3.04766E-08	FBgn0003319
GB47512	-2.948860591	5.107519438	4.27993E-06	FBgn0000448
GB47224	-2.95108648	7.719645545	2.98163E-09	FBgn0000568
GB53319	-2.951494067	7.08454234	1.21727E-08	FBgn0034301
GB41262	-2.954017282	5.592180227	1.55172E-07	FBgn0259247
GB44002	-2.955571868	5.994403051	3.7773E-09	FBgn0000299
GB52036	-2.964350953	5.127349798	3.68602E-06	FBgn0003975
GB46410	-2.968389493	3.59743572	0.009240393	FBgn0000212
GB54921	-2.979916838	6.322017708	2.98121E-09	FBgn0037487
GB48858	-2.980154647	7.559894221	1.7364E-08	FBgn0040601
GB55664	-2.983259522	5.858838241	1.18903E-09	FBgn0051374
GB52028	-2.9852983	9.037545723	1.87328E-09	FBgn0000556
GB55423	-2.986842601	4.336457135	0.000240143	FBgn0036662
GB53163	-2.999258688	4.070189531	0.000625287	FBgn0035113
GB40626	-3.000600839	3.636155542	0.006816476	FBgn0004575
GB52002	-3.008949311	5.439212863	2.232E-07	FBgn0031414
GB46976	-3.009486316	5.838427403	1.21458E-05	FBgn0000451
GB53320	-3.019587992	3.645137563	0.005945219	FBgn0013813
GB45149	-3.020063745	6.605860165	3.37997E-08	FBgn0032785
GB47718	-3.023414099	4.606532237	7.13897E-05	FBgn0023091
GB40842	-3.027170988	7.042735856	1.28767E-09	FBgn0001112
GB42895	-3.030277255	4.840840567	1.34339E-05	FBgn0030716
GB45609	-3.032665692	7.294316613	1.79731E-07	FBgn0033079

GB54818	-3.038260958	7.326876217	2.95618E-09	FBgn0014863
GB44798	-3.045568798	7.63468924	6.86218E-10	FBgn0034797
GB55370	-3.046896671	5.485560635	3.25648E-08	FBgn0025878
GB45364	-3.046941837	6.713696004	1.93139E-09	FBgn0261509
GB44647	-3.053941933	6.41634167	1.04587E-10	FBgn0261999
GB47499	-3.060260988	7.973510911	5.10726E-10	FBgn0085384
GB47838	-3.060883014	8.08435274	3.86579E-10	FBgn0034974
GB49946	-3.070478444	5.337620259	3.59238E-07	FBgn0011653
GB45771	-3.0858501	6.103622752	1.54436E-10	0
GB55225	-3.095971529	7.272958432	4.4126E-10	FBgn0001083
GB48709	-3.102188942	6.196294826	9.84566E-11	FBgn0033061
GB44110	-3.119360338	5.413588381	1.29483E-07	FBgn0086673
GB50441	-3.119498522	8.161709031	1.40988E-08	FBgn0038405
GB47977	-3.123774577	10.28533363	7.95798E-10	FBgn0086906
GB40009	-3.12706303	4.719576831	1.77373E-05	FBgn0261836
GB49078	-3.137702477	5.128706767	2.98459E-07	FBgn0052645
GB55216	-3.161541951	5.285369534	3.92224E-07	FBgn0039896
GB40446	-3.164497908	6.645400578	6.74827E-10	FBgn0033869
GB55547	-3.16555119	7.624567439	3.89229E-08	FBgn0050172
GB42062	-3.173992804	8.38583813	3.355E-09	FBgn0039527
GB53345	-3.181444525	8.212626461	5.48915E-10	FBgn0037217
GB50000	-3.181476353	8.868521481	2.61782E-10	FBgn0032453
GB43087	-3.189431735	3.266272753	0.009564805	FBgn0035880
GB55986	-3.201585444	4.7333504	3.25112E-05	FBgn0030884
GB54762	-3.212531047	5.135331752	1.75464E-06	FBgn0033362
GB51355	-3.241408545	5.212683856	9.7527E-08	FBgn0052627
GB54775	-3.241429035	8.655641555	3.95432E-10	FBgn0030027
GB45188	-3.244369679	7.328111521	6.55002E-11	FBgn0031630
GB40609	-3.249393477	8.08824625	1.18551E-09	FBgn0039210
GB43983	-3.259221983	6.513294295	1.22472E-11	FBgn0030617
GB41308	-3.261581469	9.024593034	2.31228E-10	FBgn0000046
GB54417	-3.261931823	4.28860968	7.30111E-05	FBgn0026268
GB41730	-3.262093339	3.882081509	0.001161445	FBgn0262636
GB49273	-3.26739832	3.887526185	0.001049378	FBgn0038837
GB52186	-3.280000296	5.091021008	5.16702E-08	FBgn0052808
GB55546	-3.290437965	4.614257227	1.46644E-05	FBgn0039501
GB53736	-3.293699898	3.351168376	0.005278769	FBgn0086913
GB47990	-3.295593094	7.345253542	3.86579E-10	FBgn0004117
GB55613	-3.298266407	5.526298719	3.08701E-08	FBgn0030161
GB47948	-3.303372991	7.112833257	2.35403E-10	FBgn0013988
GB48007	-3.304029523	8.068474651	2.39652E-10	FBgn0034412
GB44777	-3.307542315	8.410463594	4.73055E-10	FBgn0032299
GB49080	-3.319672502	8.234822824	3.48163E-10	FBgn0052645
GB44832	-3.32169496	6.111077774	6.27074E-11	FBgn0038629
GB44723	-3.342967043	4.629824779	1.96409E-05	FBgn0259240
GB40610	-3.346871447	3.400633098	0.005098975	FBgn0036716
GB42485	-3.355278219	5.480863306	2.20232E-09	FBgn0260450
GB42892	-3.357129016	5.326145635	1.12416E-08	FBgn0004650
GB50660	-3.358081238	6.328591752	4.75547E-12	FBgn0036995
GB43500	-3.35969621	3.977231228	0.000527406	FBgn0033196
GB52025	-3.369708472	8.166835967	3.28091E-11	FBgn0027570
GB49734	-3.376588871	5.075775307	2.28068E-06	FBgn0260386
GB47944	-3.376621343	5.882451779	1.33729E-10	FBgn0035410
GB46975	-3.380730127	6.1511523	1.68211E-06	FBgn0002183
GB54750	-3.401846364	5.156963665	9.54848E-08	FBgn0259226
GB41616	-3.423433276	5.358728891	1.8741E-08	FBgn0031449

GB43892	-3.441720327	7.97802173	1.74363E-11	FBgn0032945
GB55231	-3.444761283	6.959167909	5.76545E-12	FBgn0033602
GB40681	-3.452849119	4.417577522	0.00035031	FBgn0051522
GB43276	-3.461052251	5.960775657	6.52267E-12	FBgn0058470
GB44452	-3.469214775	6.971253865	1.5109E-11	FBgn0032803
GB40996	-3.471097482	5.224161641	6.95305E-08	FBgn0260793
GB49223	-3.472313093	8.083933718	7.83667E-12	FBgn0259108
GB51828	-3.479089194	5.86285037	6.31114E-11	FBgn0036377
GB41945	-3.486713237	7.337082766	6.8411E-11	FBgn0031097
GB48258	-3.487935181	4.087511058	0.000140949	FBgn0038151
GB41723	-3.508829278	8.438320911	3.83109E-12	FBgn0261269
GB42427	-3.515309425	8.342531206	9.98691E-12	FBgn0039640
GB40165	-3.524288341	10.61114485	6.73676E-11	FBgn0003065
GB50236	-3.524514394	6.596171141	2.52686E-07	FBgn0035788
GB45019	-3.549271145	5.833765206	1.79639E-08	FBgn0038894
GB55650	-3.55026924	7.498486834	1.1553E-12	FBgn0011286
GB43465	-3.554733566	6.560950536	1.06227E-12	FBgn0039208
GB42303	-3.564357315	6.208746255	3.49874E-11	FBgn0052816
GB51560	-3.585347755	9.581586872	2.24793E-12	FBgn0034022
GB50041	-3.586787599	3.591192908	0.002159812	FBgn0039749
GB55729	-3.594898287	6.801643981	2.99515E-12	FBgn0041712
GB51051	-3.605699894	4.083894106	0.001435012	FBgn0083978
GB44200	-3.611829434	3.646066184	0.001461311	FBgn0030491
GB51482	-3.612227205	7.392198715	3.1723E-12	FBgn0085446
GB40234	-3.614210599	5.158856937	2.92287E-08	FBgn0037565
GB43643	-3.629612568	5.680497071	6.37075E-10	FBgn0016694
GB47976	-3.633746327	6.314898271	8.58503E-14	FBgn0035308
GB41622	-3.639176694	4.992834688	0.000487882	FBgn0035429
GB55403	-3.67261937	6.92688279	6.96727E-13	FBgn0259994
GB41306	-3.677720438	8.656734182	3.29372E-11	FBgn0000046
GB51089	-3.682510723	4.658406155	1.84272E-06	FBgn0035917
GB54393	-3.682632495	6.857884052	2.24793E-12	FBgn0031646
GB49275	-3.687817518	6.030506029	1.59628E-11	FBgn0038837
GB47788	-3.690337725	3.697438861	0.000820001	FBgn0261588
GB43691	-3.696550957	6.569915354	3.16717E-11	FBgn0038017
GB40008	-3.697204164	7.131445421	4.12557E-13	FBgn0261836
GB55445	-3.706237289	4.682719729	1.42693E-06	FBgn0021767
GB47906	-3.713820006	9.585188602	3.19889E-13	FBgn0033731
GB50171	-3.718417147	7.2436332	5.86962E-13	FBgn0262717
GB41516	-3.729579953	4.668018002	2.02586E-06	FBgn0004865
GB45938	-3.738566975	5.01881578	3.10912E-08	FBgn0000075
GB51901	-3.778849615	6.962300232	7.01313E-13	FBgn0052645
GB54302	-3.780411667	5.610976399	9.42342E-09	FBgn0038986
GB51060	-3.793913475	4.318265439	4.69368E-05	FBgn0035625
GB53579	-3.811421911	5.531300666	2.65795E-09	FBgn0051148
GB46705	-3.823282605	8.669435626	7.46009E-12	FBgn0053519
GB42769	-3.85637941	10.4818936	7.64406E-14	FBgn0038405
GB47037	-3.86421826	7.804414959	6.78622E-13	FBgn0264562
GB41310	-3.879307412	9.603679115	6.02421E-14	FBgn0000046
GB55202	-3.896139926	4.861703186	1.68094E-07	FBgn0041711
GB42851	-3.903595877	4.292643963	0.001182561	FBgn0085472
GB48003	-3.910967283	5.348645302	2.36146E-08	FBgn0039214
GB50570	-3.927145337	6.486204948	1.62753E-10	FBgn0037419
GB48125	-3.936235972	7.037391903	8.3825E-13	FBgn0031869
GB44222	-3.955825696	7.246981119	3.97435E-12	FBgn0039897
GB45853	-3.959210184	4.929079531	2.66783E-08	FBgn0039075

GB47221	-3.959906843	3.022790921	0.008352991	FBgn0052212
GB55617	-3.959916752	5.876954362	5.917E-14	FBgn0029836
GB42594	-3.96597476	6.280442858	1.33742E-14	FBgn0036834
GB46298	-3.982096772	7.257393685	6.83032E-12	FBgn0033728
GB51818	-3.990948484	4.939022905	6.17849E-08	FBgn0030591
GB42206	-3.992052356	6.902002641	3.16339E-12	FBgn0260856
GB46819	-4.001311994	6.502530089	1.969E-13	FBgn0051296
GB54884	-4.005734896	9.039128608	5.6353E-15	FBgn0053978
GB49639	-4.028903205	7.864100196	2.23242E-12	FBgn0039648
GB52723	-4.030291432	6.619578357	1.68466E-13	FBgn0262647
GB53286	-4.045021291	8.204435484	1.27841E-13	FBgn0262111
GB46365	-4.057024912	4.974456709	5.70199E-07	FBgn0011693
GB42599	-4.067659803	4.592873065	7.36671E-06	FBgn0053196
GB53197	-4.104794396	3.206992681	0.00121451	FBgn0039633
GB43052	-4.111074482	9.056160042	8.23506E-14	FBgn0003149
GB55604	-4.127228218	4.660725096	2.86254E-07	FBgn0010316
GB43029	-4.150704452	8.518273872	8.78612E-13	FBgn0000047
GB55158	-4.152293777	6.749309648	9.55066E-14	FBgn0027495
GB53420	-4.183383145	4.180465351	7.45652E-06	FBgn0050044
GB50973	-4.185056482	7.327724912	5.917E-14	FBgn0036044
GB42967	-4.188475931	3.267654717	0.00181652	FBgn0036096
GB47669	-4.202419067	12.1009711	4.95355E-17	FBgn0015766
GB53935	-4.228564203	3.343086793	0.000515938	FBgn0011476
GB41621	-4.228605778	4.235715691	5.13241E-06	FBgn0002440
GB49405	-4.228605778	4.079442027	0.000484898	0
GB52992	-4.23549102	8.019294916	5.76923E-10	FBgn0052354
GB50610	-4.240363463	10.71306543	3.08031E-17	FBgn0050101
GB52666	-4.242892344	5.156182554	9.19826E-10	FBgn0053196
GB42286	-4.246850402	6.254045241	1.13207E-15	FBgn0037122
GB41178	-4.256662856	4.260622205	4.03648E-06	FBgn0038727
GB40085	-4.27637473	4.192402129	3.161E-05	FBgn0029807
GB55598	-4.277513258	8.708018994	5.62437E-14	FBgn0004028
GB54455	-4.290474886	3.293843861	0.002512822	FBgn0052829
GB50170	-4.291997564	8.286038948	1.39962E-16	FBgn0262717
GB48443	-4.301960365	7.483445411	1.34227E-15	FBgn0029922
GB55325	-4.320665639	6.892554585	1.09111E-14	FBgn0003721
GB52824	-4.327509458	5.992688385	7.71415E-13	FBgn0033869
GB46698	-4.343799181	5.501416226	2.07835E-10	FBgn0039862
GB48167	-4.34877614	7.965360417	2.97617E-15	FBgn0262508
GB45732	-4.348780763	8.329065124	9.50218E-17	FBgn0036486
GB41311	-4.35737666	8.724073903	4.02128E-16	FBgn0000045
GB40944	-4.358924532	8.063150791	4.90243E-17	FBgn0034903
GB46038	-4.361571307	5.197874416	1.40988E-08	FBgn0052072
GB50529	-4.367414409	4.217287977	0.000121677	FBgn0037421
GB54970	-4.380997987	8.273651195	2.95582E-15	FBgn0035089
GB43053	-4.381968889	8.866576482	1.39634E-14	FBgn0003149
GB41946	-4.43670816	8.680577925	2.07706E-17	FBgn0022770
GB41495	-4.450448858	5.679412883	1.03428E-13	FBgn0016693
GB42469	-4.460801723	5.32883527	1.30455E-10	FBgn0036939
GB42889	-4.467249783	5.691400816	2.91771E-14	FBgn0039478
GB49646	-4.469094607	7.288566811	1.33937E-16	FBgn0038511
GB42891	-4.469905314	7.714773098	1.14918E-15	FBgn0024366
GB42673	-4.470292705	7.758095292	7.96992E-17	FBgn0032405
GB43580	-4.475025676	10.26650598	2.60627E-18	FBgn0039126
GB50062	-4.505707397	5.694865164	2.08034E-13	FBgn0037665
GB50006	-4.527455125	3.548210442	0.000692386	FBgn0039927

GB45654	-4.549987965	6.200704744	5.66322E-16	FBgn0025837
GB41792	-4.583154146	9.43318071	1.01665E-17	FBgn0040601
GB40623	-4.585361679	4.493376025	1.40963E-06	FBgn0013733
GB41771	-4.5862443	6.194284264	2.54357E-15	FBgn0024897
GB50766	-4.589644777	7.140310981	4.95599E-17	FBgn0264357
GB50450	-4.601842795	10.14761913	2.8631E-13	FBgn0001250
GB42358	-4.60382056	7.155705155	3.82888E-17	FBgn0087011
GB46310	-4.607940378	7.643967304	6.07039E-18	FBgn0000551
GB48216	-4.614427248	6.259634902	2.77797E-17	FBgn0031692
GB40007	-4.619548741	6.591943511	5.25613E-18	FBgn0261836
GB52179	-4.620784914	3.580099331	0.000393494	FBgn0026314
GB55396	-4.627097253	7.722834951	5.82491E-17	FBgn0052694
GB41241	-4.630434324	6.080065548	1.38526E-16	FBgn0033633
GB53288	-4.659290927	4.494329268	7.94652E-06	FBgn0040211
GB51698	-4.673926477	13.27167587	2.7876E-19	FBgn0002564
GB47223	-4.691949181	4.65111896	4.23085E-08	FBgn0000568
GB53516	-4.692738241	8.414489587	1.41516E-17	FBgn0043792
GB49400	-4.711722856	6.515135488	2.48472E-17	FBgn0262599
GB55611	-4.711751657	5.188350976	4.16404E-09	FBgn0010399
GB42852	-4.729615713	4.685471151	4.70181E-08	FBgn0085472
GB54963	-4.741010312	4.588816508	1.06104E-06	FBgn0039335
GB51407	-4.752951863	6.633454006	2.7075E-13	FBgn0050069
GB54133	-4.764054675	3.748066578	9.93925E-05	FBgn0038958
GB42704	-4.78891552	7.865172691	3.78287E-17	FBgn0037290
GB50439	-4.812631542	9.561017666	1.03485E-16	FBgn0039480
GB40607	-4.822213342	5.264951247	5.01474E-08	FBgn0037556
GB51214	-4.824596702	9.540007	1.60611E-16	FBgn0004169
GB40228	-4.843687604	6.034784023	1.18387E-17	0
GB50650	-4.846043513	6.515542172	9.28512E-10	FBgn0033359
GB42901	-4.852877411	6.302846388	1.6544E-06	FBgn0030539
GB50572	-4.867533146	7.192236873	9.74409E-15	FBgn0040279
GB42596	-4.879066174	7.765935055	1.44483E-12	FBgn0021742
GB54456	-4.883901725	5.776622852	1.93286E-16	FBgn0032462
GB53986	-4.894279481	5.381626728	1.18701E-11	FBgn0026268
GB48769	-4.894279481	5.290398982	1.04048E-09	FBgn0032362
GB49979	-4.904307411	3.922275751	1.22672E-05	FBgn0043550
GB42890	-4.905604028	5.89594232	6.4459E-07	FBgn0039027
GB41904	-4.907472425	6.81923209	1.05998E-17	FBgn0030884
GB46386	-4.91301712	5.407530424	2.62001E-13	FBgn0050420
GB42580	-4.913995417	6.314129938	3.36435E-18	FBgn0261446
GB48967	-4.929588106	4.867347663	1.87104E-09	FBgn0260006
GB51787	-4.93659812	9.41056331	2.99533E-19	FBgn0002772
GB49401	-4.937038118	4.882325592	2.442E-09	FBgn0030257
GB50636	-4.963164009	9.196844097	4.67465E-20	FBgn0031097
GB49270	-4.967264033	3.974971812	8.19462E-06	FBgn0032402
GB49802	-4.979491775	5.840499613	3.35978E-14	FBgn0086906
GB48979	-5.000042478	4.957946378	2.28613E-10	FBgn0037323
GB48474	-5.003769054	9.951906886	2.71662E-21	FBgn0035398
GB49795	-5.022669546	7.469707682	2.79262E-12	FBgn0029681
GB52105	-5.050533717	6.322343394	5.65212E-11	FBgn0051807
GB53119	-5.059572344	10.27461094	3.74174E-12	0
GB50574	-5.063927966	5.347371478	2.26061E-06	FBgn0051561
GB40240	-5.07906629	10.60085808	8.67376E-19	FBgn0002773
GB53517	-5.0903041	6.453748069	2.21579E-16	FBgn0035935
GB42909	-5.123520862	6.133214261	3.05489E-10	FBgn0039027
GB54767	-5.128500968	4.106690252	2.36331E-06	FBgn0030947

GB40837	-5.131977626	9.946964316	9.27766E-21	FBgn0037323
GB50612	-5.150752422	4.862733946	5.50144E-05	FBgn0050101
GB53524	-5.157743436	7.638743803	1.21816E-18	FBgn0043792
GB43298	-5.195641863	9.388802988	1.26578E-18	FBgn0030539
GB50505	-5.20020672	6.984923936	1.0472E-07	FBgn0032536
GB53112	-5.201284026	6.032861077	3.17327E-15	FBgn0034289
GB52184	-5.20133813	6.566573206	5.42856E-20	0
GB40771	-5.209001121	6.584630874	8.68534E-21	FBgn0033763
GB40299	-5.225665665	5.095214389	2.35123E-08	FBgn0035513
GB50828	-5.227328837	7.733309261	1.13311E-20	FBgn0261836
GB45850	-5.232215992	9.397465583	4.2515E-16	FBgn0035636
GB41015	-5.261376716	11.86560702	9.15957E-24	FBgn0031957
GB49772	-5.261518662	6.735665951	1.23187E-12	FBgn0029681
GB42908	-5.308979012	6.525762316	3.20014E-08	FBgn0039027
GB48066	-5.312705556	6.147827156	8.45065E-19	FBgn0020389
GB40604	-5.316092425	6.538449632	6.83322E-09	FBgn0261526
GB46385	-5.322495424	4.329238054	1.35352E-07	FBgn0003292
GB41383	-5.334975854	5.189007021	3.80274E-08	FBgn0035788
GB48161	-5.364973733	9.671282629	8.48684E-23	FBgn0034391
GB51653	-5.376571988	11.50213939	1.21098E-20	FBgn0264695
GB47947	-5.425736042	8.108635449	1.25406E-21	FBgn0013988
GB49268	-5.431318343	5.342953824	1.69128E-13	FBgn0038837
GB48860	-5.468234985	6.272513028	2.10193E-16	FBgn0038819
GB50827	-5.515666778	8.34261141	4.27784E-21	FBgn0051205
GB54489	-5.533143841	5.460062199	2.09285E-15	FBgn0052829
GB51652	-5.54118547	8.856351513	1.42371E-19	FBgn0264695
GB42702	-5.553517274	9.856758185	1.9642E-24	FBgn0037288
GB42763	-5.626882386	7.991567755	1.36579E-21	FBgn0023388
GB45764	-5.628085043	8.11348145	1.53118E-22	FBgn0004117
GB44561	-5.638680894	6.7162285	7.37718E-19	FBgn0039075
GB51441	-5.659804983	11.71782084	3.704E-26	FBgn0019960
GB43181	-5.669761698	8.073281109	8.92631E-12	FBgn0037992
GB48107	-5.67121616	7.509795368	3.78135E-20	FBgn0017561
GB47752	-5.67807148	7.154193147	1.53204E-17	FBgn0001992
GB42911	-5.686775429	5.921264102	1.70245E-07	FBgn0053299
GB49689	-5.69838433	8.174291769	1.19704E-22	FBgn0004577
GB55100	-5.698601303	6.771440493	7.62528E-17	FBgn0003016
GB47118	-5.72962484	8.948216195	1.77079E-23	FBgn0038439
GB50562	-5.731825364	8.213861947	1.68094E-07	FBgn0037411
GB46973	-5.750173258	8.304950112	9.13244E-23	FBgn0263038
GB42912	-5.78890332	7.942726717	1.19258E-17	FBgn0053196
GB48861	-5.811916254	7.514993833	1.62522E-20	FBgn0038819
GB55614	-5.832397539	7.499334266	1.22106E-20	FBgn0011225
GB52634	-5.837307846	4.809996582	1.0221E-10	FBgn0040087
GB53120	-5.860395155	7.170134786	5.55599E-20	FBgn0013733
GB40447	-5.882934827	8.651582743	8.6541E-17	FBgn0033869
GB50563	-5.883237002	4.614939424	7.95893E-05	FBgn0037413
GB49021	-5.883730462	10.62075115	9.8961E-25	FBgn0034030
GB45860	-5.891897718	7.199745564	5.05317E-16	FBgn0066365
GB47126	-5.895739824	4.867065402	5.19466E-11	FBgn0004577
GB54791	-5.900710771	4.872948606	2.70353E-11	FBgn0003089
GB42582	-5.901817777	7.20257652	6.60602E-20	FBgn0035280
GB48174	-5.927315155	9.705478366	5.22463E-22	FBgn0033158
GB53110	-5.942960909	14.12357249	6.8628E-27	FBgn0034289
GB47550	-5.948815592	5.761671763	1.16654E-15	FBgn0052537
GB50613	-5.958593634	11.53710397	1.58924E-21	FBgn0050101

GB46518	-5.985827536	8.953630884	9.88118E-12	0
GB52052	-6.017306843	8.498174344	2.51944E-20	FBgn0029690
GB48173	-6.099895663	8.453035554	5.58318E-25	FBgn0033158
GB42597	-6.13397977	13.03752651	1.86784E-29	FBgn0032538
GB40624	-6.136362757	8.944943441	2.54615E-26	FBgn0259247
GB50875	-6.147274236	7.949667458	1.22351E-20	FBgn0053126
GB46585	-6.151727588	12.7367896	2.23647E-27	FBgn0032538
GB46591	-6.203496162	12.43374947	1.12512E-28	FBgn0032538
GB50442	-6.20580769	6.075424801	1.08959E-21	FBgn0039480
GB50449	-6.250275011	7.888936183	9.5565E-22	0
GB40659	-6.278391126	5.224259603	7.64406E-14	FBgn0043043
GB50451	-6.315005955	11.71534458	1.22376E-25	FBgn0053196
GB55612	-6.334357845	8.446978476	1.22376E-25	FBgn0263216
GB43769	-6.345322502	7.508605589	6.11743E-08	FBgn0037411
GB49394	-6.370538984	8.710313977	1.29816E-26	FBgn0259247
GB44399	-6.372248625	11.70118153	9.51302E-25	FBgn0050101
GB42888	-6.375866164	10.44978855	1.64607E-29	FBgn0053196
GB41624	-6.390529412	7.094602688	9.56119E-22	FBgn0035845
GB45957	-6.469093576	8.59068493	3.34109E-25	FBgn0034157
GB53525	-6.666614307	6.947096958	3.62627E-21	FBgn0035641
GB55599	-6.673623889	6.99200816	9.5983E-22	FBgn0052656
GB45763	-6.68566318	7.703670621	7.3201E-22	FBgn0004117
GB53113	-6.75553388	8.998949457	3.34109E-25	FBgn0040496
GB46414	-6.775798514	5.490457143	1.47678E-08	0
GB47903	-6.828087231	8.086577502	3.31662E-26	FBgn0036110
GB52104	-6.832826765	5.675314861	3.02205E-16	FBgn0039479
GB47946	-6.87511309	5.794020067	1.01541E-19	FBgn0013988
GB48794	-6.906083237	11.61150205	3.58626E-32	FBgn0004034
GB46394	-6.95173624	7.641176016	1.09364E-25	FBgn0035091
GB42910	-7.046498393	6.667044408	6.62905E-10	FBgn0053299
GB43877	-7.076041295	6.765485901	1.19762E-18	FBgn0034883
GB46399	-7.257683071	8.913178316	3.89876E-26	FBgn0032256
GB50876	-7.357848942	6.078127027	1.19691E-13	FBgn0053126
GB49981	-7.840435983	2.497368882	0.003059751	FBgn0030056
GB43805	-7.840435983	2.505384318	0.005729616	FBgn0029843
GB42923	-7.854314648	2.508635403	0.003217833	FBgn0015562
GB47536	-8.035778123	2.688680349	0.002286752	FBgn0038181
GB46403	-8.071862003	2.734406378	0.001185691	FBgn0036299
GB48044	-8.118611466	2.755880972	0.001584801	FBgn0005666
GB47129	-8.207797358	2.824937088	0.002196346	FBgn0041711
GB53313	-8.271245351	2.830567209	0.002104741	0
GB42635	-8.28155394	2.823128043	0.002920167	FBgn0085458
GB52635	-8.341904934	2.962246102	0.000404198	FBgn0034071
GB48796	-8.399832361	3.037085578	0.000176534	FBgn0012344
GB48489	-8.399832361	3.013259907	0.000482801	FBgn0031835
GB50611	-8.666880929	3.100639235	0.000996708	FBgn0050101
GB49962	-8.674723755	3.169430899	0.000464735	FBgn0004511
GB49295	-8.938473177	3.529689897	1.01333E-05	FBgn0014859
GB53689	-8.944973238	3.510224825	1.36985E-05	FBgn0032707
GB42641	-8.957886157	3.426289291	0.000105351	FBgn0039200
GB44199	-9.150168536	3.732171326	2.83119E-06	FBgn0052635
GB54457	-9.161376107	3.739067584	1.59643E-06	FBgn0052829
GB43367	-9.242767548	3.633996971	0.00020064	FBgn0053003
GB47120	-9.319811375	3.880090172	4.24908E-07	FBgn0038880
GB54904	-9.407141415	3.978870415	9.61901E-08	FBgn0032402
GB53285	-9.511548787	4.003998832	6.37447E-07	FBgn0002709

GB52017	-9.637279865	4.187954151	3.77301E-08	FBgn0037552
GB51819	-9.66510502	4.211463133	9.08161E-09	FBgn0030590
GB42581	-10.16569194	4.693007026	1.05534E-11	FBgn0035873
GB50887	-10.31617728	4.830383946	3.18149E-12	FBgn0029961
GB46417	-10.32617086	4.640635227	8.05254E-07	FBgn0037662
GB50577	-10.35818001	4.703918873	2.48321E-08	FBgn0037427
GB47927	-10.54091321	4.936161678	5.95629E-11	0

Contrast 5
High SBV+DWV vs. high DWV alone

Gene Label	logFC	logCPM	FDR	Flybase ortholog
GB50423	11.1294679	10.02259936	2.63866E-20	FBgn0052055
GB47618	10.73008124	6.846675562	6.29063E-12	FBgn0010385
GB51306	10.66243523	6.788443299	9.97726E-14	0
GB47318	10.45491324	9.337038613	6.40191E-14	FBgn0032835
GB41637	9.44816399	7.518765168	4.4782E-16	FBgn0030905
GB48823	9.059994519	8.849007475	5.87727E-17	FBgn0004778
GB47520	8.80285493	4.855140229	2.86879E-05	FBgn0045827
GB41428	8.737737519	9.050191849	6.40191E-14	FBgn0010385
GB43112	8.612284858	9.639760112	4.76932E-19	FBgn0028573
GB49890	8.399584379	7.817103953	1.58854E-10	FBgn0033302
GB41097	8.28640088	4.310261249	0.000633874	FBgn0051954
GB45912	8.285742369	11.01649526	2.6302E-20	FBgn0011296
GB48833	8.209042737	8.304320765	9.85224E-12	FBgn0004778
GB54238	8.133610133	6.972836359	4.48161E-13	FBgn0053547
GB53798	8.123963084	9.712437427	3.71765E-12	FBgn0032132
GB50121	8.067856435	9.468565805	2.36924E-19	FBgn0029167
GB42310	7.997920747	4.016861727	0.002901491	FBgn0045827
GB45954	7.906737128	6.792605007	4.1665E-09	FBgn0053196
GB47475	7.811441399	10.81471708	7.02186E-20	FBgn0011296
GB55435	7.735402372	5.736645493	5.19261E-09	FBgn0020626
GB54139	7.70989919	5.692771841	5.33886E-09	FBgn0000299
GB50363	7.68733836	3.708040187	0.017391675	FBgn0038642
GB42626	7.667318782	8.690130254	6.18207E-17	FBgn0005613
GB48148	7.62539667	7.678255802	2.12554E-17	FBgn0034140
GB48134	7.557757705	10.31468734	2.95337E-11	FBgn0001258
GB48146	7.453857028	6.813468517	9.62766E-09	FBgn0038071
GB51223	7.410290925	11.43488687	9.33872E-17	FBgn0014002
GB51174	7.161873481	9.974727698	9.33872E-17	FBgn0033661
GB50477	7.037438926	9.121530994	5.25023E-14	FBgn0263774
GB45910	7.033663626	12.22179445	1.00758E-17	FBgn0011296
GB55203	6.947876435	6.655438377	2.28278E-13	FBgn0034856
GB55515	6.932888132	8.725130529	1.2836E-10	FBgn0036262
GB42554	6.834766269	5.600380669	2.20095E-09	FBgn0036191
GB42540	6.83190309	7.978471707	7.69901E-17	FBgn0037329
GB42797	6.788055969	8.37528956	8.75404E-16	FBgn0037288
GB49219	6.737188256	4.712163821	0.00015029	FBgn0031905
GB44871	6.650904194	7.335061496	8.46375E-17	FBgn0038074
GB45909	6.627495586	11.38868179	1.00129E-16	FBgn0011296
GB40227	6.577143471	4.531621804	0.000164117	FBgn0036316
GB41722	6.447827503	10.19077215	1.06473E-11	FBgn0033246
GB45708	6.397102907	4.365162204	0.000553601	FBgn0031975
GB53860	6.305850813	9.367883266	2.09477E-13	FBgn0026575
GB41709	6.260343936	8.522190073	3.65266E-13	FBgn0030691
GB54881	6.226075161	6.254751387	3.07551E-10	FBgn0036829

GB45713	6.191347009	4.150863096	0.001671168	FBgn0031975
GB41636	6.101601962	6.329582157	3.46494E-14	FBgn0013733
GB43924	6.076098185	10.20877693	1.07679E-13	FBgn0263072
GB45906	6.068702278	11.12313033	1.87517E-14	FBgn0011296
GB43007	5.994173766	3.98774223	0.008845594	FBgn0030592
GB47805	5.989048573	5.666078553	2.7424E-09	FBgn0043575
GB42621	5.907641241	10.03641208	1.31023E-12	FBgn0016075
GB47505	5.715345478	3.623138046	0.006272339	FBgn0053870
GB51419	5.698987784	6.703772561	9.14619E-11	FBgn0035439
GB46223	5.694128476	4.473993142	0.000893744	FBgn0034470
GB45907	5.654590275	9.747264003	4.24378E-13	FBgn0011296
GB47127	5.632912132	4.400055677	0.000371029	FBgn0261832
GB52100	5.596284069	11.7717312	2.38575E-11	FBgn0000299
GB44633	5.552716247	3.484463769	0.019754841	0
GB49886	5.521563303	7.105955777	2.89026E-11	FBgn0033302
GB41222	5.395545605	4.144735037	0.0012432	FBgn0035132
GB51583	5.380060614	4.667098109	8.0076E-05	FBgn0036117
GB47270	5.358208027	4.131931043	0.00237264	FBgn0015032
GB44168	5.298498848	5.26404005	3.9326E-07	FBgn0000490
GB44996	5.25335743	10.44355556	4.96308E-09	FBgn0002564
GB40137	5.247545268	6.229461256	1.21462E-09	FBgn0038595
GB40288	5.244496579	4.97437399	0.000192078	FBgn0033302
GB42099	5.198372362	3.944261599	0.002823827	FBgn0027600
GB41708	5.191619434	9.119020515	1.24153E-10	FBgn0036597
GB44146	5.176476577	4.835076433	1.36054E-05	FBgn0034709
GB41284	5.175909775	7.145372657	5.73346E-12	FBgn0050197
GB42146	5.170313335	8.222071295	3.76735E-08	FBgn0085446
GB45714	5.159347267	3.054772915	0.046190762	FBgn0031975
GB42900	5.102074913	11.14627648	4.30045E-10	FBgn0261564
GB55451	5.096673349	10.17475596	1.59205E-11	FBgn0002526
GB40148	5.085940409	5.33318858	4.39313E-05	FBgn0032810
GB46587	4.927616862	9.127435911	7.28891E-10	FBgn0053998
GB47546	4.924760654	5.168641208	2.16899E-05	0
GB52348	4.895413392	4.540899352	7.95272E-05	FBgn0003162
GB55452	4.840728458	9.620107041	5.87037E-07	FBgn0052626
GB52598	4.820914594	9.84912665	3.98306E-11	FBgn0032213
GB43027	4.809952672	4.114970606	0.003512583	0
GB43208	4.746808998	9.215952778	8.92134E-11	FBgn0036985
GB41642	4.734071927	6.245593612	6.22006E-12	0
GB46640	4.731996688	4.688932696	3.86225E-05	FBgn0033628
GB42985	4.724179768	6.736660965	7.98055E-07	0
GB40708	4.64261627	9.161252959	3.59607E-10	FBgn0029507
GB52428	4.602525934	6.681691605	2.67718E-13	FBgn0037126
GB56028	4.575404306	7.44731739	1.44905E-10	FBgn0036665
GB55029	4.559006628	5.875961442	1.22246E-08	FBgn0031176
GB52919	4.547502276	7.993066475	3.37378E-09	FBgn0036617
GB43713	4.523567926	6.971255284	1.84874E-12	FBgn0038037
GB41706	4.49406445	5.289609618	5.46184E-06	FBgn0028533
GB42410	4.489310066	5.936982565	3.09258E-06	FBgn0034638
GB49442	4.459994459	3.739620352	0.012769134	FBgn0036237
GB42514	4.448993319	4.404175332	0.000235805	FBgn0037297
GB45911	4.421919414	6.192672842	4.57142E-11	FBgn0011296
GB49552	4.373032294	6.290686246	2.23257E-06	FBgn0035501
GB49441	4.365457036	8.385729962	5.04421E-09	FBgn0003450
GB48391	4.312443687	4.022446817	0.027093223	FBgn0038135
GB45913	4.309407012	12.26934563	1.72669E-09	FBgn0011296

GB44995	4.303260569	3.53617174	0.008079992	FBgn0051008
GB50880	4.293018318	4.516122209	0.000830375	FBgn0263968
GB46142	4.265784314	5.354535029	2.78182E-05	FBgn0026144
GB50609	4.251525129	11.80309353	1.72316E-08	FBgn0001219
GB50608	4.225453994	5.446458397	8.30743E-05	FBgn0033495
GB51218	4.223871587	5.735557378	1.85412E-08	0
GB54404	4.21162192	6.643048523	6.22006E-12	FBgn0051522
GB49440	4.209572911	6.688522666	4.45182E-12	FBgn0039630
GB49993	4.204165676	7.865052197	1.16919E-06	FBgn0035787
GB54219	4.198682952	10.11322659	3.53316E-08	FBgn0086708
GB52360	4.19666122	8.221367837	7.6453E-09	FBgn0034886
GB43739	4.174289215	4.137341659	0.005584259	FBgn0035779
GB50481	4.170530471	4.394178539	0.002522679	FBgn0023479
GB48820	4.151192817	10.9726422	1.29137E-08	FBgn0028985
GB54942	4.131732126	5.638044876	2.25238E-07	FBgn0051720
GB54289	4.127165786	6.66143285	1.438E-10	FBgn0085407
GB42244	4.123132921	8.600724768	4.03629E-09	FBgn0043903
GB45797	4.064664188	4.713818586	0.004469395	FBgn0039896
GB43716	4.004444538	6.013121291	8.5283E-09	FBgn0038037
GB49888	3.999365708	5.614989179	8.21336E-07	FBgn0000473
GB48903	3.980661144	5.287766586	3.6393E-06	FBgn0038139
GB53028	3.961433763	7.003973228	2.28983E-10	FBgn0032116
GB55204	3.958645823	5.506600717	0.00018384	FBgn0039896
GB50005	3.954222781	8.905968428	1.23642E-06	FBgn0063923
GB44503	3.89846719	4.521011337	0.000893744	FBgn0085201
GB49887	3.889107374	4.107168596	0.005214709	FBgn0033304
GB55205	3.880705361	6.694777373	7.01809E-09	FBgn0039896
GB52721	3.850395987	5.939238921	9.50989E-07	FBgn0002576
GB42217	3.849731549	4.051454581	0.004624744	FBgn0039755
GB51682	3.820926531	4.639529771	0.005937205	FBgn0029958
GB45955	3.810252639	9.910155643	1.12039E-07	FBgn0260746
GB48687	3.797587123	6.975583519	3.81927E-10	FBgn0243514
GB42135	3.768453279	5.185599528	3.71858E-05	FBgn0038201
GB49774	3.766635404	5.477297409	2.44078E-05	0
GB41418	3.751062352	5.463727319	2.53862E-05	FBgn0002526
GB50526	3.739130295	6.494129775	3.53949E-10	FBgn0038652
GB47248	3.727980819	6.153398154	5.37827E-09	FBgn0031146
GB54732	3.719844416	5.801399364	1.30113E-06	FBgn0030309
GB54941	3.717682464	6.637841386	8.97431E-10	FBgn0051720
GB40021	3.717257232	11.55896028	2.10028E-07	FBgn0036124
GB50883	3.67640867	6.364616072	4.99515E-07	FBgn0032638
GB49885	3.658369869	7.587589683	1.96453E-05	FBgn0033304
GB40967	3.647267501	6.061740656	3.14672E-06	FBgn0005626
GB50124	3.63900998	4.086339558	0.010339884	0
GB50137	3.624718287	5.490636873	1.22643E-05	FBgn0004378
GB43728	3.62381054	6.578149623	4.43512E-10	FBgn0038037
GB54231	3.606619038	4.248631565	0.014908015	FBgn0024352
GB49672	3.574643847	5.090872448	6.31963E-05	FBgn0038842
GB50906	3.557670475	6.354951169	7.10285E-07	FBgn0032421
GB41283	3.555979529	7.190061137	1.72123E-08	FBgn0003137
GB41202	3.54957026	6.080476368	6.10069E-08	FBgn0027600
GB46774	3.512426519	9.947412057	5.49594E-06	FBgn0031322
GB50761	3.474079449	4.691921194	0.003620539	FBgn0038485
GB47142	3.47332525	9.421042516	1.3691E-06	FBgn0030608
GB42053	3.457045108	5.411289174	0.000109527	FBgn0031381
GB54946	3.414699675	4.021887998	0.013709805	FBgn0040299

GB49154	3.410928532	5.635031349	1.5954E-05	FBgn0040491
GB48194	3.395088505	5.181952362	6.49957E-05	FBgn0039755
GB47580	3.394397729	6.023600882	2.89943E-07	FBgn0032817
GB47279	3.39183888	4.60230988	0.002581786	FBgn0033696
GB42433	3.337394243	5.203380583	7.80187E-05	FBgn0261797
GB52920	3.315744317	5.031226852	0.000545961	FBgn0036618
GB44634	3.305685002	5.936706167	7.98055E-07	FBgn0243512
GB45701	3.305206487	6.636634366	2.9042E-08	FBgn0027930
GB46286	3.304625289	4.519408679	0.005380208	FBgn0032144
GB50156	3.304483443	5.879180574	3.33889E-07	FBgn0039225
GB55764	3.301209626	4.399096323	0.010860903	FBgn0028675
GB55068	3.274341784	4.48878369	0.006254304	FBgn0085351
GB40136	3.260728279	4.221831579	0.012889064	FBgn0038595
GB45796	3.260728279	4.222521886	0.013486858	FBgn0004034
GB47940	3.213377272	9.529851625	5.68352E-06	FBgn0034096
GB55889	3.206775689	6.67049121	1.50089E-08	FBgn0035049
GB55212	3.184581598	5.314651796	0.004982172	FBgn0039896
GB45157	3.181250122	5.76766538	6.04768E-06	FBgn0013753
GB50290	3.171603769	7.066121407	1.38796E-07	FBgn0033033
GB53865	3.152581729	6.900088242	9.48524E-08	FBgn0013984
GB51345	3.14829286	8.774969826	1.17262E-05	FBgn0039151
GB53077	3.140942869	5.525809779	4.16025E-05	FBgn0039055
GB46013	3.13374675	7.11524215	1.82451E-07	FBgn0032010
GB54541	3.129977843	7.2249687	3.32388E-06	FBgn0031973
GB42829	3.092119576	9.017384796	1.61837E-05	FBgn0034405
GB48905	3.050156158	8.383869331	9.80306E-05	FBgn0010226
GB53549	3.034451015	5.372412182	0.000244146	FBgn0031037
GB46438	3.031800402	6.320625766	1.56074E-06	FBgn0027594
GB48969	3.020799911	5.012171231	0.01257857	FBgn0025592
GB48505	3.019333081	10.48827024	4.69493E-05	FBgn0026415
GB45248	3.014350744	7.979796248	1.76504E-05	FBgn0032180
GB52631	3.011900841	6.325690826	8.18971E-06	FBgn0003495
GB50508	3.006930726	6.84484898	5.57022E-07	FBgn0243514
GB51613	3.00349064	5.597791055	4.85555E-05	FBgn0263219
GB43362	2.999464487	6.5503424	6.87715E-07	FBgn0030421
GB53318	2.98482137	5.273202101	0.000358895	FBgn0001291
GB54097	2.975779751	8.599422118	5.39239E-05	FBgn0011672
GB52829	2.971310149	11.43432723	6.91716E-05	FBgn0026415
GB45696	2.970210713	7.344337974	1.76467E-05	FBgn0000567
GB53550	2.941681022	6.778667806	1.19144E-06	FBgn0031037
GB54233	2.928900705	5.436803803	0.000374555	FBgn0030964
GB47055	2.928522341	6.077508473	7.63118E-06	FBgn0031307
GB53978	2.928493018	6.16302977	0.000112841	FBgn0023479
GB47939	2.914688826	5.649213885	5.76266E-05	FBgn0040296
GB49327	2.894121953	5.952862962	1.5418E-05	FBgn0039131
GB47749	2.876287006	7.14375698	3.68958E-06	FBgn0039419
GB51650	2.873271261	5.214769574	0.000486029	FBgn0034392
GB53143	2.844166283	7.013944238	1.59843E-06	FBgn0016122
GB44213	2.836226639	7.81552594	3.9083E-05	FBgn0014141
GB49775	2.820079322	9.365406311	0.000159962	FBgn0011296
GB48841	2.800165742	4.996215553	0.003536907	FBgn0038819
GB40141	2.789285777	8.701243658	0.000229391	FBgn0038738
GB54949	2.788988982	5.865121042	8.28606E-06	FBgn0028978
GB53221	2.777496141	4.956290566	0.003967032	0
GB47929	2.741696942	5.133804064	0.000479945	FBgn0025686
GB52810	2.731104778	6.229821512	2.73058E-06	FBgn0039896

GB55301	2.716066468	8.31352636	0.000118883	FBgn0028926
GB42981	2.706519228	8.004356003	7.86665E-05	FBgn0040323
GB44004	2.698176104	5.60360255	0.000111066	FBgn0034490
GB48310	2.663025852	6.741548061	4.50031E-06	FBgn0038412
GB51671	2.660067223	6.895700507	5.94299E-06	FBgn0030869
GB51043	2.658475665	4.840658171	0.004126054	FBgn0023535
GB45775	2.647172864	5.653590853	4.12857E-05	FBgn0034166
GB49347	2.646804705	7.507882973	0.000374385	FBgn0033883
GB46686	2.63666322	5.468266441	0.000352342	FBgn0033913
GB47849	2.636030572	6.945931314	1.14861E-05	FBgn0038516
GB55030	2.626156667	9.514312475	0.000294743	FBgn0046706
GB54294	2.624572817	5.862834833	9.86468E-05	FBgn0035348
GB46612	2.622684021	5.238617705	0.000766702	FBgn0033936
GB44214	2.607353117	5.3094762	0.000776189	FBgn0014141
GB50448	2.603610916	5.221109616	0.001317943	FBgn0039804
GB51760	2.596439784	6.449294165	3.91853E-05	FBgn0000406
GB45700	2.584759896	9.941366322	0.000256475	FBgn0000533
GB47931	2.580878762	5.949451167	0.000554292	FBgn0036316
GB45704	2.580535095	6.582661318	4.18476E-06	FBgn0053229
GB45495	2.569338515	10.76757841	0.001044578	FBgn0001233
GB40759	2.567027289	9.76696466	0.000303519	FBgn0043841
GB45023	2.560984255	7.957597226	0.000576877	FBgn0032783
GB54426	2.555802313	6.760187035	0.00054277	FBgn0052512
GB48436	2.553070412	6.966267642	3.49811E-05	FBgn0002567
GB53755	2.53613462	5.404760605	0.00214791	FBgn0029690
GB54367	2.526672508	5.44399183	0.000401296	FBgn0030884
GB52642	2.517995215	7.330431434	0.000129961	FBgn0030251
GB45688	2.502991125	9.480239392	0.000410478	FBgn0033883
GB48344	2.490873997	5.252127767	0.001316194	FBgn0025631
GB42865	2.477322888	5.884402353	6.91716E-05	FBgn0036732
GB53141	2.472892164	5.432213956	0.000858873	FBgn0033786
GB44055	2.457433333	6.815773339	5.76266E-05	FBgn0000250
GB52278	2.433821968	5.724753568	0.000193787	FBgn0014141
GB44005	2.429623831	7.030626515	3.31501E-05	FBgn0034490
GB53847	2.418863848	6.738710386	4.33397E-05	FBgn0014906
GB55302	2.405830756	9.922677681	0.001292035	FBgn0033644
GB47415	2.39670957	7.511485092	0.000199516	FBgn0031538
GB40119	2.392882241	5.159703574	0.002425712	FBgn0016013
GB49390	2.386039242	5.883652527	0.000128027	FBgn0028341
GB48029	2.384604432	6.317868198	2.72291E-05	FBgn0032219
GB49614	2.384604432	6.320680072	3.05957E-05	FBgn0250732
GB54947	2.372459096	5.136967775	0.002546551	FBgn0036235
GB47301	2.360979071	5.860047533	0.000172314	FBgn0034605
GB54611	2.358699399	9.999000011	0.00327764	FBgn0028984
GB40758	2.349981488	8.940914669	0.001214083	FBgn0033926
GB52191	2.349750735	8.112223775	0.000529251	FBgn0051217
GB50550	2.348813828	8.325083082	0.000854748	0
GB40976	2.343726976	12.75568037	0.002416278	FBgn0001233
GB45676	2.335342058	5.815596697	0.000356876	FBgn0035617
GB42741	2.322183659	5.831294523	0.006734965	FBgn0050496
GB44344	2.320743845	7.3171673	0.000303519	FBgn0053120
GB45052	2.318334252	7.466221334	0.000674779	FBgn0026376
GB55590	2.313265837	8.948702798	0.000984127	FBgn0025814
GB42306	2.301308097	5.515299791	0.003829178	FBgn0262526
GB49688	2.300813162	9.051981537	0.001234944	FBgn0004577
GB50009	2.297087041	8.136106768	0.000916002	FBgn0040532

GB47740	2.294473594	8.571132389	0.001350073	FBgn0036995
GB42625	2.270434407	6.278131791	0.003600892	FBgn0037181
GB43573	2.267880393	8.091034006	0.000781777	FBgn0032638
GB51551	2.256057294	8.122736192	0.000907988	FBgn0035499
GB41301	2.25051006	10.002739	0.001571369	FBgn0000083
GB55016	2.234681772	10.37009001	0.001860511	FBgn0033188
GB55805	2.225654082	5.861241217	0.000348125	FBgn0050118
GB52115	2.221816296	8.204016578	0.002909478	FBgn0025456
GB52630	2.219595795	5.621702021	0.001137046	FBgn0036101
GB52341	2.211675855	6.855027618	0.000240767	FBgn0013987
GB47463	2.176956201	7.546175636	0.000902337	FBgn0001104
GB50421	2.176420837	10.94801288	0.00237264	FBgn0000416
GB43184	2.174915406	8.589329123	0.001871085	FBgn0030245
GB40735	2.169565228	9.74164176	0.002425712	FBgn0000064
GB50226	2.165916353	11.53103881	0.0032643	FBgn0022355
GB51665	2.160479924	8.196623377	0.001402126	FBgn0032074
GB48598	2.155966648	6.679099249	0.000226763	FBgn0011674
GB55070	2.154799145	6.426744375	0.000410478	FBgn0027843
GB41549	2.153648591	5.835222283	0.000309466	FBgn0011837
GB40603	2.14793718	6.290809779	0.000139549	FBgn0022800
GB47104	2.141115287	8.349209133	0.002181123	FBgn0034162
GB55998	2.138770069	6.355442651	0.000217748	FBgn0027538
GB50043	2.124181108	6.786804347	0.000322647	FBgn0259178
GB47478	2.121335319	8.47788912	0.001693785	FBgn0035438
GB51741	2.119083862	7.55919038	0.001189737	FBgn0030310
GB41735	2.085295603	7.458408646	0.001577252	FBgn0036565
GB42329	2.059314509	7.419998288	0.001598201	FBgn0262975
GB44710	2.046556545	7.091841322	0.002063658	FBgn0037684
GB50149	2.046556545	7.08451977	0.004698561	FBgn0040827
GB43784	2.046334226	6.281829265	0.000379969	FBgn0035770
GB43572	2.045306967	8.346630067	0.002738623	FBgn0015575
GB49657	2.019613289	7.118640227	0.00292614	FBgn0004606
GB41225	2.012960764	6.313732562	0.000338009	FBgn0033635
GB55511	2.00121697	6.138309604	0.000681667	FBgn0031461
GB42685	2.000744467	6.875593175	0.001871085	FBgn0040323
GB46537	1.99891572	6.400803192	0.000545774	FBgn0000477
GB48626	1.992228673	7.371940057	0.006077124	FBgn0031528
GB53401	1.970447656	6.410395201	0.000641794	FBgn0028550
GB42084	1.965722966	6.880210306	0.000738543	FBgn0035132
GB41388	1.962230298	6.560382733	0.000851409	FBgn0001128
GB50418	1.962140154	6.889174122	0.001860511	FBgn0262473
GB43945	1.946903128	6.420583842	0.000538417	FBgn0061200
GB50136	1.940316455	8.75173772	0.009068093	FBgn0037007
GB42616	1.932141135	6.789666579	0.004348582	FBgn0041629
GB49259	1.932043893	7.7198349	0.018005284	FBgn0030872
GB42142	1.922952246	5.956072493	0.001416845	FBgn0001078
GB44143	1.920585251	6.620419805	0.005320678	FBgn0037022
GB47880	1.91872998	9.166710401	0.008632417	FBgn0003462
GB44976	1.913210764	6.193478188	0.001292035	FBgn0016076
GB48634	1.912544229	9.179991969	0.00760188	FBgn0035438
GB47310	1.910070789	6.369419152	0.00110543	FBgn0030791
GB40565	1.90599151	6.403210615	0.000766702	FBgn0262866
GB45497	1.898342109	7.041388227	0.003828187	FBgn0050296
GB49123	1.895946761	6.915911053	0.003239973	FBgn0035719
GB53805	1.886414345	6.510956601	0.001139971	FBgn0038098
GB41807	1.875536478	8.324370726	0.007167109	FBgn0030740

GB55096	1.868914857	9.325395098	0.009578195	FBgn0002719
GB46276	1.868050528	8.437423948	0.007728444	FBgn0033799
GB45973	1.864908068	6.61382532	0.018411106	FBgn0000422
GB44100	1.863882962	6.243064537	0.000931191	FBgn0013733
GB42738	1.859629136	6.150468026	0.001639531	FBgn0011204
GB51210	1.844169622	7.069888074	0.005320678	FBgn0039492
GB41604	1.835490013	8.116479786	0.009038786	FBgn0030529
GB47565	1.812195243	9.361499589	0.024241963	FBgn0023095
GB44882	1.803122677	7.185970936	0.00701291	FBgn0035094
GB49607	1.787752204	9.993790073	0.01456849	FBgn0032949
GB42141	1.786718732	9.737369855	0.015604466	FBgn0035811
GB55482	1.784194224	7.375680788	0.008559697	FBgn0010620
GB45617	1.777496369	6.954969592	0.00548385	FBgn0028343
GB45736	1.776791168	7.771882062	0.010624785	FBgn0016693
GB49826	1.764792548	7.297210639	0.015265522	FBgn0037612
GB43708	1.745131927	12.45255587	0.01834029	FBgn0015221
GB52158	1.74331546	6.781420946	0.008605342	FBgn0031975
GB45135	1.742244954	6.910040695	0.005555039	FBgn0028982
GB43302	1.724826988	9.279614225	0.019986414	FBgn0026562
GB42500	1.723433871	6.751519509	0.004641824	FBgn0035976
GB40866	1.720461906	13.22053821	0.021345151	FBgn0001219
GB53830	1.700732034	7.860928987	0.02160631	FBgn0039728
GB47201	1.68586055	8.667132058	0.02062881	FBgn0004657
GB53831	1.682786195	7.499630232	0.021476733	FBgn0033382
GB48853	1.667454286	7.758830587	0.02042266	FBgn0032456
GB45147	1.657843662	8.927441415	0.027204902	FBgn0031913
GB53632	1.654916139	8.31961585	0.029570274	FBgn0039257
GB50970	1.653735966	9.49420017	0.024905514	FBgn0040064
GB47107	1.632996907	8.430379879	0.027106639	FBgn0036165
GB49240	1.630562532	7.542460778	0.019815253	FBgn0012036
GB42940	1.606339713	7.71791057	0.022977086	FBgn0013305
GB49260	1.604775797	10.30777817	0.030416071	FBgn0262720
GB40773	1.593589947	7.605255398	0.024241963	FBgn0030485
GB51029	1.570821358	7.049783016	0.022473566	FBgn0004049
GB46766	1.563398983	7.636379496	0.027380265	FBgn0034577
GB46984	1.562659594	7.139247625	0.019465125	FBgn0086691
GB41806	1.561418526	7.688315584	0.035853756	FBgn0038088
GB55426	1.559281449	8.697221788	0.039322274	FBgn0087007
GB41313	1.551176584	7.264888541	0.025714238	FBgn0036501
GB53540	1.544356833	6.9340229	0.02471976	FBgn0263350
GB52074	1.540553573	8.295490239	0.035138568	FBgn0004654
GB50931	1.522736871	7.291687904	0.031179281	FBgn0003507
GB52427	1.513327891	7.460006337	0.033264633	FBgn0024238
GB54331	1.503691419	10.21874237	0.046190762	FBgn0013770
GB48195	1.493162176	7.504298126	0.03407712	FBgn0086687
GB54863	1.467324303	7.624413296	0.045963195	FBgn0250848
GB50271	1.453861019	7.127277501	0.037694882	FBgn0035432
GB47955	1.4102668	7.239646669	0.047776084	FBgn0005278
GB48699	-1.327444838	10.88358365	0.049799333	FBgn0013325
GB53219	-1.363250112	10.59762065	0.037042583	FBgn0005533
GB40703	-1.369339769	7.842591609	0.037617358	FBgn0036715
GB55643	-1.390595132	8.155623553	0.029079321	FBgn0016691
GB42663	-1.423332182	7.837554323	0.024241963	FBgn0031066
GB50683	-1.425346407	7.569601357	0.027224549	FBgn0037855
GB55979	-1.463666951	7.59376426	0.02042266	FBgn0031528
GB49177	-1.482639722	10.34414547	0.022806701	FBgn0039359

GB48929	-1.485872747	8.158404089	0.023640263	FBgn0020660
GB55781	-1.538202103	7.805063041	0.020555712	FBgn0053196
GB47650	-1.548547023	8.534401336	0.012229552	FBgn0023170
GB48454	-1.576819997	7.623393263	0.011641998	FBgn0000108
GB45152	-1.577120029	10.69043703	0.012644294	FBgn0261341
GB47938	-1.580311422	8.31249236	0.011759668	FBgn0031879
GB52997	-1.602972421	7.378934345	0.008996253	FBgn0262110
GB41633	-1.610841162	7.902875621	0.008237535	FBgn0016120
GB46776	-1.629293044	10.44334748	0.008469441	FBgn0033699
GB54570	-1.631076214	7.187397513	0.004859238	FBgn0034697
GB48837	-1.640712086	7.738980989	0.01257857	FBgn0039008
GB40445	-1.651548696	9.071397639	0.007979069	FBgn0031918
GB44903	-1.662306724	7.607081978	0.007144354	FBgn0015614
GB43198	-1.673635116	7.214867154	0.013178234	FBgn0000667
GB41143	-1.68710428	7.76379005	0.005165162	FBgn0037001
GB40808	-1.69147899	7.221097611	0.005560661	FBgn0029688
GB51342	-1.699523814	7.277006744	0.003606574	FBgn0014023
GB53946	-1.70112752	7.500089854	0.006039552	FBgn0040793
GB44558	-1.71323322	6.988623429	0.003157565	FBgn0036691
GB41973	-1.718220817	6.999806583	0.004675231	FBgn0037843
GB50101	-1.730857515	6.981502715	0.003751145	FBgn0039594
GB41625	-1.744719583	8.219231196	0.004365864	FBgn0035844
GB46268	-1.744745329	7.947946983	0.004326957	FBgn0024183
GB54384	-1.744892888	6.977913322	0.002183164	FBgn0033961
GB50061	-1.754792337	7.15499818	0.003499097	FBgn0001321
GB53090	-1.759010734	6.690276333	0.000707447	FBgn0036557
GB40395	-1.768454405	8.077029823	0.002535696	FBgn0045866
GB45635	-1.770523765	6.637259466	0.000905021	FBgn0035122
GB44897	-1.773856107	8.085985375	0.003133764	FBgn0259749
GB44693	-1.792102917	8.834483716	0.003081169	FBgn0034259
GB55683	-1.794075067	6.896817134	0.001616529	FBgn0010808
GB54555	-1.816099585	6.727112349	0.000528514	FBgn0039868
GB55568	-1.816139814	6.974157839	0.002782362	FBgn0038742
GB49106	-1.818677504	8.070768253	0.002846594	FBgn0250789
GB40203	-1.829177837	6.899524167	0.002368222	FBgn0051028
GB49764	-1.850309995	6.526245801	0.001871085	FBgn0261277
GB45937	-1.856422072	7.23144863	0.002446304	FBgn0013765
GB42612	-1.861726346	8.043755331	0.00310255	FBgn0033603
GB41270	-1.87908515	8.918880193	0.002655037	FBgn0026077
GB45434	-1.880270003	8.29373075	0.001139971	FBgn0010612
GB50690	-1.883889523	6.751875806	0.001616529	FBgn0261397
GB55479	-1.887724811	6.45671869	0.000220327	FBgn0041249
GB43540	-1.8930137	9.282847178	0.00132543	FBgn0263968
GB53749	-1.89652136	7.952231743	0.001717837	FBgn0034877
GB52783	-1.897388443	6.71434863	0.000476527	FBgn0039635
GB48641	-1.898193368	6.662381957	0.00021562	FBgn0038319
GB44033	-1.918487822	10.37756029	0.001175091	FBgn0031980
GB51802	-1.920689465	6.500179334	0.00021562	FBgn0052043
GB51736	-1.923372993	7.270995208	0.002113821	FBgn0031957
GB43629	-1.924315757	7.155379058	0.000582404	FBgn0021967
GB54652	-1.924730834	7.125572062	0.000479526	FBgn0039765
GB55782	-1.934223396	7.022741874	0.000454107	FBgn0033570
GB48813	-1.936109648	6.461443998	0.014658972	FBgn0066365
GB41648	-1.939356311	6.535216371	0.000193891	FBgn0067317
GB49534	-1.951877034	8.999321683	0.001127394	FBgn0027341
GB40399	-1.953686703	6.7458885	0.000410478	FBgn0260858

GB41068	-1.955110285	6.710628368	0.000476224	FBgn0011787
GB42804	-1.956128419	7.364023124	0.001005659	FBgn0033192
GB45947	-1.956267069	6.651345355	0.000237911	FBgn0024841
GB40590	-1.972622495	6.236169824	7.80187E-05	FBgn0031766
GB49095	-1.987035349	6.406239472	0.000583685	FBgn0062413
GB54643	-1.991722581	7.128314461	0.001218771	FBgn0019960
GB41855	-1.993220557	6.325275188	0.000881531	FBgn0262867
GB44891	-1.993739068	6.551814366	9.80306E-05	FBgn0039212
GB50918	-1.993739068	6.544386563	0.00015965	FBgn0031684
GB53270	-1.993805737	6.298691719	8.32812E-05	FBgn0028919
GB55477	-1.994038277	6.904135599	0.000440769	FBgn0037608
GB54387	-1.994864087	6.836771804	0.000195252	FBgn0037899
GB40801	-1.995736326	6.368149564	0.000228634	FBgn0031601
GB51063	-2.005613145	8.178295473	0.00186094	FBgn0051973
GB47177	-2.01509367	6.483922425	0.000204549	FBgn0037549
GB51624	-2.036551913	6.220710894	3.87952E-05	FBgn0035534
GB45866	-2.039018995	6.641424207	0.000146784	FBgn0035374
GB46701	-2.042819624	6.187027126	0.000656429	FBgn0051204
GB51046	-2.043656088	8.15185693	0.000382158	FBgn0030306
GB40492	-2.046547606	9.389756125	0.000545774	FBgn0030616
GB43231	-2.055355765	6.484074961	0.001311303	FBgn0035888
GB47181	-2.063791899	7.246100332	0.000241296	FBgn0031021
GB44608	-2.064102759	6.728001446	6.36255E-05	FBgn0034645
GB54100	-2.073655376	6.320368922	5.99018E-05	FBgn0035469
GB41290	-2.080193511	6.61506416	0.000273139	FBgn0051224
GB52250	-2.090185876	6.287093548	0.0001693	FBgn0260390
GB54213	-2.094526184	6.660265818	0.000769605	FBgn0052000
GB49086	-2.095023607	6.253072887	3.73114E-05	FBgn0030407
GB51900	-2.099932668	6.394800866	3.81338E-05	FBgn0038746
GB54976	-2.101047419	7.009273988	0.000232481	FBgn0034791
GB43482	-2.101168343	8.739559502	0.000363242	FBgn0019644
GB47886	-2.103446087	6.198466192	3.53139E-05	FBgn0037172
GB41203	-2.105655541	10.10120513	0.000781777	FBgn0026077
GB42239	-2.109459995	6.629599548	0.000434788	FBgn0034943
GB49930	-2.1106919	6.383539506	0.000240975	FBgn0028491
GB40609	-2.114191625	6.775124848	0.001356693	FBgn0039210
GB52644	-2.117394849	7.699431156	0.000223644	FBgn0016119
GB55912	-2.11783314	7.10555769	0.000319475	FBgn0026059
GB48981	-2.117848556	7.093599292	0.000188126	FBgn0034517
GB51993	-2.134383112	5.972897504	4.93042E-05	FBgn0035592
GB48293	-2.135958195	6.668844597	2.85351E-05	FBgn0039159
GB55275	-2.138407407	7.379969028	0.000204549	FBgn0036289
GB53661	-2.142892574	6.856751424	6.28793E-05	FBgn0035247
GB51722	-2.149026991	6.654701239	0.000314656	FBgn0035936
GB52446	-2.159959684	5.954090664	0.000473346	FBgn0020300
GB54390	-2.16300256	6.174222105	0.000264503	FBgn0025393
GB46748	-2.164125783	6.687574327	2.25596E-05	FBgn0034579
GB41308	-2.176512885	7.746175833	0.000432674	FBgn0000046
GB41196	-2.194717298	6.221473967	0.000311892	FBgn0023518
GB40489	-2.203309252	7.857514451	0.00010017	FBgn0019957
GB49584	-2.210317886	6.936805205	8.30743E-05	FBgn0086712
GB51581	-2.211105611	6.739534739	1.96492E-05	FBgn0034854
GB43808	-2.220674652	6.419748485	1.97541E-05	FBgn0036237
GB40462	-2.22478701	7.693913988	0.000843204	FBgn0040773
GB52028	-2.233168682	8.04126268	0.00017091	FBgn0000556
GB53716	-2.234076402	6.040797924	2.27241E-05	FBgn0033887

GB53568	-2.241777581	7.776351144	0.000545774	FBgn0263911
GB43187	-2.244182503	6.137476614	5.19374E-05	FBgn0085468
GB54450	-2.258160605	6.310785979	3.04087E-06	FBgn0038302
GB47224	-2.279644376	6.811014507	0.000125079	FBgn0000568
GB49283	-2.281488415	6.337188781	0.000255734	FBgn0036398
GB50335	-2.281543905	7.067360976	2.59557E-05	FBgn0050354
GB49115	-2.287965406	6.475361587	4.58254E-05	FBgn0062413
GB53947	-2.290639292	6.200375563	2.95564E-06	FBgn0037890
GB47948	-2.29592373	5.91802964	0.000303519	FBgn0013988
GB54469	-2.299685305	6.135751171	3.68958E-06	FBgn0035980
GB54483	-2.301175387	6.713438258	8.15091E-05	FBgn0035539
GB46272	-2.302988585	6.025985801	4.85932E-05	FBgn0085220
GB42487	-2.30327337	6.667148487	7.91422E-05	FBgn0260450
GB42493	-2.303778516	6.38777502	0.000201287	FBgn0028573
GB51623	-2.304205359	6.226640836	1.02957E-05	FBgn0033754
GB50167	-2.310793402	6.332362377	5.84154E-06	FBgn0036774
GB51539	-2.312899375	6.543714921	3.91262E-06	FBgn0034986
GB56016	-2.315243541	6.735362318	1.59195E-05	FBgn0029718
GB47977	-2.315413909	9.214539552	0.000123866	FBgn0086906
GB44842	-2.319819128	6.250950685	6.33006E-05	FBgn0082582
GB42015	-2.323513171	6.305102858	1.0575E-05	FBgn0037838
GB55615	-2.32815251	7.717337388	9.2716E-05	FBgn0034860
GB40653	-2.331448137	10.20202733	3.33157E-05	FBgn0032518
GB40892	-2.33564666	6.219968874	5.49594E-06	FBgn0260401
GB41359	-2.336976019	7.083929872	2.44089E-05	FBgn0044030
GB50866	-2.338051364	7.156303537	4.73211E-05	FBgn0010435
GB51800	-2.342204678	7.934640946	2.99906E-05	FBgn0015903
GB53970	-2.350284061	7.709353303	5.48946E-05	FBgn0035574
GB50032	-2.351654347	7.286542131	2.59557E-05	FBgn0036918
GB53343	-2.352292943	5.926609882	0.000108964	FBgn0043903
GB47176	-2.359428678	5.958883753	1.33584E-05	FBgn0033480
GB54818	-2.366398724	6.400012502	0.000116718	FBgn0014863
GB55359	-2.370622729	7.082714137	7.46977E-05	FBgn0036365
GB55765	-2.374123613	5.900184049	0.00011146	FBgn0000442
GB45025	-2.382086243	7.296240822	1.21906E-05	FBgn0037008
GB54301	-2.383153675	6.797763151	2.76981E-05	FBgn0030605
GB48484	-2.385013236	6.249695502	3.99921E-06	FBgn0038426
GB48958	-2.401392805	7.096421159	7.07342E-06	FBgn0040931
GB44426	-2.401486762	6.189078487	5.45762E-06	FBgn0037537
GB42959	-2.405396576	6.087335681	2.21035E-06	FBgn0000183
GB46785	-2.422003117	5.770666183	1.61317E-05	FBgn0037378
GB42661	-2.426742479	6.05473006	3.55979E-06	FBgn0051759
GB44647	-2.433341878	5.561726195	2.56491E-05	FBgn0261999
GB42043	-2.448572808	7.088964141	5.49594E-06	FBgn0051159
GB43552	-2.459583615	6.775844557	2.27941E-05	FBgn0035575
GB43537	-2.476993401	6.512585596	9.50989E-07	FBgn0033907
GB50601	-2.477980804	8.365990527	1.00241E-05	FBgn0038224
GB53010	-2.485588641	8.212184749	1.38664E-05	FBgn0037579
GB44798	-2.492948271	6.801002617	1.9578E-05	FBgn0034797
GB47199	-2.496099205	6.485890188	3.33361E-06	FBgn0010222
GB44223	-2.497194833	6.772154862	8.93519E-05	FBgn0027611
GB50441	-2.500151858	7.279213552	9.94927E-05	FBgn0038405
GB51206	-2.514970453	8.026933571	5.32838E-06	FBgn0015031
GB46817	-2.518110643	8.987894543	0.000303519	FBgn0053257
GB47499	-2.522018828	7.134204358	1.38577E-05	FBgn0085384
GB51118	-2.523640049	6.714843451	6.42768E-07	FBgn0034361

GB45211	-2.541351588	7.791927538	4.15675E-05	FBgn0010423
GB46793	-2.542284778	6.067317301	4.22213E-07	FBgn0050185
GB49223	-2.547785391	6.909813745	1.79236E-05	FBgn0259108
GB40032	-2.564658255	6.321488476	1.59477E-07	FBgn0031361
GB44799	-2.582162344	6.386023953	8.46454E-07	FBgn0030060
GB53331	-2.590075421	6.06159362	8.28606E-06	FBgn0039294
GB54921	-2.590870715	5.662195266	1.39601E-05	FBgn0037487
GB43639	-2.594196819	6.058466283	2.97378E-07	FBgn0034939
GB53037	-2.615824187	5.813323295	1.73849E-06	FBgn0038662
GB45364	-2.618481269	5.98304064	1.37662E-05	FBgn0261509
GB47838	-2.619566077	7.32339512	4.21639E-06	FBgn0034974
GB50453	-2.628743472	5.714259966	1.31136E-06	FBgn0039805
GB45771	-2.647086965	5.391141542	4.31901E-06	0
GB50928	-2.650007597	5.826799719	2.14019E-06	FBgn0040389
GB53539	-2.650797103	6.001591672	2.93434E-07	FBgn0039764
GB50564	-2.657368997	6.339313152	0.000665942	FBgn0027527
GB40503	-2.661300986	7.888547787	3.08312E-06	FBgn0032350
GB40165	-2.662325239	9.44743991	1.76467E-05	FBgn0003065
GB55944	-2.666048809	5.67860226	1.30113E-06	FBgn0038323
GB44931	-2.668121049	5.371804711	2.44089E-05	FBgn0028436
GB55650	-2.711235687	6.388321009	4.03841E-06	FBgn0011286
GB48709	-2.716039207	5.513522176	2.13466E-06	FBgn0033061
GB50870	-2.723194763	6.107715415	5.40693E-08	FBgn0034001
GB40008	-2.724047522	5.912785795	8.11535E-06	FBgn0261836
GB45629	-2.727727066	8.484376572	1.01117E-06	FBgn0031228
GB44452	-2.734678797	5.967633348	7.86523E-06	FBgn0032803
GB53319	-2.737980666	6.507838312	6.02772E-06	FBgn0034301
GB50408	-2.738123565	6.938124704	5.59792E-07	FBgn0036726
GB48007	-2.739150814	7.164906122	7.07342E-06	FBgn0034412
GB51003	-2.73935178	6.341890865	9.43215E-09	FBgn0051913
GB43983	-2.772249722	5.747104069	8.46454E-07	FBgn0030617
GB49308	-2.776118138	6.96703872	3.52045E-07	FBgn0085638
GB47976	-2.779899719	5.244101425	2.96901E-06	FBgn0035308
GB51442	-2.782066374	6.568064169	9.92973E-06	FBgn0052036
GB54743	-2.785944684	5.715363837	1.91639E-07	FBgn0004959
GB50000	-2.794144374	8.122885689	1.31136E-06	FBgn0032453
GB42903	-2.800808794	5.699963119	0.011886599	FBgn0045443
GB40842	-2.804291229	6.45835156	1.21615E-06	FBgn0001112
GB42821	-2.819438428	6.766229864	1.47919E-08	FBgn0036581
GB42427	-2.823251105	7.314929786	2.21031E-06	FBgn0039640
GB45122	-2.830463095	5.6541747	1.40656E-06	FBgn0029514
GB48858	-2.845565689	7.056638886	2.36839E-06	FBgn0040601
GB41656	-2.848514787	5.253222116	1.33715E-05	FBgn0069913
GB45149	-2.855752934	6.102082653	5.81023E-06	FBgn0032785
GB55225	-2.865152577	6.681832153	6.06324E-07	FBgn0001083
GB44683	-2.865232759	7.916433103	2.77709E-06	FBgn0250838
GB45609	-2.883699026	6.765898859	1.62619E-05	FBgn0033079
GB52025	-2.912701929	7.366243705	4.91814E-07	FBgn0027570
GB40862	-2.914197837	5.930529863	3.81791E-08	FBgn0033085
GB50236	-2.915624423	5.633878009	0.000382158	FBgn0035788
GB46975	-2.922234984	5.409125246	0.000344162	FBgn0002183
GB55547	-2.924836575	7.033456504	8.02485E-06	FBgn0050172
GB47822	-2.929972888	5.575523877	1.30113E-06	FBgn0034726
GB47990	-2.93398	6.618919424	1.35977E-06	FBgn0004117
GB47321	-2.969859969	6.572023746	6.47462E-09	FBgn0039111
GB50660	-2.972867621	5.631393875	1.92519E-07	FBgn0036995

GB52690	-2.976273653	5.13811868	1.52693E-05	FBgn0033100
GB50981	-2.981433837	5.529320857	1.7797E-06	FBgn0035169
GB50567	-2.982847553	6.306674881	3.52045E-07	FBgn0037416
GB42062	-2.985213007	7.818538337	8.53372E-07	FBgn0039527
GB50171	-2.985773889	6.206905787	6.975E-07	FBgn0262717
GB45188	-2.992875536	6.712056213	1.37209E-07	FBgn0031630
GB54775	-2.997157715	8.022691908	3.39643E-07	FBgn0030027
GB55231	-3.007833322	6.182566338	2.10028E-07	FBgn0033602
GB40446	-3.034039497	6.15031963	2.34413E-07	FBgn0033869
GB55298	-3.055273971	6.345242246	2.91708E-09	FBgn0011227
GB51560	-3.066020896	8.674698329	1.00812E-07	FBgn0034022
GB49275	-3.070203161	5.118221432	3.77562E-06	FBgn0038837
GB53345	-3.077139359	7.708070269	1.09853E-07	FBgn0037217
GB44832	-3.091437957	5.549782504	1.82451E-07	FBgn0038629
GB46705	-3.097710079	7.563248322	1.09993E-06	FBgn0053519
GB51482	-3.104783177	6.524094003	1.82304E-07	FBgn0085446
GB43052	-3.122878124	7.7019461	5.08634E-07	FBgn0003149
GB55403	-3.129093939	6.054023098	1.09979E-07	FBgn0259994
GB47082	-3.137611276	5.394602557	4.12206E-07	FBgn0030660
GB43465	-3.14961518	5.824160482	5.69386E-08	FBgn0039208
GB41945	-3.158678937	6.643498856	1.87254E-07	FBgn0031097
GB41306	-3.15891823	7.730512009	5.59792E-07	FBgn0000046
GB43892	-3.161350777	7.307320155	5.13363E-08	FBgn0032945
GB46298	-3.20783493	6.141627604	1.90327E-06	FBgn0033728
GB45553	-3.211942463	5.130911944	5.4835E-07	FBgn0034157
GB47037	-3.230666843	6.813651649	1.06496E-07	FBgn0264562
GB49639	-3.23320562	6.692633924	8.10694E-07	FBgn0039648
GB49080	-3.257814369	7.757744553	3.90859E-08	FBgn0052645
GB48125	-3.294743104	6.040281701	1.88243E-07	FBgn0031869
GB50973	-3.311391598	6.113710684	2.4472E-07	FBgn0036044
GB41310	-3.314256815	8.625245921	9.068E-09	FBgn0000046
GB55729	-3.330238004	6.15063216	1.58278E-08	FBgn0041712
GB42892	-3.348568288	4.951402041	6.96439E-07	FBgn0004650
GB42303	-3.364162017	5.654384733	5.26425E-08	FBgn0052816
GB47906	-3.394451082	8.82793568	2.44953E-09	FBgn0033731
GB44209	-3.395423774	8.215981342	4.1665E-09	FBgn0037537
GB51901	-3.395605373	6.195743078	1.90556E-08	FBgn0052645
GB54393	-3.398159455	6.200558129	1.26666E-08	FBgn0031646
GB41723	-3.4124176	7.90697497	1.60796E-09	FBgn0261269
GB42206	-3.413615738	5.993259911	1.60084E-07	FBgn0260856
GB52723	-3.429080356	5.695670408	4.0651E-08	FBgn0262647
GB43053	-3.446057825	7.52937411	5.45541E-08	FBgn0003149
GB50566	-3.462873495	6.028320282	0.000641464	FBgn0037415
GB41311	-3.485890491	7.463354007	5.0934E-09	FBgn0000045
GB48167	-3.48721605	6.733726181	1.6683E-08	FBgn0262508
GB43691	-3.489520401	5.987545861	3.63168E-08	FBgn0038017
GB55325	-3.491202857	5.734348621	6.34476E-08	FBgn0003721
GB44222	-3.494020601	6.37587391	7.67195E-08	FBgn0039897
GB40007	-3.494609496	5.214767521	5.21119E-08	FBgn0261836
GB54302	-3.516694832	5.020214805	3.09066E-06	FBgn0038986
GB54750	-3.523856586	4.896540821	1.0459E-06	FBgn0259226
GB47669	-3.527785298	11.00058167	1.09221E-10	FBgn0015766
GB41622	-3.588230139	4.584139451	0.00237264	FBgn0035429
GB50565	-3.589764369	6.569122076	0.000380353	FBgn0037414
GB54884	-3.600956023	8.209769842	2.15865E-10	FBgn0053978
GB41241	-3.603548881	4.816428302	2.26867E-07	FBgn0033633

GB55158	-3.630864245	5.848075475	1.28464E-08	FBgn0027495
GB55598	-3.651894415	7.64948384	7.06927E-09	FBgn0004028
GB43029	-3.665056301	7.577843955	1.515E-08	FBgn0000047
GB53675	-3.710591707	5.225246373	2.35955E-08	FBgn0031999
GB53407	-3.711856244	4.860016686	0.003898501	FBgn0053196
GB42769	-3.713678312	9.88287298	5.75596E-11	FBgn0038405
GB42594	-3.719827475	5.657822076	2.13441E-10	FBgn0036834
GB55617	-3.731780256	5.290328005	7.47773E-10	FBgn0029836
GB50170	-3.738678998	7.309725846	9.09148E-11	FBgn0262717
GB50570	-3.749245918	5.923129689	5.54174E-08	FBgn0037419
GB42908	-3.768930456	4.738037636	0.000721067	FBgn0039027
GB41495	-3.773615913	4.718074182	2.45462E-07	FBgn0016693
GB49734	-3.783009876	5.046366287	1.4893E-06	FBgn0260386
GB52184	-3.795852386	4.950805289	5.41282E-08	0
GB52992	-3.796249157	7.181362483	4.49039E-07	FBgn0052354
GB46310	-3.835528529	6.489982557	1.19374E-10	FBgn0000551
GB42286	-3.858578986	5.518490935	2.27746E-10	FBgn0037122
GB50450	-3.867915962	8.997644356	1.36737E-08	FBgn0001250
GB55895	-3.870528692	5.783159697	3.21261E-11	FBgn0040705
GB52824	-3.878157771	5.170711189	4.37973E-08	FBgn0033869
GB40771	-3.88630937	5.0328827	9.39491E-09	FBgn0033763
GB44777	-3.89578203	8.477311239	3.59566E-11	FBgn0032299
GB40944	-3.901497806	7.178151963	1.15084E-11	FBgn0034903
GB45938	-3.904959886	4.789473503	3.00944E-07	FBgn0000075
GB53579	-3.91558267	5.187899448	7.20211E-08	FBgn0051148
GB42891	-3.961347935	6.769932348	2.16693E-10	FBgn0024366
GB46819	-3.986463376	6.058862269	7.16887E-11	FBgn0051296
GB46415	-4.010960389	4.434713722	0.000201628	FBgn0035077
GB41792	-4.012031807	8.395892572	6.22006E-12	FBgn0040601
GB49646	-4.040246107	6.444546277	2.38575E-11	FBgn0038511
GB50610	-4.049362512	10.03049605	1.6804E-13	FBgn0050101
GB40604	-4.062927958	4.992019164	9.91544E-05	FBgn0261526
GB45732	-4.063791099	7.587378151	1.76722E-12	FBgn0036486
GB41946	-4.069295233	7.849953515	1.43542E-12	FBgn0022770
GB43580	-4.091344853	9.408747739	1.9951E-13	FBgn0039126
GB50439	-4.098978607	8.406700205	5.67136E-11	FBgn0039480
GB42673	-4.103109096	6.957250843	4.90245E-12	FBgn0032405
GB53119	-4.126544575	8.831506432	1.87617E-07	0
GB48443	-4.130035222	6.861972986	5.42117E-12	FBgn0029922
GB53286	-4.13438557	7.81025151	6.29063E-12	FBgn0262111
GB49400	-4.145030599	5.58078813	1.25781E-10	FBgn0262599
GB52186	-4.153454364	5.428034172	1.41736E-10	FBgn0052808
GB42580	-4.15418035	5.244899895	3.68615E-10	FBgn0261446
GB51214	-4.170157562	8.405894142	5.41912E-11	FBgn0004169
GB40607	-4.179249243	4.260449342	5.82225E-05	FBgn0037556
GB49802	-4.202158053	4.749772019	1.3083E-07	FBgn0086906
GB47947	-4.245021994	6.529766459	1.15084E-11	FBgn0013988
GB50766	-4.262300145	6.377480743	3.56512E-12	FBgn0264357
GB42704	-4.280772976	6.922574134	8.58973E-12	FBgn0037290
GB42911	-4.285194278	4.268906387	0.00087741	FBgn0053299
GB41015	-4.29524533	10.42041963	7.72374E-15	FBgn0031957
GB42909	-4.296998382	4.994634481	3.94549E-06	FBgn0039027
GB51653	-4.323983691	9.949695784	1.4993E-12	FBgn0264695
GB45654	-4.345898806	5.587065567	9.49577E-12	FBgn0025837
GB50062	-4.384342501	5.203318366	1.53764E-10	FBgn0037665
GB51652	-4.393546032	7.247760648	3.59566E-11	FBgn0264695

GB55396	-4.398977568	7.027490692	6.39544E-13	FBgn0052694
GB51698	-4.39920812	12.49495934	4.79488E-15	FBgn0002564
GB50650	-4.417668728	5.699561089	4.7011E-07	FBgn0033359
GB55611	-4.426610754	4.511146388	2.6136E-06	FBgn0010399
GB48216	-4.447949682	5.685230752	8.71233E-13	FBgn0031692
GB42358	-4.457991824	6.545332714	2.43123E-13	FBgn0087011
GB53986	-4.459900338	4.598200596	2.24146E-07	FBgn0026268
GB53516	-4.468713195	7.707142401	1.07679E-13	FBgn0043792
GB51787	-4.486097058	8.461177666	6.13982E-14	FBgn0002772
GB42596	-4.524388257	6.974676099	1.56677E-09	FBgn0021742
GB50572	-4.553841331	6.395377682	8.92134E-11	FBgn0040279
GB42912	-4.559433535	6.270345053	1.04528E-09	FBgn0053196
GB43298	-4.561352493	8.24852293	8.19303E-13	FBgn0030539
GB46386	-4.578311889	4.740112494	5.47883E-09	FBgn0050420
GB50636	-4.578376089	8.330225653	5.63584E-15	FBgn0031097
GB41771	-4.580349204	5.770766114	1.3016E-12	FBgn0024897
GB49268	-4.595960309	4.263065388	2.10088E-07	FBgn0038837
GB51407	-4.597501477	6.054029553	1.34093E-10	FBgn0050069
GB48769	-4.60087107	4.659875332	6.06324E-07	FBgn0032362
GB48474	-4.622925475	9.074181379	4.32468E-16	FBgn0035398
GB42533	-4.640865751	5.416941161	9.49577E-12	FBgn0040233
GB50828	-4.659221767	6.714720104	6.17191E-14	FBgn0261836
GB40228	-4.667228424	5.465484348	4.19976E-13	0
GB54456	-4.669655165	5.199546846	6.29063E-12	FBgn0032462
GB41383	-4.691567102	4.181475379	3.40442E-05	FBgn0035788
GB40837	-4.702765761	8.994391224	2.03617E-15	FBgn0037323
GB40240	-4.772412934	9.755302011	1.36199E-14	FBgn0002773
GB48979	-4.781229937	4.403630132	1.62527E-07	FBgn0037323
GB48860	-4.793674967	5.219316263	6.61086E-10	FBgn0038819
GB52105	-4.814539406	5.68213681	1.38158E-08	FBgn0051807
GB41904	-4.840540973	6.299983432	3.16656E-14	FBgn0030884
GB43181	-4.849804332	6.828745911	5.41282E-08	FBgn0037992
GB48161	-4.853143579	8.665731234	9.71311E-17	FBgn0034391
GB49795	-4.915246456	6.903317118	2.66225E-10	FBgn0029681
GB50612	-4.945392296	4.305814953	0.000604461	FBgn0050101
GB53524	-5.009408596	7.01736633	6.64325E-15	FBgn0043792
GB47550	-5.020961529	4.591849725	2.2754E-08	FBgn0052537
GB55100	-5.037654095	5.678492502	5.31068E-11	FBgn0003016
GB45860	-5.042394798	5.891888242	7.28891E-10	FBgn0066365
GB49772	-5.093231582	6.13242056	3.59607E-10	FBgn0029681
GB50827	-5.09371907	7.432592339	9.95696E-16	FBgn0051205
GB48173	-5.126409464	7.024740572	8.87691E-16	FBgn0033158
GB48861	-5.126434021	6.372146309	1.45394E-13	FBgn0038819
GB42763	-5.15219759	7.047287941	8.85323E-16	FBgn0023388
GB53288	-5.16117053	4.572146997	5.82817E-06	FBgn0040211
GB53517	-5.168778212	6.053449995	6.8703E-14	FBgn0035935
GB44561	-5.202946852	5.848810631	3.33097E-13	FBgn0039075
GB48107	-5.226869736	6.61848971	1.36199E-14	FBgn0017561
GB53110	-5.231349229	12.8707348	1.33521E-19	FBgn0034289
GB43769	-5.251332107	6.005617828	3.82098E-05	FBgn0037411
GB53120	-5.258607944	6.117281959	1.34192E-13	FBgn0013733
GB54963	-5.288952108	4.702027602	7.38335E-07	FBgn0039335
GB45764	-5.323483863	7.304549464	2.4212E-17	FBgn0004117
GB40447	-5.331158072	7.553781991	3.63496E-12	FBgn0033869
GB51441	-5.383502436	10.89502157	5.0489E-21	FBgn0019960
GB50562	-5.387185933	7.405980626	3.93468E-06	FBgn0037411

GB53935	-5.413069086	4.043393827	5.36496E-06	FBgn0011476
GB50613	-5.419681068	10.49255556	1.33789E-16	FBgn0050101
GB53112	-5.428345938	5.766254309	1.12752E-13	FBgn0034289
GB42910	-5.43731013	4.757646001	2.35868E-05	FBgn0053299
GB52052	-5.438315129	7.392983209	1.40832E-14	FBgn0029690
GB45850	-5.446486661	9.096473943	4.85148E-15	FBgn0035636
GB48174	-5.4485501	8.723268326	6.85682E-17	FBgn0033158
GB50875	-5.463818656	6.820607706	3.05615E-14	FBgn0053126
GB42702	-5.477908322	9.248887724	1.1941E-20	FBgn0037288
GB47752	-5.515043291	6.534164177	3.71505E-14	FBgn0001992
GB46973	-5.518363451	7.574785907	4.34143E-18	FBgn0263038
GB49021	-5.562206042	9.777492894	6.36414E-20	FBgn0034030
GB54489	-5.566229543	5.111492264	7.3639E-13	FBgn0052829
GB54791	-5.578623085	4.279545686	8.17152E-08	FBgn0003089
GB47118	-5.617979587	8.317954969	8.76706E-20	FBgn0038439
GB49689	-5.65175419	7.604236631	4.76932E-19	FBgn0004577
GB50442	-5.658512342	5.205383155	4.54202E-14	FBgn0039480
GB40659	-5.669542012	4.378644984	9.43215E-09	FBgn0043043
GB50449	-5.674011106	6.858139047	1.14124E-15	0
GB47946	-5.67929931	4.43118507	1.35079E-09	FBgn0013988
GB42597	-5.737129889	12.09356337	5.44984E-23	FBgn0032538
GB46591	-5.741803772	11.43232851	2.67613E-22	FBgn0032538
GB42888	-5.776241908	9.32207576	4.66981E-22	FBgn0053196
GB46585	-5.800148373	11.8424213	4.66981E-22	FBgn0032538
GB50451	-5.80726974	10.68343613	4.35406E-20	FBgn0053196
GB55614	-5.839313135	7.015867608	1.19816E-17	FBgn0011225
GB45957	-5.85113678	7.454507235	2.85306E-18	FBgn0034157
GB40624	-5.85765917	8.143762399	5.35733E-21	FBgn0259247
GB46518	-5.85800219	8.335852717	3.44452E-10	0
GB49394	-5.891907014	7.725687819	4.35406E-20	FBgn0259247
GB53113	-5.896334073	7.610868771	2.71541E-17	FBgn0040496
GB42582	-5.906404172	6.694968304	1.06213E-16	FBgn0035280
GB45763	-5.914022382	6.449705193	8.04592E-15	FBgn0004117
GB44399	-5.917663422	10.72414426	7.91223E-20	FBgn0050101
GB54970	-6.122837235	9.406342098	2.67613E-22	FBgn0035089
GB52104	-6.169279346	4.76782576	1.71935E-10	FBgn0039479
GB53525	-6.171543806	6.045173189	1.69069E-15	FBgn0035641
GB43877	-6.297896662	5.631954329	1.8211E-12	FBgn0034883
GB55612	-6.317779852	7.895072307	1.00707E-21	FBgn0263216
GB47903	-6.423742269	7.171670631	6.32601E-20	FBgn0036110
GB50876	-6.622986705	5.058142695	2.34858E-09	FBgn0053126
GB46399	-6.662085923	7.774255526	7.67769E-20	FBgn0032256
GB48794	-6.752198095	10.88586947	2.33167E-27	FBgn0004034
GB46394	-6.868363921	7.050956778	2.43504E-21	FBgn0035091
GB51819	-7.807856572	3.518344203	1.34466E-05	FBgn0030590
GB46417	-7.977066023	3.476835302	0.000211095	FBgn0037662
GB50611	-8.024187021	3.469908441	0.000697375	FBgn0050101
GB50887	-8.142800669	3.856928975	1.7978E-07	FBgn0029961
GB47927	-8.265531204	3.861301851	2.36401E-06	0
GB50577	-8.426190415	3.915174307	4.89528E-06	FBgn0037427
GB42581	-8.449393087	4.11138689	1.37911E-08	FBgn0035873
GB42643	-8.622691746	3.998422497	0.00012058	FBgn0039200
