# Supplementary material for: The Iflaviruses Sacbrood virus and Deformed wing virus evoke different transcriptional responses in the honeybee which may facilitate their horizontal or vertical transmission
Source: PeerJ. 2016 Jan 18;4:e1591. doi: 10.7717/peerj.1591 (PMC4727977; doi:10.7717/peerj.1591)
Supplement: Table S3 [file peerj-04-1591-s003.pdf]

Table S3
Larval feeding NGS experiments, Gene Ontology (GO) terms associated with the differentially expressed (DE) genes in Contrasts 1 to 5 and the commonality between Contrasts 2, 4, and 5. For each contrast and the commonality, GO terms associated with all DE genes, upregulated DE genes, and downregualted DE genes are shown.

**Contrast 1**<br>**High SBV+DWV and high DWV vs. control**
None

**Contrast 2**<br>**High SBV+DWV vs. high DWV and control**<br>**All DE genes**				
**Biological Process**				
**GO Term**	**Aspect**	**P-value**	**Sample frequency**	**Background frequency**
GO:0042335 cuticle development	P	7.68E-13	48/764 (6.3%)	234/14580 (1.6%)
GO:0055002 striated muscle cell development	P	1.31E-09	20/764 (2.6%)	51/14580 (0.3%)
GO:0055001 muscle cell development	P	1.31E-09	20/764 (2.6%)	51/14580 (0.3%)
GO:0040003 chitin-based cuticle development	P	3.11E-09	37/764 (4.8%)	182/14580 (1.2%)
GO:0030239 myofibril assembly	P	3.46E-09	17/764 (2.2%)	37/14580 (0.3%)
GO:0007275 multicellular organismal development	P	1.95E-08	265/764 (34.7%)	3495/14580 (24.0%)
GO:0044707 single-multicellular organism process	P	2.35E-08	304/764 (39.8%)	4170/14580 (28.6%)
GO:0065008 regulation of biological quality	P	3.70E-08	105/764 (13.7%)	1018/14580 (7.0%)
GO:0006040 amino sugar metabolic process	P	1.32E-07	29/764 (3.8%)	132/14580 (0.9%)
GO:0032502 developmental process	P	1.91E-07	303/764 (39.7%)	4222/14580 (29.0%)
GO:0044699 single-organism process	P	1.96E-07	507/764 (66.4%)	8031/14580 (55.1%)
GO:0006022 aminoglycan metabolic process	P	4.24E-07	30/764 (3.9%)	147/14580 (1.0%)
GO:0006030 chitin metabolic process	P	5.29E-07	27/764 (3.5%)	122/14580 (0.8%)
GO:1901071 glucosamine-containing compound metabolic pro	P	5.73E-07	28/764 (3.7%)	131/14580 (0.9%)
GO:0031032 actomyosin structure organization	P	1.06E-06	20/764 (2.6%)	70/14580 (0.5%)
GO:0044767 single-organism developmental process	P	1.15E-06	297/764 (38.9%)	4179/14580 (28.7%)
GO:1901564 organonitrogen compound metabolic process	P	2.02E-06	81/764 (10.6%)	757/14580 (5.2%)
GO:0055114 oxidation-reduction process	P	2.56E-06	65/764 (8.5%)	552/14580 (3.8%)
GO:0048856 anatomical structure development	P	1.13E-05	273/764 (35.7%)	3842/14580 (26.4%)
GO:0051146 striated muscle cell differentiation	P	1.37E-05	22/764 (2.9%)	96/14580 (0.7%)
GO:0065007 biological regulation	P	2.05E-05	259/764 (33.9%)	3621/14580 (24.8%)
GO:0030036 actin cytoskeleton organization	P	3.98E-05	36/764 (4.7%)	239/14580 (1.6%)
GO:0061061 muscle structure development	P	4.97E-05	38/764 (5.0%)	263/14580 (1.8%)
GO:0030029 actin filament-based process	P	6.20E-05	37/764 (4.8%)	254/14580 (1.7%)
GO:0045214 sarcomere organization	P	1.17E-04	11/764 (1.4%)	26/14580 (0.2%)
GO:0044710 single-organism metabolic process	P	1.29E-04	161/764 (21.1%)	2045/14580 (14.0%)
GO:0014070 response to organic cyclic compound	P	1.36E-04	23/764 (3.0%)	117/14580 (0.8%)
GO:0002376 immune system process	P	1.52E-04	49/764 (6.4%)	405/14580 (2.8%)
GO:0044763 single-organism cellular process	P	2.24E-04	382/764 (50.0%)	5930/14580 (40.7%)
GO:0042692 muscle cell differentiation	P	2.73E-04	22/764 (2.9%)	112/14580 (0.8%)
GO:0050896 response to stimulus	P	3.70E-04	208/764 (27.2%)	2855/14580 (19.6%)
GO:0032989 cellular component morphogenesis	P	6.11E-04	86/764 (11.3%)	931/14580 (6.4%)
GO:0003012 muscle system process	P	1.01E-03	11/764 (1.4%)	31/14580 (0.2%)
GO:0009888 tissue development	P	1.44E-03	114/764 (14.9%)	1372/14580 (9.4%)
GO:0032501 multicellular organismal process	P	1.76E-03	316/764 (41.4%)	4813/14580 (33.0%)
GO:1901615 organic hydroxy compound metabolic process	P	1.78E-03	24/764 (3.1%)	144/14580 (1.0%)
GO:0006979 response to oxidative stress	P	2.64E-03	19/764 (2.5%)	98/14580 (0.7%)
GO:0006950 response to stress	P	5.17E-03	93/764 (12.2%)	1084/14580 (7.4%)
GO:0042221 response to chemical	P	6.68E-03	87/764 (11.4%)	1000/14580 (6.9%)
GO:1901135 carbohydrate derivative metabolic process	P	6.72E-03	52/764 (6.8%)	499/14580 (3.4%)
GO:0009791 post-embryonic development	P	6.75E-03	69/764 (9.0%)	736/14580 (5.0%)
GO:0018958 phenol-containing compound metabolic process	P	7.71E-03	16/764 (2.1%)	77/14580 (0.5%)
GO:0010033 response to organic substance	P	9.81E-03	42/764 (5.5%)	373/14580 (2.6%)
**Cellular Component**				
**GO Term**	**Aspect**	**P-value**	**Sample frequency**	**Background frequency**
GO:0044449 contractile fiber part	C	2.73E-15	24/764 (3.1%)	47/14580 (0.3%)
GO:0043292 contractile fiber	C	5.19E-15	24/764 (3.1%)	48/14580 (0.3%)
GO:0030016 myofibril	C	2.74E-14	23/764 (3.0%)	46/14580 (0.3%)
GO:0030017 sarcomere	C	1.42E-12	21/764 (2.7%)	43/14580 (0.3%)
GO:0015629 actin cytoskeleton	C	3.05E-09	28/764 (3.7%)	107/14580 (0.7%)
GO:0005576 extracellular region	C	1.29E-08	100/764 (13.1%)	933/14580 (6.4%)
GO:0044459 plasma membrane part	C	2.30E-08	69/764 (9.0%)	543/14580 (3.7%)
GO:0016020 membrane	C	2.80E-08	204/764 (26.7%)	2500/14580 (17.1%)
GO:0071944 cell periphery	C	6.62E-08	104/764 (13.6%)	1014/14580 (7.0%)
GO:0016459 myosin complex	C	8.70E-08	14/764 (1.8%)	28/14580 (0.2%)

GO:0005886 plasma membrane	C	2.21E-07	94/764 (12.3%)	897/14580 (6.2%)
GO:0031224 intrinsic component of membrane	C	4.44E-07	128/764 (16.8%)	1393/14580 (9.6%)
GO:0031226 intrinsic component of plasma membrane	C	1.05E-06	42/764 (5.5%)	272/14580 (1.9%)
GO:0036379 myofilament	C	1.94E-06	8/764 (1.0%)	9/14580 (0.1%)
GO:0044425 membrane part	C	2.35E-06	154/764 (20.2%)	1825/14580 (12.5%)
GO:0031012 extracellular matrix	C	4.25E-06	34/764 (4.5%)	200/14580 (1.4%)
GO:0016021 integral component of membrane	C	6.25E-06	123/764 (16.1%)	1376/14580 (9.4%)
GO:0005887 integral component of plasma membrane	C	3.61E-05	38/764 (5.0%)	260/14580 (1.8%)
GO:0005859 muscle myosin complex	C	8.36E-05	6/764 (0.8%)	6/14580 (0.0%)
GO:0030018 Z disc	C	6.98E-03	10/764 (1.3%)	30/14580 (0.2%)
GO:0031674 I band	C	6.98E-03	10/764 (1.3%)	30/14580 (0.2%)
GO:0045177 apical part of cell	C	9.19E-03	19/764 (2.5%)	106/14580 (0.7%)
GO:0005865 striated muscle thin filament	C	9.21E-03	5/764 (0.7%)	6/14580 (0.0%)

Molecular Function

GO Term	Aspect	P-value	Sample frequency	Background frequency
GO:0016491 oxidoreductase activity	F	2.74E-09	79/764 (10.3%)	640/14580 (4.4%)
GO:0043167 ion binding	F	9.75E-09	195/764 (25.5%)	2330/14580 (16.0%)
GO:0003824 catalytic activity	F	3.10E-07	298/764 (39.0%)	4151/14580 (28.5%)
GO:0005488 binding	F	5.27E-07	360/764 (47.1%)	5276/14580 (36.2%)
GO:0005509 calcium ion binding	F	1.41E-06	35/764 (4.6%)	202/14580 (1.4%)
GO:0008061 chitin binding	F	5.17E-06	24/764 (3.1%)	108/14580 (0.7%)
GO:0097367 carbohydrate derivative binding	F	9.68E-06	94/764 (12.3%)	963/14580 (6.6%)
GO:0042302 structural constituent of cuticle	F	5.53E-05	27/764 (3.5%)	149/14580 (1.0%)
GO:0003779 actin binding	F	6.74E-05	24/764 (3.1%)	122/14580 (0.8%)
GO:0046872 metal ion binding	F	1.07E-04	118/764 (15.4%)	1367/14580 (9.4%)
GO:0005214 structural constituent of chitin-based cuticle	F	1.11E-04	26/764 (3.4%)	144/14580 (1.0%)
GO:0043169 cation binding	F	2.61E-04	118/764 (15.4%)	1389/14580 (9.5%)
GO:0016918 retinal binding	F	1.60E-03	5/764 (0.7%)	5/14580 (0.0%)
GO:0005515 protein binding	F	1.74E-03	141/764 (18.5%)	1803/14580 (12.4%)
GO:0008010 structural constituent of chitin-based larval cuticle	F	3.39E-03	20/764 (2.6%)	109/14580 (0.7%)
GO:0008092 cytoskeletal protein binding	F	3.90E-03	36/764 (4.7%)	286/14580 (2.0%)

Contrast 2
High SBV+DWV vs. high DWV and control
Upregulated DE genes

Biological Process

GO Term	Aspect	P-value	Sample frequency	Background frequency
GO:0006950 response to stress	P	2.51E-10	68/346 (19.7%)	1084/14580 (7.4%)
GO:0044699 single-organism process	P	7.82E-10	256/346 (74.0%)	8052/14580 (55.2%)
GO:0050896 response to stimulus	P	8.78E-10	125/346 (36.1%)	2855/14580 (19.6%)
GO:0044763 single-organism cellular process	P	1.19E-08	204/346 (59.0%)	5939/14580 (40.7%)
GO:0002376 immune system process	P	0.00000012	35/346 (10.1%)	405/14580 (2.8%)
GO:0044710 single-organism metabolic process	P	7.31E-07	96/346 (27.7%)	2155/14580 (14.8%)
GO:0006952 defense response	P	0.00000105	32/346 (9.2%)	373/14580 (2.6%)
GO:0065007 biological regulation	P	0.00000449	136/346 (39.3%)	3623/14580 (24.8%)
GO:0055114 oxidation-reduction process	P	0.0000547	37/346 (10.7%)	559/14580 (3.8%)
GO:0006955 immune response	P	0.000152	25/346 (7.2%)	298/14580 (2.0%)
GO:0007568 aging	P	0.000204	18/346 (5.2%)	163/14580 (1.1%)
GO:0044707 single-multicellular organism process	P	0.00035	144/346 (41.6%)	4170/14580 (28.6%)
GO:0045087 innate immune response	P	0.000635	17/346 (4.9%)	157/14580 (1.1%)
GO:0006582 melanin metabolic process	P	0.000644	11/346 (3.2%)	62/14580 (0.4%)
GO:0019748 secondary metabolic process	P	0.000746	14/346 (4.0%)	107/14580 (0.7%)
GO:0010259 multicellular organismal aging	P	0.000763	17/346 (4.9%)	159/14580 (1.1%)
GO:0018958 phenol-containing compound metabolic process	P	0.00079	12/346 (3.5%)	77/14580 (0.5%)
GO:1901615 organic hydroxy compound metabolic process	P	0.00102	16/346 (4.6%)	144/14580 (1.0%)
GO:0051179 localization	P	0.00146	86/346 (24.9%)	2155/14580 (14.8%)
GO:0009611 response to wounding	P	0.00172	13/346 (3.8%)	98/14580 (0.7%)
GO:0007275 multicellular organismal development	P	0.00215	123/346 (35.5%)	3495/14580 (24.0%)
GO:0008340 determination of adult lifespan	P	0.00304	16/346 (4.6%)	156/14580 (1.1%)
GO:0009987 cellular process	P	0.00309	231/346 (66.8%)	7896/14580 (54.2%)
GO:1901700 response to oxygen-containing compound	P	0.00361	20/346 (5.8%)	239/14580 (1.6%)
GO:0043603 cellular amide metabolic process	P	0.0042	14/346 (4.0%)	123/14580 (0.8%)
GO:0032502 developmental process	P	0.0044	141/346 (40.8%)	4222/14580 (29.0%)
GO:0006629 lipid metabolic process	P	0.0044	26/346 (7.5%)	380/14580 (2.6%)
GO:0050789 regulation of biological process	P	0.00514	117/346 (33.8%)	3328/14580 (22.8%)
GO:0055072 iron ion homeostasis	P	0.00544	5/346 (1.4%)	10/14580 (0.1%)
GO:0043604 amide biosynthetic process	P	0.00786	10/346 (2.9%)	64/14580 (0.4%)
GO:0002682 regulation of immune system process	P	0.00812	17/346 (4.9%)	188/14580 (1.3%)

Cellular Component

GO Term	Aspect	P-value	Sample frequency	Background frequency
GO:0005576 extracellular region	C	0.0000587	51/346 (14.7%)	933/14580 (6.4%)
GO:0005811 lipid particle	C	0.00222	19/346 (5.5%)	211/14580 (1.4%)

Molecular Function

GO Term	Aspect	P-value	Sample frequency	Background frequency
GO:0003824 catalytic activity	F	6.42E-07	153/346 (44.2%)	4151/14580 (28.5%)
GO:0016491 oxidoreductase activity	F	0.000205	39/346 (11.3%)	640/14580 (4.4%)
GO:0043167 ion binding	F	0.00148	91/346 (26.3%)	2327/14580 (16.0%)
GO:0005539 glycosaminoglycan binding	F	0.0032	8/346 (2.3%)	34/14580 (0.2%)
GO:0042834 peptidoglycan binding	F	0.00813	6/346 (1.7%)	18/14580 (0.1%)

Contrast 2
High SBV+DWV vs. high DWV and control
Downregulated DE genes

Biological Process

GO Term	Aspect	P-value	Sample frequency	Background frequency
GO:0042335 cuticle development	P	6.84E-15	39/442 (8.8%)	234/14580 (1.6%)
GO:0030239 myofibril assembly	P	8.38E-12	16/442 (3.6%)	37/14580 (0.3%)
GO:0055002 striated muscle cell development	P	1.11E-11	18/442 (4.1%)	51/14580 (0.3%)
GO:0055001 muscle cell development	P	1.11E-11	18/442 (4.1%)	51/14580 (0.3%)
GO:0040003 chitin-based cuticle development	P	1.26E-11	31/442 (7.0%)	182/14580 (1.2%)
GO:0006040 amino sugar metabolic process	P	2.89E-08	23/442 (5.2%)	132/14580 (0.9%)
GO:0006030 chitin metabolic process	P	4.05E-08	22/442 (5.0%)	122/14580 (0.8%)
GO:0031032 actomyosin structure organization	P	6.13E-08	17/442 (3.8%)	70/14580 (0.5%)
GO:1901071 glucosamine-containing compound metabolic process	P	1.75E-07	22/442 (5.0%)	131/14580 (0.9%)
GO:0051146 striated muscle cell differentiation	P	0.00000154	18/442 (4.1%)	96/14580 (0.7%)
GO:0006022 aminoglycan metabolic process	P	0.00000175	22/442 (5.0%)	147/14580 (1.0%)
GO:0045214 sarcomere organization	P	0.00000611	10/442 (2.3%)	26/14580 (0.2%)
GO:0042692 muscle cell differentiation	P	0.0000205	18/442 (4.1%)	112/14580 (0.8%)
GO:0007275 multicellular organismal development	P	0.000111	156/442 (35.3%)	3495/14580 (24.0%)
GO:0061061 muscle structure development	P	0.000366	26/442 (5.9%)	263/14580 (1.8%)
GO:0048856 anatomical structure development	P	0.000536	165/442 (37.3%)	3842/14580 (26.4%)
GO:0030029 actin filament-based process	P	0.000698	25/442 (5.7%)	254/14580 (1.7%)
GO:0065008 regulation of biological quality	P	0.000741	61/442 (13.8%)	1022/14580 (7.0%)
GO:0032502 developmental process	P	0.000773	177/442 (40.0%)	4222/14580 (29.0%)
GO:0030036 actin cytoskeleton organization	P	0.000842	24/442 (5.4%)	239/14580 (1.6%)
GO:0044707 single-multicellular organism process	P	0.000894	175/442 (39.6%)	4170/14580 (28.6%)
GO:0044767 single-organism developmental process	P	0.00175	174/442 (39.4%)	4179/14580 (28.7%)
GO:0003012 muscle system process	P	0.00858	8/442 (1.8%)	31/14580 (0.2%)
GO:0044331 cell-cell adhesion mediated by cadherin	P	0.00893	6/442 (1.4%)	15/14580 (0.1%)
GO:0042438 melanin biosynthetic process	P	0.00893	6/442 (1.4%)	15/14580 (0.1%)
GO:0032989 cellular component morphogenesis	P	0.0093	54/442 (12.2%)	931/14580 (6.4%)

Cellular Component

GO Term	Aspect	P-value	Sample frequency	Background frequency
GO:0044449 contractile fiber part	C	7.4E-10	16/442 (3.6%)	47/14580 (0.3%)
GO:0043292 contractile fiber	C	1.08E-09	16/442 (3.6%)	48/14580 (0.3%)
GO:0015629 actin cytoskeleton	C	2.54E-09	22/442 (5.0%)	107/14580 (0.7%)
GO:0030016 myofibril	C	8.61E-09	15/442 (3.4%)	46/14580 (0.3%)
GO:0030017 sarcomere	C	4.75E-08	14/442 (3.2%)	43/14580 (0.3%)
GO:0016459 myosin complex	C	7.11E-07	11/442 (2.5%)	28/14580 (0.2%)
GO:0031012 extracellular matrix	C	0.00000113	26/442 (5.9%)	200/14580 (1.4%)
GO:0005886 plasma membrane	C	0.0000161	60/442 (13.6%)	897/14580 (6.2%)
GO:0071944 cell periphery	C	0.0000185	65/442 (14.7%)	1014/14580 (7.0%)
GO:0005576 extracellular region	C	0.0000685	60/442 (13.6%)	933/14580 (6.4%)
GO:0036379 myofilament	C	0.000175	6/442 (1.4%)	9/14580 (0.1%)
GO:0044459 plasma membrane part	C	0.000212	41/442 (9.3%)	543/14580 (3.7%)
GO:0005859 muscle myosin complex	C	0.00044	5/442 (1.1%)	6/14580 (0.0%)
GO:0016020 membrane	C	0.00117	117/442 (26.5%)	2500/14580 (17.1%)
GO:0044425 membrane part	C	0.00129	92/442 (20.8%)	1825/14580 (12.5%)
GO:0031226 intrinsic component of plasma membrane	C	0.00254	25/442 (5.7%)	272/14580 (1.9%)
GO:0005887 integral component of plasma membrane	C	0.0039	24/442 (5.4%)	260/14580 (1.8%)

Molecular Function				
**GO Term**	**Aspect**	**P-value**	**Sample frequency**	**Background frequency**
GO:0005509 calcium ion binding	F	8.82E-09	29/442 (6.6%)	202/14580 (1.4%)
GO:0008061 chitin binding	F	2.11E-07	20/442 (4.5%)	108/14580 (0.7%)
GO:0042302 structural constituent of cuticle	F	0.00000227	22/442 (5.0%)	149/14580 (1.0%)
GO:0005214 structural constituent of chitin-based cuticle	F	0.000007	21/442 (4.8%)	144/14580 (1.0%)
GO:0003779 actin binding	F	0.0000134	19/442 (4.3%)	122/14580 (0.8%)
GO:0008010 structural constituent of chitin-based larval cuticle	F	0.0000857	17/442 (3.8%)	109/14580 (0.7%)
GO:0005198 structural molecule activity	F	0.00034	44/442 (10.0%)	616/14580 (4.2%)
GO:0008092 cytoskeletal protein binding	F	0.000536	27/442 (6.1%)	286/14580 (2.0%)
GO:0043167 ion binding	F	0.00184	110/442 (24.9%)	2327/14580 (16.0%)
GO:0016491 oxidoreductase activity	F	0.00627	42/442 (9.5%)	640/14580 (4.4%)
GO:0005488 binding	F	0.00953	206/442 (46.6%)	5275/14580 (36.2%)

Contrast 3<br>High DWV vs. control<br>All DE genes				
**GO Term**	**Aspect**	**P-value**	**Sample frequency**	**Background frequency**
GO:0006412 translation	P	4.27E-05	31/188 (16.5%)	787/14580 (5.4%)
GO:0044237 cellular metabolic process	P	5.50E-05	96/188 (51.1%)	4646/14580 (31.9%)
GO:0046128 purine ribonucleoside metabolic process	P	4.62E-04	12/188 (6.4%)	140/14580 (1.0%)
GO:0042278 purine nucleoside metabolic process	P	4.62E-04	12/188 (6.4%)	140/14580 (1.0%)
GO:0032543 mitochondrial translation	P	6.40E-04	6/188 (3.2%)	23/14580 (0.2%)
GO:0009119 ribonucleoside metabolic process	P	9.77E-04	12/188 (6.4%)	150/14580 (1.0%)
GO:0009161 ribonucleoside monophosphate metabolic process	P	1.33E-03	11/188 (5.9%)	127/14580 (0.9%)
GO:0009123 nucleoside monophosphate metabolic process	P	1.44E-03	11/188 (5.9%)	128/14580 (0.9%)
GO:0071704 organic substance metabolic process	P	1.53E-03	101/188 (53.7%)	5310/14580 (36.4%)
GO:0009116 nucleoside metabolic process	P	1.95E-03	12/188 (6.4%)	160/14580 (1.1%)
GO:0046034 ATP metabolic process	P	2.33E-03	10/188 (5.3%)	108/14580 (0.7%)
GO:1901657 glycosyl compound metabolic process	P	2.53E-03	12/188 (6.4%)	164/14580 (1.1%)
GO:0009205 purine ribonucleoside triphosphate metabolic proc	P	3.25E-03	10/188 (5.3%)	112/14580 (0.8%)
GO:0009144 purine nucleoside triphosphate metabolic process	P	3.25E-03	10/188 (5.3%)	112/14580 (0.8%)
GO:0009987 cellular process	P	3.39E-03	133/188 (70.7%)	7885/14580 (54.1%)
GO:0010467 gene expression	P	3.73E-03	54/188 (28.7%)	2242/14580 (15.4%)
GO:0009199 ribonucleoside triphosphate metabolic process	P	3.83E-03	10/188 (5.3%)	114/14580 (0.8%)
GO:0009141 nucleoside triphosphate metabolic process	P	4.49E-03	10/188 (5.3%)	116/14580 (0.8%)
GO:0006753 nucleoside phosphate metabolic process	P	5.26E-03	14/188 (7.4%)	241/14580 (1.7%)
GO:0009167 purine ribonucleoside monophosphate metabolic p	P	6.58E-03	10/188 (5.3%)	121/14580 (0.8%)
GO:0009126 purine nucleoside monophosphate metabolic proc	P	6.58E-03	10/188 (5.3%)	121/14580 (0.8%)
GO:0044249 cellular biosynthetic process	P	7.68E-03	56/188 (29.8%)	2418/14580 (16.6%)
GO:1901576 organic substance biosynthetic process	P	9.93E-03	56/188 (29.8%)	2438/14580 (16.7%)

Cellular Component				
**GO Term**	**Aspect**	**P-value**	**Sample frequency**	**Background frequency**
GO:0032991 macromolecular complex	C	5.16E-24	103/188 (54.8%)	2838/14580 (19.5%)
GO:0005761 mitochondrial ribosome	C	1.55E-23	24/188 (12.8%)	80/14580 (0.5%)
GO:0000313 organellar ribosome	C	1.55E-23	24/188 (12.8%)	80/14580 (0.5%)
GO:0044429 mitochondrial part	C	1.55E-22	42/188 (22.3%)	419/14580 (2.9%)
GO:0044446 intracellular organelle part	C	8.04E-19	90/188 (47.9%)	2544/14580 (17.4%)
GO:0044422 organelle part	C	2.30E-18	90/188 (47.9%)	2582/14580 (17.7%)
GO:0044391 ribosomal subunit	C	2.37E-18	27/188 (14.4%)	176/14580 (1.2%)
GO:0005759 mitochondrial matrix	C	3.06E-18	26/188 (13.8%)	160/14580 (1.1%)
GO:0005739 mitochondrion	C	6.81E-18	45/188 (23.9%)	644/14580 (4.4%)
GO:0030529 ribonucleoprotein complex	C	1.79E-16	43/188 (22.9%)	633/14580 (4.3%)
GO:0005840 ribosome	C	6.11E-16	30/188 (16.0%)	283/14580 (1.9%)
GO:0005622 intracellular	C	3.25E-12	125/188 (66.5%)	5545/14580 (38.0%)
GO:0000314 organellar small ribosomal subunit	C	4.80E-12	12/188 (6.4%)	32/14580 (0.2%)
GO:0005763 mitochondrial small ribosomal subunit	C	4.80E-12	12/188 (6.4%)	32/14580 (0.2%)
GO:0044444 cytoplasmic part	C	1.34E-11	76/188 (40.4%)	2439/14580 (16.7%)
GO:0070013 intracellular organelle lumen	C	1.73E-11	41/188 (21.8%)	788/14580 (5.4%)
GO:0043233 organelle lumen	C	1.73E-11	41/188 (21.8%)	788/14580 (5.4%)
GO:0031974 membrane-enclosed lumen	C	2.54E-11	41/188 (21.8%)	797/14580 (5.5%)
GO:0044424 intracellular part	C	2.75E-11	122/188 (64.9%)	5457/14580 (37.4%)
GO:0043231 intracellular membrane-bounded organelle	C	4.73E-11	94/188 (50.0%)	3569/14580 (24.5%)
GO:0043227 membrane-bounded organelle	C	5.19E-11	94/188 (50.0%)	3574/14580 (24.5%)
GO:0043229 intracellular organelle	C	6.02E-11	106/188 (56.4%)	4369/14580 (30.0%)
GO:0015934 large ribosomal subunit	C	6.45E-11	17/188 (9.0%)	108/14580 (0.7%)
GO:0043226 organelle	C	1.47E-10	106/188 (56.4%)	4423/14580 (30.3%)

GO:0005737 cytoplasm	C	9.63E-10	84/188 (44.7%)	3114/14580 (21.4%)
GO:0005623 cell	C	1.40E-09	130/188 (69.1%)	6340/14580 (43.5%)
GO:0044464 cell part	C	1.40E-09	130/188 (69.1%)	6340/14580 (43.5%)
GO:0005762 mitochondrial large ribosomal subunit	C	4.17E-08	11/188 (5.9%)	49/14580 (0.3%)
GO:0000315 organellar large ribosomal subunit	C	4.17E-08	11/188 (5.9%)	49/14580 (0.3%)
GO:0043234 protein complex	C	1.86E-07	63/188 (33.5%)	2169/14580 (14.9%)
GO:0015935 small ribosomal subunit	C	2.91E-07	12/188 (6.4%)	74/14580 (0.5%)
GO:0005743 mitochondrial inner membrane	C	6.54E-06	16/188 (8.5%)	192/14580 (1.3%)
GO:0044455 mitochondrial membrane part	C	1.06E-05	14/188 (7.4%)	146/14580 (1.0%)
GO:0019866 organelle inner membrane	C	1.27E-05	16/188 (8.5%)	201/14580 (1.4%)
GO:0043228 non-membrane-bounded organelle	C	4.64E-05	49/188 (26.1%)	1685/14580 (11.6%)
GO:0043232 intracellular non-membrane-bounded organelle	C	4.64E-05	49/188 (26.1%)	1685/14580 (11.6%)
GO:0031966 mitochondrial membrane	C	3.37E-04	16/188 (8.5%)	254/14580 (1.7%)
GO:0031090 organelle membrane	C	3.63E-04	23/188 (12.2%)	514/14580 (3.5%)
GO:0005740 mitochondrial envelope	C	5.41E-04	16/188 (8.5%)	263/14580 (1.8%)

**Molecular Function**				
**GO Term**	**Aspect**	**P-value**	**Sample frequency**	**Background frequency**
GO:0003735 structural constituent of ribosome	F	2.16E-15	29/188 (15.4%)	272/14580 (1.9%)
GO:0005198 structural molecule activity	F	1.17E-07	31/188 (16.5%)	616/14580 (4.2%)

**Contrast 3<br>High DWV vs. control<br>Upregulated DE genes**				
**Biological Process**				
**GO Term**	**Aspect**	**P-value**	**Sample frequency**	**Background frequency**
GO:0006412 translation	P	3.31E-05	31/186 (16.7%)	787/14580 (5.4%)
GO:0044237 cellular metabolic process	P	1.87E-04	96/186 (51.6%)	4811/14580 (33.0%)
GO:0009161 ribonucleoside monophosphate metabolic process	P	2.16E-04	12/186 (6.5%)	132/14580 (0.9%)
GO:0009123 nucleoside monophosphate metabolic process	P	2.35E-04	12/186 (6.5%)	133/14580 (0.9%)
GO:0046034 ATP metabolic process	P	3.67E-04	11/186 (5.9%)	113/14580 (0.8%)
GO:0032543 mitochondrial translation	P	6.05E-04	6/186 (3.2%)	23/14580 (0.2%)
GO:0009167 purine ribonucleoside monophosphate metabolic process	P	1.11E-03	11/186 (5.9%)	126/14580 (0.9%)
GO:0009126 purine nucleoside monophosphate metabolic process	P	1.11E-03	11/186 (5.9%)	126/14580 (0.9%)
GO:0010467 gene expression	P	4.08E-03	56/186 (30.1%)	2397/14580 (16.4%)
GO:0044249 cellular biosynthetic process	P	5.38E-03	56/186 (30.1%)	2418/14580 (16.6%)
GO:0009987 cellular process	P	6.55E-03	131/186 (70.4%)	7896/14580 (54.2%)
GO:1901576 organic substance biosynthetic process	P	6.88E-03	56/186 (30.1%)	2437/14580 (16.7%)
GO:0071704 organic substance metabolic process	P	8.31E-03	100/186 (53.8%)	5478/14580 (37.6%)
GO:0046907 intracellular transport	P	9.97E-03	21/186 (11.3%)	540/14580 (3.7%)
**Cellular Component**				
**GO Term**	**Aspect**	**P-value**	**Sample frequency**	**Background frequency**
GO:0032991 macromolecular complex	C	1.60E-24	103/186 (55.4%)	2838/14580 (19.5%)
GO:0005761 mitochondrial ribosome	C	1.20E-23	24/186 (12.9%)	80/14580 (0.5%)
GO:0000313 organellar ribosome	C	1.20E-23	24/186 (12.9%)	80/14580 (0.5%)
GO:0044429 mitochondrial part	C	9.91E-23	42/186 (22.6%)	419/14580 (2.9%)
GO:0044446 intracellular organelle part	C	3.16E-19	90/186 (48.4%)	2544/14580 (17.4%)
GO:0044422 organelle part	C	9.11E-19	90/186 (48.4%)	2582/14580 (17.7%)
GO:0044391 ribosomal subunit	C	1.78E-18	27/186 (14.5%)	176/14580 (1.2%)
GO:0005759 mitochondrial matrix	C	2.33E-18	26/186 (14.0%)	160/14580 (1.1%)
GO:0005739 mitochondrion	C	4.30E-18	45/186 (24.2%)	644/14580 (4.4%)
GO:0030529 ribonucleoprotein complex	C	1.16E-16	43/186 (23.1%)	633/14580 (4.3%)
GO:0005840 ribosome	C	4.49E-16	30/186 (16.1%)	283/14580 (1.9%)
GO:0005622 intracellular	C	9.22E-13	125/186 (67.2%)	5545/14580 (38.0%)
GO:0000314 organellar small ribosomal subunit	C	4.24E-12	12/186 (6.5%)	32/14580 (0.2%)
GO:0005763 mitochondrial small ribosomal subunit	C	4.24E-12	12/186 (6.5%)	32/14580 (0.2%)
GO:0044444 cytoplasmic part	C	6.77E-12	76/186 (40.9%)	2439/14580 (16.7%)
GO:0044424 intracellular part	C	8.42E-12	122/186 (65.6%)	5457/14580 (37.4%)
GO:0070013 intracellular organelle lumen	C	1.18E-11	41/186 (22.0%)	788/14580 (5.4%)
GO:0043233 organelle lumen	C	1.18E-11	41/186 (22.0%)	788/14580 (5.4%)
GO:0031974 membrane-enclosed lumen	C	1.73E-11	41/186 (22.0%)	797/14580 (5.5%)
GO:0043231 intracellular membrane-bounded organelle	C	2.04E-11	94/186 (50.5%)	3569/14580 (24.5%)

GO:0043227 membrane-bounded organelle	C	2.24E-11	94/186 (50.5%)	3574/14580 (24.5%)
GO:0043229 intracellular organelle	C	2.28E-11	106/186 (57.0%)	4369/14580 (30.0%)
GO:0015934 large ribosomal subunit	C	5.43E-11	17/186 (9.1%)	108/14580 (0.7%)
GO:0043226 organelle	C	5.62E-11	106/186 (57.0%)	4423/14580 (30.3%)
GO:0005623 cell	C	4.04E-10	130/186 (69.9%)	6340/14580 (43.5%)
GO:0044464 cell part	C	4.04E-10	130/186 (69.9%)	6340/14580 (43.5%)
GO:0005737 cytoplasm	C	4.69E-10	84/186 (45.2%)	3114/14580 (21.4%)
GO:0005762 mitochondrial large ribosomal subunit	C	3.73E-08	11/186 (5.9%)	49/14580 (0.3%)
GO:0000315 organellar large ribosomal subunit	C	3.73E-08	11/186 (5.9%)	49/14580 (0.3%)
GO:0043234 protein complex	C	1.12E-07	63/186 (33.9%)	2169/14580 (14.9%)
GO:0015935 small ribosomal subunit	C	2.59E-07	12/186 (6.5%)	74/14580 (0.5%)
GO:0005743 mitochondrial inner membrane	C	5.63E-06	16/186 (8.6%)	192/14580 (1.3%)
GO:0044455 mitochondrial membrane part	C	9.27E-06	14/186 (7.5%)	146/14580 (1.0%)
GO:0019866 organelle inner membrane	C	1.09E-05	16/186 (8.6%)	201/14580 (1.4%)
GO:0043228 non-membrane-bounded organelle	C	3.22E-05	49/186 (26.3%)	1685/14580 (11.6%)
GO:0043232 intracellular non-membrane-bounded organelle	C	3.22E-05	49/186 (26.3%)	1685/14580 (11.6%)
GO:0031966 mitochondrial membrane	C	2.93E-04	16/186 (8.6%)	254/14580 (1.7%)
GO:0031090 organelle membrane	C	3.00E-04	23/186 (12.4%)	514/14580 (3.5%)
GO:0005740 mitochondrial envelope	C	4.70E-04	16/186 (8.6%)	263/14580 (1.8%)

Molecular Function

GO Term	Aspect	P-value	Sample frequency	Background frequency
GO:0003735 structural constituent of ribosome	F	1.60E-15	29/186 (15.6%)	272/14580 (1.9%)
GO:0005198 structural molecule activity	F	8.87E-08	31/186 (16.7%)	616/14580 (4.2%)

Contrast 3. "DWV" vs "Control"
High DWV vs. control
Downregulated DE genes

None

Contrast 4.
High SBV+DWV vs. control
All DE genes

GO Term	Aspect	P-value	Sample frequency	Background frequency
GO:0044699 single-organism process	P	9.42E-20	819/1194 (68.6%)	8031/14580 (55.1%)
GO:0044763 single-organism cellular process	P	5.51E-18	642/1194 (53.8%)	5930/14580 (40.7%)
GO:0065007 biological regulation	P	6.17E-18	438/1194 (36.7%)	3621/14580 (24.8%)
GO:0007275 multicellular organismal development	P	1.77E-15	417/1194 (34.9%)	3495/14580 (24.0%)
GO:0044707 single-multicellular organism process	P	1.37E-14	475/1194 (39.8%)	4170/14580 (28.6%)
GO:0065008 regulation of biological quality	P	2.20E-14	164/1194 (13.7%)	1018/14580 (7.0%)
GO:0032502 developmental process	P	1.44E-12	471/1194 (39.4%)	4222/14580 (29.0%)
GO:0050789 regulation of biological process	P	2.22E-12	389/1194 (32.6%)	3328/14580 (22.8%)
GO:0009791 post-embryonic development	P	2.31E-12	126/1194 (10.6%)	736/14580 (5.0%)
GO:0042335 cuticle development	P	2.79E-12	60/1194 (5.0%)	234/14580 (1.6%)
GO:0050896 response to stimulus	P	3.36E-12	344/1194 (28.8%)	2855/14580 (19.6%)
GO:0044710 single-organism metabolic process	P	4.49E-12	265/1194 (22.2%)	2045/14580 (14.0%)
GO:0055002 striated muscle cell development	P	6.99E-12	26/1194 (2.2%)	51/14580 (0.3%)
GO:0055001 muscle cell development	P	6.99E-12	26/1194 (2.2%)	51/14580 (0.3%)
GO:0044767 single-organism developmental process	P	7.37E-12	464/1194 (38.9%)	4179/14580 (28.7%)
GO:0051179 localization	P	8.68E-11	271/1194 (22.7%)	2155/14580 (14.8%)
GO:0030239 myofibril assembly	P	2.23E-10	21/1194 (1.8%)	37/14580 (0.3%)
GO:0007154 cell communication	P	2.30E-10	233/1194 (19.5%)	1788/14580 (12.3%)
GO:0002165 instar larval or pupal development	P	2.36E-10	116/1194 (9.7%)	695/14580 (4.8%)
GO:0048856 anatomical structure development	P	3.64E-10	426/1194 (35.7%)	3842/14580 (26.4%)
GO:0050794 regulation of cellular process	P	1.76E-09	354/1194 (29.6%)	3087/14580 (21.2%)
GO:0009888 tissue development	P	2.27E-09	187/1194 (15.7%)	1372/14580 (9.4%)
GO:0007552 metamorphosis	P	3.16E-09	104/1194 (8.7%)	618/14580 (4.2%)
GO:0044700 single organism signaling	P	5.39E-09	224/1194 (18.8%)	1750/14580 (12.0%)
GO:0023052 signaling	P	5.39E-09	224/1194 (18.8%)	1750/14580 (12.0%)
GO:0048731 system development	P	5.63E-09	318/1194 (26.6%)	2725/14580 (18.7%)
GO:0009653 anatomical structure morphogenesis	P	8.58E-09	264/1194 (22.1%)	2166/14580 (14.9%)
GO:0040003 chitin-based cuticle development	P	1.27E-08	46/1194 (3.9%)	182/14580 (1.2%)
GO:0032989 cellular component morphogenesis	P	2.11E-08	137/1194 (11.5%)	931/14580 (6.4%)
GO:0048569 post-embryonic organ development	P	2.28E-08	93/1194 (7.8%)	544/14580 (3.7%)
GO:0048878 chemical homeostasis	P	3.07E-08	35/1194 (2.9%)	117/14580 (0.8%)
GO:0048707 instar larval or pupal morphogenesis	P	4.11E-08	98/1194 (8.2%)	592/14580 (4.1%)
GO:0048513 organ development	P	5.13E-08	206/1194 (17.3%)	1608/14580 (11.0%)
GO:0051146 striated muscle cell differentiation	P	5.41E-08	31/1194 (2.6%)	96/14580 (0.7%)
GO:0009886 post-embryonic morphogenesis	P	5.79E-08	99/1194 (8.3%)	604/14580 (4.1%)

GO:0030036 actin cytoskeleton organization	P	6.84E-08	53/1194 (4.4%)	239/14580 (1.6%)
GO:0007444 imaginal disc development	P	1.73E-07	110/1194 (9.2%)	712/14580 (4.9%)
GO:1901564 organonitrogen compound metabolic process	P	1.77E-07	115/1194 (9.6%)	757/14580 (5.2%)
GO:0030029 actin filament-based process	P	2.44E-07	54/1194 (4.5%)	254/14580 (1.7%)
GO:0032501 multicellular organismal process	P	3.68E-07	496/1194 (41.5%)	4813/14580 (33.0%)
GO:0009987 cellular process	P	3.96E-07	751/1194 (62.9%)	7885/14580 (54.1%)
GO:0051234 establishment of localization	P	4.45E-07	214/1194 (17.9%)	1727/14580 (11.8%)
GO:0035295 tube development	P	6.92E-07	128/1194 (10.7%)	893/14580 (6.1%)
GO:0050793 regulation of developmental process	P	9.33E-07	103/1194 (8.6%)	669/14580 (4.6%)
GO:0048563 post-embryonic organ morphogenesis	P	1.31E-06	84/1194 (7.0%)	507/14580 (3.5%)
GO:0007560 imaginal disc morphogenesis	P	1.31E-06	84/1194 (7.0%)	507/14580 (3.5%)
GO:0007165 signal transduction	P	1.43E-06	176/1194 (14.7%)	1366/14580 (9.4%)
GO:0006810 transport	P	1.49E-06	205/1194 (17.2%)	1658/14580 (11.4%)
GO:0031032 actomyosin structure organization	P	2.86E-06	24/1194 (2.0%)	70/14580 (0.5%)
GO:0061061 muscle structure development	P	2.91E-06	53/1194 (4.4%)	263/14580 (1.8%)
GO:0030182 neuron differentiation	P	3.25E-06	122/1194 (10.2%)	858/14580 (5.9%)
GO:0042692 muscle cell differentiation	P	4.23E-06	31/1194 (2.6%)	112/14580 (0.8%)
GO:1902578 single-organism localization	P	5.08E-06	198/1194 (16.6%)	1610/14580 (11.0%)
GO:0010033 response to organic substance	P	7.55E-06	66/1194 (5.5%)	373/14580 (2.6%)
GO:0006022 aminoglycan metabolic process	P	7.70E-06	36/1194 (3.0%)	147/14580 (1.0%)
GO:0060429 epithelium development	P	8.08E-06	155/1194 (13.0%)	1188/14580 (8.1%)
GO:0055114 oxidation-reduction process	P	8.12E-06	87/1194 (7.3%)	552/14580 (3.8%)
GO:0042221 response to chemical	P	1.20E-05	135/1194 (11.3%)	1000/14580 (6.9%)
GO:0042592 homeostatic process	P	1.21E-05	54/1194 (4.5%)	281/14580 (1.9%)
GO:0006629 lipid metabolic process	P	1.65E-05	66/1194 (5.5%)	380/14580 (2.6%)
GO:0051716 cellular response to stimulus	P	1.86E-05	210/1194 (17.6%)	1759/14580 (12.1%)
GO:0048699 generation of neurons	P	1.90E-05	128/1194 (10.7%)	940/14580 (6.4%)
GO:0006040 amino sugar metabolic process	P	2.10E-05	33/1194 (2.8%)	132/14580 (0.9%)
GO:0035239 tube morphogenesis	P	2.22E-05	97/1194 (8.1%)	653/14580 (4.5%)
GO:0060562 epithelial tube morphogenesis	P	2.25E-05	93/1194 (7.8%)	617/14580 (4.2%)
GO:0051239 regulation of multicellular organismal process	P	2.44E-05	93/1194 (7.8%)	618/14580 (4.2%)
GO:0044281 small molecule metabolic process	P	2.46E-05	107/1194 (9.0%)	746/14580 (5.1%)
GO:0008152 metabolic process	P	2.58E-05	583/1194 (48.8%)	5968/14580 (40.9%)
GO:0006950 response to stress	P	3.30E-05	142/1194 (11.9%)	1084/14580 (7.4%)
GO:0035220 wing disc development	P	3.34E-05	81/1194 (6.8%)	515/14580 (3.5%)
GO:0044765 single-organism transport	P	3.50E-05	180/1194 (15.1%)	1464/14580 (10.0%)
GO:0006030 chitin metabolic process	P	4.06E-05	31/1194 (2.6%)	122/14580 (0.8%)
GO:0014070 response to organic cyclic compound	P	5.62E-05	30/1194 (2.5%)	117/14580 (0.8%)
GO:0006979 response to oxidative stress	P	5.73E-05	27/1194 (2.3%)	98/14580 (0.7%)
GO:1901071 glucosamine-containing compound metabolic pro	P	6.61E-05	32/1194 (2.7%)	131/14580 (0.9%)
GO:0048468 cell development	P	7.33E-05	205/1194 (17.2%)	1736/14580 (11.9%)
GO:0002376 immune system process	P	9.42E-05	67/1194 (5.6%)	405/14580 (2.8%)
GO:0007015 actin filament organization	P	1.22E-04	33/1194 (2.8%)	141/14580 (1.0%)
GO:0045214 sarcomere organization	P	1.29E-04	13/1194 (1.1%)	26/14580 (0.2%)
GO:0048729 tissue morphogenesis	P	1.34E-04	108/1194 (9.0%)	779/14580 (5.3%)
GO:0002009 morphogenesis of an epithelium	P	1.63E-04	106/1194 (8.9%)	763/14580 (5.2%)
GO:0044255 cellular lipid metabolic process	P	3.03E-04	48/1194 (4.0%)	259/14580 (1.8%)
GO:0048666 neuron development	P	3.41E-04	102/1194 (8.5%)	736/14580 (5.0%)
GO:0048583 regulation of response to stimulus	P	3.74E-04	119/1194 (10.0%)	900/14580 (6.2%)
GO:1901700 response to oxygen-containing compound	P	5.49E-04	45/1194 (3.8%)	240/14580 (1.6%)
GO:1901615 organic hydroxy compound metabolic process	P	7.21E-04	32/1194 (2.7%)	144/14580 (1.0%)
GO:0009719 response to endogenous stimulus	P	9.66E-04	36/1194 (3.0%)	175/14580 (1.2%)
GO:0070887 cellular response to chemical stimulus	P	1.02E-03	46/1194 (3.9%)	253/14580 (1.7%)
GO:0009887 organ morphogenesis	P	1.21E-03	136/1194 (11.4%)	1088/14580 (7.5%)
GO:0048522 positive regulation of cellular process	P	1.36E-03	123/1194 (10.3%)	961/14580 (6.6%)
GO:0000902 cell morphogenesis	P	1.46E-03	107/1194 (9.0%)	806/14580 (5.5%)
GO:0030030 cell projection organization	P	1.48E-03	100/1194 (8.4%)	739/14580 (5.1%)
GO:0048518 positive regulation of biological process	P	1.50E-03	137/1194 (11.5%)	1102/14580 (7.6%)
GO:0007399 nervous system development	P	1.55E-03	195/1194 (16.3%)	1700/14580 (11.7%)
GO:0030154 cell differentiation	P	1.64E-03	271/1194 (22.7%)	2519/14580 (17.3%)
GO:0003012 muscle system process	P	1.73E-03	13/1194 (1.1%)	31/14580 (0.2%)
GO:0009605 response to external stimulus	P	1.80E-03	116/1194 (9.7%)	897/14580 (6.2%)
GO:0048869 cellular developmental process	P	1.83E-03	279/1194 (23.4%)	2610/14580 (17.9%)
GO:0006082 organic acid metabolic process	P	1.89E-03	60/1194 (5.0%)	376/14580 (2.6%)
GO:0043436 oxoacid metabolic process	P	1.89E-03	60/1194 (5.0%)	376/14580 (2.6%)
GO:0000904 cell morphogenesis involved in differentiation	P	2.04E-03	80/1194 (6.7%)	556/14580 (3.8%)
GO:0019752 carboxylic acid metabolic process	P	2.13E-03	58/1194 (4.9%)	360/14580 (2.5%)
GO:0007472 wing disc morphogenesis	P	2.18E-03	62/1194 (5.2%)	395/14580 (2.7%)
GO:0050801 ion homeostasis	P	2.26E-03	21/1194 (1.8%)	76/14580 (0.5%)
GO:0035120 post-embryonic appendage morphogenesis	P	2.35E-03	67/1194 (5.6%)	440/14580 (3.0%)

GO:0035114 imaginal disc-derived appendage morphogenesis	P	2.36E-03	68/1194 (5.7%)	449/14580 (3.1%)
GO:0007476 imaginal disc-derived wing morphogenesis	P	2.79E-03	61/1194 (5.1%)	389/14580 (2.7%)
GO:0035107 appendage morphogenesis	P	3.03E-03	68/1194 (5.7%)	452/14580 (3.1%)
GO:0055080 cation homeostasis	P	3.06E-03	19/1194 (1.6%)	65/14580 (0.4%)
GO:0048737 imaginal disc-derived appendage development	P	3.29E-03	68/1194 (5.7%)	453/14580 (3.1%)
GO:0048736 appendage development	P	4.19E-03	68/1194 (5.7%)	456/14580 (3.1%)
GO:0048667 cell morphogenesis involved in neuron differentia	P	4.35E-03	74/1194 (6.2%)	511/14580 (3.5%)
GO:0007568 aging	P	4.51E-03	33/1194 (2.8%)	163/14580 (1.1%)
GO:0044712 single-organism catabolic process	P	6.13E-03	49/1194 (4.1%)	294/14580 (2.0%)
GO:0055088 lipid homeostasis	P	6.17E-03	13/1194 (1.1%)	34/14580 (0.2%)
GO:0007623 circadian rhythm	P	7.36E-03	26/1194 (2.2%)	115/14580 (0.8%)
GO:0010259 multicellular organismal aging	P	7.53E-03	32/1194 (2.7%)	159/14580 (1.1%)
GO:0071310 cellular response to organic substance	P	7.55E-03	35/1194 (2.9%)	182/14580 (1.2%)
GO:0055082 cellular chemical homeostasis	P	8.69E-03	18/1194 (1.5%)	63/14580 (0.4%)

Cellular Component

GO Term	Aspect	P-value	Sample frequency	Background frequency
GO:0016020 membrane	C	7.62E-17	327/1194 (27.4%)	2500/14580 (17.1%)
GO:0044449 contractile fiber part	C	2.04E-15	28/1194 (2.3%)	47/14580 (0.3%)
GO:0043292 contractile fiber	C	4.52E-15	28/1194 (2.3%)	48/14580 (0.3%)
GO:0071944 cell periphery	C	1.49E-14	164/1194 (13.7%)	1014/14580 (7.0%)
GO:0030016 myofibril	C	1.52E-14	27/1194 (2.3%)	46/14580 (0.3%)
GO:0005886 plasma membrane	C	5.72E-14	149/1194 (12.5%)	897/14580 (6.2%)
GO:0030017 sarcomere	C	3.75E-13	25/1194 (2.1%)	43/14580 (0.3%)
GO:0015629 actin cytoskeleton	C	7.48E-12	38/1194 (3.2%)	107/14580 (0.7%)
GO:0044425 membrane part	C	2.93E-11	240/1194 (20.1%)	1825/14580 (12.5%)
GO:0044459 plasma membrane part	C	7.24E-11	99/1194 (8.3%)	543/14580 (3.7%)
GO:0031224 intrinsic component of membrane	C	8.90E-11	194/1194 (16.2%)	1393/14580 (9.6%)
GO:0016021 integral component of membrane	C	1.54E-09	188/1194 (15.7%)	1376/14580 (9.4%)
GO:0016459 myosin complex	C	1.32E-08	17/1194 (1.4%)	28/14580 (0.2%)
GO:0045177 apical part of cell	C	1.88E-07	32/1194 (2.7%)	106/14580 (0.7%)
GO:0005623 cell	C	6.13E-07	624/1194 (52.3%)	6340/14580 (43.5%)
GO:0044464 cell part	C	6.13E-07	624/1194 (52.3%)	6340/14580 (43.5%)
GO:0031226 intrinsic component of plasma membrane	C	3.55E-06	54/1194 (4.5%)	272/14580 (1.9%)
GO:0098590 plasma membrane region	C	2.05E-05	25/1194 (2.1%)	82/14580 (0.6%)
GO:0016324 apical plasma membrane	C	3.64E-05	19/1194 (1.6%)	51/14580 (0.3%)
GO:0036379 myofilament	C	8.05E-05	8/1194 (0.7%)	9/14580 (0.1%)
GO:0005887 integral component of plasma membrane	C	1.27E-04	49/1194 (4.1%)	260/14580 (1.8%)
GO:0005576 extracellular region	C	2.62E-04	123/1194 (10.3%)	933/14580 (6.4%)
GO:0030018 Z disc	C	1.09E-03	13/1194 (1.1%)	30/14580 (0.2%)
GO:0031674 I band	C	1.09E-03	13/1194 (1.1%)	30/14580 (0.2%)
GO:0031012 extracellular matrix	C	1.33E-03	39/1194 (3.3%)	200/14580 (1.4%)
GO:0005859 muscle myosin complex	C	1.45E-03	6/1194 (0.5%)	6/14580 (0.0%)
GO:0005856 cytoskeleton	C	3.65E-03	86/1194 (7.2%)	620/14580 (4.3%)
GO:0031672 A band	C	5.28E-03	9/1194 (0.8%)	16/14580 (0.1%)

Molecular Function

GO Term	Aspect	P-value	Sample frequency	Background frequency
GO:0043167 ion binding	F	1.65E-16	309/1194 (25.9%)	2330/14580 (16.0%)
GO:0003824 catalytic activity	F	2.81E-14	472/1194 (39.5%)	4151/14580 (28.5%)
GO:0005488 binding	F	4.46E-12	561/1194 (47.0%)	5276/14580 (36.2%)
GO:0005509 calcium ion binding	F	1.21E-11	54/1194 (4.5%)	202/14580 (1.4%)
GO:0016491 oxidoreductase activity	F	3.73E-10	109/1194 (9.1%)	640/14580 (4.4%)
GO:0046872 metal ion binding	F	1.14E-08	184/1194 (15.4%)	1367/14580 (9.4%)
GO:0043169 cation binding	F	4.72E-08	184/1194 (15.4%)	1389/14580 (9.5%)
GO:0003779 actin binding	F	5.31E-07	34/1194 (2.8%)	122/14580 (0.8%)
GO:0005515 protein binding	F	9.67E-07	220/1194 (18.4%)	1803/14580 (12.4%)
GO:0097367 carbohydrate derivative binding	F	6.90E-06	132/1194 (11.1%)	963/14580 (6.6%)
GO:0008061 chitin binding	F	1.33E-04	28/1194 (2.3%)	108/14580 (0.7%)
GO:0005215 transporter activity	F	2.58E-04	119/1194 (10.0%)	894/14580 (6.1%)
GO:0043168 anion binding	F	5.62E-04	134/1194 (11.2%)	1054/14580 (7.2%)
GO:0008289 lipid binding	F	3.90E-03	33/1194 (2.8%)	162/14580 (1.1%)
GO:0042302 structural constituent of cuticle	F	5.19E-03	31/1194 (2.6%)	149/14580 (1.0%)
GO:0003707 steroid hormone receptor activity	F	5.48E-03	10/1194 (0.8%)	20/14580 (0.1%)
GO:0008092 cytoskeletal protein binding	F	6.40E-03	48/1194 (4.0%)	286/14580 (2.0%)
GO:0022857 transmembrane transporter activity	F	7.41E-03	100/1194 (8.4%)	765/14580 (5.2%)
GO:0003674 molecular_function	F	7.48E-03	1093/1194 (91.5%)	12743/14580 (87.4%)
GO:0046873 metal ion transmembrane transporter activity	F	7.98E-03	39/1194 (3.3%)	214/14580 (1.5%)
GO:0005381 iron ion transmembrane transporter activity	F	9.45E-03	6/1194 (0.5%)	7/14580 (0.0%)

Contrast 4.
High SBV+DWV vs. control
Upregulated DE genes

Biological Process

GO Term	Aspect	P-value	Sample frequency	Background frequency
GO:0050896 response to stimulus	P	1.80E-15	191/535 (35.7%)	2855/14580 (19.6%)
GO:0006950 response to stress	P	1.12E-14	100/535 (18.7%)	1084/14580 (7.4%)
GO:0044763 single-organism cellular process	P	2.06E-13	312/535 (58.3%)	5939/14580 (40.7%)
GO:0044699 single-organism process	P	3.23E-13	387/535 (72.3%)	8052/14580 (55.2%)
GO:0044710 single-organism metabolic process	P	1.26E-11	148/535 (27.7%)	2155/14580 (14.8%)
GO:0065007 biological regulation	P	2.20E-11	213/535 (39.8%)	3623/14580 (24.8%)
GO:0002376 immune system process	P	8.67E-09	47/535 (8.8%)	405/14580 (2.8%)
GO:0051179 localization	P	3.42E-08	138/535 (25.8%)	2155/14580 (14.8%)
GO:0006952 defense response	P	9.77E-08	43/535 (8.0%)	373/14580 (2.6%)
GO:0007568 aging	P	1.70E-07	27/535 (5.0%)	163/14580 (1.1%)
GO:0009987 cellular process	P	2.49E-07	362/535 (67.7%)	7896/14580 (54.2%)
GO:0010259 multicellular organismal aging	P	5.24E-07	26/535 (4.9%)	159/14580 (1.1%)
GO:0006629 lipid metabolic process	P	6.42E-07	42/535 (7.9%)	380/14580 (2.6%)
GO:0050789 regulation of biological process	P	7.70E-07	185/535 (34.6%)	3328/14580 (22.8%)
GO:0051716 cellular response to stimulus	P	9.47E-07	115/535 (21.5%)	1759/14580 (12.1%)
GO:0009791 post-embryonic development	P	2.65E-06	62/535 (11.6%)	736/14580 (5.0%)
GO:0065008 regulation of biological quality	P	3.56E-06	77/535 (14.4%)	1022/14580 (7.0%)
GO:1901700 response to oxygen-containing compound	P	3.62E-06	31/535 (5.8%)	239/14580 (1.6%)
GO:0010033 response to organic substance	P	4.37E-06	40/535 (7.5%)	373/14580 (2.6%)
GO:0007275 multicellular organismal development	P	4.68E-06	189/535 (35.3%)	3495/14580 (24.0%)
GO:0048878 chemical homeostasis	P	5.54E-06	21/535 (3.9%)	117/14580 (0.8%)
GO:0009888 tissue development	P	5.81E-06	94/535 (17.6%)	1372/14580 (9.4%)
GO:0044707 single-multicellular organism process	P	6.80E-06	216/535 (40.4%)	4170/14580 (28.6%)
GO:0050794 regulation of cellular process	P	7.69E-06	171/535 (32.0%)	3087/14580 (21.2%)
GO:0006979 response to oxidative stress	P	8.78E-06	19/535 (3.6%)	98/14580 (0.7%)
GO:0008340 determination of adult lifespan	P	9.66E-06	24/535 (4.5%)	156/14580 (1.1%)
GO:0007552 metamorphosis	P	1.02E-05	54/535 (10.1%)	618/14580 (4.2%)
GO:0045087 innate immune response	P	1.10E-05	24/535 (4.5%)	157/14580 (1.1%)
GO:0002165 instar larval or pupal development	P	1.40E-05	58/535 (10.8%)	695/14580 (4.8%)
GO:0048707 instar larval or pupal morphogenesis	P	1.72E-05	52/535 (9.7%)	592/14580 (4.1%)
GO:0048584 positive regulation of response to stimulus	P	2.44E-05	38/535 (7.1%)	364/14580 (2.5%)
GO:0044255 cellular lipid metabolic process	P	2.59E-05	31/535 (5.8%)	259/14580 (1.8%)
GO:0009886 post-embryonic morphogenesis	P	3.40E-05	52/535 (9.7%)	604/14580 (4.1%)
GO:0007165 signal transduction	P	4.47E-05	91/535 (17.0%)	1366/14580 (9.4%)
GO:0051234 establishment of localization	P	4.68E-05	108/535 (20.2%)	1727/14580 (11.8%)
GO:0007154 cell communication	P	4.69E-05	111/535 (20.7%)	1792/14580 (12.3%)
GO:0006955 immune response	P	5.92E-05	33/535 (6.2%)	298/14580 (2.0%)
GO:0032502 developmental process	P	6.23E-05	214/535 (40.0%)	4222/14580 (29.0%)
GO:1902578 single-organism localization	P	6.81E-05	102/535 (19.1%)	1610/14580 (11.0%)
GO:0006810 transport	P	1.57E-04	103/535 (19.3%)	1658/14580 (11.4%)
GO:0044767 single-organism developmental process	P	2.02E-04	210/535 (39.3%)	4179/14580 (28.7%)
GO:0044700 single organism signaling	P	2.07E-04	107/535 (20.0%)	1754/14580 (12.0%)
GO:0023052 signaling	P	2.07E-04	107/535 (20.0%)	1754/14580 (12.0%)
GO:0007444 imaginal disc development	P	2.11E-04	56/535 (10.5%)	712/14580 (4.9%)
GO:1901615 organic hydroxy compound metabolic process	P	2.51E-04	21/535 (3.9%)	144/14580 (1.0%)
GO:0044765 single-organism transport	P	2.97E-04	93/535 (17.4%)	1464/14580 (10.0%)
GO:0048518 positive regulation of biological process	P	3.53E-04	75/535 (14.0%)	1095/14580 (7.5%)
GO:0042221 response to chemical	P	4.37E-04	70/535 (13.1%)	1000/14580 (6.9%)
GO:0048732 gland development	P	5.12E-04	26/535 (4.9%)	219/14580 (1.5%)
GO:0042592 homeostatic process	P	6.08E-04	30/535 (5.6%)	281/14580 (1.9%)
GO:0010623 developmental programmed cell death	P	6.47E-04	21/535 (3.9%)	152/14580 (1.0%)
GO:0009056 catabolic process	P	6.49E-04	60/535 (11.2%)	813/14580 (5.6%)
GO:0035295 tube development	P	6.62E-04	66/535 (12.3%)	931/14580 (6.4%)
GO:0048583 regulation of response to stimulus	P	9.31E-04	64/535 (12.0%)	900/14580 (6.2%)
GO:0055088 lipid homeostasis	P	9.32E-04	10/535 (1.9%)	34/14580 (0.2%)
GO:0006582 melanin metabolic process	P	1.17E-03	13/535 (2.4%)	62/14580 (0.4%)
GO:0007431 salivary gland development	P	1.19E-03	23/535 (4.3%)	185/14580 (1.3%)
GO:0035272 exocrine system development	P	1.19E-03	23/535 (4.3%)	185/14580 (1.3%)
GO:0019748 secondary metabolic process	P	1.29E-03	17/535 (3.2%)	107/14580 (0.7%)
GO:0008152 metabolic process	P	1.39E-03	279/535 (52.1%)	6067/14580 (41.6%)
GO:0097305 response to alcohol	P	1.42E-03	18/535 (3.4%)	120/14580 (0.8%)
GO:0008219 cell death	P	1.47E-03	33/535 (6.2%)	341/14580 (2.3%)
GO:0002682 regulation of immune system process	P	1.59E-03	23/535 (4.3%)	188/14580 (1.3%)

GO:0055072 iron ion homeostasis	P	1.70E-03	6/535 (1.1%)	10/14580 (0.1%)
GO:0048731 system development	P	1.79E-03	147/535 (27.5%)	2750/14580 (18.9%)
GO:0016265 death	P	1.92E-03	33/535 (6.2%)	345/14580 (2.4%)
GO:0009611 response to wounding	P	1.96E-03	16/535 (3.0%)	98/14580 (0.7%)
GO:0055114 oxidation-reduction process	P	2.44E-03	45/535 (8.4%)	559/14580 (3.8%)
GO:0070887 cellular response to chemical stimulus	P	2.57E-03	27/535 (5.0%)	253/14580 (1.7%)
GO:0018958 phenol-containing compound metabolic process	P	2.62E-03	14/535 (2.6%)	77/14580 (0.5%)
GO:0048569 post-embryonic organ development	P	2.87E-03	44/535 (8.2%)	544/14580 (3.7%)
GO:0035070 salivary gland histolysis	P	3.38E-03	15/535 (2.8%)	90/14580 (0.6%)
GO:0048522 positive regulation of cellular process	P	3.48E-03	66/535 (12.3%)	975/14580 (6.7%)
GO:0007435 salivary gland morphogenesis	P	3.49E-03	20/535 (3.7%)	154/14580 (1.1%)
GO:0022612 gland morphogenesis	P	3.49E-03	20/535 (3.7%)	154/14580 (1.1%)
GO:0006914 autophagy	P	3.70E-03	17/535 (3.2%)	115/14580 (0.8%)
GO:0012501 programmed cell death	P	4.08E-03	30/535 (5.6%)	307/14580 (2.1%)
GO:0042551 neuron maturation	P	4.27E-03	9/535 (1.7%)	31/14580 (0.2%)
GO:0060429 epithelium development	P	4.31E-03	76/535 (14.2%)	1188/14580 (8.1%)
GO:0016271 tissue death	P	4.47E-03	16/535 (3.0%)	104/14580 (0.7%)
GO:0007559 histolysis	P	4.47E-03	16/535 (3.0%)	104/14580 (0.7%)
GO:0021700 developmental maturation	P	5.68E-03	24/535 (4.5%)	217/14580 (1.5%)
GO:0048513 organ development	P	6.01E-03	95/535 (17.8%)	1609/14580 (11.0%)
GO:0009607 response to biotic stimulus	P	6.28E-03	31/535 (5.8%)	330/14580 (2.3%)
GO:0048563 post-embryonic organ morphogenesis	P	6.97E-03	41/535 (7.7%)	507/14580 (3.5%)
GO:0007560 imaginal disc morphogenesis	P	6.97E-03	41/535 (7.7%)	507/14580 (3.5%)
GO:0009605 response to external stimulus	P	8.10E-03	61/535 (11.4%)	897/14580 (6.2%)
GO:0043603 cellular amide metabolic process	P	9.64E-03	17/535 (3.2%)	123/14580 (0.8%)

**Cellular Component**				
**GO Term**	**Aspect**	**P-value**	**Sample frequency**	**Background frequency**
GO:0016020 membrane	C	7.23E-04	138/535 (25.8%)	2500/14580 (17.1%)
GO:0005811 lipid particle	C	9.29E-04	25/535 (4.7%)	211/14580 (1.4%)
GO:0030016 myofibril	C	2.35E-03	11/535 (2.1%)	46/14580 (0.3%)
GO:0044449 contractile fiber part	C	2.97E-03	11/535 (2.1%)	47/14580 (0.3%)
GO:0005623 cell	C	3.71E-03	287/535 (53.6%)	6340/14580 (43.5%)
GO:0044464 cell part	C	3.71E-03	287/535 (53.6%)	6340/14580 (43.5%)
GO:0043292 contractile fiber	C	3.73E-03	11/535 (2.1%)	48/14580 (0.3%)
GO:0005576 extracellular region	C	6.88E-03	63/535 (11.8%)	933/14580 (6.4%)

**Molecular Function**				
**GO Term**	**Aspect**	**P-value**	**Sample frequency**	**Background frequency**
GO:0003824 catalytic activity	F	1.05E-08	226/535 (42.2%)	4151/14580 (28.5%)
GO:0043167 ion binding	F	1.05E-06	141/535 (26.4%)	2327/14580 (16.0%)
GO:0005515 protein binding	F	2.35E-04	109/535 (20.4%)	1802/14580 (12.4%)
GO:0005488 binding	F	5.32E-04	251/535 (46.9%)	5275/14580 (36.2%)
GO:0016491 oxidoreductase activity	F	5.75E-04	51/535 (9.5%)	640/14580 (4.4%)
GO:0005381 iron ion transmembrane transporter activity	F	4.99E-03	5/535 (0.9%)	7/14580 (0.0%)
GO:0046872 metal ion binding	F	5.65E-03	84/535 (15.7%)	1367/14580 (9.4%)
GO:0008289 lipid binding	F	7.81E-03	20/535 (3.7%)	162/14580 (1.1%)

**Contrast 4**				
**High SBV+DWV vs. control**				
**Downregulated DE genes**				
**Biological Process**				
**GO Term**	**Aspect**	**P-value**	**Sample frequency**	**Background frequency**
GO:0042335 cuticle development	P	1.04E-13	47/698 (6.7%)	234/14580 (1.6%)
GO:0055002 striated muscle cell development	P	1.06E-12	22/698 (3.2%)	51/14580 (0.3%)
GO:0055001 muscle cell development	P	1.06E-12	22/698 (3.2%)	51/14580 (0.3%)
GO:0030239 myofibril assembly	P	1.96E-12	19/698 (2.7%)	37/14580 (0.3%)
GO:0040003 chitin-based cuticle development	P	1.82E-10	37/698 (5.3%)	182/14580 (1.2%)
GO:0007275 multicellular organismal development	P	1.56E-09	250/698 (35.8%)	3495/14580 (24.0%)
GO:0044707 single-multicellular organism process	P	1.80E-08	282/698 (40.4%)	4170/14580 (28.6%)
GO:0032989 cellular component morphogenesis	P	1.37E-07	91/698 (13.0%)	931/14580 (6.4%)
GO:0031032 actomyosin structure organization	P	1.97E-07	20/698 (2.9%)	70/14580 (0.5%)
GO:0048856 anatomical structure development	P	2.36E-07	260/698 (37.2%)	3842/14580 (26.4%)
GO:0032502 developmental process	P	2.42E-07	280/698 (40.1%)	4222/14580 (29.0%)
GO:0006030 chitin metabolic process	P	3.67E-07	26/698 (3.7%)	122/14580 (0.8%)
GO:0051146 striated muscle cell differentiation	P	3.67E-07	23/698 (3.3%)	96/14580 (0.7%)
GO:0006040 amino sugar metabolic process	P	4.32E-07	27/698 (3.9%)	132/14580 (0.9%)

GO:0044767 single-organism developmental process	P	6.09E-07	276/698 (39.5%)	4179/14580 (28.7%)
GO:0065008 regulation of biological quality	P	7.72E-07	95/698 (13.6%)	1022/14580 (7.0%)
GO:0009653 anatomical structure morphogenesis	P	8.46E-07	165/698 (23.6%)	2166/14580 (14.9%)
GO:0006022 aminoglycan metabolic process	P	1.13E-06	28/698 (4.0%)	147/14580 (1.0%)
GO:1901071 glucosamine-containing compound metabolic process	P	1.92E-06	26/698 (3.7%)	131/14580 (0.9%)
GO:0030036 actin cytoskeleton organization	P	3.35E-06	36/698 (5.2%)	239/14580 (1.6%)
GO:0044699 single-organism process	P	3.83E-06	462/698 (66.2%)	8052/14580 (55.2%)
GO:0030029 actin filament-based process	P	5.07E-06	37/698 (5.3%)	254/14580 (1.7%)
GO:0042692 muscle cell differentiation	P	9.57E-06	23/698 (3.3%)	112/14580 (0.8%)
GO:0065007 biological regulation	P	3.01E-05	239/698 (34.2%)	3623/14580 (24.8%)
GO:0045214 sarcomere organization	P	4.26E-05	11/698 (1.6%)	26/14580 (0.2%)
GO:0061061 muscle structure development	P	4.59E-05	36/698 (5.2%)	263/14580 (1.8%)
GO:0009791 post-embryonic development	P	9.54E-05	70/698 (10.0%)	736/14580 (5.0%)
GO:0048731 system development	P	1.27E-04	189/698 (27.1%)	2750/14580 (18.9%)
GO:0048513 organ development	P	1.40E-04	124/698 (17.8%)	1609/14580 (11.0%)
GO:0044763 single-organism cellular process	P	2.74E-04	352/698 (50.4%)	5939/14580 (40.7%)
GO:0032501 multicellular organismal process	P	3.95E-04	295/698 (42.3%)	4813/14580 (33.0%)
GO:0009888 tissue development	P	8.11E-04	107/698 (15.3%)	1372/14580 (9.4%)
GO:0030182 neuron differentiation	P	9.54E-04	75/698 (10.7%)	858/14580 (5.9%)
GO:0050789 regulation of biological process	P	2.32E-03	214/698 (30.7%)	3328/14580 (22.8%)
GO:0048569 post-embryonic organ development	P	2.48E-03	53/698 (7.6%)	544/14580 (3.7%)
GO:0002165 instar larval or pupal development	P	2.72E-03	63/698 (9.0%)	695/14580 (4.8%)
GO:0007010 cytoskeleton organization	P	2.92E-03	61/698 (8.7%)	666/14580 (4.6%)
GO:0050793 regulation of developmental process	P	3.39E-03	61/698 (8.7%)	669/14580 (4.6%)
GO:0007154 cell communication	P	3.40E-03	129/698 (18.5%)	1792/14580 (12.3%)
GO:0048699 generation of neurons	P	4.31E-03	78/698 (11.2%)	940/14580 (6.4%)
GO:0048666 neuron development	P	9.18E-03	64/698 (9.2%)	736/14580 (5.0%)
GO:0044331 cell-cell adhesion mediated by cadherin	P	9.77E-03	7/698 (1.0%)	15/14580 (0.1%)
GO:0042438 melanin biosynthetic process	P	9.77E-03	7/698 (1.0%)	15/14580 (0.1%)

Cellular Component

GO Term	Aspect	P-value	Sample frequency	Background frequency
GO:0071944 cell periphery	C	3.07E-11	106/698 (15.2%)	1014/14580 (7.0%)
GO:0005886 plasma membrane	C	6.19E-11	97/698 (13.9%)	897/14580 (6.2%)
GO:0016020 membrane	C	1.71E-09	194/698 (27.8%)	2500/14580 (17.1%)
GO:0015629 actin cytoskeleton	C	2.23E-09	27/698 (3.9%)	107/14580 (0.7%)
GO:0044459 plasma membrane part	C	2.45E-08	65/698 (9.3%)	543/14580 (3.7%)
GO:0044425 membrane part	C	5.49E-08	149/698 (21.3%)	1825/14580 (12.5%)
GO:0044449 contractile fiber part	C	8.14E-08	17/698 (2.4%)	47/14580 (0.3%)
GO:0043292 contractile fiber	C	1.20E-07	17/698 (2.4%)	48/14580 (0.3%)
GO:0016459 myosin complex	C	4.55E-07	13/698 (1.9%)	28/14580 (0.2%)
GO:0030016 myofibril	C	6.31E-07	16/698 (2.3%)	46/14580 (0.3%)
GO:0030017 sarcomere	C	2.26E-06	15/698 (2.1%)	43/14580 (0.3%)
GO:0031224 intrinsic component of membrane	C	2.68E-06	117/698 (16.8%)	1393/14580 (9.6%)
GO:0016021 integral component of membrane	C	5.18E-06	115/698 (16.5%)	1376/14580 (9.4%)
GO:0031012 extracellular matrix	C	2.49E-05	31/698 (4.4%)	200/14580 (1.4%)
GO:0045177 apical part of cell	C	9.22E-05	21/698 (3.0%)	106/14580 (0.7%)
GO:0031226 intrinsic component of plasma membrane	C	1.12E-04	36/698 (5.2%)	272/14580 (1.9%)
GO:0016324 apical plasma membrane	C	2.76E-04	14/698 (2.0%)	51/14580 (0.3%)
GO:0005887 integral component of plasma membrane	C	3.65E-04	34/698 (4.9%)	260/14580 (1.8%)
GO:0036379 myofilament	C	3.33E-03	6/698 (0.9%)	9/14580 (0.1%)
GO:0098590 plasma membrane region	C	5.29E-03	16/698 (2.3%)	82/14580 (0.6%)
GO:0005859 muscle myosin complex	C	5.43E-03	5/698 (0.7%)	6/14580 (0.0%)

Molecular Function

GO Term	Aspect	P-value	Sample frequency	Background frequency
GO:0005509 calcium ion binding	F	1.46E-12	42/698 (6.0%)	202/14580 (1.4%)
GO:0043167 ion binding	F	4.46E-08	179/698 (25.6%)	2327/14580 (16.0%)
GO:0008061 chitin binding	F	7.73E-07	24/698 (3.4%)	108/14580 (0.7%)
GO:0005488 binding	F	1.83E-06	330/698 (47.3%)	5275/14580 (36.2%)
GO:0003779 actin binding	F	2.03E-06	25/698 (3.6%)	122/14580 (0.8%)
GO:0003824 catalytic activity	F	4.26E-05	266/698 (38.1%)	4151/14580 (28.5%)
GO:0042302 structural constituent of cuticle	F	1.47E-04	25/698 (3.6%)	149/14580 (1.0%)
GO:0005214 structural constituent of chitin-based cuticle	F	3.14E-04	24/698 (3.4%)	144/14580 (1.0%)
GO:0097367 carbohydrate derivative binding	F	3.78E-04	83/698 (11.9%)	963/14580 (6.6%)
GO:0046872 metal ion binding	F	1.24E-03	106/698 (15.2%)	1367/14580 (9.4%)
GO:0016491 oxidoreductase activity	F	1.65E-03	60/698 (8.6%)	640/14580 (4.4%)
GO:0043169 cation binding	F	2.68E-03	106/698 (15.2%)	1389/14580 (9.5%)
GO:0008092 cytoskeletal protein binding	F	3.54E-03	34/698 (4.9%)	286/14580 (2.0%)

Contrast 5
High SBV+DWV vs. high DWV
All DE genes

Biological Process

GO Term	Aspect	P-value	Sample frequency	Background frequency
GO:1901564 organonitrogen compound metabolic process	P	4.01E-12	82/610 (13.4%)	757/14580 (5.2%)
GO:0042335 cuticle development	P	3.91E-10	39/610 (6.4%)	234/14580 (1.6%)
GO:0055114 oxidation-reduction process	P	1.09E-09	63/610 (10.3%)	552/14580 (3.8%)
GO:0044281 small molecule metabolic process	P	7.42E-08	72/610 (11.8%)	746/14580 (5.1%)
GO:1901135 carbohydrate derivative metabolic process	P	1.52E-07	55/610 (9.0%)	499/14580 (3.4%)
GO:0046034 ATP metabolic process	P	3.03E-07	23/610 (3.8%)	108/14580 (0.7%)
GO:0040003 chitin-based cuticle development	P	3.79E-07	30/610 (4.9%)	182/14580 (1.2%)
GO:0009126 purine nucleoside monophosphate metabolic proc	P	5.67E-07	24/610 (3.9%)	121/14580 (0.8%)
GO:0009167 purine ribonucleoside monophosphate metabolic p	P	5.67E-07	24/610 (3.9%)	121/14580 (0.8%)
GO:0009205 purine ribonucleoside triphosphate metabolic proc	P	6.58E-07	23/610 (3.8%)	112/14580 (0.8%)
GO:0009144 purine nucleoside triphosphate metabolic process	P	6.58E-07	23/610 (3.8%)	112/14580 (0.8%)
GO:0009199 ribonucleoside triphosphate metabolic process	P	9.57E-07	23/610 (3.8%)	114/14580 (0.8%)
GO:0009141 nucleoside triphosphate metabolic process	P	1.38E-06	23/610 (3.8%)	116/14580 (0.8%)
GO:0009161 ribonucleoside monophosphate metabolic process	P	1.62E-06	24/610 (3.9%)	127/14580 (0.9%)
GO:0009123 nucleoside monophosphate metabolic process	P	1.92E-06	24/610 (3.9%)	128/14580 (0.9%)
GO:0042278 purine nucleoside metabolic process	P	2.46E-06	25/610 (4.1%)	140/14580 (1.0%)
GO:0046128 purine ribonucleoside metabolic process	P	2.46E-06	25/610 (4.1%)	140/14580 (1.0%)
GO:0030239 myofibril assembly	P	5.36E-06	13/610 (2.1%)	37/14580 (0.3%)
GO:0044710 single-organism metabolic process	P	6.27E-06	139/610 (22.8%)	2045/14580 (14.0%)
GO:0022900 electron transport chain	P	8.53E-06	19/610 (3.1%)	87/14580 (0.6%)
GO:0009116 nucleoside metabolic process	P	9.22E-06	26/610 (4.3%)	160/14580 (1.1%)
GO:0009119 ribonucleoside metabolic process	P	1.09E-05	25/610 (4.1%)	150/14580 (1.0%)
GO:1901657 glycosyl compound metabolic process	P	1.58E-05	26/610 (4.3%)	164/14580 (1.1%)
GO:0022904 respiratory electron transport chain	P	1.68E-05	18/610 (3.0%)	81/14580 (0.6%)
GO:0007275 multicellular organismal development	P	1.90E-05	208/610 (34.1%)	3495/14580 (24.0%)
GO:0042773 ATP synthesis coupled electron transport	P	2.63E-05	17/610 (2.8%)	74/14580 (0.5%)
GO:0006022 aminoglycan metabolic process	P	3.44E-05	24/610 (3.9%)	147/14580 (1.0%)
GO:0055002 striated muscle cell development	P	4.80E-05	14/610 (2.3%)	51/14580 (0.3%)
GO:0055001 muscle cell development	P	4.80E-05	14/610 (2.3%)	51/14580 (0.3%)
GO:0044707 single-multicellular organism process	P	5.90E-05	237/610 (38.9%)	4170/14580 (28.6%)
GO:0042775 mitochondrial ATP synthesis coupled electron trar	P	7.86E-05	16/610 (2.6%)	70/14580 (0.5%)
GO:1901071 glucosamine-containing compound metabolic pro	P	8.40E-05	22/610 (3.6%)	131/14580 (0.9%)
GO:0006119 oxidative phosphorylation	P	9.19E-05	17/610 (2.8%)	80/14580 (0.5%)
GO:0006040 amino sugar metabolic process	P	9.69E-05	22/610 (3.6%)	132/14580 (0.9%)
GO:0006030 chitin metabolic process	P	1.12E-04	21/610 (3.4%)	122/14580 (0.8%)
GO:0044699 single-organism process	P	1.72E-04	400/610 (65.6%)	8031/14580 (55.1%)
GO:0032502 developmental process	P	1.86E-04	237/610 (38.9%)	4222/14580 (29.0%)
GO:0002376 immune system process	P	1.94E-04	42/610 (6.9%)	405/14580 (2.8%)
GO:0065008 regulation of biological quality	P	2.03E-04	79/610 (13.0%)	1018/14580 (7.0%)
GO:0008152 metabolic process	P	2.88E-04	313/610 (51.3%)	5968/14580 (40.9%)
GO:0072521 purine-containing compound metabolic process	P	4.36E-04	26/610 (4.3%)	192/14580 (1.3%)
GO:0009150 purine ribonucleotide metabolic process	P	5.41E-04	24/610 (3.9%)	169/14580 (1.2%)
GO:0031032 actomyosin structure organization	P	5.42E-04	15/610 (2.5%)	70/14580 (0.5%)
GO:0019693 ribose phosphate metabolic process	P	6.42E-04	25/610 (4.1%)	183/14580 (1.3%)
GO:0006163 purine nucleotide metabolic process	P	8.44E-04	24/610 (3.9%)	173/14580 (1.2%)
GO:0009259 ribonucleotide metabolic process	P	1.05E-03	24/610 (3.9%)	175/14580 (1.2%)
GO:0044767 single-organism developmental process	P	1.22E-03	231/610 (37.9%)	4179/14580 (28.7%)
GO:0044763 single-organism cellular process	P	1.93E-03	307/610 (50.3%)	5930/14580 (40.7%)
GO:0030036 actin cytoskeleton organization	P	3.03E-03	28/610 (4.6%)	239/14580 (1.6%)
GO:0030029 actin filament-based process	P	3.31E-03	29/610 (4.8%)	254/14580 (1.7%)
GO:0048856 anatomical structure development	P	6.22E-03	212/610 (34.8%)	3842/14580 (26.4%)
GO:0051146 striated muscle cell differentiation	P	7.64E-03	16/610 (2.6%)	96/14580 (0.7%)
GO:1901615 organic hydroxy compound metabolic process	P	8.34E-03	20/610 (3.3%)	144/14580 (1.0%)
GO:0006952 defense response	P	9.70E-03	36/610 (5.9%)	373/14580 (2.6%)
GO:0045333 cellular respiration	P	9.82E-03	18/610 (3.0%)	121/14580 (0.8%)
GO:0006950 response to stress	P	9.98E-03	77/610 (12.6%)	1084/14580 (7.4%)

Cellular Component

GO Term	Aspect	P-value	Sample frequency	Background frequency
GO:0005739 mitochondrion	C	3.88E-14	78/610 (12.8%)	644/14580 (4.4%)
GO:0044449 contractile fiber part	C	1.31E-13	21/610 (3.4%)	47/14580 (0.3%)
GO:0043292 contractile fiber	C	2.24E-13	21/610 (3.4%)	48/14580 (0.3%)
GO:0044429 mitochondrial part	C	5.25E-13	59/610 (9.7%)	419/14580 (2.9%)

GO:0030016 myofibril	C	1.44E-12	20/610 (3.3%)	46/14580 (0.3%)
GO:0030017 sarcomere	C	9.89E-11	18/610 (3.0%)	43/14580 (0.3%)
GO:0044455 mitochondrial membrane part	C	6.62E-09	29/610 (4.8%)	146/14580 (1.0%)
GO:0005746 mitochondrial respiratory chain	C	2.64E-08	22/610 (3.6%)	88/14580 (0.6%)
GO:0070469 respiratory chain	C	2.64E-08	22/610 (3.6%)	88/14580 (0.6%)
GO:0005761 mitochondrial ribosome	C	2.87E-08	21/610 (3.4%)	80/14580 (0.5%)
GO:0000313 organellar ribosome	C	2.87E-08	21/610 (3.4%)	80/14580 (0.5%)
GO:0015629 actin cytoskeleton	C	3.64E-08	24/610 (3.9%)	107/14580 (0.7%)
GO:0044444 cytoplasmic part	C	1.37E-07	165/610 (27.0%)	2439/14580 (16.7%)
GO:0044391 ribosomal subunit	C	1.60E-07	30/610 (4.9%)	176/14580 (1.2%)
GO:0005811 lipid particle	C	1.87E-07	33/610 (5.4%)	211/14580 (1.4%)
GO:0005743 mitochondrial inner membrane	C	3.17E-07	31/610 (5.1%)	192/14580 (1.3%)
GO:0005763 mitochondrial small ribosomal subunit	C	6.34E-07	13/610 (2.1%)	32/14580 (0.2%)
GO:0000314 organellar small ribosomal subunit	C	6.34E-07	13/610 (2.1%)	32/14580 (0.2%)
GO:0005576 extracellular region	C	7.55E-07	81/610 (13.3%)	933/14580 (6.4%)
GO:0019866 organelle inner membrane	C	1.04E-06	31/610 (5.1%)	201/14580 (1.4%)
GO:0005737 cytoplasm	C	3.43E-06	193/610 (31.6%)	3114/14580 (21.4%)
GO:0031012 extracellular matrix	C	4.01E-06	30/610 (4.9%)	200/14580 (1.4%)
GO:0005740 mitochondrial envelope	C	4.54E-06	35/610 (5.7%)	263/14580 (1.8%)
GO:0031966 mitochondrial membrane	C	6.73E-06	34/610 (5.6%)	254/14580 (1.7%)
GO:0043228 non-membrane-bounded organelle	C	7.37E-06	120/610 (19.7%)	1685/14580 (11.6%)
GO:0043232 intracellular non-membrane-bounded organelle	C	7.37E-06	120/610 (19.7%)	1685/14580 (11.6%)
GO:0036379 myofilament	C	2.60E-05	7/610 (1.1%)	9/14580 (0.1%)
GO:0005759 mitochondrial matrix	C	4.25E-05	25/610 (4.1%)	160/14580 (1.1%)
GO:0031967 organelle envelope	C	8.28E-05	40/610 (6.6%)	364/14580 (2.5%)
GO:0031975 envelope	C	8.94E-05	40/610 (6.6%)	365/14580 (2.5%)
GO:0005623 cell	C	1.09E-04	331/610 (54.3%)	6340/14580 (43.5%)
GO:0044464 cell part	C	1.09E-04	331/610 (54.3%)	6340/14580 (43.5%)
GO:0031226 intrinsic component of plasma membrane	C	1.37E-04	33/610 (5.4%)	272/14580 (1.9%)
GO:0016020 membrane	C	1.92E-04	156/610 (25.6%)	2500/14580 (17.1%)
GO:0044425 membrane part	C	2.18E-04	122/610 (20.0%)	1825/14580 (12.5%)
GO:0005747 mitochondrial respiratory chain complex I	C	2.49E-04	13/610 (2.1%)	49/14580 (0.3%)
GO:0030964 NADH dehydrogenase complex	C	2.49E-04	13/610 (2.1%)	49/14580 (0.3%)
GO:0045271 respiratory chain complex I	C	2.49E-04	13/610 (2.1%)	49/14580 (0.3%)
GO:0016459 myosin complex	C	3.63E-04	10/610 (1.6%)	28/14580 (0.2%)
GO:0015935 small ribosomal subunit	C	1.17E-03	15/610 (2.5%)	74/14580 (0.5%)
GO:0005887 integral component of plasma membrane	C	1.71E-03	30/610 (4.9%)	260/14580 (1.8%)
GO:0015934 large ribosomal subunit	C	1.78E-03	18/610 (3.0%)	108/14580 (0.7%)
GO:0043229 intracellular organelle	C	2.43E-03	238/610 (39.0%)	4369/14580 (30.0%)
GO:0005865 striated muscle thin filament	C	2.62E-03	5/610 (0.8%)	6/14580 (0.0%)
GO:0005859 muscle myosin complex	C	2.62E-03	5/610 (0.8%)	6/14580 (0.0%)
GO:0005840 ribosome	C	3.45E-03	31/610 (5.1%)	283/14580 (1.9%)
GO:0044422 organelle part	C	4.24E-03	154/610 (25.2%)	2582/14580 (17.7%)
GO:0043226 organelle	C	4.46E-03	239/610 (39.2%)	4423/14580 (30.3%)
GO:0044459 plasma membrane part	C	6.62E-03	47/610 (7.7%)	543/14580 (3.7%)

**Molecular Function**				
**GO Term**	**Aspect**	**P-value**	**Sample frequency**	**Background frequency**
GO:0005198 structural molecule activity	F	1.32E-13	75/610 (12.3%)	616/14580 (4.2%)
GO:0016491 oxidoreductase activity	F	3.70E-12	74/610 (12.1%)	640/14580 (4.4%)
GO:0042302 structural constituent of cuticle	F	2.06E-04	23/610 (3.8%)	149/14580 (1.0%)
GO:0005509 calcium ion binding	F	3.28E-04	27/610 (4.4%)	202/14580 (1.4%)
GO:0003824 catalytic activity	F	4.31E-04	232/610 (38.0%)	4151/14580 (28.5%)
GO:0003954 NADH dehydrogenase activity	F	4.32E-04	12/610 (2.0%)	43/14580 (0.3%)
GO:0016918 retinal binding	F	4.52E-04	5/610 (0.8%)	5/14580 (0.0%)
GO:0005214 structural constituent of chitin-based cuticle	F	4.83E-04	22/610 (3.6%)	144/14580 (1.0%)
GO:0003779 actin binding	F	5.50E-04	20/610 (3.3%)	122/14580 (0.8%)
GO:0016651 oxidoreductase activity, acting on NAD(P)H	F	1.70E-03	13/610 (2.1%)	57/14580 (0.4%)
GO:0008061 chitin binding	F	1.78E-03	18/610 (3.0%)	108/14580 (0.7%)
GO:0005539 glycosaminoglycan binding	F	2.89E-03	10/610 (1.6%)	34/14580 (0.2%)
GO:0003735 structural constituent of ribosome	F	4.47E-03	30/610 (4.9%)	272/14580 (1.9%)
GO:0008010 structural constituent of chitin-based larval cuticle	F	9.54E-03	17/610 (2.8%)	109/14580 (0.7%)

**Contrast 5**				
**High SBV+DWV vs. high DWV**				
**Upregulated DE genes**				
**Biological Process**				
**GO Term**	**Aspect**	**P-value**	**Sample frequency**	**Background frequency**

GO:0006950 response to stress	P	3.64E-11	61/280 (21.8%)	1084/14580 (7.4%)
GO:0050896 response to stimulus	P	9.78E-11	109/280 (38.9%)	2855/14580 (19.6%)
GO:0002376 immune system process	P	2.26E-10	35/280 (12.5%)	405/14580 (2.8%)
GO:0006952 defense response	P	1.85E-08	31/280 (11.1%)	373/14580 (2.6%)
GO:0044699 single-organism process	P	6.71E-08	208/280 (74.3%)	8052/14580 (55.2%)
GO:0044763 single-organism cellular process	P	6.13E-07	166/280 (59.3%)	5939/14580 (40.7%)
GO:0065007 biological regulation	P	7.95E-07	117/280 (41.8%)	3623/14580 (24.8%)
GO:0006955 immune response	P	1.89E-06	25/280 (8.9%)	298/14580 (2.0%)
GO:0009888 tissue development	P	6.66E-06	59/280 (21.1%)	1372/14580 (9.4%)
GO:0007275 multicellular organismal development	P	1.96E-05	110/280 (39.3%)	3495/14580 (24.0%)
GO:0035295 tube development	P	2.44E-05	45/280 (16.1%)	931/14580 (6.4%)
GO:0045087 innate immune response	P	2.63E-05	17/280 (6.1%)	157/14580 (1.1%)
GO:0044707 single-multicellular organism process	P	3.65E-05	124/280 (44.3%)	4170/14580 (28.6%)
GO:0032502 developmental process	P	8.03E-05	124/280 (44.3%)	4222/14580 (29.0%)
GO:0009611 response to wounding	P	1.35E-04	13/280 (4.6%)	98/14580 (0.7%)
GO:0010259 multicellular organismal aging	P	2.06E-04	16/280 (5.7%)	159/14580 (1.1%)
GO:0060429 epithelium development	P	2.84E-04	50/280 (17.9%)	1188/14580 (8.1%)
GO:0007568 aging	P	2.91E-04	16/280 (5.7%)	163/14580 (1.1%)
GO:0048513 organ development	P	3.70E-04	61/280 (21.8%)	1609/14580 (11.0%)
GO:0002682 regulation of immune system process	P	3.84E-04	17/280 (6.1%)	188/14580 (1.3%)
GO:0044767 single-organism developmental process	P	6.43E-04	120/280 (42.9%)	4179/14580 (28.7%)
GO:0044710 single-organism metabolic process	P	6.64E-04	74/280 (26.4%)	2155/14580 (14.8%)
GO:0018958 phenol-containing compound metabolic process	P	6.92E-04	11/280 (3.9%)	77/14580 (0.5%)
GO:0007444 imaginal disc development	P	9.08E-04	35/280 (12.5%)	712/14580 (4.9%)
GO:0065008 regulation of biological quality	P	1.02E-03	44/280 (15.7%)	1022/14580 (7.0%)
GO:0050789 regulation of biological process	P	1.64E-03	100/280 (35.7%)	3328/14580 (22.8%)
GO:0050776 regulation of immune response	P	2.67E-03	13/280 (4.6%)	126/14580 (0.9%)
GO:0051179 localization	P	2.96E-03	72/280 (25.7%)	2155/14580 (14.8%)
GO:0048731 system development	P	3.00E-03	86/280 (30.7%)	2750/14580 (18.9%)
GO:0006629 lipid metabolic process	P	3.64E-03	23/280 (8.2%)	380/14580 (2.6%)
GO:0002520 immune system development	P	3.67E-03	10/280 (3.6%)	73/14580 (0.5%)
GO:0048534 hematopoietic or lymphoid organ development	P	3.67E-03	10/280 (3.6%)	73/14580 (0.5%)
GO:0030097 hemopoiesis	P	3.71E-03	9/280 (3.2%)	57/14580 (0.4%)
GO:0032501 multicellular organismal process	P	5.09E-03	130/280 (46.4%)	4813/14580 (33.0%)
GO:0008340 determination of adult lifespan	P	5.51E-03	14/280 (5.0%)	156/14580 (1.1%)
GO:0048856 anatomical structure development	P	6.92E-03	109/280 (38.9%)	3842/14580 (26.4%)
GO:0006582 melanin metabolic process	P	7.69E-03	9/280 (3.2%)	62/14580 (0.4%)

Cellular Component

GO Term	Aspect	P-value	Sample frequency	Background frequency
GO:0005576 extracellular region	C	8.70E-06	46/280 (16.4%)	933/14580 (6.4%)
GO:0005604 basement membrane	C	6.08E-04	6/280 (2.1%)	15/14580 (0.1%)
GO:0005811 lipid particle	C	2.00E-03	17/280 (6.1%)	211/14580 (1.4%)
GO:0044420 extracellular matrix part	C	2.15E-03	6/280 (2.1%)	18/14580 (0.1%)

Molecular Function

GO Term	Aspect	P-value	Sample frequency	Background frequency
GO:0003824 catalytic activity	F	4.33E-04	120/280 (42.9%)	4151/14580 (28.5%)
GO:0005539 glycosaminoglycan binding	F	5.82E-04	8/280 (2.9%)	34/14580 (0.2%)
GO:0042834 peptidoglycan binding	F	2.15E-03	6/280 (2.1%)	18/14580 (0.1%)
GO:0043167 ion binding	F	3.22E-03	76/280 (27.1%)	2327/14580 (16.0%)
GO:0005515 protein binding	F	3.85E-03	63/280 (22.5%)	1802/14580 (12.4%)

Contrast 5
High SBV+DWV vs. high DWV
Downregulated DE genes

Biological Process

GO Term	Aspect	P-value	Sample frequency	Background frequency
GO:0046034 ATP metabolic process	P	3.26E-13	24/345 (7.0%)	113/14580 (0.8%)
GO:0009126 purine nucleoside monophosphate metabolic process	P	4.50E-12	24/345 (7.0%)	126/14580 (0.9%)
GO:0009167 purine ribonucleoside monophosphate metabolic process	P	4.50E-12	24/345 (7.0%)	126/14580 (0.9%)
GO:0009161 ribonucleoside monophosphate metabolic process	P	1.35E-11	24/345 (7.0%)	132/14580 (0.9%)
GO:0009123 nucleoside monophosphate metabolic process	P	1.62E-11	24/345 (7.0%)	133/14580 (0.9%)
GO:0042773 ATP synthesis coupled electron transport	P	2.46E-09	17/345 (4.9%)	74/14580 (0.5%)
GO:0030239 myofibril assembly	P	2.93E-09	13/345 (3.8%)	37/14580 (0.3%)
GO:0022900 electron transport chain	P	3.58E-09	18/345 (5.2%)	87/14580 (0.6%)

GO:0042335 cuticle development	P	3.82E-09	28/345 (8.1%)	234/14580 (1.6%)
GO:0006119 oxidative phosphorylation	P	9.55E-09	17/345 (4.9%)	80/14580 (0.5%)
GO:0022904 respiratory electron transport chain	P	1.18E-08	17/345 (4.9%)	81/14580 (0.6%)
GO:0042775 mitochondrial ATP synthesis coupled electron transport	P	1.25E-08	16/345 (4.6%)	70/14580 (0.5%)
GO:0040003 chitin-based cuticle development	P	1.96E-08	24/345 (7.0%)	182/14580 (1.2%)
GO:1901564 organonitrogen compound metabolic process	P	8.81E-08	55/345 (15.9%)	886/14580 (6.1%)
GO:0055002 striated muscle cell development	P	2.91E-07	13/345 (3.8%)	51/14580 (0.3%)
GO:0055001 muscle cell development	P	2.91E-07	13/345 (3.8%)	51/14580 (0.3%)
GO:0031032 actomyosin structure organization	P	1.83E-06	14/345 (4.1%)	70/14580 (0.5%)
GO:1901135 carbohydrate derivative metabolic process	P	6.44E-06	41/345 (11.9%)	622/14580 (4.3%)
GO:0045333 cellular respiration	P	8.61E-06	17/345 (4.9%)	121/14580 (0.8%)
GO:0006030 chitin metabolic process	P	9.80E-06	17/345 (4.9%)	122/14580 (0.8%)
GO:0009205 purine ribonucleoside triphosphate metabolic process	P	1.23E-05	24/345 (7.0%)	248/14580 (1.7%)
GO:0009144 purine nucleoside triphosphate metabolic process	P	1.23E-05	24/345 (7.0%)	248/14580 (1.7%)
GO:0009199 ribonucleoside triphosphate metabolic process	P	1.33E-05	24/345 (7.0%)	249/14580 (1.7%)
GO:0009141 nucleoside triphosphate metabolic process	P	1.56E-05	24/345 (7.0%)	251/14580 (1.7%)
GO:1901071 glucosamine-containing compound metabolic process	P	2.95E-05	17/345 (4.9%)	131/14580 (0.9%)
GO:0006040 amino sugar metabolic process	P	3.31E-05	17/345 (4.9%)	132/14580 (0.9%)
GO:0055114 oxidation-reduction process	P	3.67E-05	37/345 (10.7%)	559/14580 (3.8%)
GO:0015980 energy derivation by oxidation of organic compounds	P	4.16E-05	17/345 (4.9%)	134/14580 (0.9%)
GO:0042278 purine nucleoside metabolic process	P	9.89E-05	24/345 (7.0%)	276/14580 (1.9%)
GO:0046128 purine ribonucleoside metabolic process	P	9.89E-05	24/345 (7.0%)	276/14580 (1.9%)
GO:0006120 mitochondrial electron transport, NADH to ubiquinone	P	1.54E-04	9/345 (2.6%)	34/14580 (0.2%)
GO:0006022 aminoglycan metabolic process	P	1.68E-04	17/345 (4.9%)	147/14580 (1.0%)
GO:0009119 ribonucleoside metabolic process	P	1.82E-04	24/345 (7.0%)	285/14580 (2.0%)
GO:0045214 sarcomere organization	P	2.30E-04	8/345 (2.3%)	26/14580 (0.2%)
GO:0009116 nucleoside metabolic process	P	3.48E-04	24/345 (7.0%)	295/14580 (2.0%)
GO:0006091 generation of precursor metabolites and energy	P	3.53E-04	18/345 (5.2%)	173/14580 (1.2%)
GO:1901657 glycosyl compound metabolic process	P	4.46E-04	24/345 (7.0%)	299/14580 (2.1%)
GO:0009150 purine ribonucleotide metabolic process	P	4.75E-04	24/345 (7.0%)	300/14580 (2.1%)
GO:0006163 purine nucleotide metabolic process	P	6.07E-04	24/345 (7.0%)	304/14580 (2.1%)
GO:0019693 ribose phosphate metabolic process	P	6.44E-04	24/345 (7.0%)	305/14580 (2.1%)
GO:0009259 ribonucleotide metabolic process	P	6.44E-04	24/345 (7.0%)	305/14580 (2.1%)
GO:0051146 striated muscle cell differentiation	P	9.44E-04	13/345 (3.8%)	96/14580 (0.7%)
GO:0072521 purine-containing compound metabolic process	P	1.83E-03	24/345 (7.0%)	323/14580 (2.2%)
GO:0042692 muscle cell differentiation	P	5.65E-03	13/345 (3.8%)	112/14580 (0.8%)
GO:0030029 actin filament-based process	P	6.41E-03	20/345 (5.8%)	254/14580 (1.7%)
GO:0007010 cytoskeleton organization	P	7.79E-03	36/345 (10.4%)	666/14580 (4.6%)
GO:0030036 actin cytoskeleton organization	P	9.92E-03	19/345 (5.5%)	239/14580 (1.6%)

Cellular Component

GO Term	Aspect	P-value	Sample frequency	Background frequency
GO:0044429 mitochondrial part	C	7.89E-20	52/345 (15.1%)	419/14580 (2.9%)
GO:0005739 mitochondrion	C	5.16E-19	63/345 (18.3%)	644/14580 (4.4%)
GO:0044455 mitochondrial membrane part	C	1.56E-14	28/345 (8.1%)	146/14580 (1.0%)
GO:0044391 ribosomal subunit	C	3.16E-14	30/345 (8.7%)	176/14580 (1.2%)
GO:0005761 mitochondrial ribosome	C	2.62E-13	21/345 (6.1%)	80/14580 (0.5%)
GO:0000313 organellar ribosome	C	2.62E-13	21/345 (6.1%)	80/14580 (0.5%)
GO:0005746 mitochondrial respiratory chain	C	2.17E-12	21/345 (6.1%)	88/14580 (0.6%)
GO:0070469 respiratory chain	C	2.17E-12	21/345 (6.1%)	88/14580 (0.6%)
GO:0005743 mitochondrial inner membrane	C	3.17E-12	29/345 (8.4%)	192/14580 (1.3%)
GO:0019866 organelle inner membrane	C	1.10E-11	29/345 (8.4%)	201/14580 (1.4%)
GO:0044444 cytoplasmic part	C	4.68E-11	115/345 (33.3%)	2439/14580 (16.7%)
GO:0044422 organelle part	C	6.80E-11	119/345 (34.5%)	2582/14580 (17.7%)
GO:0005763 mitochondrial small ribosomal subunit	C	3.18E-10	13/345 (3.8%)	32/14580 (0.2%)
GO:0000314 organellar small ribosomal subunit	C	3.18E-10	13/345 (3.8%)	32/14580 (0.2%)
GO:0044446 intracellular organelle part	C	3.72E-10	116/345 (33.6%)	2544/14580 (17.4%)
GO:0031966 mitochondrial membrane	C	8.34E-10	30/345 (8.7%)	254/14580 (1.7%)
GO:0015629 actin cytoskeleton	C	1.52E-09	20/345 (5.8%)	107/14580 (0.7%)
GO:0005740 mitochondrial envelope	C	2.08E-09	30/345 (8.7%)	263/14580 (1.8%)
GO:0044449 contractile fiber part	C	5.28E-09	14/345 (4.1%)	47/14580 (0.3%)
GO:0043292 contractile fiber	C	7.29E-09	14/345 (4.1%)	48/14580 (0.3%)
GO:0005759 mitochondrial matrix	C	8.74E-09	23/345 (6.7%)	160/14580 (1.1%)
GO:0005737 cytoplasm	C	1.03E-08	129/345 (37.4%)	3114/14580 (21.4%)

GO:0005840 ribosome	C	1.39E-08	30/345 (8.7%)	283/14580 (1.9%)
GO:0043228 non-membrane-bounded organelle	C	3.34E-08	84/345 (24.3%)	1685/14580 (11.6%)
GO:0043232 intracellular non-membrane-bounded organelle	C	3.34E-08	84/345 (24.3%)	1685/14580 (11.6%)
GO:0030016 myofibril	C	6.91E-08	13/345 (3.8%)	46/14580 (0.3%)
GO:0015934 large ribosomal subunit	C	1.65E-07	18/345 (5.2%)	108/14580 (0.7%)
GO:0005747 mitochondrial respiratory chain complex I	C	1.67E-07	13/345 (3.8%)	49/14580 (0.3%)
GO:0030964 NADH dehydrogenase complex	C	1.67E-07	13/345 (3.8%)	49/14580 (0.3%)
GO:0045271 respiratory chain complex I	C	1.67E-07	13/345 (3.8%)	49/14580 (0.3%)
GO:0015935 small ribosomal subunit	C	3.66E-07	15/345 (4.3%)	74/14580 (0.5%)
GO:0031967 organelle envelope	C	3.77E-07	32/345 (9.3%)	364/14580 (2.5%)
GO:0031975 envelope	C	4.04E-07	32/345 (9.3%)	365/14580 (2.5%)
GO:0030017 sarcomere	C	4.77E-07	12/345 (3.5%)	43/14580 (0.3%)
GO:0032991 macromolecular complex	C	2.66E-06	114/345 (33.0%)	2838/14580 (19.5%)
GO:1990204 oxidoreductase complex	C	1.71E-05	16/345 (4.6%)	111/14580 (0.8%)
GO:0016459 myosin complex	C	2.30E-05	9/345 (2.6%)	28/14580 (0.2%)
GO:0036379 myofilament	C	3.14E-05	6/345 (1.7%)	9/14580 (0.1%)
GO:0000315 organellar large ribosomal subunit	C	3.42E-05	11/345 (3.2%)	49/14580 (0.3%)
GO:0005762 mitochondrial large ribosomal subunit	C	3.42E-05	11/345 (3.2%)	49/14580 (0.3%)
GO:0031090 organelle membrane	C	4.54E-05	35/345 (10.1%)	514/14580 (3.5%)
GO:0005859 muscle myosin complex	C	1.00E-04	5/345 (1.4%)	6/14580 (0.0%)
GO:0043229 intracellular organelle	C	2.31E-04	149/345 (43.2%)	4369/14580 (30.0%)
GO:0005623 cell	C	4.42E-04	197/345 (57.1%)	6340/14580 (43.5%)
GO:0044464 cell part	C	4.42E-04	197/345 (57.1%)	6340/14580 (43.5%)
GO:0043226 organelle	C	5.31E-04	149/345 (43.2%)	4423/14580 (30.3%)
GO:0031012 extracellular matrix	C	6.70E-04	19/345 (5.5%)	200/14580 (1.4%)
GO:0070069 cytochrome complex	C	1.05E-03	8/345 (2.3%)	31/14580 (0.2%)
GO:0016460 myosin II complex	C	3.88E-03	5/345 (1.4%)	10/14580 (0.1%)
GO:0005751 mitochondrial respiratory chain complex IV	C	5.79E-03	6/345 (1.7%)	18/14580 (0.1%)
GO:0045277 respiratory chain complex IV	C	5.79E-03	6/345 (1.7%)	18/14580 (0.1%)
GO:0030529 ribonucleoprotein complex	C	6.50E-03	35/345 (10.1%)	633/14580 (4.3%)
GO:0044425 membrane part	C	8.46E-03	73/345 (21.2%)	1825/14580 (12.5%)

Molecular Function

GO Term	Aspect	P-value	Sample frequency	Background frequency
GO:0005198 structural molecule activity	F	4.74E-20	63/345 (18.3%)	616/14580 (4.2%)
GO:0003735 structural constituent of ribosome	F	5.00E-09	30/345 (8.7%)	272/14580 (1.9%)
GO:0016491 oxidoreductase activity	F	1.21E-07	45/345 (13.0%)	640/14580 (4.4%)
GO:0003954 NADH dehydrogenase activity	F	4.77E-07	12/345 (3.5%)	43/14580 (0.3%)
GO:0016651 oxidoreductase activity, acting on NAD(P)H	F	1.31E-06	13/345 (3.8%)	57/14580 (0.4%)
GO:0042302 structural constituent of cuticle	F	3.45E-05	18/345 (5.2%)	149/14580 (1.0%)
GO:0008061 chitin binding	F	8.49E-05	15/345 (4.3%)	108/14580 (0.7%)
GO:0005214 structural constituent of chitin-based cuticle	F	1.23E-04	17/345 (4.9%)	144/14580 (1.0%)
GO:0015078 hydrogen ion transmembrane transporter activity	F	2.50E-04	14/345 (4.1%)	101/14580 (0.7%)
GO:0050136 NADH dehydrogenase (quinone) activity	F	2.65E-04	9/345 (2.6%)	36/14580 (0.2%)
GO:0016655 oxidoreductase activity, acting on NAD(P)H, quinone or similar compound as acceptor	F	2.65E-04	9/345 (2.6%)	36/14580 (0.2%)
GO:0008137 NADH dehydrogenase (ubiquinone) activity	F	2.65E-04	9/345 (2.6%)	36/14580 (0.2%)
GO:0003779 actin binding	F	4.42E-04	15/345 (4.3%)	122/14580 (0.8%)
GO:0008010 structural constituent of chitin-based larval cuticle	F	6.58E-04	14/345 (4.1%)	109/14580 (0.7%)
GO:0005509 calcium ion binding	F	7.83E-04	19/345 (5.5%)	202/14580 (1.4%)

Commonality between all DE genes in Contrasts 2, 4 and 5 (high SBV)

Biological Process

GO Term	Aspect	P-value	Sample frequency	Background frequency
GO:0042335 cuticle development	P	2.25E-11	37/503 (7.4%)	234/14580 (1.6%)
GO:0040003 chitin-based cuticle development	P	1.67E-08	29/503 (5.8%)	182/14580 (1.2%)
GO:0007275 multicellular organismal development	P	7.48E-08	186/503 (37.0%)	3495/14580 (24.0%)
GO:0044707 single-multicellular organism process	P	9.15E-08	212/503 (42.1%)	4170/14580 (28.6%)
GO:0055114 oxidation-reduction process	P	1.37E-07	52/503 (10.3%)	552/14580 (3.8%)
GO:0032502 developmental process	P	3.15E-07	212/503 (42.1%)	4222/14580 (29.0%)
GO:0006022 aminoglycan metabolic process	P	4.01E-06	23/503 (4.6%)	147/14580 (1.0%)
GO:0044767 single-organism developmental process	P	4.25E-06	206/503 (41.0%)	4179/14580 (28.7%)
GO:0002376 immune system process	P	7.12E-06	40/503 (8.0%)	405/14580 (2.8%)
GO:0030239 myofibril assembly	P	7.13E-06	12/503 (2.4%)	37/14580 (0.3%)
GO:0065008 regulation of biological quality	P	1.20E-05	72/503 (14.3%)	1018/14580 (7.0%)
GO:1901071 glucosamine-containing compound metabolic pro	P	1.35E-05	21/503 (4.2%)	131/14580 (0.9%)
GO:0048856 anatomical structure development	P	1.43E-05	191/503 (38.0%)	3842/14580 (26.4%)
GO:0006040 amino sugar metabolic process	P	1.56E-05	21/503 (4.2%)	132/14580 (0.9%)

GO:0006030 chitin metabolic process	P	2.14E-05	20/503 (4.0%)	122/14580 (0.8%)
GO:1901564 organonitrogen compound metabolic process	P	2.87E-05	58/503 (11.5%)	757/14580 (5.2%)
GO:0055002 striated muscle cell development	P	4.08E-05	13/503 (2.6%)	51/14580 (0.3%)
GO:0055001 muscle cell development	P	4.08E-05	13/503 (2.6%)	51/14580 (0.3%)
GO:0032501 multicellular organismal process	P	1.64E-04	223/503 (44.3%)	4813/14580 (33.0%)
GO:0044699 single-organism process	P	1.94E-04	335/503 (66.6%)	8031/14580 (55.1%)
GO:0030029 actin filament-based process	P	1.98E-04	28/503 (5.6%)	254/14580 (1.7%)
GO:0030036 actin cytoskeleton organization	P	2.06E-04	27/503 (5.4%)	239/14580 (1.6%)
GO:0031032 actomyosin structure organization	P	3.14E-04	14/503 (2.8%)	70/14580 (0.5%)
GO:0006950 response to stress	P	7.59E-04	70/503 (13.9%)	1084/14580 (7.4%)
GO:0009888 tissue development	P	8.16E-04	83/503 (16.5%)	1372/14580 (9.4%)
GO:0050896 response to stimulus	P	1.46E-03	144/503 (28.6%)	2855/14580 (19.6%)
GO:1901615 organic hydroxy compound metabolic process	P	1.78E-03	19/503 (3.8%)	144/14580 (1.0%)
GO:0003012 muscle system process	P	2.24E-03	9/503 (1.8%)	31/14580 (0.2%)
GO:0065007 biological regulation	P	2.27E-03	173/503 (34.4%)	3621/14580 (24.8%)
GO:0006952 defense response	P	2.47E-03	33/503 (6.6%)	373/14580 (2.6%)
GO:0044710 single-organism metabolic process	P	2.73E-03	110/503 (21.9%)	2045/14580 (14.0%)
GO:0051146 striated muscle cell differentiation	P	3.21E-03	15/503 (3.0%)	96/14580 (0.7%)
GO:0009791 post-embryonic development	P	5.27E-03	51/503 (10.1%)	736/14580 (5.0%)
GO:0006955 immune response	P	5.35E-03	28/503 (5.6%)	298/14580 (2.0%)
GO:0018958 phenol-containing compound metabolic process	P	6.94E-03	13/503 (2.6%)	77/14580 (0.5%)
GO:0010259 multicellular organismal aging	P	8.17E-03	19/503 (3.8%)	159/14580 (1.1%)
GO:0048878 chemical homeostasis	P	8.70E-03	16/503 (3.2%)	117/14580 (0.8%)

Cellular Component

GO Term	Aspect	P-value	Sample frequency	Background frequency
GO:0044449 contractile fiber part	C	2.41E-15	21/503 (4.2%)	47/14580 (0.3%)
GO:0043292 contractile fiber	C	4.15E-15	21/503 (4.2%)	48/14580 (0.3%)
GO:0030016 myofibril	C	3.25E-14	20/503 (4.0%)	46/14580 (0.3%)
GO:0030017 sarcomere	C	3.29E-12	18/503 (3.6%)	43/14580 (0.3%)
GO:0005576 extracellular region	C	2.10E-10	79/503 (15.7%)	933/14580 (6.4%)
GO:0015629 actin cytoskeleton	C	5.57E-10	24/503 (4.8%)	107/14580 (0.7%)
GO:0031012 extracellular matrix	C	9.00E-07	28/503 (5.6%)	200/14580 (1.4%)
GO:0036379 myofilament	C	6.41E-06	7/503 (1.4%)	9/14580 (0.1%)
GO:0016459 myosin complex	C	5.55E-05	10/503 (2.0%)	28/14580 (0.2%)
GO:0031226 intrinsic component of plasma membrane	C	6.55E-05	30/503 (6.0%)	272/14580 (1.9%)
GO:0005865 striated muscle thin filament	C	9.47E-04	5/503 (1.0%)	6/14580 (0.0%)
GO:0005859 muscle myosin complex	C	9.47E-04	5/503 (1.0%)	6/14580 (0.0%)
GO:0044459 plasma membrane part	C	1.13E-03	43/503 (8.5%)	543/14580 (3.7%)
GO:0005887 integral component of plasma membrane	C	1.16E-03	27/503 (5.4%)	260/14580 (1.8%)
GO:0045177 apical part of cell	C	2.26E-03	16/503 (3.2%)	106/14580 (0.7%)

Molecular Function

GO Term	Aspect	P-value	Sample frequency	Background frequency
GO:0016491 oxidoreductase activity	F	1.46E-08	59/503 (11.7%)	640/14580 (4.4%)
GO:0042302 structural constituent of cuticle	F	5.26E-06	23/503 (4.6%)	149/14580 (1.0%)
GO:0005214 structural constituent of chitin-based cuticle	F	1.46E-05	22/503 (4.4%)	144/14580 (1.0%)
GO:0003779 actin binding	F	2.14E-05	20/503 (4.0%)	122/14580 (0.8%)
GO:0008061 chitin binding	F	9.42E-05	18/503 (3.6%)	108/14580 (0.7%)
GO:0005509 calcium ion binding	F	1.05E-04	25/503 (5.0%)	202/14580 (1.4%)
GO:0016918 retinal binding	F	1.62E-04	5/503 (1.0%)	5/14580 (0.0%)
GO:0043167 ion binding	F	4.17E-04	125/503 (24.9%)	2330/14580 (16.0%)
GO:0005539 glycosaminoglycan binding	F	4.60E-04	10/503 (2.0%)	34/14580 (0.2%)
GO:0008010 structural constituent of chitin-based larval cuticle	F	6.24E-04	17/503 (3.4%)	109/14580 (0.7%)
GO:0005488 binding	F	1.12E-03	236/503 (46.9%)	5276/14580 (36.2%)
GO:0003824 catalytic activity	F	2.36E-03	193/503 (38.4%)	4151/14580 (28.5%)
GO:0046872 metal ion binding	F	5.28E-03	80/503 (15.9%)	1367/14580 (9.4%)
GO:0005198 structural molecule activity	F	5.59E-03	45/503 (8.9%)	616/14580 (4.2%)
GO:0005501 retinoid binding	F	8.34E-03	5/503 (1.0%)	8/14580 (0.1%)
GO:0038024 cargo receptor activity	F	8.95E-03	9/503 (1.8%)	36/14580 (0.2%)
GO:0043169 cation binding	F	9.80E-03	80/503 (15.9%)	1389/14580 (9.5%)

Commonality between upregulated DE genes in Contrasts 2, 4 and 5 (high SBV)

Biological Process

GO Term	Aspect	P-value	Sample frequency	Background frequency
GO:0050896 response to stimulus	P	1.11E-10	104/263 (39.5%)	2855/14580 (19.6%)
GO:0006950 response to stress	P	3.34E-10	57/263 (21.7%)	1084/14580 (7.4%)

GO:0002376 immune system process	P	1.04E-09	33/263 (12.5%)	405/14580 (2.8%)
GO:0006952 defense response	P	9.74E-08	29/263 (11.0%)	373/14580 (2.6%)
GO:0044699 single-organism process	P	6.35E-07	194/263 (73.8%)	8031/14580 (55.1%)
GO:0006955 immune response	P	2.61E-06	24/263 (9.1%)	298/14580 (2.0%)
GO:0065007 biological regulation	P	5.27E-06	109/263 (41.4%)	3621/14580 (24.8%)
GO:0044763 single-organism cellular process	P	7.96E-06	154/263 (58.6%)	5930/14580 (40.7%)
GO:0045087 innate immune response	P	6.92E-05	16/263 (6.1%)	157/14580 (1.1%)
GO:0044707 single-multicellular organism process	P	2.72E-04	115/263 (43.7%)	4170/14580 (28.6%)
GO:0007275 multicellular organismal development	P	2.95E-04	101/263 (38.4%)	3495/14580 (24.0%)
GO:0010259 multicellular organismal aging	P	5.33E-04	15/263 (5.7%)	159/14580 (1.1%)
GO:0009611 response to wounding	P	5.40E-04	12/263 (4.6%)	98/14580 (0.7%)
GO:0032502 developmental process	P	5.57E-04	115/263 (43.7%)	4222/14580 (29.0%)
GO:0044710 single-organism metabolic process	P	6.59E-04	68/263 (25.9%)	2045/14580 (14.0%)
GO:0007568 aging	P	7.38E-04	15/263 (5.7%)	163/14580 (1.1%)
GO:0002682 regulation of immune system process	P	8.59E-04	16/263 (6.1%)	188/14580 (1.3%)
GO:0009888 tissue development	P	1.24E-03	51/263 (19.4%)	1372/14580 (9.4%)
GO:0050776 regulation of immune response	P	1.26E-03	13/263 (4.9%)	126/14580 (0.9%)
GO:0008340 determination of adult lifespan	P	2.53E-03	14/263 (5.3%)	156/14580 (1.1%)
GO:0050789 regulation of biological process	P	3.29E-03	94/263 (35.7%)	3328/14580 (22.8%)
GO:0018958 phenol-containing compound metabolic process	P	3.32E-03	10/263 (3.8%)	77/14580 (0.5%)
GO:0044767 single-organism developmental process	P	4.30E-03	111/263 (42.2%)	4179/14580 (28.7%)
GO:0006582 melanin metabolic process	P	4.42E-03	9/263 (3.4%)	62/14580 (0.4%)
GO:0006629 lipid metabolic process	P	4.43E-03	22/263 (8.4%)	380/14580 (2.6%)
GO:0050794 regulation of cellular process	P	6.06E-03	88/263 (33.5%)	3087/14580 (21.2%)
GO:0065008 regulation of biological quality	P	7.04E-03	40/263 (15.2%)	1018/14580 (7.0%)
GO:0055114 oxidation-reduction process	P	7.32E-03	27/263 (10.3%)	552/14580 (3.8%)
GO:0051179 localization	P	9.49E-03	67/263 (25.5%)	2155/14580 (14.8%)

Cellular Component

GO Term	Aspect	P-value	Sample frequency	Background frequency
GO:0005576 extracellular region	C	3.20E-05	43/263 (16.3%)	933/14580 (6.4%)
GO:0005811 lipid particle	C	4.05E-03	16/263 (6.1%)	211/14580 (1.4%)

Molecular Function

GO Term	Aspect	P-value	Sample frequency	Background frequency
GO:0005539 glycosaminoglycan binding	F	3.47E-04	8/263 (3.0%)	34/14580 (0.2%)
GO:0042834 peptidoglycan binding	F	1.43E-03	6/263 (2.3%)	18/14580 (0.1%)
GO:0003824 catalytic activity	F	1.58E-03	112/263 (42.6%)	4151/14580 (28.5%)
GO:0016491 oxidoreductase activity	F	4.70E-03	30/263 (11.4%)	640/14580 (4.4%)

Commonality between downpregulated DE genes in Contrasts 2, 4 and 5 (high SBV)

Biological Process

GO Term	Aspect	P-value	Sample frequency	Background frequency
**GO:0042335 cuticle development**	**P**	**3.78E-12**	**27/242 (11.2%)**	**234/14580 (1.6%)**
GO:0040003 chitin-based cuticle development	P	7.15E-11	23/242 (9.5%)	182/14580 (1.2%)
GO:0030239 myofibril assembly	P	8.95E-10	12/242 (5.0%)	37/14580 (0.3%)
GO:0055002 striated muscle cell development	P	6.24E-08	12/242 (5.0%)	51/14580 (0.3%)
GO:0055001 muscle cell development	P	6.24E-08	12/242 (5.0%)	51/14580 (0.3%)
GO:0031032 actomyosin structure organization	P	2.26E-07	13/242 (5.4%)	70/14580 (0.5%)
GO:0006030 chitin metabolic process	P	3.65E-07	16/242 (6.6%)	122/14580 (0.8%)
GO:1901071 glucosamine-containing compound metabolic pro	P	1.07E-06	16/242 (6.6%)	131/14580 (0.9%)
GO:0006040 amino sugar metabolic process	P	1.21E-06	16/242 (6.6%)	132/14580 (0.9%)
GO:0006022 aminoglycan metabolic process	P	5.98E-06	16/242 (6.6%)	147/14580 (1.0%)
GO:0030029 actin filament-based process	P	9.98E-05	19/242 (7.9%)	254/14580 (1.7%)
GO:0051146 striated muscle cell differentiation	P	1.27E-04	12/242 (5.0%)	96/14580 (0.7%)
GO:0030036 actin cytoskeleton organization	P	2.06E-04	18/242 (7.4%)	239/14580 (1.6%)
GO:0045214 sarcomere organization	P	3.35E-04	7/242 (2.9%)	26/14580 (0.2%)
GO:0042692 muscle cell differentiation	P	7.11E-04	12/242 (5.0%)	112/14580 (0.8%)
GO:0048856 anatomical structure development	P	1.02E-03	99/242 (40.9%)	3842/14580 (26.4%)
GO:0003012 muscle system process	P	1.25E-03	7/242 (2.9%)	31/14580 (0.2%)
GO:0007275 multicellular organismal development	P	1.34E-03	92/242 (38.0%)	3495/14580 (24.0%)
GO:0032502 developmental process	P	2.14E-03	105/242 (43.4%)	4222/14580 (29.0%)
GO:0044707 single-multicellular organism process	P	2.20E-03	104/242 (43.0%)	4170/14580 (28.6%)
GO:0010927 cellular component assembly involved in morpho	P	3.34E-03	15/242 (6.2%)	204/14580 (1.4%)
GO:1901564 organonitrogen compound metabolic process	P	6.45E-03	31/242 (12.8%)	757/14580 (5.2%)
GO:0044767 single-organism developmental process	P	8.88E-03	102/242 (42.1%)	4179/14580 (28.7%)
