# Supplementary material for: The Iflaviruses Sacbrood virus and Deformed wing virus evoke different transcriptional responses in the honeybee which may facilitate their horizontal or vertical transmission
Source: PeerJ. 2016 Jan 18;4:e1591. doi: 10.7717/peerj.1591 (PMC4727977; doi:10.7717/peerj.1591)
Supplement: Table S4 [file peerj-04-1591-s004.pdf]

Table S4

Larval feeding NGS experiments. Gene ontology (GO) terms associated with the genes upregulated in Contrast 3 (high DWV vs. control) and downregulated in Contrast 5 (high SBV+DWV vs. high DWV).

Biological Process

GO Term	Aspect	P-value	Sample frequency	Background frequency
GO:0046034 ATP metabolic process	P	4.21E-07	9/60 (15.0%)	108/14580 (0.7%)
GO:0009205 purine ribonucleoside triphosphate metabolic process	P	5.84E-07	9/60 (15.0%)	112/14580 (0.8%)
GO:0009144 purine nucleoside triphosphate metabolic process	P	5.84E-07	9/60 (15.0%)	112/14580 (0.8%)
GO:0009199 ribonucleoside triphosphate metabolic process	P	6.85E-07	9/60 (15.0%)	114/14580 (0.8%)
GO:0009141 nucleoside triphosphate metabolic process	P	8.00E-07	9/60 (15.0%)	116/14580 (0.8%)
GO:0006412 translation	P	1.10E-06	18/60 (30.0%)	787/14580 (5.4%)
GO:0009126 purine nucleoside monophosphate metabolic process	P	1.17E-06	9/60 (15.0%)	121/14580 (0.8%)
GO:0009167 purine ribonucleoside monophosphate metabolic process	P	1.17E-06	9/60 (15.0%)	121/14580 (0.8%)
GO:0009161 ribonucleoside monophosphate metabolic process	P	1.80E-06	9/60 (15.0%)	127/14580 (0.9%)
GO:0009123 nucleoside monophosphate metabolic process	P	1.93E-06	9/60 (15.0%)	128/14580 (0.9%)
GO:0046128 purine ribonucleoside metabolic process	P	4.26E-06	9/60 (15.0%)	140/14580 (1.0%)
GO:0042278 purine nucleoside metabolic process	P	4.26E-06	9/60 (15.0%)	140/14580 (1.0%)
GO:0009119 ribonucleoside metabolic process	P	7.82E-06	9/60 (15.0%)	150/14580 (1.0%)
GO:0042775 mitochondrial ATP synthesis coupled electron transport	P	1.11E-05	7/60 (11.7%)	70/14580 (0.5%)
GO:0009116 nucleoside metabolic process	P	1.38E-05	9/60 (15.0%)	160/14580 (1.1%)
GO:0042773 ATP synthesis coupled electron transport	P	1.65E-05	7/60 (11.7%)	74/14580 (0.5%)
GO:1901657 glycosyl compound metabolic process	P	1.71E-05	9/60 (15.0%)	164/14580 (1.1%)
GO:0009150 purine ribonucleotide metabolic process	P	2.21E-05	9/60 (15.0%)	169/14580 (1.2%)
GO:0044237 cellular metabolic process	P	2.60E-05	40/60 (66.7%)	4646/14580 (31.9%)
GO:0006163 purine nucleotide metabolic process	P	2.71E-05	9/60 (15.0%)	173/14580 (1.2%)
GO:0006119 oxidative phosphorylation	P	2.86E-05	7/60 (11.7%)	80/14580 (0.5%)
GO:0009259 ribonucleotide metabolic process	P	3.00E-05	9/60 (15.0%)	175/14580 (1.2%)
GO:0022904 respiratory electron transport chain	P	3.12E-05	7/60 (11.7%)	81/14580 (0.6%)
GO:0019693 ribose phosphate metabolic process	P	4.41E-05	9/60 (15.0%)	183/14580 (1.3%)
GO:0022900 electron transport chain	P	5.14E-05	7/60 (11.7%)	87/14580 (0.6%)
GO:0072521 purine-containing compound metabolic process	P	6.67E-05	9/60 (15.0%)	192/14580 (1.3%)
GO:0071704 organic substance metabolic process	P	4.01E-04	41/60 (68.3%)	5310/14580 (36.4%)
GO:0009117 nucleotide metabolic process	P	4.13E-04	9/60 (15.0%)	238/14580 (1.6%)
GO:0006753 nucleoside phosphate metabolic process	P	4.59E-04	9/60 (15.0%)	241/14580 (1.7%)
GO:0045333 cellular respiration	P	4.98E-04	7/60 (11.7%)	121/14580 (0.8%)
GO:0015980 energy derivation by oxidation of organic compounds	P	9.93E-04	7/60 (11.7%)	134/14580 (0.9%)
GO:0055086 nucleobase-containing small molecule metabolic process	P	1.34E-03	9/60 (15.0%)	274/14580 (1.9%)
GO:0044238 primary metabolic process	P	1.91E-03	38/60 (63.3%)	4903/14580 (33.6%)
GO:0019637 organophosphate metabolic process	P	2.14E-03	10/60 (16.7%)	374/14580 (2.6%)
GO:0044249 cellular biosynthetic process	P	2.96E-03	25/60 (41.7%)	2418/14580 (16.6%)
GO:0044267 cellular protein metabolic process	P	3.01E-03	23/60 (38.3%)	2086/14580 (14.3%)
GO:1901576 organic substance biosynthetic process	P	3.44E-03	25/60 (41.7%)	2438/14580 (16.7%)
GO:0008152 metabolic process	P	3.92E-03	42/60 (70.0%)	5968/14580 (40.9%)
GO:0034645 cellular macromolecule biosynthetic process	P	5.35E-03	22/60 (36.7%)	1993/14580 (13.7%)
GO:0009058 biosynthetic process	P	5.35E-03	25/60 (41.7%)	2497/14580 (17.1%)
GO:0006091 generation of precursor metabolites and energy	P	5.44E-03	7/60 (11.7%)	173/14580 (1.2%)
GO:0009059 macromolecule biosynthetic process	P	5.76E-03	22/60 (36.7%)	2002/14580 (13.7%)
GO:0006120 mitochondrial electron transport, NADH to ubiquinone	P	8.93E-03	4/60 (6.7%)	34/14580 (0.2%)

Cellular Component

GO Term	Aspect	P-value	Sample frequency	Background frequency
GO:0044429 mitochondrial part	C	1.38E-17	23/60 (38.3%)	419/14580 (2.9%)
GO:0044391 ribosomal subunit	C	2.25E-16	17/60 (28.3%)	176/14580 (1.2%)
GO:0005739 mitochondrion	C	1.31E-14	24/60 (40.0%)	644/14580 (4.4%)
GO:0032991 macromolecular complex	C	1.97E-14	42/60 (70.0%)	2838/14580 (19.5%)
GO:0044446 intracellular organelle part	C	3.14E-14	40/60 (66.7%)	2544/14580 (17.4%)
GO:0044422 organelle part	C	5.37E-14	40/60 (66.7%)	2582/14580 (17.7%)
GO:0005761 mitochondrial ribosome	C	2.91E-13	12/60 (20.0%)	80/14580 (0.5%)
GO:0000313 organellar ribosome	C	2.91E-13	12/60 (20.0%)	80/14580 (0.5%)
GO:0005840 ribosome	C	7.27E-13	17/60 (28.3%)	283/14580 (1.9%)
GO:0000314 organellar small ribosomal subunit	C	3.84E-12	9/60 (15.0%)	32/14580 (0.2%)
GO:0005763 mitochondrial small ribosomal subunit	C	3.84E-12	9/60 (15.0%)	32/14580 (0.2%)
GO:0044444 cytoplasmic part	C	3.92E-11	36/60 (60.0%)	2439/14580 (16.7%)
GO:0005759 mitochondrial matrix	C	1.46E-09	12/60 (20.0%)	160/14580 (1.1%)
GO:0030529 ribonucleoprotein complex	C	3.14E-09	19/60 (31.7%)	633/14580 (4.3%)
GO:0043229 intracellular organelle	C	4.03E-09	44/60 (73.3%)	4369/14580 (30.0%)
GO:0043226 organelle	C	6.40E-09	44/60 (73.3%)	4423/14580 (30.3%)
GO:0005737 cytoplasm	C	1.26E-08	37/60 (61.7%)	3114/14580 (21.4%)
GO:0044455 mitochondrial membrane part	C	1.27E-08	11/60 (18.3%)	146/14580 (1.0%)
GO:0015935 small ribosomal subunit	C	1.33E-08	9/60 (15.0%)	74/14580 (0.5%)
GO:0015934 large ribosomal subunit	C	1.47E-08	10/60 (16.7%)	108/14580 (0.7%)
GO:0044424 intracellular part	C	9.02E-08	47/60 (78.3%)	5457/14580 (37.4%)
GO:0005622 intracellular	C	1.70E-07	47/60 (78.3%)	5545/14580 (38.0%)
GO:0005743 mitochondrial inner membrane	C	2.47E-07	11/60 (18.3%)	192/14580 (1.3%)
GO:0043228 non-membrane-bounded organelle	C	4.00E-07	26/60 (43.3%)	1685/14580 (11.6%)
GO:0043232 intracellular non-membrane-bounded organelle	C	4.00E-07	26/60 (43.3%)	1685/14580 (11.6%)
GO:0019866 organelle inner membrane	C	4.03E-07	11/60 (18.3%)	201/14580 (1.4%)
GO:0043231 intracellular membrane-bounded organelle	C	8.22E-07	37/60 (61.7%)	3569/14580 (24.5%)
GO:0043227 membrane-bounded organelle	C	8.57E-07	37/60 (61.7%)	3574/14580 (24.5%)
GO:0070469 respiratory chain	C	2.06E-06	8/60 (13.3%)	88/14580 (0.6%)
GO:0005746 mitochondrial respiratory chain	C	2.06E-06	8/60 (13.3%)	88/14580 (0.6%)
GO:0031966 mitochondrial membrane	C	4.75E-06	11/60 (18.3%)	254/14580 (1.7%)
GO:0005740 mitochondrial envelope	C	6.83E-06	11/60 (18.3%)	263/14580 (1.8%)
GO:0043233 organelle lumen	C	8.49E-06	17/60 (28.3%)	788/14580 (5.4%)
GO:0070013 intracellular organelle lumen	C	8.49E-06	17/60 (28.3%)	788/14580 (5.4%)
GO:0031974 membrane-enclosed lumen	C	1.01E-05	17/60 (28.3%)	797/14580 (5.5%)
GO:0044464 cell part	C	3.00E-05	47/60 (78.3%)	6340/14580 (43.5%)
GO:0005623 cell	C	3.00E-05	47/60 (78.3%)	6340/14580 (43.5%)
GO:0031090 organelle membrane	C	1.03E-04	13/60 (21.7%)	514/14580 (3.5%)
GO:0031967 organelle envelope	C	1.89E-04	11/60 (18.3%)	364/14580 (2.5%)
GO:0031975 envelope	C	1.94E-04	11/60 (18.3%)	365/14580 (2.5%)
GO:1990204 oxidoreductase complex	C	2.77E-04	7/60 (11.7%)	111/14580 (0.8%)
GO:0005747 mitochondrial respiratory chain complex I	C	1.35E-03	5/60 (8.3%)	49/14580 (0.3%)
GO:0030964 NADH dehydrogenase complex	C	1.35E-03	5/60 (8.3%)	49/14580 (0.3%)
GO:0045271 respiratory chain complex I	C	1.35E-03	5/60 (8.3%)	49/14580 (0.3%)
GO:0005762 mitochondrial large ribosomal subunit	C	1.35E-03	5/60 (8.3%)	49/14580 (0.3%)
GO:0000315 organellar large ribosomal subunit	C	1.35E-03	5/60 (8.3%)	49/14580 (0.3%)
GO:0022625 cytosolic large ribosomal subunit	C	3.43E-03	5/60 (8.3%)	59/14580 (0.4%)
GO:0043234 protein complex	C	5.88E-03	23/60 (38.3%)	2169/14580 (14.9%)

Molecular Function

GO Term	Aspect	P-value	Sample frequency	Background frequency
GO:0003735 structural constituent of ribosome	F	3.75E-13	17/60 (28.3%)	272/14580 (1.9%)
GO:0005198 structural molecule activity	F	2.06E-08	18/60 (30.0%)	616/14580 (4.2%)
