# Supplementary material for: The Iflaviruses Sacbrood virus and Deformed wing virus evoke different transcriptional responses in the honeybee which may facilitate their horizontal or vertical transmission
Source: PeerJ. 2016 Jan 18;4:e1591. doi: 10.7717/peerj.1591 (PMC4727977; doi:10.7717/peerj.1591)
Supplement: Table S5 [file peerj-04-1591-s005.pdf]

Table S5.
Honeybee immune-related genes differentially expressed in response to SBV and DWV in oral larvae inoculation experiment. Fold change values (log2 transformed) are shown only for the genes DE in the contrasts. Expression of the genes marked with * was quantified by qRT-PCR. DE genes were identified by both DEseq and edgeR and analyses, adjusted p<0.05 and false discovery rate, FDR <0.05.

Honeybee gene (OGS3 ID)	Drosophila ortholog (Flybase ID)	Gene name / description	Pathway, group	Fold change (log2 transformed)			
				Contrast 2	Contrast 3	Contrast 4	Contrast 5
				High SBV+DWV vs. high DWV and control	High DWV vs. control	High SBV+DWV vs. control	High SBV+DWV vs. high DWV
GB41428 *	FBgn0010385	Defensin-1	AMP	9.203	.	9.328	8.738
GB47318	FBgn0032835	Abaecin	AMP	6.474	.	6.143	10.455
GB47546		Apidaecin	AMP	5.322	.	5.423	4.925
GB47618	FBgn0010385	Defensin-2	AMP	10.171	.	9.828	10.730
GB51223 *	FBgn0014002	Hymenoptaecin	AMP	7.894	.	8.057	7.410
GB53576	FBgn0261922	Apisimin	AMP	.	.	2.738	.
GB50013 *	FBgn0036891	Prophenoloxidase-activating enzyme (PPAE)	Melanisation	-2.612	.	-2.791	.
GB48820 *	FBgn0028985	Serpin (NEC LIKE)	Toll / Melanisation	4.681	.	4.867	4.151
GB54611	FBgn0028984	Serpin (NEC LIKE)	Toll / Melanisation	2.092	.	2.027	2.359
GB40699	FBgn0029114	Tollo (Receptor)	Toll	.	.	-1.187	.
GB43456	FBgn0034476	Toll-7 (Receptor)	Toll	-1.681	.	-1.780	.
GB49441	FBgn0003450	persephone-Serine protease	Toll	4.182	.	4.134	4.365
GB54611	FBgn0028984	NEC-like	Toll	2.092	.	2.027	2.359
GB55007	FBgn0030051	persephone-Serine Protease	Toll	2.067	.	1.975	.
GB44055	FBgn0000250	Immune Response Integrator cactus (NF-kappa-B inhibitor)	Toll	.	.	2.372	2.457
GB50418	FBgn0262473	Toll-1 (Receptor)	Toll	2.073	.	2.104	1.962
GB51741	FBgn0030310	Peptidoglycan recognition protein SA	Toll	2.070	.	2.056	2.119
GB52631	FBgn0003495	spatzle	Toll	3.224	.	3.284	3.012
GB51498	FBgn0033402	Myd88	Toll	.	1.549	.	.
GB48707	FBgn0024222	immune response deficient 5	Toll	.	1.340	.	.
GB42500	FBgn0035976	PGRP-LC	Imd	1.515	.	1.462	1.723
GB45648	FBgn0013983	imd	Imd	.	.	1.240	.
GB40445	FBgn0031918	CTL5	C-lectin domain	-1.966	.	-2.041	-1.652
GB43691	FBgn0038017	CTL11	C-lectin domain	-3.657	.	-3.697	-3.490
GB43983	FBgn0030617	CTL12	C-lectin domain	-3.169	.	-3.259	-2.772
GB45248	FBgn0032180	CTL2	C-lectin domain	2.684	.	2.605	3.014
GB46626	FBgn0035199	CTL3	C-lectin domain	.	.	-1.637	.
GB47938	FBgn0031879	CTL4	C-lectin domain	-2.013	.	-2.112	-1.580
GB49260	FBgn0262720	CTL1	C-lectin domain	.	.	.	1.605
GB55225	FBgn0001083	CTL6	C-lectin domain	-3.050	.	-3.096	-2.865
GB47977	FBgn0086906		IG Superfamily Genes	-2.984	.	-3.124	-2.315
GB49767	FBgn0263219		IG Superfamily Genes	.	.	-2.263	.
GB53012	FBgn0010473		IG Superfamily Genes	-1.922	.	-2.073	.
GB55483	FBgn0005666		IG Superfamily Genes	-2.097	.	-2.276	.
GB40741	FBgn0052133	ptip	Immune system process	.	.	-1.324	.
GB40759	FBgn0043841	virus-induced RNA 1	Immune system process	2.053	.	1.940	2.567
GB41629	FBgn0025936	Eph receptor tyrosine kinase	Immune system process	.	.	-1.204	.
GB41740	FBgn0021760	chromosome bows	Immune system process	.	.	-1.231	.
GB41850	FBgn0029167	Hemolectin	Immune system process	-1.549	.	-1.573	.
GB42579	FBgn0262738	no receptor potential A	Immune system process	.	.	-1.184	.
GB42608	FBgn0264294	Cytochrome b5	Immune system process	1.439	.	1.379	.
GB42981	FBgn0040323	Gram-negative bacteria binding protein 1	Immune system process	2.806	.	2.834	2.707
GB43099	FBgn0028982	Spt6	Immune system process	.	.	-1.252	.
GB44213	FBgn0014141	cheerio	Immune system process	2.110	.	1.961	2.836
GB44931	FBgn0028436	ECSIT	Immune system process	.	.	.	-2.668
GB45135	FBgn0028982	Spt6	Immune system process	1.704	.	1.693	1.742
GB45708	FBgn0031975	Transglutaminase	Immune system process	5.015	.	4.758	6.397
GB46165	FBgn0010389	heartless	Immune system process	.	.	-1.939	.
GB46302	FBgn0262738	no receptor potential A	Immune system process	-2.634	.	-2.761	.
GB46338	FBgn0043550	Tetraspanin 68C	Immune system process	.	.	-1.593	.
GB47201	FBgn0004657	myospheroid	Immune system process	1.406	.	1.338	1.686
GB47224	FBgn0000568	Ecdysone-induced protein 75B	Immune system process	-2.831	.	-2.951	-2.280
GB47805	FBgn0043575	PGRP-SC2	Immune system process	4.172	.	3.907	5.989
GB49657	FBgn0004606	Zn finger homeodomain 1	Immune system process	1.538	.	1.430	2.020
GB49949	FBgn0035850	Autophagy-specific gene 18	Immune system process	.	.	1.067	.
GB49979	FBgn0043550	Tetraspanin 68C	Immune system process	-4.806	.	-4.904	.
GB51481	FBgn0031464	Dual oxidase	Immune system process	-1.559	.	-1.552	.
GB51962	FBgn0086902	kismet	Immune system process	.	.	-1.377	.
GB52158	FBgn0031975	Transglutaminase	Immune system process	1.764	.	1.769	1.743
GB52278	FBgn0014141	cheerio	Immune system process	1.804	.	1.670	2.434
GB52721	FBgn0002576	lozenge	Immune system process	4.204	.	4.310	3.850
GB54764	FBgn0026404	Nedd2-like caspase	Immune system process	.	.	1.208	.
GB54852	FBgn0032006	PDGF- and VEGF-receptor related	Immune system process	.	.	1.070	.
GB55808	FBgn0020306		Immune system process	-1.665	.	-1.737	.
GB42358	FBgn0087011		immune-related	-4.576	.	-4.604	-4.458

GB42685	FBgn0040323		immune-related	1.502	.	1.390	2.001
GB48687	FBgn0243514		immune-related	3.132	.	2.992	3.798
GB50507	FBgn0261514		immune-related	.	.	-2.137	.
GB50508	FBgn0243514		immune-related	2.448	.	2.326	3.007
GB42244	FBgn0043903	domeless	JAK-STAT cascade	3.878	.	3.816	4.123
GB52985	FBgn0043884	multiple ankyrin repeats single KH domain	JAK-STAT cascade	.	.	-1.286	.
GB44634	FBgn0243512		JNK cascade	3.327	.	3.329	3.306
GB45540	FBgn0000097	anterior open	JNK cascade	-1.634	.	-1.698	.
GB53318	FBgn0001291	Jun-related antigen	JNK cascade	2.851	.	2.812	2.985
GB47104	FBgn0034162		Lysozyme	2.259	.	2.293	2.141
GB40306	FBgn0010435	AmSCR-B8	Scav. Receptor A	-2.763	.	-2.873	.
GB44045	FBgn0015924	AmSCR-B9	Scav. Receptor A	2.391	.	1.520	.
GB50866	FBgn0010435	AmSCR-B10	Scav. Receptor A	-2.657	.	-2.733	-2.338
GB52097	FBgn0058006	AmSCR-B1	Scav. Receptor A	.	.	-1.967	.
GB40137	FBgn0038595	SP17	serine protease	5.775	.	5.939	5.248
GB41097	FBgn0051954	SP18	serine protease	7.724	.	7.382	8.286
GB41178	FBgn0038727	SP15	serine protease	-4.149	.	-4.257	.
GB42804	FBgn0033192	SPH54	serine protease	-2.412	.	-2.515	-1.956
GB44146	FBgn0034709	0	serine protease	4.144	.	3.943	5.176
GB45700	FBgn0000533	cSP2	serine protease	2.153	.	2.055	2.585
GB45701	FBgn0027930	cSP1	serine protease	2.852	.	2.748	3.305
GB48079	FBgn0051954	SP22	serine protease	4.075	.	4.578	.
GB49552	FBgn0035501	cSP3	serine protease	4.555	.	4.601	4.373
GB49946	FBgn0011653	cSPH41	serine protease	-2.974	.	-3.070	.
GB50290	FBgn0033033	cSPH55	serine protease	2.937	.	2.878	3.172
GB50586	FBgn0003319	SP16	serine protease	-1.651	.	-1.728	.
GB50648	FBgn0003319	cSP25	serine protease	-2.817	.	-2.939	.
GB50650	FBgn0033359	cSP33	serine protease	-4.769	.	-4.846	-4.418
GB50761	FBgn0038485	SP36	serine protease	3.308	.	3.258	3.474
GB52191	FBgn0051217	SP49	serine protease	2.432	.	2.455	2.350
GB52598	FBgn0032213	cSPH42	serine protease	4.552	.	4.486	4.821
GB54762	FBgn0033362	cSP6	serine protease	-3.142	.	-3.213	.
GB54775	FBgn0030027	SPH56	serine protease	-3.193	.	-3.241	-2.997
