# Supplementary material for: The Iflaviruses Sacbrood virus and Deformed wing virus evoke different transcriptional responses in the honeybee which may facilitate their horizontal or vertical transmission
Source: PeerJ. 2016 Jan 18;4:e1591. doi: 10.7717/peerj.1591 (PMC4727977; doi:10.7717/peerj.1591)

Figure S1. Bacterial load in the experimental pupae. ΔCt values were obtained by subtracting the Ct values for the honeybee rp49 gene from the Ct values for bacterial 16s rRNA.

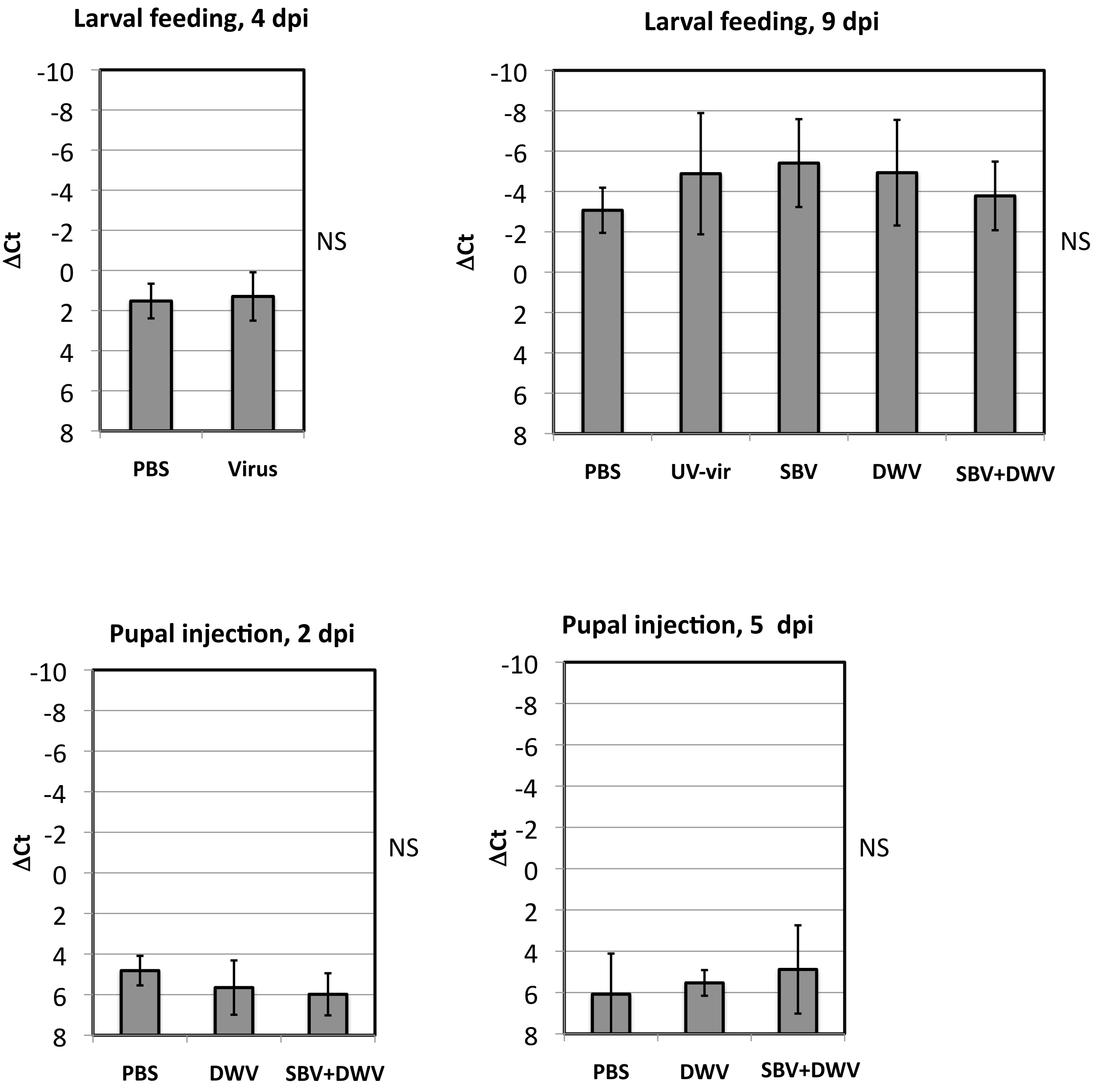

Supplement: Figure S1 [file peerj-04-1591-s006.pdf]
